# Ruthenaelectro-catalyzed C–H phosphorylation: *ortho* to *para* position-selectivity switch†

**DOI:** 10.1039/d4sc06219a

**Published:** 2024-11-29

**Authors:** Xue-Ya Gou, João C. A. Oliveira, Shan Chen, Simon L. Homölle, Sven Trienes, Tristan von Münchow, Bo-Sheng Zhang, Lutz Ackermann

**Affiliations:** a Wöhler Research Institute for Sustainable Chemistry (WISCh), Georg-August-Universität Tammannstraße 2 37077 Göttingen Germany Lutz.Ackermann@chemie.uni-goettingen.de

## Abstract

The position-selective C–H bond activation of arenes has long been a challenging topic. Herein, we report an expedient ruthenium-electrocatalyzed site-selective *ortho*-C–H phosphorylation of arenes driven by electrochemical hydrogen evolution reaction (HER), avoiding stoichiometric amounts of chemical redox-waste products. This strategy paved the way to achieve unprecedented ruthenaelectro-catalyzed *para*-C–H phosphorylation with excellent levels of site-selectivity. This electrocatalytic approach was characterized by an ample substrate scope with a broad range of arenes containing N-heterocycles, as well as several aryl/alkylphosphine oxides were well tolerated. Moreover, late-stage C–H phosphorylation of medicinal relevant drugs could also be achieved. DFT mechanistic studies provided support for an unusual ruthenium(iii/iv/ii) regime for the *ortho*-C–H phosphorylation.

## Abstract 2

Sustainable ruthenaelectro-catalyzed position-selective *ortho*- and *para*-C–H phosphorylation of arenes by HER enabled late-stage functionalization, and the catalyst's mode of action was unraveled through experimental and computational studies.

## Graphical abstract

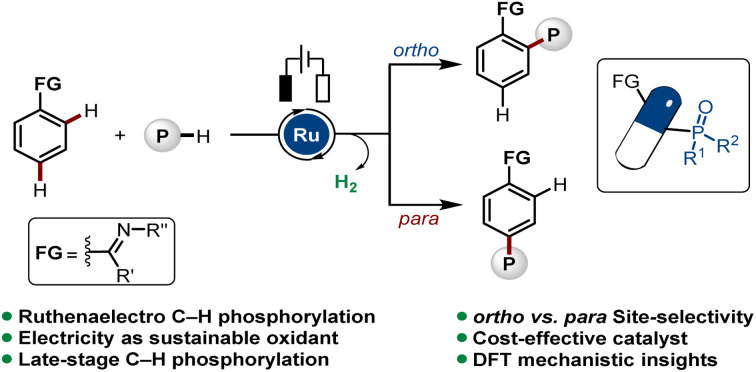

## Introduction

**Scheme 1:**
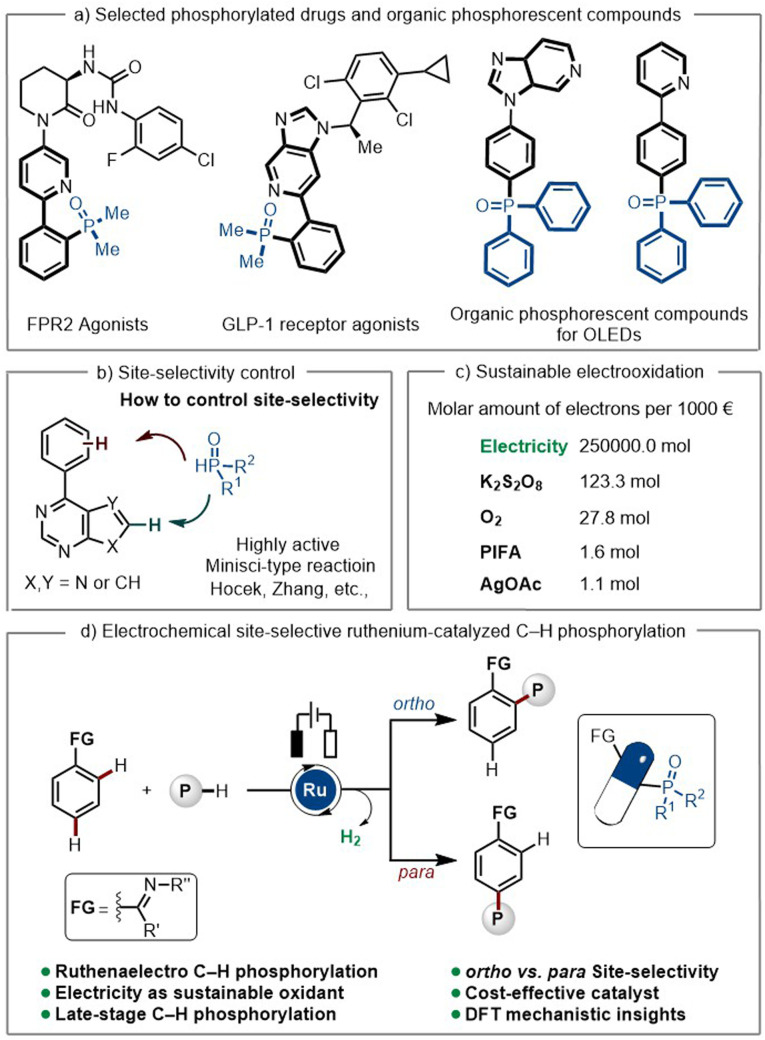
Electrochemical ruthenium-catalyzed site-selective C–H phosphorylation.

Organophosphorus compounds have been widely employed in organic synthesis,^1^ medicinal chemistry,^2^ material sciences,^3^ as well as prominent ligands^4^ in catalytic reactions. The presence of a phosphoryl group in target molecules has often been reported to enhance their physical-chemical properties, such as their hydrophilicity, thereby improving their solubility and tolerance in biological systems.^5^ Compounds bearing phosphine oxide motifs have revealed potential in decreasing inflammation, reducing blood sugar, and even anti-HIV activity.^6^ Additionally, phosphorylated heteroarenes have been reported as the one of the main components in phosphorescent OLEDs, due to their excellent luminescence properties (Scheme 1a).^7^ In this context, the development of novel and efficient strategies for the construction of aromatic C–P bonds poses great significance.

Transition metal-catalyzed C–H activation has emerged as a powerful tool in modern synthesis.^8^ Direct C–H phosphorylation of arenes has proven to be challenging due to the strong coordination ability of the phosphine reagents, which often leads to catalyst deactivation. To overcome this drawback, distinct strategies have been developed. These involve the slow addition of the phosphine reagents,^9^ the use of masked phosphine reagents, which slowly release the active phosphine compounds^10^ or their sequential addition.^11^ However, these approaches involve the use of stoichiometric amounts of chemical oxidants, which strongly jeopardizes the sustainability of the overall approaches. On a different note, phosphine radical-based strategies for the synthesis of aryl phosphonyl compounds have been limited to electron-rich arenes, with poor position-selectivity.^12^ Furthermore, Minisci-type reactivity of nitrogen-containing heterocycles based on phosphine radicals is predominant,^13^ rendering phosphorylation at remote position difficult.

Ruthenium catalysis has surfaced as a uniquely versatile platform for proximal and distal bond functionalizations.^14–16^ Hence, we wondered whether position-selective ruthenium-catalyzed C–H phosphorylation would be viable in a position-selectivity-divergent manner by the judicious choice of the reaction conditions. The emergence of electrochemistry applied to organic synthesis has strongly revolutionized molecular synthesis by avoiding the use of chemical oxidants, leading to more sustainable and environmentally friendly synthetic routes,^17^ such as in transition metal-catalyzed C–H activation.^18–23^

Electrochemically^24^ driven *ortho*-C–H phosphorylation has solely been accomplished with expensive rhodium catalysts^25^ or through nickel catalysis, employing high-temperature conditions (110 °C, DG = 8-aminoquinoline).^26^ In sharp contrast, studies on metalla-electrocatalyzed C–H phosphorylations with versatile ruthenium catalysts have thus far proven elusive. Herein, we report a mild, electrochemically driven and cost-effective position-selective ruthenaelectro-catalyzed C–H phosphorylation with controlled position-switch from *ortho* to *para*. Moreover, phosphonyl units could be successfully introduced into relevant pharmaceutical compounds *via* late-stage C–H phosphorylation to access structurally diverse active compounds in a single step.

## Results and discussion

### Reaction optimization of *ortho*-C–H phosphorylation

We initiated our studies by exploring the envisioned ruthenium-electrocatalyzed *ortho*-C–H phosphorylation of arene 1a and diphenylphosphine oxide 2a using a graphite felt (GF) and a platinum (Pt) electrode as anode and cathode materials, respectively, in an undivided cell setup (Table 1). The use of [Ru(OAc)_2_(*p*-cymene)] as the catalyst, in the presence of HFIP as the solvent, led to the formation of the desired phosphorylated product 3a, with complete *ortho*-selectivity, in 73% isolated yield (entry 1). Several other solvents and bases were also considered, but significantly reduced efficacy was noted (entries 2–3). Furthermore, other transition metal catalysts, such as Co(OAc)_2_·4H_2_O, Cu(OAc)_2_ or Ni(DME)Cl_2_, gave unsatisfactory results (entry 4). Control experiments demonstrated that electricity is crucial for the C–H phosphorylation (entry 6).

**Table 1:** Optimization of the *ortho*-C–H phosphorylation reaction conditions^a^

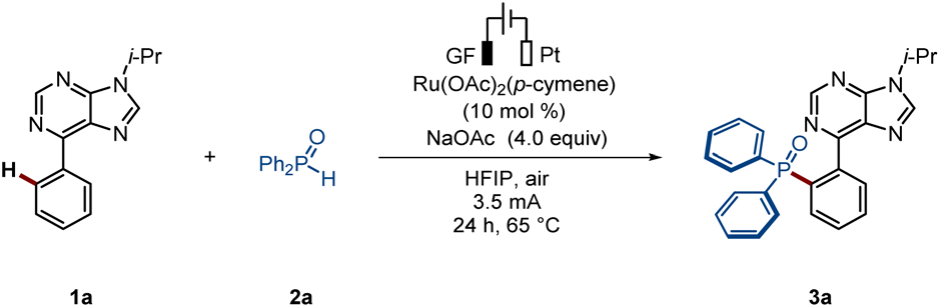		
Entry	Deviation from standard conditions	Yield of 3a^b^(%)
1	None	73
2	H_2_O/CH_3_CN/TFE as solvent	−/−/65
3	Na_2_CO_3_/Na_3_PO_4_/NaOPiv as base	62/66/53
4^c^	Co(OAc)_2_·4H_2_O/Cu(OAc)_2_/Ni(DME)Cl_2_	Trace/0/0
5	N_2_ instead of air	72
6	Without electricity	0

a Reaction conditions A: undivided cell, GF anode, Pt cathode, 1a (0.3 mmol), 2a (4.0 equiv.), [Ru(OAc)_2_(*p*-cymene)] (10 mol%), NaOAc (4.0 equiv.), HFIP: 1,1,1,3,3,3-hexafluoropropan-2-ol (3.0 mL), air, 65 °C, 3.5 mA, 24 h.

b Isolated yields.

c Co(OAc)_2_·4H_2_O (10 mol%)/Cu(OAc)_2_ (10 mol%)/Ni(DME)Cl_2_ (10 mol%).

### Substrate scope investigation for the *ortho*-C–H phosphorylation

**Scheme 2:**
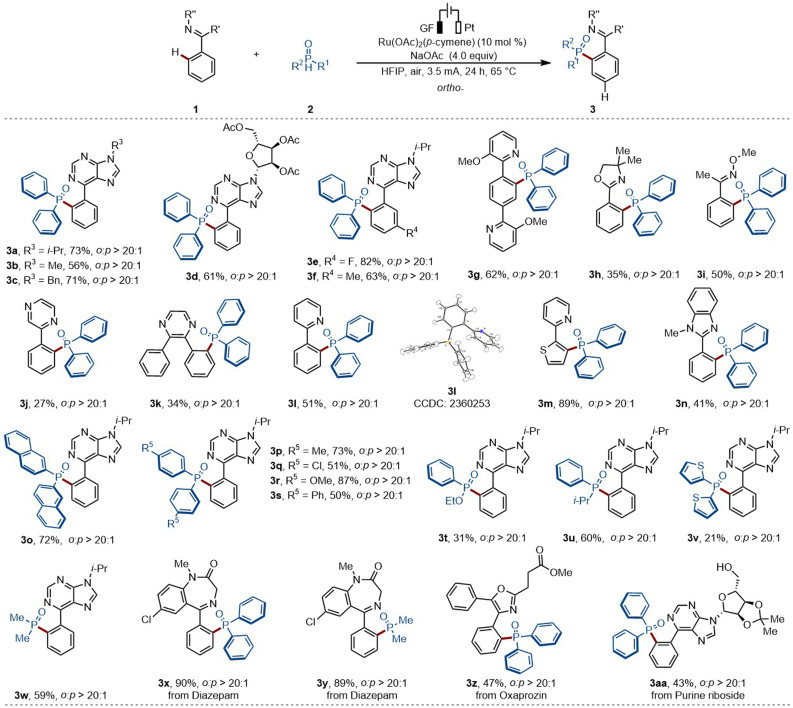
Scope of the ortho-selective C–H phosphorylation.

With the optimized reaction conditions in hand, we first investigated the viable scope of the ruthenium-electrocatalyzed *ortho*-C–H phosphorylation (Scheme 2). First, various *N*-substituted arylpurines were probed, including bio-relevant 6-phenylpurine nucleosides, efficiently affording the desired products in good yields (3a–3d). Additionally, both electron-withdrawing groups (*e.g.*, –F) and electron-donating groups (*e.g.*, –Me) on the arenes were well tolerated (3e–3f). Second, a bipyridine-substituted arene was also tolerated, delivering solely the mono-phosphorylation product (3g). Moreover, oxime derivatives selectively delivered the desired product (3i). Third, various types of arenes were explored, featuring a wide range of oxazolinyl, pyrazyl or pyridyl substituents (3j–3n). Afterwards, the scope of phosphorus coupling partners was further investigated. Fourth, diphenylphosphine oxides bearing electron-donating and electron-withdrawing groups were shown to be suitable coupling partners leading to the formation of the expected products 3o–3s in good yields. Other pentavalent phosphine oxides, comprised of phenylalkyl phosphine oxides, dithienyl phosphine oxides, and commonly used dimethyl phosphine oxides, in phosphorylated drugs, have been demonstrated to be successful coupling partners (3t–3w).

Phosphorylation motifs can confide biological activity to drug molecules.^5^ In this context, the direct installation of phosphorylation units into target molecules, accounts for a more sustainable access to structural diversity, facilitating the expansion of the chemical space. Hence, we wondered whether our strategy could be exploited for late-stage C–H phosphorylations. To our delight, diazepam - a therapeutic drug used for acute tension and anxiety states - proved to be an amenable substrate, delivering the desired products 3x–3y in high yields. Additionally, oxaprozin and 6-phenylpurine riboside, delivered the desired products (3z–3aa) with excellent levels of position-selectivity.

### Mechanistic studies of *ortho*-C–H phosphorylation

**Scheme 3:**
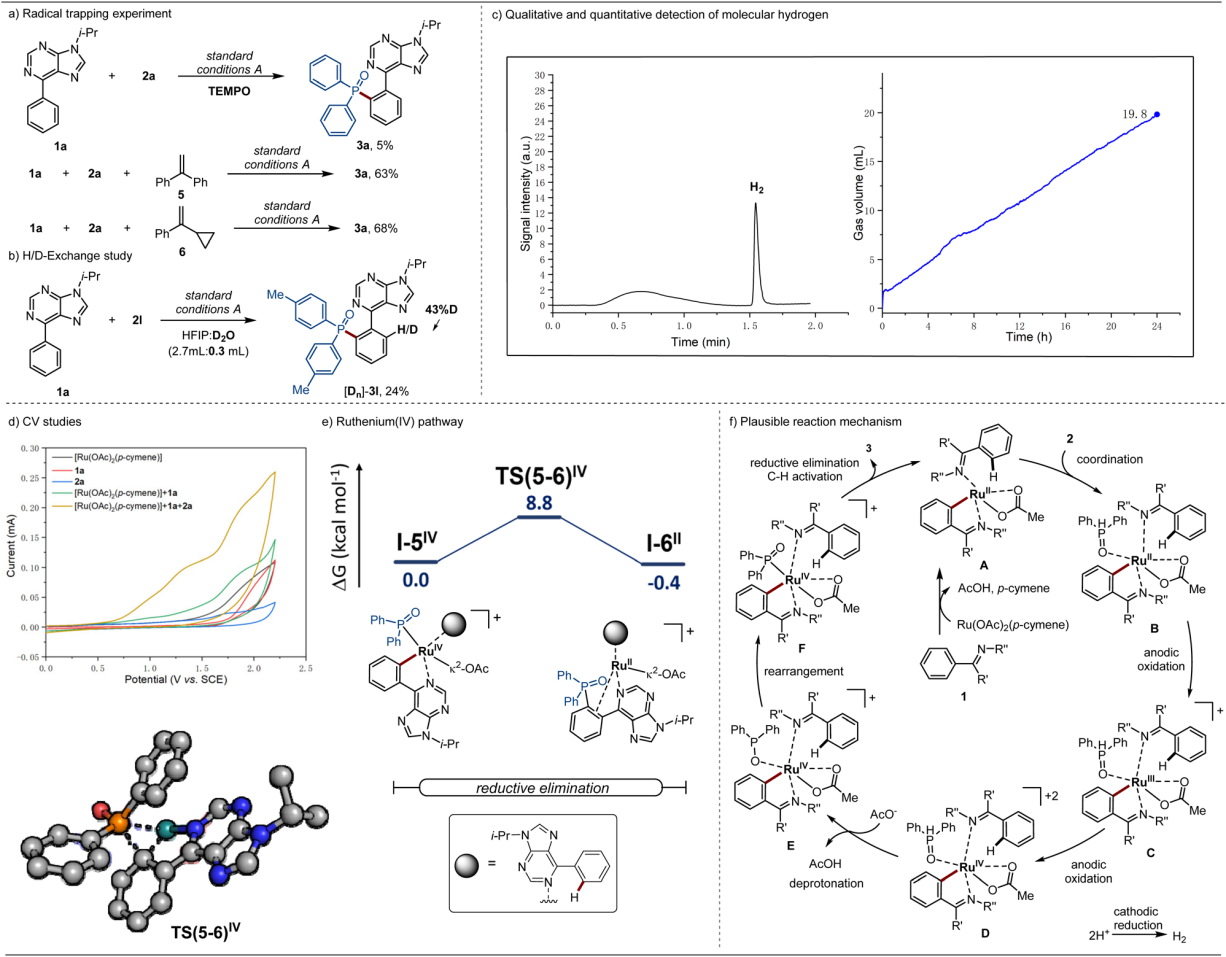
(a) Radical trapping experiment. (b) H/D-Exchange study. (c) Headspace GC analysis after catalysis (left side) and measurement of gas evolution during catalysis (right side). (d) Cyclic voltammetry studies. (e) Relative Gibbs free energies (Δ*G*_338.15_) are given in kcal mol^−1^ for the ruthenium-catalyzed *ortho*-C–H phosphorylation reductive elimination step at the PBE0-D4/def2-TZVPP-SMD(HFIP)//PBE0-D3BJ/def2-SVP level of theory. In the computed transition state structure, non-relevant hydrogens were omitted for clarity. (f) Plausible reaction mechanism for the ruthenaelectro-catalyzed *ortho*-C–H phosphorylation.^27^

To gain insights into the reaction mechanism, a series of control experiments were conducted. The addition of equimolar amounts of TEMPO resulted in the inhibition of the *ortho*-C–H phosphorylation. However, given that TEMPO is a good reducing agent,^27^ such observations do not conclusively support the formation of a *P*-centered radical. Therefore, additional mechanistic experiments were conducted in the presence of representative radical scavengers, including 1,2-diphenylethylene and vinylcyclopropane 6, which is known to undergo ring opening in the presence of radial species. As a result, the *ortho*-phosphorylation product 3a was obtained in comparable isolated yields of 63% and 68%, respectively. These findings provide support for a non-radical pathway (Scheme 3a). Next, the addition of D_2_O as co-solvent resulted in a H/D scrambling at the *ortho*-position, suggesting a reversible *ortho*-C–H activation (Scheme 3b).

In order to gain insights into the cathodic process, headspace gas chromatography was used and the formation of molecular hydrogen was probed. Thus, we monitored and quantified the formation of molecular hydrogen during the electrocatalytic reaction, which was determined to be 19.8 mL by the end of the electrocatalysis, translating into a faradaic efficiency of 59% (Scheme 3c and ESI Fig. S2, S3†). The result confirmed the hydrogen evolution reaction (HER) as the primary cathodic process, highlighting the unique potential of ruthenium electrocatalysis as a sustainable technology for organic synthesis.

Additionally, cyclic voltammetric (CV) experiments were conducted to assess the redox potential of the substrates as well as the catalyst (Scheme 3d). For the *ortho*-C–H phosphorylation, the mixture of phenyl-9*H*-purine (1a), diphenylphosphine oxide (2a), and the catalyst [Ru(OAc)_2_(*p*-cymene)] exhibited oxidation peaks at *E*_p/2_ = 0.95 V and *E*_p/2_ = 1.26 V *vs.* SCE. These findings are indicative of a ruthenacycle generated after C–H activation being coordinated by SPO 2a.

The catalyst mode of action for the ruthenium-electrocatalyzed *ortho*-C–H phosphorylation was further investigated through DFT calculations at the PBE0-D4/def2-TZVPP-SMD(HFIP)//PBE0-D3BJ/def2-SVP level of theory (Scheme 3e, Fig. S8 and S9, in the ESI†).^28^ Upon phosphine coordination two single electron oxidation steps take place, leading to the formation of the ruthenium(iv) intermediate I–1^IV^ (Fig. S8†). Such is consistent with the CV studies, where two oxidation peaks were observed in the presence of phosphine. Subsequently, a facile phosphine deprotonation takes place (TS(2-3)) with an energy barrier of 6.8 kcal mol^−1^ giving rise to intermediate I-3, which after rearrangement originates a more exergonic intermediate I-5. The latter undergoes reductive elimination through transition state TS(5-6) with an energy barrier of 8.8 kcal mol^−1^. Additionally, an alternative pathway for reductive elimination under ruthenium(iii) was also investigated (Fig. S9†). The latter has proven to be energetically disfavored, not only by the prohibitive calculated barrier of 40.3 kcal mol^−1^ but also by the formation of an endergonic ruthenium(i) intermediate I-6^I^. Such observations provide support for a ruthenium(III/IV/II) regime.

Based on our experimental and computational mechanistic studies, a plausible reaction mechanism is depicted in Scheme 3f. The *ortho*-phosphorylation commences with the *ortho*-C–H activation, forming the ruthenacycle complex A. Subsequently, ligand exchange occurs, leading to a more easily oxidized intermediate B, which after two single electron oxidation steps originates the ruthenium(iv) intermediate D. Then, deprotonation gives rise to intermediate E, which rearranges to form intermediate F. Finally, F undergoes reductive elimination to yield the desired product 3 and to regenerate the active ruthenium(ii) catalyst A.

### Reaction optimization of *para*-C–H phosphorylation

Next, we focused our attention on the envisioned switch towards ruthenaelectro-catalyzed *para*-C–H phosphorylation. We began our studies with arene 1a and diphenylphosphine oxide 2a using graphite felt (GF) and platinum (Pt) electrodes as anode and cathode materials, respectively, in an undivided cell setup (Table 2). When RuCl_3_·3H_2_O was used as a catalyst in the presence of MeCN as solvent a minor amount of *para*-C–H phosphorylated product was obtained. Upon further experimentation, the *para*-phosphorylated product 4a was selectively obtained in an isolated yield of 82% in the presence of RuCl_3_·3H_2_O, triethylamine, and a solvent mixture of MeCN and water (Entry 1). Notably, only unconverted substrate 1a was accounted for in the mass balance here, with no side product, from possible phosphorylation at the purine C–H bond, being detected. Other tested solvents and bases did not improve the efficacy of the reaction (entries 3–4). Moreover, the electricity was shown to be essential for the catalysis to take place (entry 6).

**Table 2:** Optimization of the *para*-C–H phosphorylation reaction conditions^a^

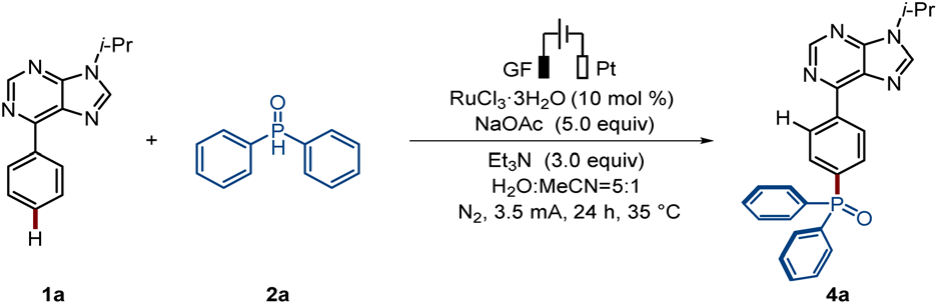		
Entry	Deviation from standard conditions	Yield of 4a^b^ (%)
1	None	82
2	Under air	66
3	H_2_O/CH_3_CN/TFE as solvent	25/12/—
4	Na_2_CO_3_/Na_3_PO_4_/NaOPiv as base	78/55/23
5^c^	PPh_3_/2,2′-bipyridine as ligand	55/42
6	Without electricity	0

a Reaction conditions B: undivided cell, GF anode, Pt cathode, 1a (0.3 mmol), 2a (4.0 equiv.), [RuCl_3_·3H_2_O] (10 mol%), NaOAc (5.0 equiv.), Et_3_N (3.0 equiv.), MeCN : H_2_O = (0.5 : 2.5 mL), N_2_, 35 °C, 3.5 mA, 24 h.

b Isolated yields.

c PPh_3_ (10 mol%), 2,2′-bipyridine (10 mol%).

### Substrate scope investigation for the *para*-C–H phosphorylation

**Scheme 4:**
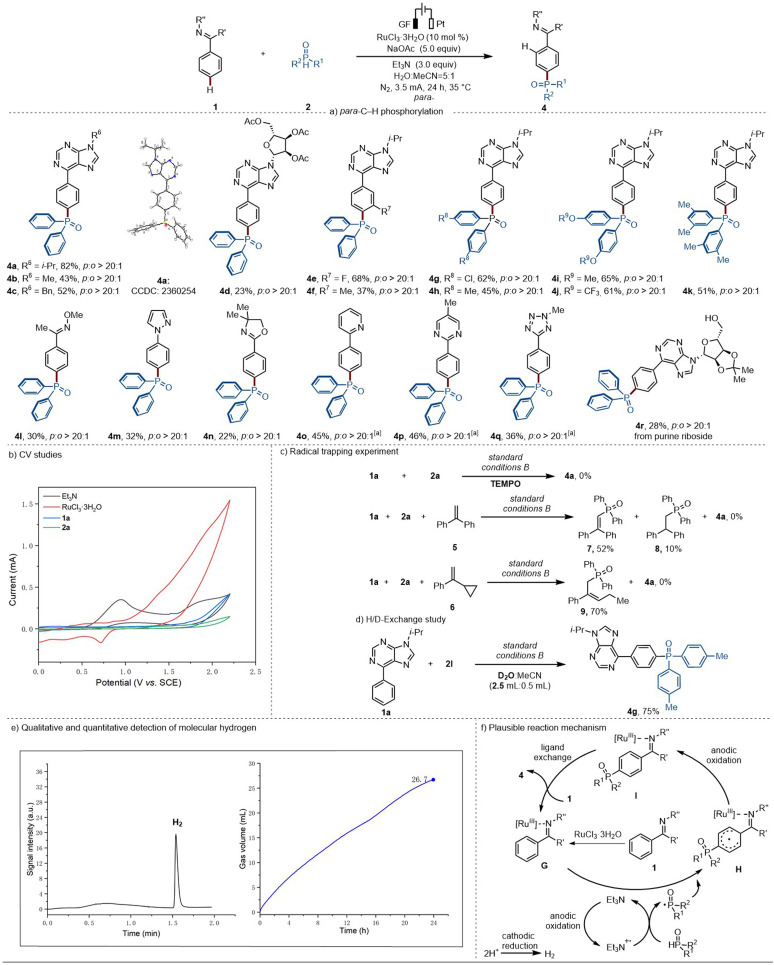
(a) Scope of the ruthenium-electrocatalyzed *para*-selective C–H phosphorylation, ^a^no Et_3_N. (b) Cyclic Voltammograms. (c) Radical trapping experiment. Yield ratio calculated by phosphorus NMR from a mixture of 7 and 8. (d) H/D-exchange study. (e) Headspace GC analysis after catalysis (left side) and measurement of gas evolution during catalysis (right side). (f) Plausible reaction mechanism involved in the ruthenaelectro-catalyzed of *para*-C–H phosphorylation.

With the optimized reaction conditions in hand, we directed our attention to the viable substrate scope of the ruthenium-electrocatalyzed *para*-C–H phosphorylation (Scheme 4a). Several *N*-substituted 6-phenylpurines were explored, enabling the formation of the corresponding desired products in good yields with exclusive *para*-selectivity (4a–4d). Furthermore, both electron-withdrawing groups (–F) and electron-donating (–Me) on the arenes were well tolerated (4e–4f). Diphenylphosphine oxide compounds bearing different substituents (–Cl, –Me, –OMe, –OCF_3_) were also well tolerated (4g–4k). Noticeably, the 1-phenylpyrazole yielded the expected single *para*-C–H phosphorylation product, with no phosphorylation occurring at the pyrazole ring (4m). Arenes bearing different substituents, such as pyrimidyl, pyridyl, or tetrazolyl groups, demonstrated higher efficacy, in the absence of triethylamine, with excellent *para*-selectivity (4o–4q). Moreover, our approach could be successfully applied to the late-stage C–H phosphorylation of 6-phenylpurine riboside, delivering the desired product (4r) with excellent position-selectivity.

### Mechanistic studies for the *para*-C–H phosphorylation

To gain mechanistic insights into the ruthenium-electrocatalyzed *para*-C–H phosphorylation, cyclic voltammetry (CV) experiments were conducted to access the redox potential of the substrates as well as the catalyst (Scheme 4b). The Et_3_N features an oxidation peak at *E*_p/2_ = 0.91 V *vs.* SCE, which is much lower than the one obtained for 1a, 2a, and RuCl_3_·3H_2_O, indicating that Et_3_N is more susceptible to be oxidized.

Additionally, a series of control experiments were performed. The addition of TEMPO under otherwise identical reaction conditions inhibited the *para*-C–H phosphorylation. Moreover, upon the addition of 1,2-diphenylethylene, the corresponding radical intermediates 7 and 8 were trapped. When vinylcyclopropane 6 was added, the compound resulting from the ring opening (9) could be isolated in 70% yield. These findings provide strong support for the involvement of a phosphorus-centered radical in the *para*-phosphorylation (Scheme 4c). However, when D_2_O was added to the *para*-phosphorylation reaction, no H/D-scrambling was observed, suggesting that an *ortho*-C–H cycloruthenation is not relevant for the *para*-reaction pathway (Scheme 4d).

Additionally, we monitored and quantified the formation of molecular hydrogen during the electrocatalytic reaction, which was determined to be 26.7 mL by the end of the reaction time, translating into a faradaic efficiency of 68% (Scheme 4e and ESI Fig. S4, S5†). This provides support for the hydrogen evolution reaction (HER) to be the primary cathodic process.

Based on our experimental mechanistic studies, a plausible reaction mechanism is depicted in Scheme 4f. For the *para*-phosphorylation pathway, the nitrogen-containing heteroarene first coordinates to the ruthenium(iii) catalyst to form intermediate G. Then, Et_3_N^·+^ may react with H-phosphonate or H-phosphine oxide to give rise to a phosphorus-centered radical. Subsequently, the electrophilic phosphine radical will attack at the *para*-position of the arene *via* a charge transfer-directed approach,^29^ followed by oxidative aromatization to generate the *para*-phosphorylated product. In the meanwhile, at the cathode, protons are reduced to generate molecular hydrogen by HER.

## Conclusions

In conclusion, we have devised a position-selectivity switch for electrochemical ruthenium-catalyzed C–H phosphorylations enabled by hydrogen evolution reaction (HER). Thereby, we achieved selective *ortho*- and even *para*-C–H phosphorylation. The robustness of the ruthena-electrocatalysis was reflected by a wide substrate scope including various sensitive electrophilic functional groups. Our strategy thereby enabled challenging late-stage phosphorylations of biorelevant pharmaceuticals. Experimental and computational mechanistic studies provided strong support for an unusual ruthenium(iii/iv/ii) manifold for the ruthenaelectro-catalyzed proximal phosphorylation.

## Data availability

All data associated with this study are available in the article and ESI.†

## Author contributions

Conceptualization, L. A.; methodology, X.-Y. G.; investigation, X.-Y. G.; DFT calculation, J. C. A. O.; cyclic voltammetry studies, S. L. H.; headspace GC analysis and measurement, S .T.; HRMS studies, T. v. M.; writing – original Draft, X.-Y. G., B.-S. Z. and J. C. A. O.; writing – review & editing, X.-Y. G., J. C. A. O., and S. C.; funding acquisition, L. A.; resources, L. A.; supervision, L. A.

## Conflicts of interest

There are no conflicts to declare.

## Supplementary Material

SC-OLF-D4SC06219A-s001
