# Supplementary material for: Ruthenaelectro-catalyzed C–H phosphorylation: ortho to para position-selectivity switch
Source: Chem Sci. 2024 Nov 29;16(2):824–33. doi: 10.1039/d4sc06219a (PMC11619359; doi:10.1039/d4sc06219a)
Supplement: SC-016-D4SC06219A-s001 [file SC-016-D4SC06219A-s001.pdf]

Supplementary Information (SI) for Chemical Science.
This journal is © The Royal Society of Chemistry 2024

Supporting Information

Ruthenaelectro-Catalyzed C–H Phosphorylation: ortho to para Position-Selectivity Switch

Xue-Ya Gou,[a‡] João C. A. Oliveira,[a‡] Shan Chen,[a] Simon L. Homölle,[a] Sven Trienes,[a] Tristan von Münchow,[a] Bo-Sheng Zhang,[a] and Lutz Ackermann[a]*

Institut für Organische und Biomolekulare Chemie and Wöhler-Research Institute for Sustainable Chemistry (WISCh), Georg-August-Universität, Tammannstrasse 2, 37077 Göttingen, Germany

E-mail: Lutz.Ackermann@chemie.uni-goettingen.de

‡These authors contributed equally.

Table of Contents

1. General Experimental Details 2
2. Optimization of the Reaction Conditions 3
3. General Procedure 4
4. Mechanistic Studies 6
5. X-ray Crystallographic Data 15
6. Computational Studies 19
7. References 49
8. Characterization Data 51
9. NMR Spectroscopic Data 77

1. General Experimental Details

Platinum electrodes (10 mm × 25 mm × 0.25 mm, 99.9%, obtained from ChemPur® Karlsruhe, Germany) and graphite felt (GF) electrodes (10 mm × 25 mm × 6 mm, SIGRACELL® GFA 6 EA, obtained from SGL Carbon, Wiesbaden, Germany) were connected using stainless steel adapters. Electrocatalysis was performed using a ROHDE&SCHWARZ HMP4040 potentiostat.

Solvents for column chromatography and extraction (EtOAc, *n*-hexane, MeOH, DCM) were distilled prior to their use. Routine TLC analysis was carried out on aluminium sheets coated with silica gel 60 F254, 0.2 mm thickness. Plates were analyzed using a 254 nm UV lamp. Chromatography was carried out on Merck silica gel 60 (40–63 µm). All starting materials, reagents and solvents were purchased from commercial suppliers (Aldrich, Alfa, TCI, Daicel, etc.) and used as supplied unless otherwise stated.

NMR spectra were recorded on Bruker-300 MHz spectrometer or Bruker-400 MHz spectrometer. Chemical shifts (δ) are given in ppm relative to TMS. The residual solvent signals were used as references and the chemical shifts converted to the TMS scale ($CDCl_3$: δH = 7.26 ppm, δC = 77.00 ppm). Data for ^{1}H NMR are reported as follows: chemical shift (δ), multiplicity (s = singlet, d = doublet, t = triplet, q = quartet, p = quintet, m = multiplet, dd =doublet of doublets, ddd = doublet of double doublets, dt = doublet of triplets), coupling constants *J* are reported in Hertz (Hz).

MS: EI-MS were recorded with Finnigan MAT 95, 70 eV and Finnigan LCQ; High resolution mass spectrometry (HR-MS) with APEX IV 7T FTICR; ESI-MS were recorded with Bruker *Daltonics maXis* (ESI-QTOF-MS), Bruker Daltonics *micrOTOF* (ESI-TOF-MS) or Thermo Scientific *LTQ Orbitrap XL* (ESI-Orbitrap-MS). All IR spectra were recorded on a Bruker FT-IR Alpha-P device or on a Thermo Scientific Nicolet iS5 device equipped with an iD7 ATR detector and were recorded in the range from 4000 to 400 cm^{-1}. CV studies were performed using a Metrohm Autolab PGSTAT204 workstation and Nova 2.1 software.

Headspace analysis was performed on an Agilent 7890B GC System using a Thermal Conductivity Detector and a 5Å MS column. The gas evolution study was conducted using the GasMess-System (LIKAT Rostock and MesSen Nord GmbH).

2. Optimization of the Reaction Conditions

Table S1. Reaction optimization for *ortho*-C–H phosphorylation.[a]

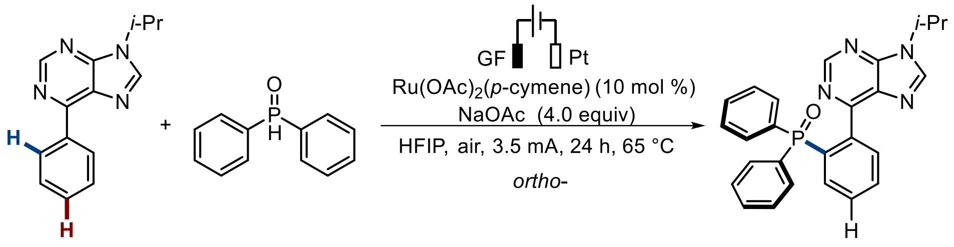

1a **2a** **3a**

Entry	Deviation from standard conditions	Yield [b]
1	None	73
2	TEF / CH_3CN / MeOH / DMA as solvent	65/-/-/-
3	35°C /45° C	19/35
4	GF(+) / GF(-), Pt(+) / GF(-), RVC(+) / Pt(-) as electrode	32/23/63
5	*n*-Bu_4NOAc, $NaPF_6$, *n*-Bu_4NBF_4, $LiClO_4$ as electrolyte	70/55/34/65
6	1 mA 72 h, 2 mA 36 h, 4 mA 18 h	49/65/61
7	No [$Ru(OAc)_2$(*p*-cymene)]	0
8	No electricity	0

[a]**Reaction conditions A**: Undivided cell, GF anode, Pt cathode, **1a** (0.3 mmol, 1.0 equiv), **2a** (4.0 equiv), [$Ru(OAc)_2$(*p*-cymene)] (10 mol %), NaOAc (4.0 equiv), HFIP (3.0 mL), electrolyte (0.1 M), air, 65 °C, 3.5 mA, 24 h. [b]Isolated yields.

Table S2. Reaction optimization for *para*-C–H phosphorylation.[a]

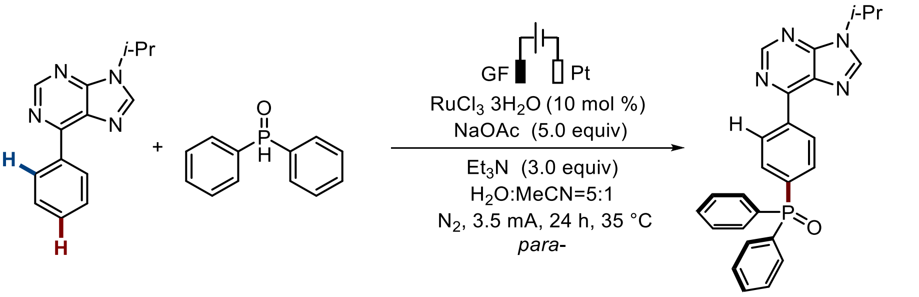

1a **2a** **4a**

Entry	Deviation from standard conditions	Yield [b]
1	None	82
2	H_2O / CH_3CN / PhMe / MeOH as solvent	25/12/-/-
3	55°C / 25°C	72/67
4	GF(+) / GF(-), Pt(+) / GF(-), RVC(+) / Pt(-) as electrode	5/8/77
5	*n*-Bu_4NOAc, $NaPF_6$, *n*-Bu_4NBF_4, Et_4NCl, *n*-Bu_4NClO_4 as electrolyte	80/61/39/79/21
6	2 mA 36 h, 4 mA 18 h, 6 mA 12 h	65/76/59
7	No [$RuCl_3 \cdot 3H_2O$]	58
8	No electricity	0

[a]**Reaction conditions B**: Undivided cell, GF anode, Pt cathode, **1a** (0.3 mmol, 1.0 equiv), **2a** (4.0 equiv), [$RuCl_3 \cdot 3H_2O$] (10 mol %), NaOAc (5.0 equiv), Et_3N (3.0 equiv), MeCN:H_2O = (0.5:2.5 mL), electrolyte (0.1 M), N_2, 35 °C, 3.5 mA, 24 h. [b]Isolated yields.

3. General Procedure

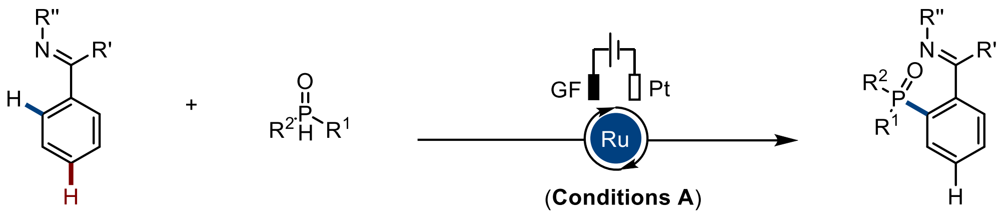

1 2 3

Conditions A:

Under air atmosphere, an undivided cell was charged with **1** (0.3 mmol, 1.0 equiv), **2** (1.2 mmol, 4.0 equiv), [Ru(OAc)$_2$(*p*-cymene)] (10 mol %), NaOAc (4.0 equiv), HFIP (3.0 mL), and equipped with a graphite felt (GF) anode (10 mm × 25 mm × 6 mm) and a platinum (Pt) cathode (10 mm × 25 mm × 0.25 mm). The resulting light-red suspension was stirred vigorously at room temperature for 10 minutes, and then placed in a pre-heated oil bath at 65 °C for electrolysis under a constant current of 3.5 mA for 24 hours. After the reaction was completed, the electrodes were carefully washed with DCM (3 × 10 mL) and MeOH (3 × 10 mL). The combined mixture was initially purified by flash column chromatography on silica gel (DCM/MeOH = 30/1-10/1) to afford the crude product, which was further purified by chromatography on silica gel to afford the corresponding product **3**.

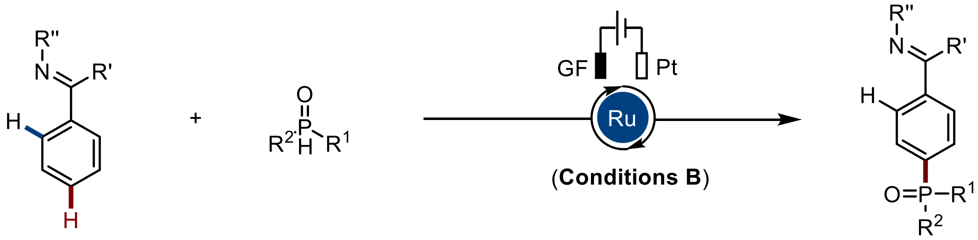

1 2 4

Conditions B:

Under N_2 atmosphere, an undivided cell was charged with **1** (0.3 mmol, 1.0 equiv), **2** (1.2 mmol, 4.0 equiv), [$RuCl_3 \cdot 3H_2O$] (10 mol %), NaOAc (5.0 equiv), Et_3N (3.0 equiv), MeCN: H_2O = (0.5 mL: 2.5 mL), and equipped with a graphite felt (GF) anode (10 mm × 25 mm × 6 mm) and a platinum (Pt) cathode (10 mm × 25 mm × 0.25 mm). The resulting black suspension was stirred vigorously at room temperature for 10 minutes, and then placed in a pre-heated oil bath at 35 °C for electrolysis under a constant current of 3.5 mA for 24 hours. After the reaction was completed, the electrodes were carefully washed with DCM (3 × 10 mL) and MeOH (3 × 10 mL). The combined mixture was initially purified by flash column chromatography on silica gel (DCM/MeOH = 30/1-10/1) to afford the crude product, which was further purified by chromatography on silica gel to afford the corresponding product **4**.

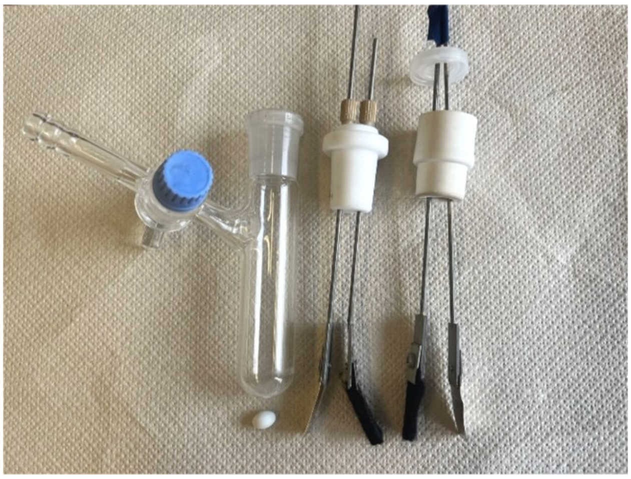

Figure S1. Picture of the electrochemical cell, stirring bar, and teflon cap with a graphite felt (GF) anode and a platinum (Pt) cathode

4. Mechanistic Studies

1) Radical trapping experiments for the *ortho*-C–H phosphorylation

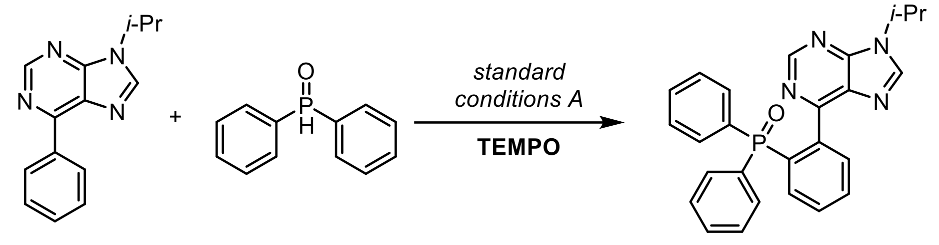

1a **2a** **3a**, 5%

Following standard procedure A using **1a** (0.3 mmol, 1.0 equiv), **2a** (1.2 mmol, 4.0 equiv), [Ru(OAc)$_2$(*p*-cymene)] (10 mol %), NaOAc (4.0 equiv), **TEMPO** (1.2 mmol, 4.0 equiv), HFIP (3.0 mL). After the reaction was completed, the residue was purified with column chromatography on silica gel to give the compound **3a** (6.5 mg, 5%).

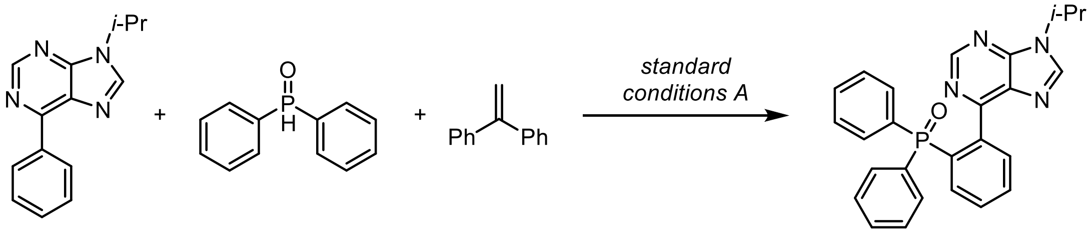

1a **2a** **5** **3a**, 63%

Following standard procedure A using **1a** (0.3 mmol, 1.0 equiv), **2a** (1.2 mmol, 4.0 equiv), [Ru(OAc)$_2$(*p*-cymene)] (10 mol %), NaOAc (4.0 equiv), **5** (1.2 mmol, 4.0 equiv), HFIP (3.0 mL). After the reaction was completed, the residue was purified with column chromatography on silica gel to give the compound **3a** (82.8 mg, 63%).

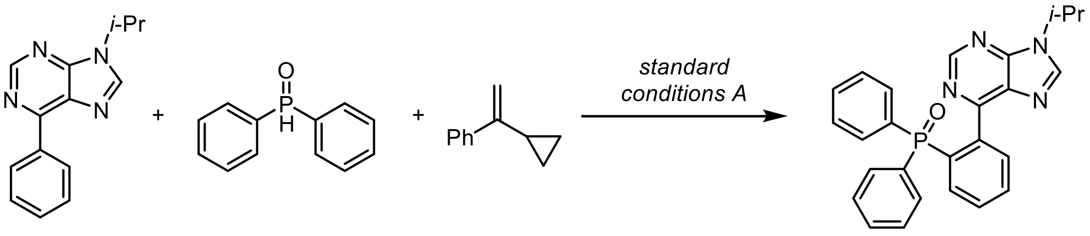

1a **2a** **6** **3a**, 68%

Following standard procedure A using **1a** (0.3 mmol, 1.0 equiv), **2a** (1.2 mmol, 4.0 equiv), [Ru(OAc)$_2$(*p*-cymene)] (10 mol %), NaOAc (4.0 equiv), **6** (1.2 mmol, 4.0 equiv), HFIP (3.0 mL). After the reaction was completed, the residue was purified with column chromatography on silica gel to give the compound **3a** (89.5 mg, 68%).

2) Radical trapping experiments for the *para*-C–H phosphorylation

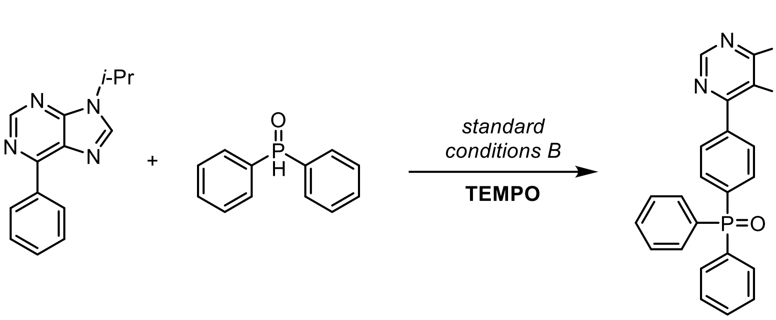

1a **2a** **4a**, 0%

Following standard procedure B using **1a** (0.3 mmol, 1.0 equiv), **2a** (1.2 mmol, 4.0 equiv), [$RuCl_3 \cdot 3H_2O$] (10 mol %), NaOAc (5.0 equiv), Et_3N (3.0 equiv), **TEMPO** (1.2 mmol, 4.0 equiv), MeCN: H_2O = (0.5 mL: 2.5 mL). After the reaction was completed, the TLC and GC-MS were employed to observe the result, no desired product had been observed.

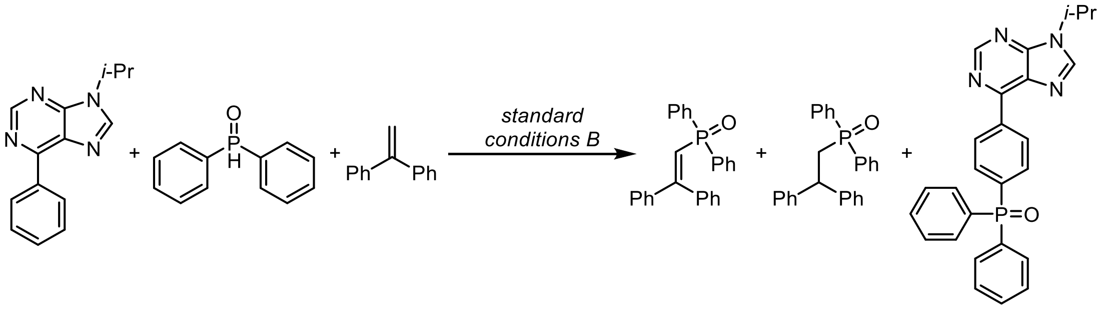

1a **2a** **5** **7**,52% **8**,10% **4a**, 0%

Following standard procedure B using **1a** (0.3 mmol, 1.0 equiv), **2a** (1.2 mmol, 4.0 equiv), [$RuCl_3 \cdot 3H_2O$] (10 mol %), NaOAc (5.0 equiv), Et_3N (3.0 equiv), **5** (1.2 mmol, 4.0 equiv), MeCN: H_2O = (0.5 mL: 2.5 mL). After the reaction was completed, the residue was purified with chromatography column on silica gel to give the mixed compounds **7** and **8** (71.5 mg, **7**:**8** = 5:1).[1]

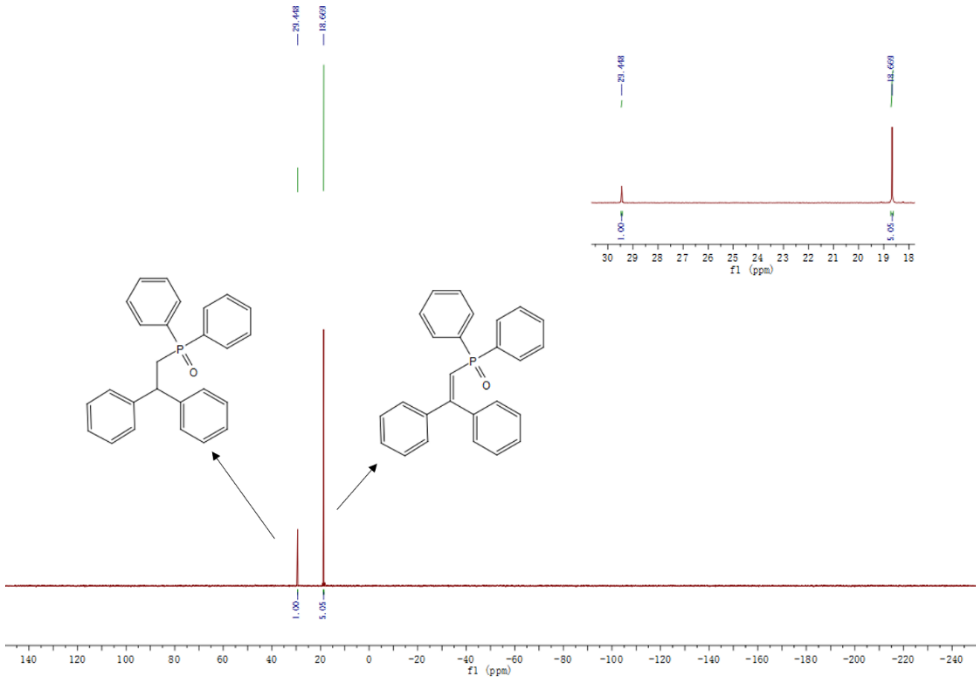

^{31}P NMR (121 MHz, $CDCl_3$) δ 29.45, 18.67.
HRMS (ESI) m/z: $[M+H]^+$ Calcd for $C_{26}H_{22}OP$ 381.1403; Found 381.1408; $[M+H]^+$ Calcd for $C_{26}H_{24}OP$ 383.1559; Found 383.1551.

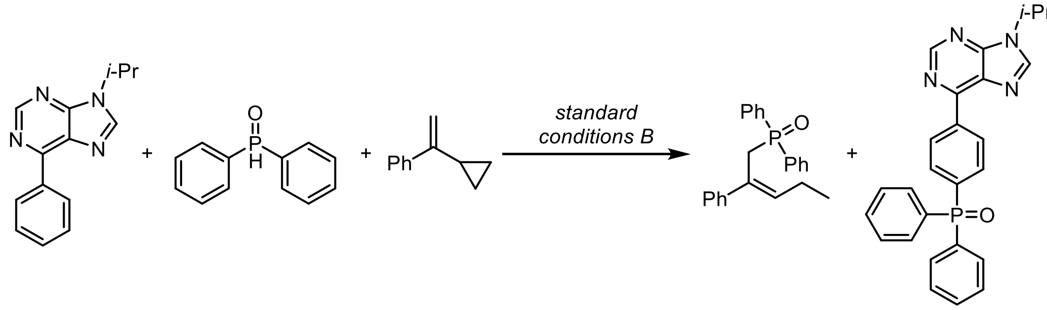

1a **2a** **6** **9**, 70% **4a**, 0%

Following standard procedure B using **1a** (0.3 mmol, 1.0 equiv), **2a** (1.2 mmol, 4.0 equiv), [$RuCl_3·3H_2O$] (10 mol %), NaOAc (5.0 equiv), Et_3N (3.0 equiv), **6** (1.2 mmol, 4.0 equiv), MeCN: H_2O = (0.5 mL: 2.5 mL). After the reaction was completed, the residue was purified with chromatography column on silica gel to give the compound **9** (72.7 mg, 70%).
(Z)-Diphenyl(2-phenylpent-2-en-1-yl)phosphine oxide (**9**)

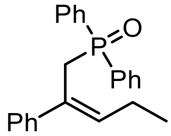

^1H NMR (300 MHz, $CDCl_3$) δ 7.60 – 7.53 (m, 4H), 7.36 – 7.30 (m, 2H), 7.27 – 7.21 (m, 4H), 7.06 – 7.00 (m, 5H), 5.71 – 5.60 (m, 1H), 3.54 (d, J = 14.8 Hz, 2H), 2.11 – 2.02 (m, 2H), 0.87 (t, J = 7.5 Hz, 3H).
^{31}P NMR (121 MHz, $CDCl_3$) δ 28.63.
HRMS (ESI) m/z: $[M+H]^+$ Calcd for $C_{23}H_{24}OP$ 347.1559; Found 347.1563.
IR (ATR): 3055, 2961, 1591, 1436, 1182, 909, 712, 506 cm^{-1}.

3) H/D-Exchange study for the *ortho*-C–H phosphorylation

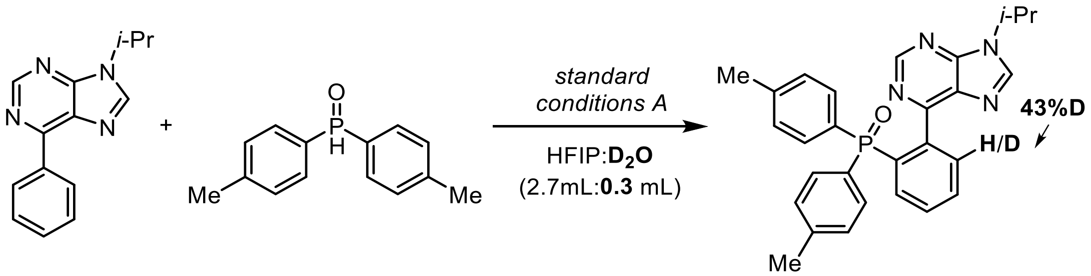

1a **2l** **[Dn]-3l**, 24%

Following standard procedure A using **1a** (0.3 mmol, 1.0 equiv), **2l** (1.2 mmol, 4.0 equiv), [$Ru(OAc)_2$(*p*-cymene)] (10 mol %), NaOAc (4.0 equiv), HFIP:$\mathbf{D_2O}$ = (2.7 mL:**0.3** mL). After the reaction was completed, the residue was purified with column chromatography on silica gel to give the product **[D]ₙ–3l** in a 24% isolated yield. The H incorporation was determined by ^1H-NMR spectroscopy.

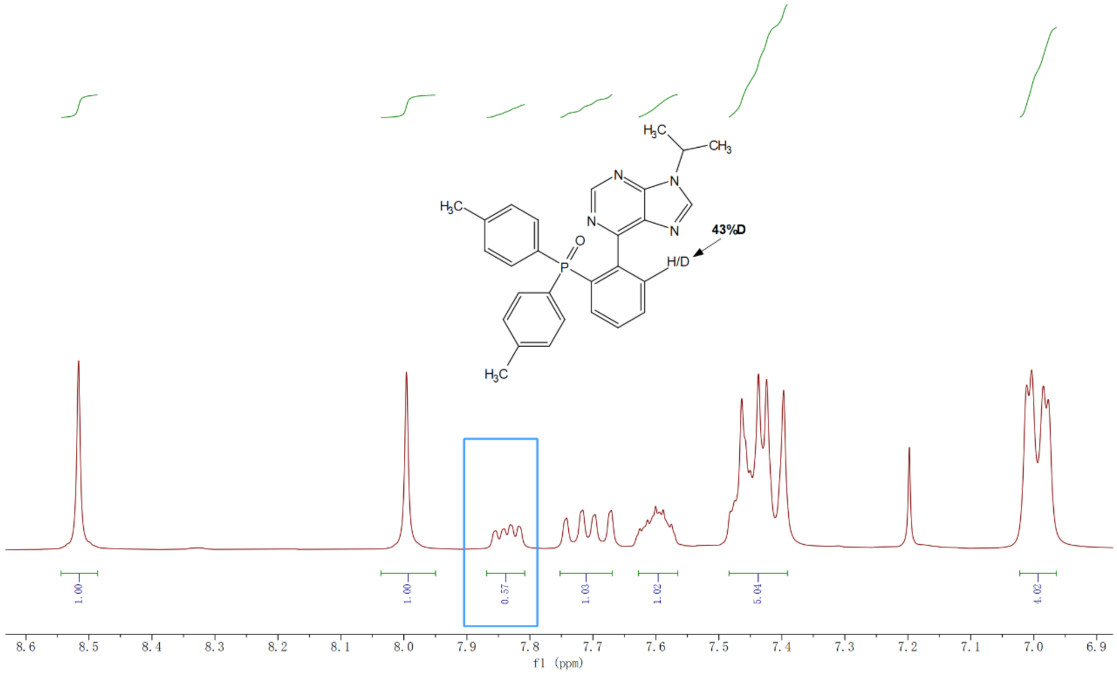

4) H/D-Exchange study for the *para*-C–H phosphorylation

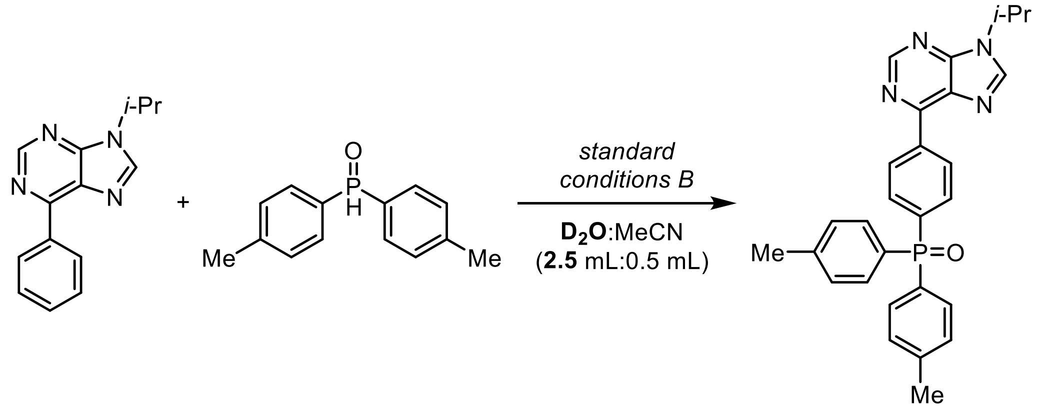

Following standard procedure B using **1a** (0.3 mmol, 1.0 equiv), **2l** (1.2 mmol, 4.0 equiv), [$RuCl_3 \cdot 3H_2O$] (10 mol %), NaOAc (5.0 equiv), Et_3N (3.0 equiv), **D_2O**:MeCN = (**2.5** mL:0.5 mL). After the reaction was completed, the residue was purified with column chromatography on silica gel to give the product **4g** in a 75% isolated yield. No H/D exchange was detected by ^{1}H-NMR spectroscopy.

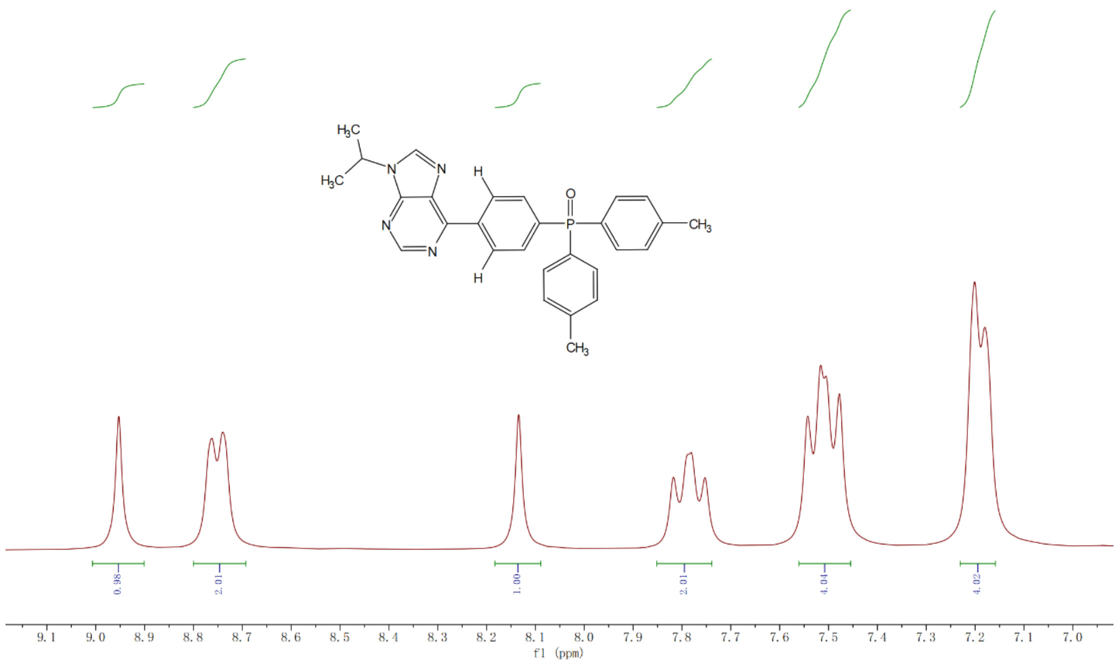

5) Hydrogen detection for the *ortho*-C–H phosphorylation

Following standard procedure A using **1a** (0.3 mmol, 1.0 equiv), **2a** (1.2 mmol, 4.0 equiv), [Ru(OAc)$_2$(*p*-cymene)] (10 mol %), NaOAc (4.0 equiv), HFIP (3.0 mL). After the reaction was completed, 1.0 mL of the headspace volume was removed for GC analysis.

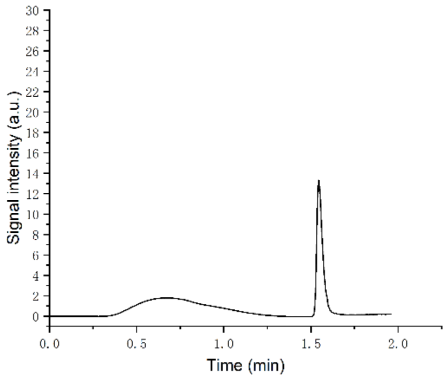

Figure S2. GC-TCD chromatogram obtained for the headspace analysis of the *ortho*-C–H phosphorylation.

6) Gas evolution measurement for the *ortho*-C–H phosphorylation:

Following standard procedure A using **1a** (0.3 mmol, 1.0 equiv), **2a** (1.2 mmol, 4.0 equiv), [Ru(OAc)$_2$(*p*-cymene)] (10 mol %), NaOAc (4.0 equiv), HFIP (3.0 mL) at **55 °C** under air atmosphere according to the general procedure.

The change in volume of the headspace was recorded under isobaric conditions.

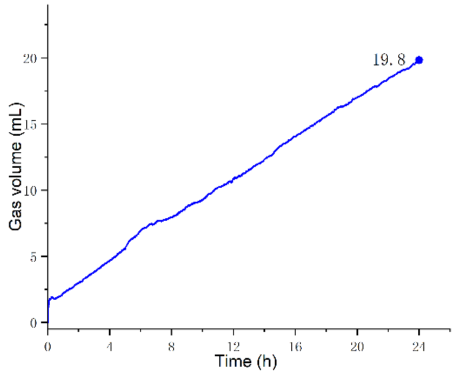

Figure S3. Measurement of the hydrogen volume generated during catalysis.

Calculation of cathode hydrogen generation efficiency:

Current: I = 3.5 mA, Time: t = 24 h = 86400 s, Faraday constant: F = 96485.3321 C/mol

Therefore, the number of electrons passing through the electron cathode is:

$$n_e = \frac{I \times t}{F} = 0.0035\,A \times 86400\,s \div 96485.3321\,C/mol = 0.003134\,mol = 3.134\,mmol$$

Theoretical hydrogen production:

$$n_{H_2} = \frac{n_e}{2} = 1.567\,mmol$$

Measured amount of hydrogen:

$$V_{mH_2} = 19.8\,mL = 0.0000198\,m^3$$

$$n_{mH_2} = \frac{P \times V_{mH_2}}{RT} = \frac{99270\,Pa \times 0.0000198\,m^3}{8.3145\,J/(K \cdot mol) \times 298.15\,K} = 0.000793\,mol = 0.793\,mmol$$

Hydrogen generation efficiency:

$$efficiency = \frac{0.793\,mmol}{1.567\,mmol} = 50\%$$

7) Hydrogen detection for the *para*-C–H phosphorylation

Following standard procedure B using **1a** (0.3 mmol, 1.0 equiv), **2a** (1.2 mmol, 4.0 equiv), [$RuCl_3 \cdot 3H_2O$] (10 mol %), NaOAc (5.0 equiv), Et_3N (3.0 equiv), MeCN: H_2O = (0.5 mL: 2.5 mL). After the reaction was completed, 1.0 mL of the headspace volume was removed for GC analysis.

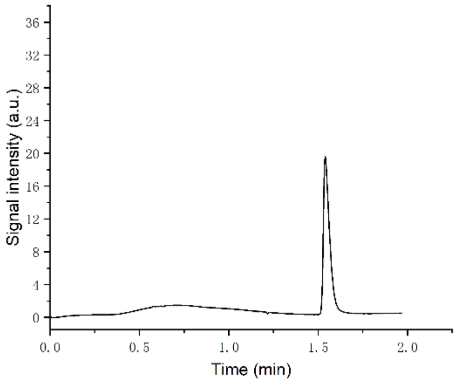

Figure S4. GC-TCD chromatogram obtained for the headspace analysis of the *para*-C–H phosphorylation.

8) Gas evolution measurement for the *para*-C–H phosphorylation:

Following standard procedure B using **1a** (0.3 mmol, 1.0 equiv), **2a** (1.2 mmol, 4.0 equiv), [$RuCl_3 \cdot 3H_2O$] (10 mol %), NaOAc (5.0 equiv), Et_3N (3.0 equiv), MeCN: H_2O = (0.5 mL: 2.5 mL) at 35 °C under N_2 atmosphere according to the general procedure.

The change in volume of the headspace was recorded under isobaric conditions.

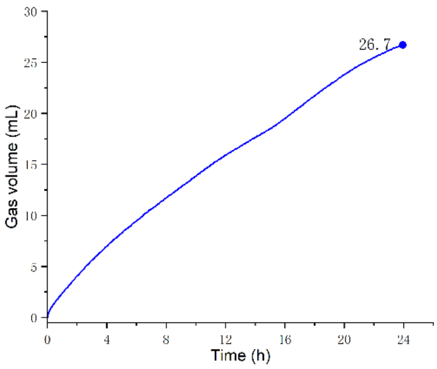

Figure S5. Measurement of the hydrogen volume generated during catalysis.

Calculation of cathode hydrogen generation efficiency:

Current: I = 3.5 mA, Time: t = 24 h = 86400 s, Faraday constant: F = 96485.3321 C/mol

Therefore, the number of electrons passing through the electron cathode is:

$$n_e = \frac{I \times t}{F} = 0.0035\,A \times 86400\,s \div 96485.3321\,C/mol = 0.003134\,mol = 3.134\,mmol$$

Theoretical hydrogen production:

$$n_{H_2} = \frac{n_e}{2} = 1.567\,mmol$$

Measured amount of hydrogen:

$$V_{mH_2} = 26.7\ mL = 0.0000267\ \mathrm{m}^3$$

$$n_{mH_2} = \frac{P \times V_{mH_2}}{RT} = \frac{99270\ Pa \times 0.0000267\ m^3}{8.3145\ J/(K \cdot mol) \times 298.15\ K} = 0.001069\ mol = 1.069\ mmol$$

Hydrogen generation efficiency:

$$efficiency = \frac{1.069\ mmol}{1.567\ mmol} = 68\%$$

9) Cyclic Voltammetry Studies

CV measurements were conducted with a Metrohm Autolab PGSTAT204 potentiostat and Nova 2.1 software. A glassy carbon working electrode (disk, diameter: 3mm), a coiled platinum wire counter electrode and a saturated calomel reference electrode (SCE) were employed.

The voltammograms were recorded at room temperature in HFIP (3 mL) at a substrate concentration of 25.0 mmol/L and with 100 mmol/L *n*-Bu_4NPF_6 as supporting electrolyte. The scan rate is 100 mV/s.

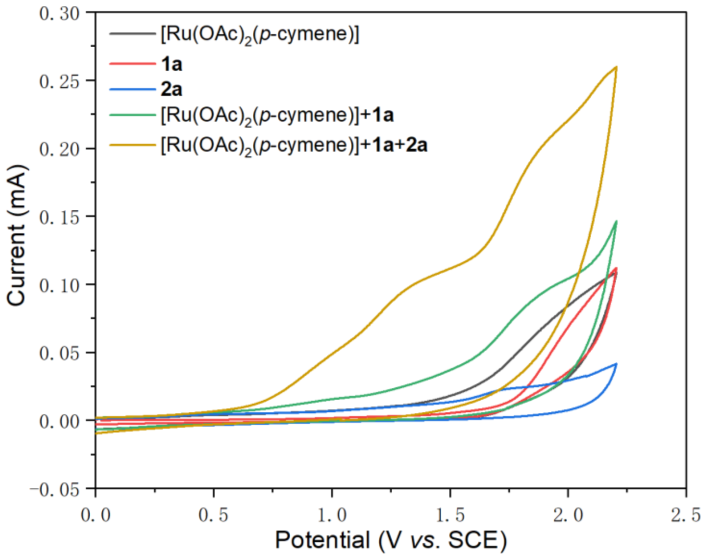

Figure S6. Cyclic voltammetry studies for *ortho*-C–H phosphorylation.

The voltammograms were recorded at room temperature in 5 mL H_2O/MeCN (1:1) at a substrate concentration of 15.0 mmol/L and with 60 mmol/L *n*-Bu_4NPF_6 as supporting electrolyte. The scan rate is 100 mV/s.

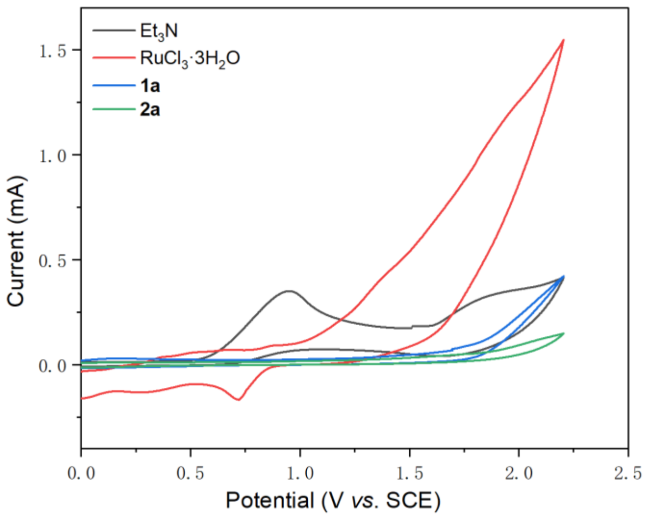

Figure S7. Cyclic voltammetry studies for *para*-C–H phosphorylation.

5. X-ray Crystallographic Data

X-ray crystallographic data of **3i**. (CCDC: 2360253)

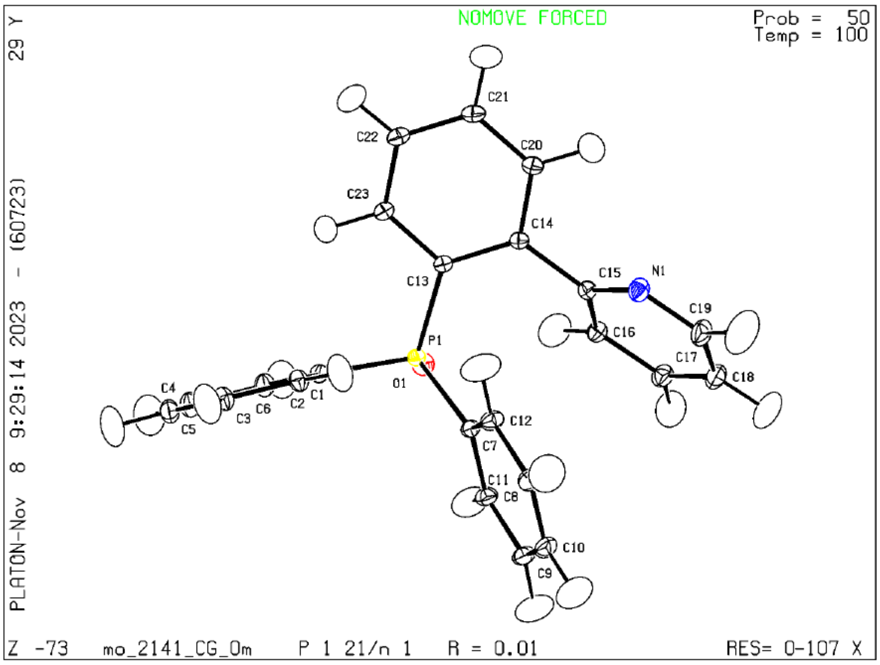

Bond precision:	C-C = 0.0004 A		Wavelength=0.71073
Cell:	a=8.8853(7)	b=21.2925(17)	c=9.3998(7)
	alpha=90	beta=102.203 (3)	gamma=90
Temperature:	100 K		
	Calculated		Reported
Volume	1738.2(2)		1738.2(2)
Space group	P 21/n		P 1 21/n 1
Hall group	-P 2yn		-P 2yn
Moiety formula	C23 H18 N O P		C23 H18 N O P
Sum formula	C23 H18 N O P		C23 H18 N O P
Mr	355.35		355.38
Dx,g cm-3	1.358		1.358
Z	4		4
Mu (mm-1)	0.170		0.170
F000	744.0		744.8
F000’	744.68		
h,k,lmax	13,33,14		13,33,14
Nref	6942		6932
Tmin,Tmax	0.924,0.932		0.912,0.976
Tmin’	0.924		

Correction method= # Reported T Limits: Tmin=0.912 Tmax=0.976
AbsCorr = NUMERICAL

Data completeness= 0.999 Theta(max)= 33.740

R(reflections)= 0.0113(6753) wR2(reflections)= 0.0275(6932)

S = 1.091 Npar= 397

X-ray crystallographic data of **4a**. (CCDC: 2360254)

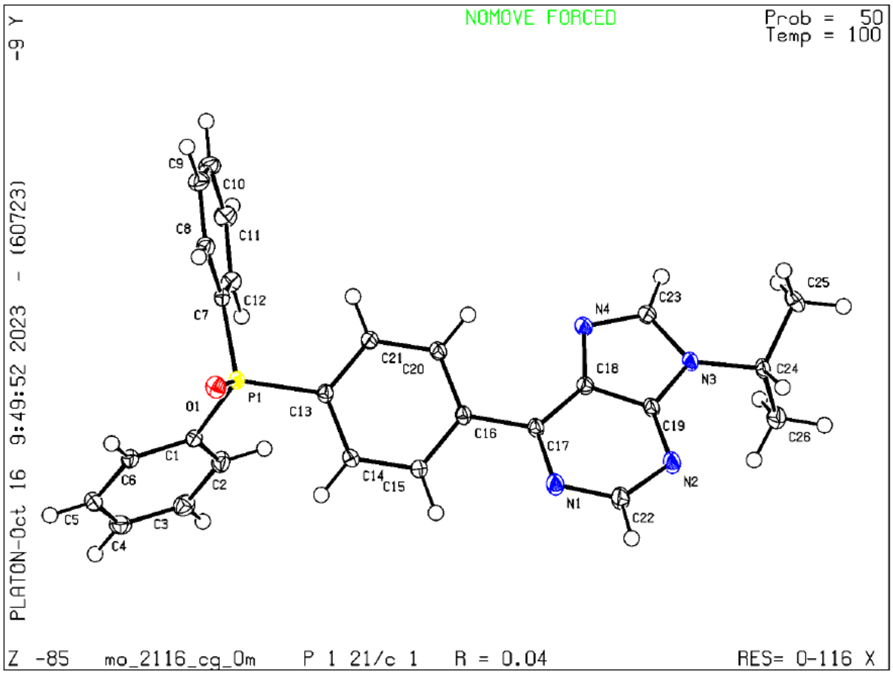

Bond precision:	C-C = 0.0016 A		Wavelength=0.71073
Cell:	a=9.7051(6)	b=22.4271(11)	c=10.9067(7)
	alpha=90	beta=113.919(2)	gamma=90
Temperature:	100 K		
	Calculated		Reported
Volume	2170.1(2)		2170.0(2)
Space group	P 21/c		P 1 21/c 1
Hall group	-P 2ybc		-P 2ybc
Moiety formula	C26 H23 N4 O P		C26 H23 N4 O P
Sum formula	C26 H23 N4 O P		C26 H23 N4 O P
Mr	438.45		438.45
Dx,g cm-3	1.342		1.342
Z	4		4
Mu (mm-1)	0.154		0.154
F000	920.0		920.0
F000’	920.71		
h,k,lmax	14,33,16		14,33,16
Nref	7253		6973
Tmin,Tmax	0.930,0.981		0.784,1.000
Tmin’	0.930		

Correction method= # Reported T Limits: Tmin=0.784 Tmax=1.000

AbsCorr = NUMERICAL

Data completeness= 0.961 Theta(max)= 33.551

R(reflections)= 0.0402(6320) wR2(reflections)= 0.1088(6973)

S = 1.024 Npar= 291

6. Computational Studies

All calculations were performed using the Gaussian 16, Revision A.03 package.[2] All structures were optimized at the PBE0[3, 4] level of theory in combination with D3 dispersion corrections with the Becke-Johnson damping scheme (D3BJ).[5, 6] Analytical frequency calculations were carried out at the same level of theory in order to identify each stationary point as either an intermediate (no imaginary frequencies) or a transition state (only one imaginary frequency) and to provided thermal and non-thermal corrections to the Gibbs free energy at 338.15 K and 1 atm. All atoms were described with the def2-SVP basis set, while ruthenium was in addition described with a SSD pseudopotential.[7-11] The electronic energy was then refined through PBE0 single-point calculations on the optimized geometries in combination with a standalone version of Grimme's most recent dispersion correction D4[12, 13] with a def2-TZVPP basis set combined with a SSD pseudopotential for ruthenium.[7-11] Solvent effects were included implicitly through the use of the SMD[14] model for HFIP the parameters were taken from Liu, Engle and coworkers.[15] Energies reported herein are based on gas-phase Gibbs free energies with a def2-SVP basis set for which the electronic energies were corrected to PBE0-D4 with a def2-TZVPP basis set and solvent effects.

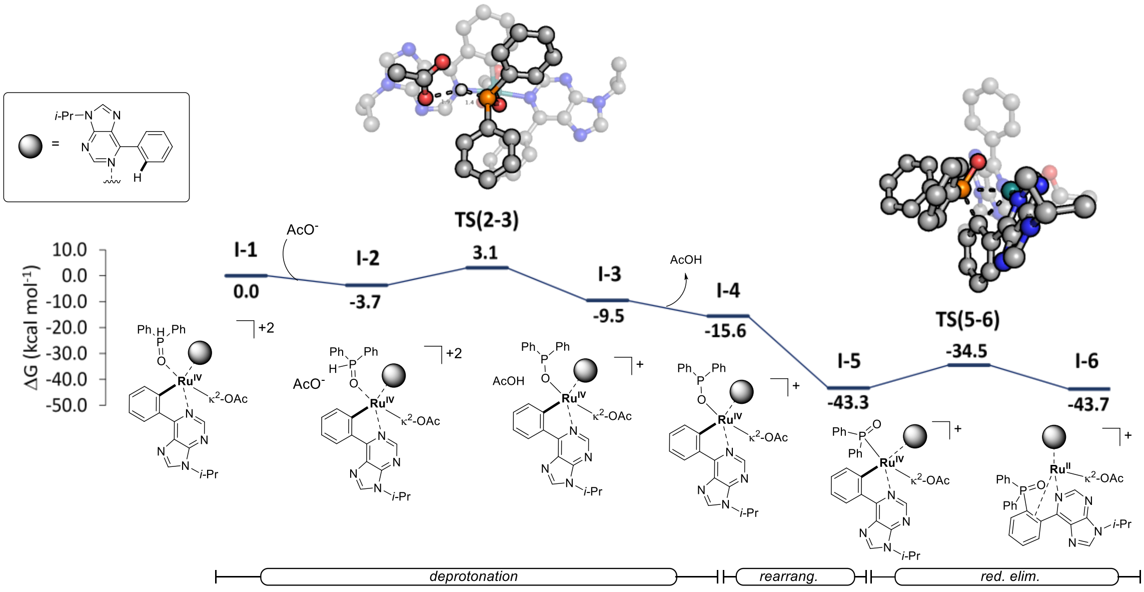

Figure S8. Computed relative Gibbs free energies ($\Delta G_{338.15}$) in kcal mol^{-1} for the ruthenium-catalyzed ortho-C–H phosphorylation at the PBE0-D4/def2-TZVPP-SMD(HFIP)//PBE0-D3BJ/def2-SVP level of theory.

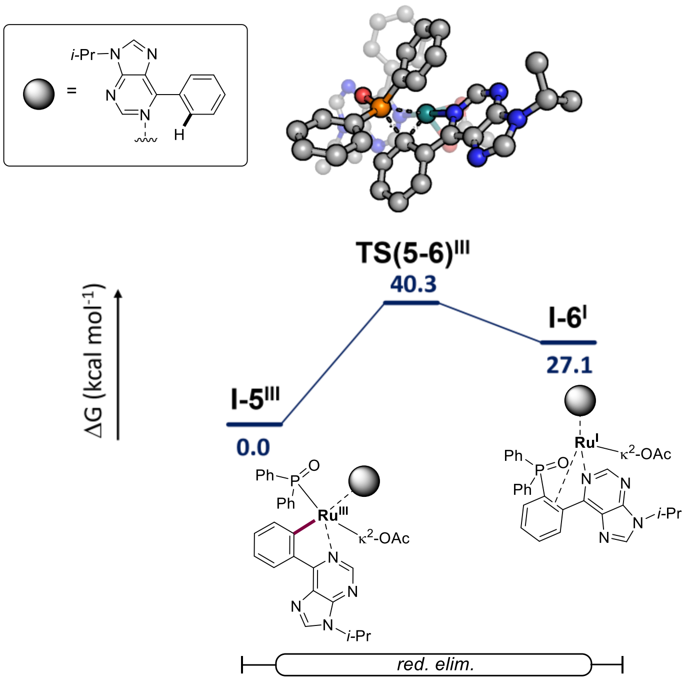

Figure S9. Computed relative Gibbs free energies ($\Delta G_{338.15}$) in kcal mol^{-1} for the ruthenium-catalyzed ortho-C–H phosphorylation reductive elimination step through a ruthenium(III) pathway at the PBE0-D4/def2-TZVPP-SMD(HFIP)//PBE0-D3BJ/def2-SVP level of theory.

Table S3. Calculated electronic energies at the PBE0-D4/def2-TZVPP-SMD(HFIP) level of theory and Gibbs free energies with dispersion corrections for all structures in the present work (all in Hartree).

Structure	Electronic Energy	Total Gibbs Free Energy
**I-1**	-2723.228119	-2722.561087
**I-2**	-2951.725394	-2951.014093
**TS(2-3)**	-2951.710840	-2951.003381
**I-3**	-2951.731412	-2951.023461
**I-4**	-2722.780299	-2722.124228
**I-5**	-2722.827029	-2722.168375
**TS(5-6)**	-2722.812487	-2722.154319
**I-6**	-2722.827574	-2722.169027
**I-5^{III}**	-2722.989976	-2722.335678
**TS(5-6)^{III}**	-2722.924999	-2722.271498
**I-6^{I}**	-2722.946189	-2722.292517
**Acetate**	-228.463443	-228.447157
**Acetic Acid**	-228.939683	-228.908851

Cartesian coordinates of the optimized structures

I-1

Lowest frequency = 12.4224 cm^{-1}

Charge = 2, Multiplicity = 1

96

Ru	-0.205080	-1.257346	-0.239294
O	0.230397	-2.725443	-1.613169
C	-0.161583	-3.673466	-0.860006
O	-0.575914	-3.246258	0.256475
C	-0.106264	-5.097317	-1.235002
H	-0.812539	-5.680260	-0.631052
H	-0.312404	-5.215244	-2.307144
H	0.912223	-5.471228	-1.041852
N	-2.317288	-1.177931	-0.400698
C	-2.836154	-0.167919	-1.150216
C	-3.135128	-1.953855	0.354580
C	-4.200131	0.093001	-1.014144
C	-1.948702	0.558364	-2.059765
N	-4.433024	-1.806357	0.494433
H	-2.652067	-2.776805	0.884110
C	-4.941895	-0.778895	-0.177219
N	-5.021468	1.030621	-1.571832
C	-0.918983	-0.143593	-2.713043
C	-2.070677	1.940121	-2.242434
N	-6.229266	-0.345323	-0.249887
C	-6.203975	0.739216	-1.094801
C	0.007529	0.543899	-3.501818
H	-0.925168	-1.236762	-2.722277
C	-1.131423	2.617317	-3.013123
H	-2.891285	2.478451	-1.768937
C	-7.418602	-0.917862	0.405876
H	-7.121351	1.284489	-1.325765
C	-0.085126	1.926549	-3.634248
H	0.774758	-0.008180	-4.049846
H	-1.220300	3.698472	-3.140661
C	-7.678195	-2.326999	-0.104164
C	-7.280883	-0.837333	1.918261
H	-8.245159	-0.263613	0.086391
H	0.632010	2.467340	-4.256013
H	-8.615099	-2.706352	0.327137

H	-7.777724	-2.343317	-1.199086
H	-6.866303	-3.007244	0.190744
H	-6.456669	-1.473813	2.271157
H	-8.208920	-1.188846	2.390302
H	-7.103152	0.196376	2.249126
N	1.858002	-1.130799	-0.259718
C	2.482043	-1.081952	0.943695
C	2.564725	-1.057433	-1.410477
C	3.867838	-0.929220	0.942134
C	1.583131	-1.189711	2.066103
N	3.869042	-0.930666	-1.499123
H	1.986522	-1.123496	-2.332914
C	4.495154	-0.868102	-0.327101
N	4.794629	-0.838405	1.939300
C	1.973851	-1.134438	3.411945
C	0.225448	-1.337492	1.719885
N	5.827051	-0.749017	-0.066016
C	5.934847	-0.738194	1.303886
C	1.002208	-1.221039	4.402698
H	3.034278	-1.026920	3.651950
C	-0.740888	-1.423957	2.716515
C	6.940744	-0.727090	-1.030765
H	6.911514	-0.654714	1.784558
C	-0.344973	-1.360700	4.055884
H	1.292050	-1.185111	5.454736
H	-1.799287	-1.530831	2.478596
C	7.015592	-2.050207	-1.777806
C	6.832973	0.477063	-1.952727
H	7.840767	-0.618485	-0.405106
H	-1.103403	-1.428553	4.839542
H	7.901996	-2.056823	-2.427389
H	7.099244	-2.896936	-1.081342
H	6.127470	-2.194312	-2.409914
H	5.926581	0.416180	-2.571978
H	7.703731	0.504511	-2.622408
H	6.817408	1.416436	-1.382058
O	-0.394283	0.676205	0.156247
P	0.204717	1.760071	1.115983
H	0.110763	1.307274	2.452821
C	-0.804564	3.215774	0.912144
C	-0.230382	4.465669	0.652660
C	-2.198224	3.071236	0.996152
C	-1.057505	5.572701	0.473254
H	0.854690	4.576243	0.589240

C	-3.012488	4.182446	0.815827
H	-2.644171	2.091682	1.188499
C	-2.441967	5.430765	0.553047
H	-0.617360	6.551346	0.271452
H	-4.097704	4.078267	0.876958
H	-3.085446	6.302238	0.412648
C	1.928003	2.105581	0.826463
C	2.803603	2.249288	1.909754
C	2.383723	2.246033	-0.493022
C	4.142292	2.548017	1.670898
H	2.448939	2.122999	2.936113
C	3.725701	2.533554	-0.718811
H	1.692849	2.128209	-1.332615
C	4.598272	2.691099	0.361006
H	4.829863	2.665550	2.510504
H	4.094421	2.641592	-1.740887
H	5.647045	2.935940	0.178385

I-2

Lowest frequency = 13.4853 cm^{-1}

Charge = 1, Multiplicity = 1

103

Ru	-0.246206	-0.986907	-1.079319
O	0.083199	-1.447083	-3.086147
C	-0.381522	-2.630916	-2.997549
O	-0.788305	-2.945796	-1.851608
C	-0.397540	-3.554556	-4.157843
H	-1.116730	-4.365094	-3.990454
H	-0.632850	-3.003676	-5.077908
H	0.606656	-3.991275	-4.273711
N	-2.347460	-0.604412	-1.177391
C	-2.813249	0.668100	-1.272808
C	-3.180637	-1.612680	-0.825597
C	-4.129267	0.886165	-0.842018
C	-1.979150	1.726650	-1.847496
N	-4.434005	-1.497902	-0.442557
H	-2.744739	-2.612406	-0.864940
C	-4.869435	-0.242606	-0.424350
N	-4.894506	2.017899	-0.734398
C	-1.097935	1.424809	-2.897080
C	-2.074949	3.047376	-1.388037

N	-6.092581	0.234154	-0.054798
C	-6.034602	1.592152	-0.262230
C	-0.299237	2.419278	-3.451012
H	-1.069176	0.417376	-3.315229
C	-1.269219	4.035051	-1.942485
H	-2.786098	3.296267	-0.600222
C	-7.232676	-0.528743	0.465365
H	-6.894963	2.225680	-0.038248
C	-0.376017	3.724491	-2.967055
H	0.372764	2.176591	-4.277425
H	-1.336348	5.058092	-1.568706
C	-7.735611	-1.517740	-0.575803
C	-6.867894	-1.185692	1.789050
H	-8.012766	0.228399	0.645411
H	0.252246	4.506669	-3.398108
H	-8.634772	-2.026607	-0.200529
H	-7.997635	-1.006270	-1.513291
H	-6.971047	-2.278146	-0.789624
H	-6.076188	-1.934873	1.644081
H	-7.748579	-1.691285	2.209729
H	-6.520406	-0.439803	2.519300
N	1.785562	-1.229364	-1.013962
C	2.277515	-1.995972	-0.007343
C	2.601040	-0.524422	-1.823521
C	3.661916	-1.999725	0.164108
C	1.258319	-2.552540	0.851498
N	3.907882	-0.436821	-1.700132
H	2.110204	0.028943	-2.628399
C	4.403523	-1.151290	-0.694877
N	4.478590	-2.557340	1.106173
C	1.525903	-3.348292	1.973019
C	-0.059418	-2.137308	0.552344
N	5.685243	-1.207859	-0.238472
C	5.656736	-2.057912	0.837894
C	0.474771	-3.731986	2.798171
H	2.557813	-3.634800	2.186322
C	-1.095142	-2.504617	1.406718
C	6.835612	-0.423108	-0.702049
H	6.567882	-2.272432	1.399105
C	-0.823871	-3.301536	2.520896
H	0.669163	-4.356483	3.672459
H	-2.114421	-2.153316	1.250785
C	7.195263	-0.792562	-2.132859
C	6.567900	1.063310	-0.509787

H	7.659366	-0.726832	-0.036072
H	-1.643285	-3.577012	3.188815
H	8.095199	-0.242451	-2.442578
H	7.401409	-1.868647	-2.227518
H	6.376980	-0.527350	-2.817705
H	5.794537	1.408507	-1.210649
H	7.488674	1.633208	-0.699263
H	6.214967	1.271643	0.509767
O	-0.172794	0.529482	-0.042539
P	0.843738	0.986719	1.320716
H	1.769303	-0.010959	1.031996
C	-0.534884	0.679820	2.459824
C	-1.833811	1.042575	2.079135
C	-0.325990	0.011838	3.669899
C	-2.912836	0.731076	2.900567
H	-1.994030	1.567369	1.137874
C	-1.413283	-0.318593	4.475387
H	0.685248	-0.257307	3.974632
C	-2.705718	0.038345	4.093856
H	-3.920788	1.031753	2.604060
H	-1.246763	-0.855217	5.411967
H	-3.553849	-0.216002	4.734015
C	1.089973	2.649427	0.658904
C	0.457179	3.737623	1.267541
C	1.983243	2.851087	-0.396227
C	0.712824	5.027163	0.808592
H	-0.222193	3.580867	2.108842
C	2.242819	4.145879	-0.839148
H	2.503817	2.007008	-0.851157
C	1.608314	5.233004	-0.240821
H	0.219262	5.878053	1.283648
H	2.952459	4.304883	-1.654185
H	1.817560	6.247281	-0.588678
O	3.755141	0.936614	1.560887
C	3.221917	1.293844	2.597059
O	1.914218	1.389975	2.745587
C	3.981090	1.691176	3.828588
H	3.539692	1.226558	4.720883
H	5.037303	1.416167	3.730197
H	3.897583	2.781322	3.956815

TS(2-3)

Lowest frequency = -173.7764 cm^{-1}

Charge = 1, Multiplicity = 1

103

Ru	-0.321971	-1.302334	-0.650503
O	0.067200	-2.583381	-2.226040
C	-0.379525	-3.615443	-1.629145
O	-0.807715	-3.373339	-0.470607
C	-0.354355	-4.963477	-2.238053
H	-1.099595	-5.611211	-1.760808
H	-0.524851	-4.895689	-3.320291
H	0.644253	-5.399546	-2.077431
N	-2.439617	-1.079412	-0.852263
C	-2.912984	0.025171	-1.488119
C	-3.274392	-1.857928	-0.122822
C	-4.242359	0.377952	-1.234236
C	-2.035550	0.763520	-2.398084
N	-4.541425	-1.616707	0.134922
H	-2.829890	-2.767867	0.284075
C	-4.991100	-0.486799	-0.402009
N	-5.017665	1.426921	-1.649198
C	-1.120653	0.058200	-3.196064
C	-2.058682	2.162712	-2.443864
N	-6.232489	0.069299	-0.319281
C	-6.174726	1.211131	-1.084304
C	-0.201248	0.747061	-3.983612
H	-1.164210	-1.031146	-3.245168
C	-1.129166	2.842584	-3.222103
H	-2.787716	2.711030	-1.846788
C	-7.393370	-0.430690	0.427889
H	-7.049721	1.856228	-1.184676
C	-0.190384	2.140497	-3.980079
H	0.497062	0.192293	-4.614671
H	-1.125098	3.934055	-3.226008
C	-7.842006	-1.777444	-0.119599
C	-7.089114	-0.459724	1.918773
H	-8.183639	0.313469	0.239012
H	0.539145	2.684612	-4.583639
H	-8.756548	-2.101223	0.397050
H	-8.060801	-1.714999	-1.195409
H	-7.066607	-2.540166	0.040504
H	-6.291479	-1.184282	2.137885
H	-7.987914	-0.759093	2.476092
H	-6.778480	0.532135	2.279390

N	1.723235	-1.319082	-0.591746
C	2.309733	-1.583911	0.603860
C	2.460433	-1.028235	-1.684552
C	3.699745	-1.588404	0.641001
C	1.361477	-1.739389	1.678644
N	3.770537	-0.960218	-1.720899
H	1.899264	-0.836053	-2.601761
C	4.357782	-1.215626	-0.557597
N	4.601920	-1.750321	1.651759
C	1.706340	-1.878005	3.027073
C	0.008519	-1.656583	1.285387
N	5.677526	-1.149353	-0.238403
C	5.751015	-1.470621	1.094493
C	0.693863	-1.957337	3.975835
H	2.762158	-1.892286	3.302344
C	-0.997564	-1.746902	2.244683
C	6.777842	-0.675945	-1.085401
H	6.712710	-1.480042	1.610263
C	-0.646348	-1.891066	3.587049
H	0.948857	-2.058505	5.032514
H	-2.050848	-1.668674	1.980932
C	7.053364	-1.665684	-2.206961
C	6.491675	0.734502	-1.583341
H	7.648131	-0.647007	-0.410034
H	-1.435704	-1.935962	4.340562
H	7.913847	-1.325478	-2.800709
H	7.282764	-2.665910	-1.811306
H	6.183302	-1.740295	-2.875571
H	5.704875	0.718222	-2.350208
H	7.403125	1.163747	-2.023239
H	6.138543	1.376315	-0.764453
O	-0.328178	0.578444	-0.287175
P	0.536118	1.481558	0.729519
H	1.892620	1.053751	0.925600
C	-0.446858	1.503675	2.229162
C	-1.844864	1.434199	2.122756
C	0.178284	1.566041	3.479557
C	-2.620408	1.436001	3.276286
H	-2.316730	1.358589	1.139908
C	-0.615920	1.565203	4.626595
H	1.272439	1.585008	3.533105
C	-2.004517	1.501965	4.528515
H	-3.709213	1.381873	3.201745
H	-0.138739	1.609478	5.608143

H	-2.615203	1.502370	5.434616
C	0.622400	3.106408	-0.013519
C	-0.446931	4.008203	0.061068
C	1.791788	3.434290	-0.714760
C	-0.355307	5.234279	-0.588504
H	-1.341510	3.758440	0.636645
C	1.867258	4.664110	-1.366366
H	2.642597	2.743266	-0.702555
C	0.798042	5.557179	-1.307757
H	-1.180785	5.946937	-0.527278
H	2.776334	4.932973	-1.908810
H	0.868704	6.523884	-1.812318
O	3.742535	1.296887	0.515865
C	3.891856	1.390508	1.767210
O	2.989333	1.132492	2.593148
C	5.233484	1.851087	2.310558
H	5.123165	2.867283	2.719676
H	5.533645	1.201843	3.145114
H	6.013710	1.866654	1.538264

I-3

Lowest frequency = 9.3613 cm^{-1}

Charge = 1, Multiplicity = 1

103

Ru	0.292215	0.976236	-1.024824
O	-0.063138	1.628059	-2.961923
C	0.324822	2.824916	-2.750411
O	0.700793	3.042815	-1.572984
C	0.291661	3.858375	-3.812643
H	0.950261	4.694965	-3.550818
H	0.577421	3.419994	-4.777854
H	-0.739061	4.234648	-3.905242
N	2.422785	0.792940	-1.104400
C	2.985007	-0.426230	-1.315710
C	3.176038	1.822067	-0.646949
C	4.314162	-0.584873	-0.904934
C	2.221889	-1.486777	-1.977287
N	4.435437	1.764991	-0.271269
H	2.664723	2.784266	-0.587295
C	4.968365	0.552110	-0.377909
N	5.164554	-1.658581	-0.913559

C	1.334824	-1.160308	-3.014221
C	2.365018	-2.824222	-1.585061
N	6.227504	0.137482	-0.060394
C	6.272160	-1.193918	-0.403122
C	0.566922	-2.152085	-3.617220
H	1.274139	-0.133583	-3.379786
C	1.590870	-3.808002	-2.187698
H	3.072212	-3.084812	-0.797666
C	7.312042	0.930933	0.529062
H	7.181266	-1.777256	-0.244868
C	0.684003	-3.475300	-3.194376
H	-0.111451	-1.891996	-4.433247
H	1.685326	-4.844172	-1.858692
C	7.730611	2.049323	-0.413876
C	6.911269	1.429384	1.910130
H	8.147764	0.220583	0.634463
H	0.077515	-4.255113	-3.660251
H	8.592308	2.586494	0.007051
H	8.022267	1.650615	-1.396300
H	6.909770	2.767288	-0.552973
H	6.066730	2.129995	1.839175
H	7.756556	1.954951	2.376436
H	6.624981	0.593389	2.565445
N	-1.750304	0.990473	-0.995440
C	-2.344321	1.631948	0.042038
C	-2.472273	0.339271	-1.929913
C	-3.740508	1.629880	0.065352
C	-1.412289	2.150156	1.010540
N	-3.783616	0.243856	-1.949102
H	-1.898947	-0.148729	-2.722192
C	-4.385247	0.889127	-0.953232
N	-4.659145	2.136660	0.941236
C	-1.782783	2.747817	2.220993
C	-0.052418	1.925692	0.697182
N	-5.716218	0.961786	-0.666117
C	-5.807084	1.720094	0.474006
C	-0.791039	3.122863	3.119427
H	-2.841973	2.884583	2.445532
C	0.928587	2.301585	1.610380
C	-6.820692	0.297435	-1.367199
H	-6.778681	1.936316	0.921770
C	0.552535	2.893501	2.817687
H	-1.066118	3.581602	4.070999
H	1.984468	2.108579	1.428222

C	-6.950915	0.827118	-2.787060
C	-6.650229	-1.214022	-1.302772
H	-7.719298	0.581245	-0.795815
H	1.326544	3.165300	3.538780
H	-7.822803	0.369460	-3.275614
H	-7.087931	1.918187	-2.793144
H	-6.056048	0.579174	-3.375740
H	-5.775569	-1.528508	-1.889593
H	-7.541578	-1.707132	-1.715738
H	-6.508571	-1.554390	-0.266994
O	0.326695	-0.669141	-0.165553
P	-0.695936	-1.252055	1.043093
H	-2.991228	-1.008455	1.344273
C	0.403761	-0.871668	2.447052
C	1.800130	-0.900523	2.304327
C	-0.167463	-0.459665	3.654963
C	2.615202	-0.528697	3.368098
H	2.240228	-1.202219	1.351328
C	0.658428	-0.098808	4.720647
H	-1.255179	-0.393437	3.749386
C	2.044033	-0.129427	4.579244
H	3.702355	-0.550699	3.255703
H	0.210832	0.221381	5.664331
H	2.685218	0.161017	5.415089
C	-0.488406	-3.039183	0.742437
C	0.455886	-3.854743	1.377210
C	-1.382417	-3.610293	-0.174544
C	0.513047	-5.215786	1.084488
H	1.140766	-3.427880	2.112815
C	-1.317956	-4.968502	-0.472829
H	-2.141517	-2.984441	-0.654621
C	-0.369573	-5.772881	0.158678
H	1.246598	-5.848666	1.589458
H	-2.018510	-5.404362	-1.188841
H	-0.326034	-6.842165	-0.061900
O	-3.968989	-1.063553	1.487800
C	-4.235758	-0.559602	2.693535
O	-3.385104	-0.105603	3.418968
C	-5.686756	-0.655871	3.059872
H	-5.838832	-1.585055	3.631109
H	-5.954245	0.187236	3.707851
H	-6.332998	-0.692876	2.174405

I-4

Lowest frequency = 12.8958 cm^{-1}

Charge = 1, Multiplicity = 1

95

Ru	-0.121660	-1.211113	-0.350593
O	0.285972	-2.398653	-2.085163
C	-0.062310	-3.470609	-1.504592
O	-0.434379	-3.324712	-0.307920
C	-0.004017	-4.794136	-2.173235
H	-0.628919	-5.521859	-1.642135
H	-0.315096	-4.700968	-3.222050
H	1.038578	-5.148084	-2.162168
N	-2.244277	-1.114633	-0.577193
C	-2.779307	-0.003920	-1.147925
C	-3.036476	-2.004449	0.063147
C	-4.143126	0.220327	-0.931685
C	-1.943001	0.869885	-1.979181
N	-4.330152	-1.888688	0.276894
H	-2.530585	-2.900553	0.428633
C	-4.851141	-0.764930	-0.204621
N	-4.988452	1.227876	-1.312894
C	-0.963588	0.302874	-2.808812
C	-2.090729	2.261396	-1.936402
N	-6.140895	-0.324623	-0.151474
C	-6.147617	0.871824	-0.829509
C	-0.109422	1.119562	-3.546545
H	-0.901103	-0.781348	-2.920792
C	-1.225399	3.070650	-2.665311
H	-2.863898	2.706642	-1.311530
C	-7.291016	-0.984323	0.477663
H	-7.071294	1.444393	-0.933721
C	-0.228304	2.506137	-3.462886
H	0.637881	0.669232	-4.204270
H	-1.330638	4.155697	-2.605037
C	-7.585492	-2.310722	-0.207511
C	-7.071065	-1.120599	1.977136
H	-8.133536	-0.296327	0.301429
H	0.442822	3.149697	-4.036383
H	-8.494008	-2.756556	0.221566
H	-7.748453	-2.172218	-1.286176
H	-6.753727	-3.014976	-0.062882
H	-6.224706	-1.790947	2.185104

H	-7.969628	-1.544209	2.447602
H	-6.871173	-0.142924	2.439554
N	1.918631	-1.093934	-0.309012
C	2.506314	-1.179070	0.909611
C	2.646048	-0.853071	-1.421447
C	3.888681	-0.979441	0.962871
C	1.582413	-1.463934	1.977811
N	3.949226	-0.684505	-1.461467
H	2.084405	-0.811556	-2.356405
C	4.538748	-0.744269	-0.269989
N	4.792176	-0.975864	1.988771
C	1.947190	-1.603434	3.324716
C	0.235245	-1.596371	1.570144
N	5.862626	-0.610702	0.038306
C	5.939646	-0.756613	1.401729
C	0.963522	-1.877353	4.265259
H	2.997136	-1.493449	3.604148
C	-0.738887	-1.864286	2.528597
C	6.988546	-0.447145	-0.888748
H	6.901275	-0.691213	1.913857
C	-0.370332	-2.004763	3.867122
H	1.231146	-1.989028	5.317799
H	-1.790837	-1.949260	2.256259
C	7.114059	-1.671028	-1.785194
C	6.867103	0.851403	-1.670690
H	7.875911	-0.389652	-0.237980
H	-1.141171	-2.210251	4.613887
H	8.008617	-1.579610	-2.417407
H	7.207803	-2.590793	-1.189694
H	6.236124	-1.762051	-2.440932
H	5.962683	0.844127	-2.295561
H	7.741406	0.971229	-2.325914
H	6.823767	1.716526	-0.995117
O	-0.357419	0.546559	0.253186
P	0.143756	1.455476	1.531087
C	-0.914077	2.914842	1.228792
C	-0.449801	4.155523	0.782305
C	-2.283655	2.739523	1.475756
C	-1.349716	5.197538	0.558522
H	0.615967	4.308411	0.599215
C	-3.180785	3.776123	1.238837
H	-2.648350	1.776323	1.845540
C	-2.713487	5.007802	0.776371
H	-0.981621	6.164910	0.208539

H	-4.247893	3.626873	1.418046
H	-3.414795	5.825798	0.596378
C	1.811845	2.006077	1.043495
C	2.771674	2.176972	2.047039
C	2.155657	2.234075	-0.296859
C	4.058868	2.600297	1.720202
H	2.510397	1.970774	3.088636
C	3.446186	2.643200	-0.620495
H	1.406944	2.092400	-1.081261
C	4.393408	2.835436	0.388028
H	4.801785	2.741569	2.508184
H	3.715160	2.819506	-1.664628
H	5.398334	3.178522	0.132041

I-5

Lowest frequency = 15.4997 cm^{-1}

Charge = 1, Multiplicity = 1

95

Ru	0.166255	0.496813	-0.842323
O	0.448816	2.209553	-2.142399
C	0.358342	1.453764	-3.149902
O	0.157929	0.222000	-2.910127
C	0.508236	1.956687	-4.540536
H	0.070829	1.250657	-5.256231
H	0.046385	2.948028	-4.633451
H	1.581724	2.063078	-4.761058
N	-1.993025	0.680639	-0.959993
C	-2.808022	1.406223	-0.141234
C	-2.518438	-0.293151	-1.742632
C	-4.129816	0.940054	-0.016006
C	-2.361178	2.642348	0.513845
N	-3.746728	-0.763059	-1.712159
H	-1.827079	-0.725920	-2.467236
C	-4.513732	-0.163582	-0.807957
N	-5.191376	1.353769	0.748299
C	-1.395261	3.457537	-0.091969
C	-2.957636	3.073394	1.712078
N	-5.817023	-0.415706	-0.493396
C	-6.154378	0.527056	0.446556
C	-0.999166	4.647276	0.506987
H	-0.961225	3.170728	-1.047597

C	-2.547627	4.257212	2.313612
H	-3.755925	2.482499	2.157979
C	-6.682751	-1.466913	-1.035613
H	-7.156762	0.548924	0.878224
C	-1.561435	5.044409	1.718845
H	-0.248998	5.272085	0.017313
H	-3.011265	4.575199	3.250033
C	-6.907484	-1.262552	-2.526656
C	-6.120331	-2.840345	-0.697600
H	-7.641481	-1.335296	-0.508443
H	-1.245853	5.977297	2.191857
H	-7.614792	-2.015161	-2.903112
H	-7.326365	-0.266450	-2.730150
H	-5.962487	-1.367151	-3.078480
H	-5.157011	-2.997425	-1.204117
H	-6.817242	-3.622391	-1.030767
H	-5.971729	-2.949353	0.386861
N	2.196264	0.395496	-0.745461
C	2.760474	-0.827313	-0.894926
C	2.935890	1.486709	-0.458287
C	4.149773	-0.900454	-0.754825
C	1.817716	-1.903962	-1.102353
N	4.240783	1.497216	-0.284357
H	2.379829	2.421908	-0.357320
C	4.813977	0.306330	-0.434648
N	5.042068	-1.936281	-0.809234
C	2.214420	-3.220899	-1.371835
C	0.452483	-1.555289	-0.970392
N	6.130824	-0.030640	-0.297651
C	6.193314	-1.381345	-0.535648
C	1.251084	-4.211909	-1.512168
H	3.279860	-3.444286	-1.455771
C	-0.494900	-2.570386	-1.104382
C	7.248418	0.850261	0.058409
H	7.147238	-1.909968	-0.490784
C	-0.098078	-3.882904	-1.379377
H	1.548882	-5.240418	-1.724882
H	-1.559822	-2.362606	-1.001695
C	7.430281	1.936727	-0.990969
C	7.059179	1.401055	1.464723
H	8.132829	0.192989	0.048377
H	-0.861357	-4.657574	-1.489263
H	8.316925	2.540309	-0.750572
H	7.573066	1.501137	-1.990507

H	6.555305	2.601738	-1.016504
H	6.168594	2.044358	1.511152
H	7.933931	2.001448	1.752008
H	6.948452	0.586526	2.195799
P	0.040427	-0.281621	1.250908
O	0.216527	1.263825	1.182722
C	-1.493156	-0.811746	2.020181
C	-2.140042	0.124990	2.836470
C	-2.049485	-2.082497	1.822352
C	-3.354123	-0.204663	3.432200
H	-1.697250	1.111179	2.987345
C	-3.266808	-2.398039	2.419466
H	-1.543822	-2.823446	1.202943
C	-3.921584	-1.459492	3.217593
H	-3.861647	0.526937	4.064315
H	-3.703901	-3.386736	2.263308
H	-4.876554	-1.712170	3.684002
C	1.469792	-1.073617	1.994513
C	2.499474	-0.243008	2.454711
C	1.604376	-2.466341	2.040516
C	3.667913	-0.812801	2.953221
H	2.378269	0.841370	2.411969
C	2.778823	-3.024267	2.534443
H	0.811890	-3.117689	1.665387
C	3.810189	-2.199717	2.985761
H	4.470747	-0.167525	3.316851
H	2.893984	-4.109636	2.558420
H	4.731169	-2.644787	3.369055

TS(5-6)

Lowest frequency = -127.7435 cm^{-1}

Charge = 1, Multiplicity = 1

95

scf done: -2720.126539

Ru	-0.001914	-1.066800	-0.009383
O	-0.106245	-3.056401	0.660761
C	-0.078348	-3.479472	-0.536405
O	-0.018087	-2.594559	-1.440050
C	-0.085423	-4.934297	-0.848852
H	-0.410273	-5.104607	-1.882083
H	0.937569	-5.324539	-0.731401
H	-0.729879	-5.471248	-0.140988

N	2.123810	-1.101716	-0.013966
C	2.979046	-0.836558	1.009266
C	2.600091	-1.216092	-1.281288
C	4.319505	-0.589537	0.655302
C	2.550589	-0.839543	2.415368
N	3.836532	-1.025991	-1.689335
H	1.855484	-1.494503	-2.029792
C	4.668928	-0.698051	-0.707280
N	5.429226	-0.272160	1.396642
C	1.579118	-1.740075	2.872658
C	3.161023	0.031802	3.333974
N	6.007265	-0.434488	-0.773042
C	6.393235	-0.186740	0.521346
C	1.200573	-1.741747	4.210270
H	1.130114	-2.452472	2.182581
C	2.764046	0.037148	4.666450
H	3.956651	0.697373	2.999480
C	6.864208	-0.438989	-1.961702
H	7.431687	0.055179	0.755509
C	1.778555	-0.845342	5.107803
H	0.447845	-2.454363	4.554674
H	3.236369	0.727781	5.368531
C	6.931241	-1.833724	-2.567194
C	6.404863	0.620266	-2.953412
H	7.862860	-0.164848	-1.584897
H	1.473313	-0.844628	6.156869
H	7.633859	-1.840289	-3.412669
H	7.278027	-2.569507	-1.827216
H	5.942475	-2.143028	-2.935272
H	5.402076	0.379993	-3.335198
H	7.098932	0.660329	-3.804950
H	6.377815	1.615201	-2.485212
N	-2.036753	-0.986442	-0.154677
C	-2.524579	-0.275298	-1.198110
C	-2.872634	-1.574812	0.726600
C	-3.908014	-0.196148	-1.345296
C	-1.512877	0.384064	-2.021279
N	-4.190734	-1.515531	0.688747
H	-2.388643	-2.136953	1.529035
C	-4.675949	-0.830276	-0.337292
N	-4.720646	0.431894	-2.246349
C	-1.681172	0.574350	-3.388708
C	-0.279489	0.729882	-1.386376
N	-5.978552	-0.561991	-0.658021

C	-5.928677	0.188730	-1.804393
C	-0.615533	1.052190	-4.155200
H	-2.635814	0.316769	-3.850390
C	0.773864	1.204218	-2.189423
C	-7.182814	-0.966098	0.076459
H	-6.847800	0.537006	-2.279383
C	0.615520	1.339516	-3.565038
H	-0.745989	1.185618	-5.231574
H	1.721825	1.481472	-1.724426
C	-7.299908	-2.482363	0.119233
C	-7.199681	-0.324803	1.456621
H	-8.017920	-0.559794	-0.516723
H	1.450745	1.686599	-4.176568
H	-8.243601	-2.767904	0.605130
H	-7.293633	-2.908970	-0.894261
H	-6.469946	-2.919611	0.692367
H	-6.363326	-0.696723	2.065917
H	-8.138960	-0.572141	1.971142
H	-7.124782	0.770000	1.383980
P	-0.328454	1.387341	0.416633
O	-0.068772	0.409009	1.580728
C	0.890325	2.713799	0.472263
C	1.827517	2.646918	1.507893
C	0.894508	3.796346	-0.417897
C	2.783181	3.653328	1.638313
H	1.794733	1.807521	2.205006
C	1.846601	4.799033	-0.275239
H	0.165603	3.858651	-1.228680
C	2.793936	4.725338	0.749435
H	3.518386	3.597236	2.444189
H	1.851741	5.644876	-0.965996
H	3.540984	5.515394	0.855156
C	-2.001616	2.058187	0.558264
C	-2.727250	1.693899	1.697490
C	-2.582434	2.875198	-0.419122
C	-4.040302	2.136535	1.846982
H	-2.256928	1.053969	2.447355
C	-3.891361	3.316652	-0.260117
H	-2.032866	3.141244	-1.324598
C	-4.621253	2.943329	0.869925
H	-4.611298	1.846062	2.731556
H	-4.348726	3.943824	-1.027727
H	-5.650848	3.289144	0.989639

I-6

Lowest frequency = 14.1755 cm^{-1}

Charge = 1, Multiplicity = 1

95

Ru	0.014496	-0.854164	0.750671
O	0.053725	-1.731913	2.651348
C	0.087356	-2.891870	2.137066
O	0.063246	-2.941787	0.870074
C	0.182983	-4.117107	2.974042
H	-0.165172	-4.993719	2.414937
H	1.237936	-4.271577	3.249790
H	-0.389766	-3.989448	3.901475
N	2.139221	-0.927546	0.543620
C	3.076972	-0.033124	0.953705
C	2.493337	-1.937310	-0.293960
C	4.363568	-0.173588	0.399034
C	2.798092	0.999210	1.961473
N	3.675265	-2.156014	-0.828977
H	1.695199	-2.639577	-0.537327
C	4.583690	-1.253336	-0.481980
N	5.526147	0.536582	0.563647
C	1.922407	0.748051	3.026967
C	3.480954	2.226719	1.912235
N	5.895927	-1.175199	-0.850887
C	6.395233	-0.080340	-0.188807
C	1.711619	1.714122	4.003981
H	1.416948	-0.213877	3.103339
C	3.263757	3.189178	2.892125
H	4.188349	2.420383	1.107042
C	6.628074	-2.072889	-1.748405
H	7.439823	0.213500	-0.308201
C	2.374181	2.939126	3.937179
H	1.028406	1.503250	4.829309
H	3.799601	4.139993	2.843193
C	6.698430	-3.475463	-1.161827
C	6.021148	-2.039865	-3.143989
H	7.645761	-1.652449	-1.792464
H	2.208852	3.695004	4.708627
H	7.311426	-4.119879	-1.807951
H	7.152404	-3.462672	-0.160353
H	5.692998	-3.914134	-1.087833

H	4.996493	-2.438747	-3.126538
H	6.621215	-2.657521	-3.827214
H	5.997291	-1.014130	-3.540589
N	-2.020384	-0.917973	0.714148
C	-2.247348	-1.046029	-0.601972
C	-3.014901	-0.910761	1.612738
C	-3.554933	-1.218679	-1.031356
C	-0.960818	-0.984785	-1.338917
N	-4.294177	-1.036888	1.301306
H	-2.726232	-0.797016	2.660545
C	-4.530488	-1.186139	0.005254
N	-4.149236	-1.372924	-2.251055
C	-0.649982	-2.011301	-2.277730
C	-0.121279	0.186894	-1.285410
N	-5.729298	-1.331690	-0.636236
C	-5.424924	-1.438001	-1.971096
C	0.467688	-1.924951	-3.067786
H	-1.325523	-2.864412	-2.354709
C	1.023820	0.236558	-2.138075
C	-7.065660	-1.402954	-0.033376
H	-6.218743	-1.566873	-2.709546
C	1.320607	-0.797471	-2.989065
H	0.695312	-2.726291	-3.773841
H	1.660782	1.123516	-2.109276
C	-7.175940	-2.622317	0.870419
C	-7.404208	-0.101057	0.676989
H	-7.747906	-1.530496	-0.889313
H	2.205923	-0.741461	-3.625336
H	-8.203110	-2.710292	1.251709
H	-6.933522	-3.544912	0.323273
H	-6.495870	-2.530953	1.729402
H	-6.717945	0.070730	1.518516
H	-8.429721	-0.148888	1.069599
H	-7.338301	0.752803	-0.011827
P	-0.580975	1.648181	-0.319688
O	-0.240192	1.273540	1.135332
C	0.343128	3.091395	-0.852296
C	1.107897	3.746786	0.116916
C	0.286028	3.577291	-2.165999
C	1.825055	4.889172	-0.233717
H	1.137149	3.355203	1.135272
C	1.010449	4.714082	-2.506602
H	-0.317345	3.072360	-2.924561
C	1.778725	5.369293	-1.540549

H	2.423809	5.402221	0.521975
H	0.974542	5.095748	-3.529162
H	2.342914	6.264220	-1.812999
C	-2.335158	1.979003	-0.571328
C	-3.115699	2.302051	0.543504
C	-2.908333	1.921732	-1.847415
C	-4.471623	2.573957	0.376029
H	-2.655837	2.326752	1.534540
C	-4.262355	2.199447	-2.007026
H	-2.311273	1.627736	-2.714740
C	-5.041084	2.527553	-0.896518
H	-5.086604	2.823189	1.243450
H	-4.712334	2.149694	-3.000605
H	-6.102388	2.752686	-1.025538

I-5^III^

Lowest frequency = 15.0030 cm^{-1}

Charge = 0, Multiplicity = 2

95

Ru	0.197771	-0.234424	0.766573
O	-0.129269	0.824985	2.663644
C	0.056370	-0.218190	3.372358
O	0.342742	-1.300951	2.815929
C	-0.102127	-0.122027	4.861173
H	0.247603	-1.038834	5.349711
H	0.445893	0.751533	5.240680
H	-1.166224	0.032688	5.096315
N	2.311835	-0.287560	0.774182
C	3.168038	0.635266	0.274511
C	2.751411	-1.541607	1.062905
C	4.452892	0.183236	-0.064369
C	2.818252	2.055489	0.230151
N	3.952950	-2.026093	0.832765
H	2.011890	-2.193722	1.530143
C	4.763661	-1.157852	0.236056
N	5.545398	0.810685	-0.610729
C	2.139146	2.623591	1.314297
C	3.259627	2.870873	-0.822511
N	6.060442	-1.334729	-0.156989
C	6.459212	-0.116996	-0.658365
C	1.914039	3.997530	1.351499

H	1.787794	1.990785	2.130761
C	3.014013	4.235204	-0.784725
H	3.769294	2.418169	-1.671177
C	6.856130	-2.557683	-0.068989
H	7.468694	0.015509	-1.052391
C	2.348438	4.803432	0.304525
H	1.381902	4.434102	2.198771
H	3.336147	4.865512	-1.616527
C	7.061978	-2.958827	1.385360
C	6.225337	-3.663192	-0.904858
H	7.831502	-2.290189	-0.507888
H	2.155211	5.878276	0.326222
H	7.714411	-3.842179	1.442949
H	7.531603	-2.144238	1.955949
H	6.097988	-3.204967	1.853260
H	5.237466	-3.930735	-0.502611
H	6.863227	-4.558739	-0.887231
H	6.103712	-3.345190	-1.950675
N	-1.849839	-0.575934	0.787888
C	-2.242874	-1.695170	0.121851
C	-2.746266	0.206745	1.418082
C	-3.617998	-1.972158	0.115216
C	-1.191618	-2.398961	-0.570947
N	-4.053014	0.030347	1.441391
H	-2.326650	1.051684	1.965894
C	-4.452657	-1.048703	0.774772
N	-4.370564	-2.962968	-0.463106
C	-1.420648	-3.538859	-1.357331
C	0.082235	-1.779960	-0.498456
N	-5.729843	-1.500553	0.575645
C	-5.602265	-2.645099	-0.172339
C	-0.383344	-4.057344	-2.118320
H	-2.419981	-3.976764	-1.381370
C	1.095993	-2.300461	-1.318550
C	-6.972251	-0.876661	1.024638
H	-6.484805	-3.210999	-0.476088
C	0.862987	-3.420376	-2.112519
H	-0.547187	-4.940001	-2.740363
H	2.057395	-1.791116	-1.391450
C	-7.022044	-0.812862	2.544870
C	-7.149314	0.485943	0.367948
H	-7.769825	-1.550060	0.669682
H	1.667335	-3.799512	-2.749204
H	-7.987560	-0.400938	2.872532

H	-6.905798	-1.814253	2.984534
H	-6.218027	-0.167078	2.925783
H	-6.345517	1.167998	0.680741
H	-8.113911	0.924788	0.661568
H	-7.128058	0.398996	-0.728255
P	0.008694	1.004494	-1.171050
C	-1.520781	0.519122	-2.079644
C	-2.795258	0.999372	-1.757842
C	-1.384979	-0.420255	-3.106297
C	-3.920516	0.510686	-2.419007
H	-2.911551	1.765259	-0.987095
C	-2.510111	-0.908818	-3.767202
H	-0.382052	-0.767110	-3.366554
C	-3.781051	-0.454480	-3.416830
H	-4.911165	0.891385	-2.156226
H	-2.393716	-1.656056	-4.556047
H	-4.663610	-0.845618	-3.929209
C	-0.386692	2.769536	-0.847121
C	-0.112280	3.674827	-1.877300
C	-1.005503	3.223182	0.320116
C	-0.484370	5.011251	-1.752435
H	0.399431	3.308897	-2.771342
C	-1.383591	4.559770	0.442346
H	-1.159535	2.534102	1.152444
C	-1.128833	5.453865	-0.596575
H	-0.269820	5.714030	-2.561812
H	-1.868436	4.906615	1.358563
H	-1.424947	6.501839	-0.502133
O	1.200486	0.873056	-2.109889

TS(5-6)^{III}

Lowest frequency = -218.7340 cm^{-1}

Charge = 0, Multiplicity = 2

95

Ru	-0.133946	0.861876	-0.595572
O	0.024512	3.028834	-1.361911
C	-0.056321	2.556861	-2.522273
O	-0.209180	1.306626	-2.673464
C	0.067553	3.435000	-3.731584
H	-0.263036	4.454913	-3.499236
H	1.127227	3.474091	-4.028294

H	-0.504547	3.019929	-4.570931
N	-2.231007	0.945780	-0.553539
C	-2.931668	1.257655	0.557779
C	-2.866880	0.456246	-1.643662
C	-4.301439	0.957431	0.554864
C	-2.279801	1.944291	1.682381
N	-4.150315	0.166388	-1.743161
H	-2.224879	0.283331	-2.509987
C	-4.830930	0.404344	-0.627782
N	-5.292077	1.104709	1.494788
C	-1.350575	2.964661	1.433829
C	-2.622092	1.625416	3.004011
N	-6.163622	0.209663	-0.379761
C	-6.363109	0.647743	0.907424
C	-0.769134	3.652878	2.497947
H	-1.100895	3.239456	0.406839
C	-2.028473	2.309280	4.059133
H	-3.338354	0.826898	3.191773
C	-7.163681	-0.346931	-1.287278
H	-7.355087	0.599309	1.360858
C	-1.103407	3.326061	3.810258
H	-0.051781	4.450771	2.292710
H	-2.286787	2.044664	5.086980
C	-7.337395	0.548693	-2.506506
C	-6.810679	-1.782072	-1.654162
H	-8.101471	-0.347031	-0.707366
H	-0.645737	3.864237	4.644297
H	-8.135558	0.155153	-3.152647
H	-7.606938	1.572296	-2.207739
H	-6.404311	0.586991	-3.086836
H	-5.864512	-1.810781	-2.213632
H	-7.601727	-2.215950	-2.282962
H	-6.703714	-2.403687	-0.753198
N	1.887590	0.718034	-0.690454
C	2.368916	-0.471826	-1.161954
C	2.720761	1.722182	-0.353153
C	3.770868	-0.569085	-1.293506
C	1.374490	-1.459825	-1.437096
N	4.036876	1.700979	-0.421945
H	2.229669	2.631435	0.003273
C	4.520660	0.547786	-0.890583
N	4.602200	-1.587257	-1.683485
C	1.613522	-2.561599	-2.282936
C	0.043395	-1.178619	-0.910322

N	5.834395	0.183559	-1.050757
C	5.807477	-1.101712	-1.527161
C	0.570834	-3.373230	-2.683675
H	2.632484	-2.733194	-2.637272
C	-1.003348	-2.050083	-1.389198
C	7.014884	0.979456	-0.729029
H	6.734070	-1.636185	-1.743252
C	-0.745151	-3.092614	-2.244974
H	0.756439	-4.217498	-3.350511
H	-2.015168	-1.903385	-1.004710
C	7.051750	2.248698	-1.569170
C	7.072724	1.260435	0.767268
H	7.871246	0.342617	-1.006455
H	-1.570249	-3.727031	-2.579507
H	7.974647	2.810554	-1.363993
H	7.022815	2.009591	-2.642303
H	6.189902	2.887657	-1.329034
H	6.202789	1.858887	1.074586
H	7.988445	1.817564	1.013493
H	7.073913	0.322598	1.342383
P	-0.118568	-1.330872	1.056072
C	0.110563	-3.133630	1.279345
C	1.172553	-3.893387	0.776416
C	-0.902733	-3.763815	2.010771
C	1.222584	-5.265021	1.013459
H	1.952699	-3.428614	0.172927
C	-0.848110	-5.136112	2.244934
H	-1.727519	-3.151428	2.383272
C	0.214790	-5.888716	1.748164
H	2.052928	-5.851177	0.612476
H	-1.642035	-5.620250	2.819257
H	0.256985	-6.965522	1.930114
C	1.276244	-0.559760	1.938033
C	0.971834	0.572913	2.703228
C	2.602134	-1.005980	1.862002
C	1.987042	1.275495	3.347195
H	-0.068464	0.889360	2.792599
C	3.613849	-0.292501	2.499868
H	2.855241	-1.908219	1.305986
C	3.311204	0.852989	3.235851
H	1.734818	2.158655	3.938353
H	4.647586	-0.638016	2.422591
H	4.109485	1.411460	3.730655
O	-1.464203	-0.951319	1.632603

I-6[I]

Lowest frequency = 12.9615 cm^{-1}

Charge = 0, Multiplicity = 2

95

Ru	0.269156	-0.549960	-0.114819
O	-0.470508	-2.949014	-2.057053
C	-0.015783	-1.985820	-2.665984
O	0.492646	-0.933329	-2.114973
C	-0.013960	-1.936977	-4.179543
H	1.000041	-1.724699	-4.547958
H	-0.378638	-2.883602	-4.596059
H	-0.655846	-1.108121	-4.515534
N	2.363276	-0.370619	-0.167972
C	3.216852	-1.275957	0.356540
C	2.835115	0.720534	-0.811666
C	4.588052	-0.984681	0.271890
C	2.692967	-2.505087	0.967614
N	4.100333	1.054021	-0.968954
H	2.076539	1.376342	-1.236692
C	4.943916	0.200417	-0.402841
N	5.714924	-1.640966	0.704241
C	1.558738	-3.138588	0.434798
C	3.319190	-3.054675	2.096684
N	6.312157	0.249413	-0.364588
C	6.700158	-0.879134	0.314337
C	1.039593	-4.275570	1.047913
H	1.082386	-2.796360	-0.491724
C	2.787892	-4.186355	2.709060
H	4.220381	-2.584596	2.491795
C	7.177402	1.287282	-0.921069
H	7.757942	-1.083273	0.491113
C	1.642549	-4.794798	2.191913
H	0.162791	-4.755820	0.607513
H	3.272862	-4.596830	3.597807
C	7.033012	1.347403	-2.435729
C	6.904346	2.625351	-0.247486
H	8.201560	0.963256	-0.672151
H	1.230786	-5.684428	2.675018
H	7.733840	2.085997	-2.851323
H	7.249342	0.370032	-2.891403

H	6.009928	1.642988	-2.709083
H	5.875881	2.954708	-0.454441
H	7.599858	3.387254	-0.628491
H	7.034227	2.551670	0.842109
N	-1.734909	-0.766852	-0.421571
C	-2.399431	0.251360	-0.996192
C	-2.386385	-1.874912	-0.005074
C	-3.791561	0.126793	-1.099452
C	-1.643086	1.384935	-1.570887
N	-3.685575	-2.084683	-0.079069
H	-1.751457	-2.650424	0.425390
C	-4.355920	-1.070368	-0.611464
N	-4.773591	0.962147	-1.570603
C	-1.949253	1.692102	-2.904980
C	-0.603462	2.097147	-0.923674
N	-5.706879	-0.940429	-0.803514
C	-5.879949	0.295182	-1.376131
C	-1.218466	2.635657	-3.617238
H	-2.762120	1.151931	-3.392500
C	0.119840	3.048737	-1.661697
C	-6.747026	-1.907684	-0.458691
H	-6.879716	0.653175	-1.629320
C	-0.167251	3.306115	-2.997194
H	-1.466170	2.837335	-4.661682
H	0.920016	3.588346	-1.147453
C	-6.563461	-3.193946	-1.252361
C	-6.779336	-2.140112	1.045960
H	-7.690875	-1.428153	-0.766722
H	0.422606	4.041883	-3.548664
H	-7.384541	-3.891685	-1.032740
H	-6.560298	-2.992692	-2.333547
H	-5.612789	-3.676716	-0.983919
H	-5.836017	-2.594497	1.381711
H	-7.605278	-2.818535	1.304367
H	-6.926153	-1.194230	1.587827
P	-0.133843	2.156619	0.856779
C	-1.286878	3.403547	1.528277
C	-2.597117	3.572389	1.065948
C	-0.808043	4.205672	2.569559
C	-3.426836	4.522918	1.657972
H	-2.974964	2.975681	0.230557
C	-1.640453	5.155334	3.157470
H	0.227732	4.080695	2.895435
C	-2.950765	5.311546	2.705192

H	-4.447974	4.653864	1.291830
H	-1.263104	5.782220	3.969193
H	-3.602060	6.058901	3.165229
C	-0.526527	0.578542	1.618955
C	0.556010	-0.266832	2.048105
C	-1.848201	0.320257	2.131367
C	0.236662	-1.394127	2.874027
H	1.566749	0.145948	2.102044
C	-2.103109	-0.784995	2.894016
H	-2.653210	1.027391	1.919006
C	-1.049721	-1.674104	3.248363
H	1.055063	-2.024796	3.227701
H	-3.114448	-0.966212	3.266538
H	-1.260955	-2.547920	3.868986
O	1.297968	2.595056	1.031423

Acetate

Lowest frequency = 40.4371 cm^{-1}

Charge = -1, Multiplicity = 1

7

C	0.278887	-1.070415	0.000011
O	1.503675	-1.297973	0.000004
O	-0.331533	0.016875	-0.000015
C	-0.650467	-2.333331	0.000066
H	-1.312426	-2.307191	0.883529
H	-1.312534	-2.307188	-0.883315
H	-0.074836	-3.273163	0.000034

Acetic Acid

Lowest frequency = 79.7380 cm^{-1}

Charge = 0, Multiplicity = 1

8

C	0.297073	-1.163279	0.000013
O	1.496903	-1.233142	-0.000014
O	-0.355861	0.010101	0.000001
H	0.326101	0.699363	-0.000032
C	-0.650394	-2.324843	0.000066
H	-1.301893	-2.274652	0.884698

H	-1.301985	-2.274662	-0.884498
H	-0.083974	-3.262061	0.000045

7. References

1 (a) S. Shi, Z. Zheng, Y. Zhang, Y. Yang, D. Ma, Y. Gao, Y. Liu, G. Tang and Y. Zhao, Photoinduced Phosphorylation/Cyclization of Cyanoaromatics for Divergent Access to Mono- and Diphosphorylated Polyheterocycles. Org. Lett., 2021, **23**, 9348-9352; (b) Y. Yin, W.-Z. Weng, J.-G. Sun and B. Zhang, Eosin Y-catalyzed, visible-light-promoted carbophosphinylation of allylic alcohols via a radical neophyl rearrangement. Org. Biomol. Chem., 2018, **16**, 2356-2361.

2 M. J. Frisch, G. W. Trucks, H. B. Schlegel, G. E. Scuseria, M. A. Robb, J. R. Cheeseman, G. Scalmani, V. Barone, G. A. Petersson, H. Nakatsuji, X. Li, M. Caricato, A. V. Marenich, J. Bloino, B. G. Janesko, R. Gomperts, B. Mennucci, H. P. Hratchian, J. V. Ortiz, A. F. Izmaylov, J. L. Sonnenberg, Williams, F. Ding, F. Lipparini, F. Egidi, J. Goings, B. Peng, A. Petrone, T. Henderson, D. Ranasinghe, V. G. Zakrzewski, J. Gao, N. Rega, G. Zheng, W. Liang, M. Hada, M. Ehara, K. Toyota, R. Fukuda, J. Hasegawa, M. Ishida, T. Nakajima, Y. Honda, O. Kitao, H. Nakai, T. Vreven, K. Throssell, J. A. Montgomery Jr., J. E. Peralta, F. Ogliaro, M. J. Bearpark, J. J. Heyd, E. N. Brothers, K. N. Kudin, V. N. Staroverov, T. A. Keith, R. Kobayashi, J. Normand, K. Raghavachari, A. P. Rendell, J. C. Burant, S. S. Iyengar, J. Tomasi, M. Cossi, J. M. Millam, M. Klene, C. Adamo, R. Cammi, J. W. Ochterski, R. L. Martin, K. Morokuma, O. Farkas, J. B. Foresman and D. J. Fox, *Gaussian 16 Rev. A.03, Wallingford, CT, 2016.*

3 M. Ernzerhof and G. E. Scuseria, Assessment of the Perdew–Burke–Ernzerhof exchange-correlation functional, *J. Chem. Phys.*, 1999, **110**, 5029-5036.

4 C. Adamo and V. Barone, Toward reliable density functional methods without adjustable parameters: The PBE0 model, *J. Chem. Phys.*, 1999, **110**, 6158-6170.

5 S. Grimme, S. Ehrlich and L. Goerigk, Effect of the damping function in dispersion corrected density functional theory, *J. Comput. Chem.*, 2011, **32**, 1456-1465.

6 S. Grimme, J. Antony, S. Ehrlich and H. Krieg, A consistent and accurate ab initio parametrization of density functional dispersion correction (DFT-D) for the 94 elements H-Pu, *J. Chem. Phys.*, 2010, **132**, 154104.

7 F. Weigend, Accurate Coulomb-fitting basis sets for H to Rn, *Phys. Chem. Chem. Phys.*, 2006, **8**, 1057-1065.

8 F. Weigend and R. Ahlrichs, Balanced basis sets of split valence, triple zeta valence and quadruple zeta valence quality for H to Rn: Design and assessment of accuracy, *Phys. Chem. Chem. Phys.*, 2005, **7**, 3297-3305.

9 J. M. L. Martin and A. Sundermann, Correlation consistent valence basis sets for use with the Stuttgart–Dresden–Bonn relativistic effective core potentials: The atoms Ga–Kr and In–Xe, *J. Chem. Phys.*, 2001, **114**, 3408-3420.

10 D. Andrae, U. Häußermann, M. Dolg, H. Stoll and H. Preuß, Energy-adjusted *ab initio* pseudopotentials for the second and third row transition elements, *Theor. Chim. Acta*, 1990, **77**, 123-141.

11 M. Dolg, U. Wedig, H. Stoll and H. Preuss, Energy-adjusted *ab initio* pseudopotentials for the first row transition elements, *J. Chem. Phys.*, 1987, **86**, 866-872.

12 E. Caldeweyher, S. Ehlert, A. Hansen, H. Neugebauer, S. Spicher, C. Bannwarth and S. Grimme, A generally applicable atomic-charge dependent London dispersion correction, *J. Chem. Phys.*, 2019, **150**, 154122.

13 E. Caldeweyher, C. Bannwarth and S. Grimme, Extension of the D3 dispersion coefficient model, *J. Chem. Phys.*, 2017, **147**, 034112.

14 A. V. Marenich, C. J. Cramer and D. G. Truhlar, Universal Solvation Model Based on Solute Electron Density and on a Continuum Model of the Solvent Defined by the Bulk Dielectric Constant and Atomic Surface Tensions, *J. Phys. Chem. B*, 2009, **113**, 6378-6396.
15 A. K. Simlandy, W. Rodphon, T. M. Alturaifi, B. K. Mai, H.-Q. Ni, J. A. Gurak, Jr., P. Liu and K. M. Engle, Catalytic Addition of Nitroalkanes to Unactivated Alkenes via Directed Carbopalladation, *ACS Catal.*, 2022, **12**, 13755-13762.

8. Characterization Data

(2-(9-Isopropyl-9H-purin-6-yl)phenyl)diphenylphosphine oxide (**3a**)

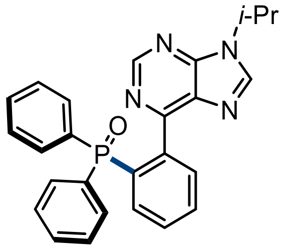

Prepared according to standard procedure A using [Ru(OAc)$_2$(*p*-cymene)] (10 mol %) on a 0.3 mmol scale, purification by column chromatography on silica gel (DCM/MeOH = 20/1, R_f = 0.13) yielded **3a** (95.9 mg, 73%) as a colorless sticky oil.

^{1}H NMR (400 MHz, CDCl$_3$) δ 8.47 (s, 1H), 7.93 (s, 1H), 7.85 – 7.73 (m, 2H), 7.58 – 7.49 (m, 5H), 7.45 – 7.39 (m, 1H), 7.19 – 7.08 (m, 6H), 4.68 (m, 6H), 1.46 (d, *J* = 6.8 Hz, 6H).

^{13}C NMR (101 MHz, CDCl$_3$) δ 156.0 (d, *J* = 3.3 Hz) (C$_q$), 150.7 (C$_q$), 150.5 (CH), 141.9 (CH), 139.4 (d, *J* = 7.1 Hz) (C$_q$), 135.0 (d, *J* = 10.3 Hz) (CH), 133.4 (d, *J* = 106.4 Hz) (C$_q$), 132.1 (d, *J* = 101.0 Hz) (C$_q$), 131.3 (CH), 131.2 (CH), 131.2 (CH), 130.4 (d, *J* = 2.7 Hz) (CH), 128.6 (d, *J* = 12.0 Hz) (CH), 127.3 (d, *J* = 12.3 Hz) (CH), 46.9 (CH), 22.1 (CH$_3$).

^{31}P NMR (162 MHz, CDCl$_3$) δ 28.79.

HRMS (ESI) m/z: [M+H]$^+$ Calcd for $C_{26}H_{24}N_4OP$ 439.1682; Found 439.1686.

IR (ATR): 2977, 1577, 1331, 1178, 931, 719, 535 cm^{-1}.

(2-(9-Methyl-9H-purin-6-yl)phenyl)diphenylphosphine oxide (**3b**)

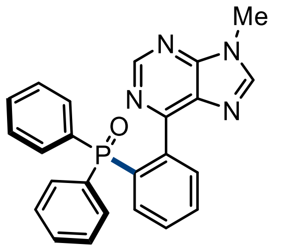

Prepared according to standard procedure A using [Ru(OAc)$_2$(*p*-cymene)] (10 mol %) on a 0.3 mmol scale, purification by column chromatography on silica gel (DCM/MeOH = 20/1, R_f = 0.14) yielded **3b** (68.9 mg, 56%) as a colorless solid. M.p.: 210-211 °C

^{1}H NMR (300 MHz, CDCl$_3$) δ 8.50 (s, 1H), 7.94 – 7.86 (m, 2H), 7.72 (dd, *J* = 13.6, 7.7 Hz, 1H), 7.63 – 7.50 (m, 5H), 7.45 (t, *J* = 7.6 Hz, 1H), 7.26 – 7.14 (m, 6H), 3.71 (s, 3H).

^{13}C NMR (75 MHz, CDCl$_3$) δ 156.2 (d, *J* = 3.1 Hz) (C$_q$), 151.7 (C$_q$), 151.1 (CH), 145.0 (CH), 139.6 (d, *J* = 6.7 Hz) (C$_q$), 135.2 (d, *J* = 10.6 Hz) (CH), 133.7 (d, *J* = 106.9 Hz) (C$_q$), 131.9 (d, *J* = 122.0 Hz) (C$_q$), 131.7 (CH), 131.5 (CH), 131.4 (CH), 130.8 (d, *J* = 2.8 Hz) (CH), 129.0 (d, *J* = 12.1 Hz) (CH), 127.7 (d, *J* = 12.3 Hz) (CH), 29.6 (CH$_3$).

^{31}P NMR (121 MHz, CDCl$_3$) δ 29.61.

HRMS (ESI) m/z: [M+H]$^+$ Calcd for $C_{24}H_{20}N_4OP$ 411.1369; Found 411.1376.

IR (ATR): 3058, 1715, 1581, 1397, 1115, 717, 640, 536 cm^{-1}.

(2-(9-Benzyl-9H-purin-6-yl)phenyl)diphenylphosphine oxide (**3c**)

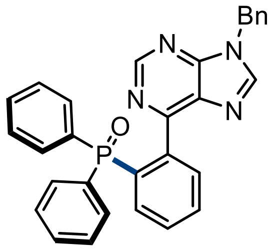

Prepared according to standard procedure A using [Ru(OAc)$_2$(*p*-cymene)] (10 mol %) on a 0.3 mmol scale, purification by column chromatography on silica gel (DCM/MeOH = 20/1, R_f = 0.3) yielded **3c** (103.1 mg, 71%) as a colorless solid. M.p.: 218-219 °C

^{1}H NMR (300 MHz, $CDCl_3$) δ 8.52 (s, 1H), 7.90 (s, 1H), 7.88 (dd, *J* = 7.7, 4.2 Hz, 1H), 7.79 – 7.71 (m, 1H), 7.61 – 7.50 (m, 5H), 7.47 – 7.40 (m, 1H), 7.28 – 7.20 (m, 5H), 7.13 – 7.08 (m, 6H), 5.23 (s, 2H).

^{13}C NMR (75 MHz, $CDCl_3$) δ 156.3 (d, *J* = 3.2 Hz) (C_q), 151.3 (C_q), 151.3 (CH), 144.2 (CH), 139.5 (d, *J* = 6.9 Hz) (C_q), 135.3 (d, *J* = 10.5 Hz) (CH), 135.0 (C_q), 133.5 (d, *J* = 106.8 Hz) (C_q), 131.9 (d, *J* = 139.6 Hz) (C_q), 131.7 (CH), 131.6 (CH), 131.5 (CH), 131.4 (CH), 130.7 (d, *J* = 2.8 Hz) (CH), 129.0 (CH), 128.9 (CH), 128.4 (CH), 127.9 (CH), 127.6 (d, *J* = 12.3 Hz) (CH), 47.0 (CH_2).

^{31}P NMR (121 MHz, $CDCl_3$) δ 29.40.

HRMS (ESI) m/z: $[M+H]^+$ Calcd for $C_{30}H_{24}N_4OP$ 487.1682; Found 487.1678.

IR (ATR): 3060, 1716, 1580, 1437, 1108, 906, 691, 647, 532 cm^{-1}.

(2R,3R,4R,5R)-2-(Acetoxymethyl)-5-(6-(2-(diphenylphosphoryl)phenyl)-9H-purin-9-yl)tetrahydrofuran-3,4-diyl diacetate (**3d**)

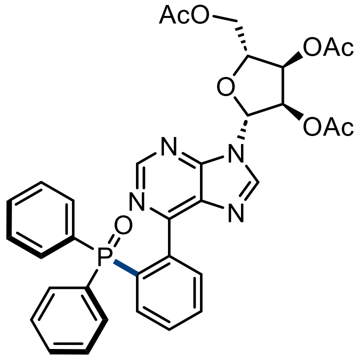

Prepared according to standard procedure A using [Ru(OAc)$_2$(*p*-cymene)] (10 mol %) on a 0.3 mmol scale, purification by column chromatography on silica gel (DCM/MeOH = 20/1, R_f = 0.25) yielded **3d** (119.7 mg, 61%) as a colorless sticky oil.

^{1}H NMR (400 MHz, $CDCl_3$) δ 8.60 (s, 1H), 8.14 (s, 1H), 7.92 – 7.83 (m, 2H), 7.73 – 7.66 (m, 2H), 7.64 – 7.56 (m, 4H), 7.36 – 7.31 (m, 2H), 7.29 – 7.22 (m, 4H), 6.17 (d, *J* = 4.7 Hz, 1H), 5.93 – 5.85 (m, 1H), 5.69 (t, *J* = 5.3 Hz, 1H), 4.51 – 4.35 (m, 3H), 2.16 – 2.11 (m, 9H).

^{13}C NMR (75 MHz, $CDCl_3$) δ 170.2 (C_q), 169.4 (C_q), 169.3 (C_q), 157.2 (d, *J* = 3.2 Hz) (C_q), 151.5 (CH), 150.8 (C_q), 142.7 (CH), 139.2 (d, *J* = 6.9 Hz) (C_q), 135.3 (d, *J* = 10.3 Hz) (CH), 134.3 (C_q), 134.2 (C_q), 133.1 (C_q), 132.9 (C_q), 132.8 (C_q), 131.9 (C_q), 131.8 (C_q), 131.6 (CH), 131.6 (CH), 131.5 (CH), 131.5 (CH), 131.0 (CH), 130.9 (CH), 130.9 (CH), 129.1 (d, *J* = 11.9 Hz) (CH), 127.7 (d, *J* = 12.3 Hz) (CH), 86.5 (CH), 80.0 (CH), 73.1 (CH), 70.3 (CH), 62.9 (CH_2), 20.6 (CH_3), 20.4 (CH_3), 20.3 (CH_3).

^{31}P NMR (121 MHz, $CDCl_3$) δ 29.06.

HRMS (ESI) m/z: $[M+H]^+$ Calcd for$C_{34}H_{32}N_4O_8P$ 655.1952; Found 655.1948.

IR (ATR): 1745, 1582, 1212, 901, 720, 541 m^{-1}.

(4-Fluoro-2-(9-isopropyl-9H-purin-6-yl)phenyl)diphenylphosphine oxide (**3e**)

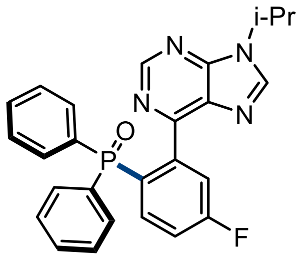

Prepared according to standard procedure A using [Ru(OAc)$_2$(*p*-cymene)] (10 mol %) on a 0.3 mmol scale, purification by column chromatography on silica gel (DCM/MeOH = 20/1, R_f = 0.22) yielded **3e** (112.2 mg, 82%) as a colorless sticky oil.

^{1}H NMR (300 MHz, $CDCl_3$) δ 8.78 (s, 1H), 8.07 (s, 1H), 7.70 – 7.58 (m, 5H), 7.51 – 7.43 (m, 1H), 7.38 – 7.24 (m, 6H), 7.21 – 7.12 (m, 1H), 4.90 – 4.78 (m, 1H), 1.61 (d, J = 6.8 Hz, 6H).

^{13}C NMR (75 MHz, $CDCl_3$) δ 172.8 (C_q), 163.9 (d, $J_{C\text{-}F}$ = 251.6 Hz) (C_q), 156.5 (t, J = 3.1 Hz) (C_q), 151.0 (CH), 150.7 (C_q), 142.4 (C_q), 142.2 (CH), 133.9 (CH), 133.7 (d, J = 1.8 Hz) (CH), 132.2 (d, J = 27.9 Hz) (C_q), 131.1 (d, J = 3.3 Hz) (CH), 131.0 (d, J = 1.8 Hz) (CH), 127.7 (d, J = 12.8 Hz) (CH), 119.9 (dd, J = 97.2, 17.6 Hz) (C_q), 117.0 (dd, J = 24.6, 5.1 Hz) (CH), 47.2 (CH), 22.3 (CH_3).

^{31}P NMR (121 MHz, $CDCl_3$) δ 23.85.

^{19}F NMR (282 MHz, $CDCl_3$) δ -94.79.

HRMS (ESI) m/z: $[M+H]^+$ Calcd for $C_{26}H_{23}FN_4OP$ 457.1588; Found 457.1590.

IR (ATR): 2978, 2227, 1561, 1438, 1195, 1117, 906, 716 cm^{-1}.

(2-(9-Isopropyl-9H-purin-6-yl)-4-methylphenyl)diphenylphosphine oxide (**3f**)

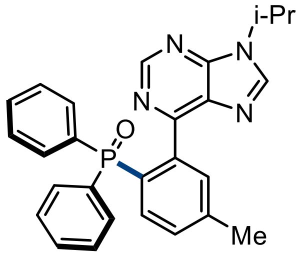

Prepared according to standard procedure A using [Ru(OAc)$_2$(*p*-cymene)] (10 mol %) on a 0.3 mmol scale, purification by column chromatography on silica gel (DCM/MeOH = 20/1, R_f = 0.3) yielded **3f** (85.5 mg, 63%) as a colorless sticky oil.

^{1}H NMR (300 MHz, $CDCl_3$) δ 8.58 (s, 1H), 8.02 (s, 1H), 7.79 – 7.57 (m, 6H), 7.36 (d, J = 7.9 Hz, 1H), 7.30 – 7.18 (m, 6H), 4.90 – 4.74 (m, 1H), 2.46 (s, 3H), 1.59 (d, J = 6.8 Hz, 6H).

^{13}C NMR (75 MHz, $CDCl_3$) δ 156.8 (d, J = 3.3 Hz) (C_q), 150.9 (CH), 150.9 (C_q), 142.1 (d, J = 2.6 Hz) (C_q), 141.9 (CH), 139.6 (d, J = 7.3 Hz) (C_q), 135.3 (d, J = 10.6 Hz) (CH), 133.8 (d, J = 106.4 Hz) (C_q), 132.1 (d, J = 9.9 Hz) (CH), 131.6 (d, J = 9.5 Hz) (CH), 131.6 (C_q), 130.6 (d, J = 2.6 Hz) (CH), 129.7 (d,

J = 12.1 Hz) (CH), 129.0 (d, J = 103.4 Hz) (C_q), 127.5 (d, J = 12.1 Hz) (CH), 47.1 (CH), 22.4 (CH_3), 21.4 (CH_3).
^{31}P NMR (121 MHz, $CDCl_3$) δ 28.76.
HRMS (ESI) m/z: $[M+H]^+$ Calcd for $C_{27}H_{26}N_4OP$ 453.1839; Found 453.1847.
IR (ATR): 3055, 2977, 2225, 1574, 1436, 1192, 907, 704 cm^{-1}.

(2,5-Bis(3-methoxypyridin-2-yl)phenyl)diphenylphosphine oxide (**3g**)

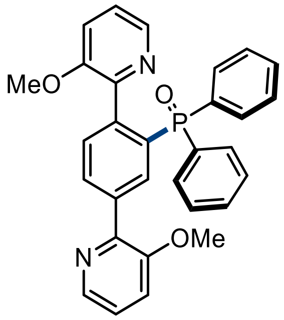

Prepared according to standard procedure A using [Ru(OAc)$_2$(*p*-cymene)] (10 mol %) on a 0.3 mmol scale, purification by column chromatography on silica gel (DCM/MeOH = 20/1, R_f = 0.10) yielded **3g** (91.8 mg, 62%) as a colorless sticky oil.
^{1}H NMR (300 MHz, $CDCl_3$) δ 8.26 – 8.09 (m, 3H), 7.95 (dd, J = 4.7, 1.1 Hz, 1H), 7.60 – 7.52 (m, 4H), 7.47 (dd, J = 8.0, 4.2 Hz, 1H), 7.35 – 7.28 (m, 2H), 7.27 – 7.20 (m, 4H), 7.13 – 7.07 (m, 2H), 6.99 – 6.93 (m, 1H), 6.75 (dd, J = 8.4, 1.1 Hz, 1H), 3.53 (s, 3H), 3.27 (s, 3H).
^{13}C NMR (75 MHz, $CDCl_3$) δ 153.6 (C_q), 153.2 (C_q), 148.6 (d, J = 3.5 Hz) (C_q), 146.2 (C_q), 142.2 (d, J = 8.0 Hz) (C_q), 141.3 (CH), 139.9 (CH), 136.7 (d, J = 12.7 Hz) (C_q), 135.0 (d, J = 12.7 Hz) (CH), 134.1 (d, J = 104.6 Hz) (C_q), 132.2 (d, J = 2.6 Hz) (CH), 131.9 (d, J = 9.8 Hz) (CH), 131.3 (d, J = 10.5 Hz) (CH), 130.8 (d, J = 2.8 Hz) (CH), 130.2 (d, J = 101.9 Hz) (C_q), 127.8 (d, J = 12.1 Hz) (CH), 123.4 (CH), 123.2 (CH), 118.5 (CH), 116.4 (CH), 55.1 (CH_3), 54.2 (CH_3).
^{31}P NMR (121 MHz, $CDCl_3$) δ 29.23.
HRMS (ESI) m/z: $[M+H]^+$ Calcd for $C_{30}H_{26}N_2O_3P$ 493.1676; Found 493.1689.
IR (ATR): 3055, 2834, 1580, 1425, 1179, 1015, 705, 540 cm^{-1}.

(2-(4,4-Dimethyl-4,5-dihydrooxazol-2-yl)phenyl)diphenylphosphine oxide (**3h**)

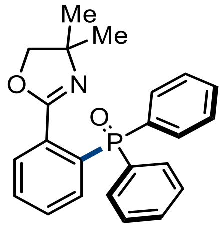

Prepared according to standard procedure A using [Ru(OAc)$_2$(*p*-cymene)] (10 mol %) on a 0.3 mmol scale, purification by column chromatography on silica gel (DCM/MeOH = 20/1, R_f = 0.23) yielded **3h** (39.4 mg, 35%) as a colorless sticky oil.
^{1}H NMR (300 MHz, $CDCl_3$) δ 7.93 (dd, J = 7.5, 4.0 Hz, 1H), 7.72 – 7.64 (m, 4H), 7.58 – 7.51 (m, 1H), 7.49 – 7.40 (m, 8H), 3.55 (s, 2H), 1.09 (s, 6H).

^{13}C NMR (75 MHz, $CDCl_3$) δ 162.4 (d, J = 3.2 Hz) (C_q), 134.8 (d, J = 10.9 Hz) (CH), 133.5 (d, J = 106.8 Hz) (C_q), 132.7 (d, J = 6.2 Hz) (C_q), 131.8 (d, J = 2.4 Hz) (CH), 131.7 (d, J = 99.2 Hz) (C_q), 131.5 (d, J = 9.7 Hz) (CH), 131.3 (d, J = 2.9 Hz) (CH), 131.2 (CH), 130.1 (d, J = 12.0 Hz) (CH), 128.3 (d, J = 12.3 Hz) (CH), 79.1 (CH_2), 67.3 (C_q), 27.8 (CH_3).
^{31}P NMR (121 MHz, $CDCl_3$) δ 29.50.
HRMS (ESI) m/z: $[M+H]^+$ Calcd for $C_{23}H_{23}NO_2P$ 376.1461; Found 376.1472.
IR (ATR): 2964, 1659, 1437, 1177, 963, 693, 543cm^{-1}.

(E)-(2-(1-(Methoxyimino)ethyl)phenyl)diphenylphosphine oxide (3**i**)

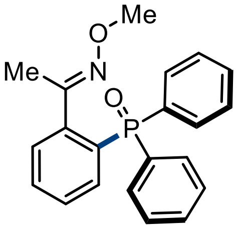

Prepared according to standard procedure A using [Ru(OAc)$_2$(*p*-cymene)] (10 mol %) on a 0.3 mmol scale, purification by column chromatography on silica gel (DCM/MeOH = 20/1, R_f = 0.33) yielded **3i** (52.1 mg, 50%) as a colorless sticky oil.
^{1}H NMR (300 MHz, $CDCl_3$) δ 7.64 – 7.57 (m, 4H), 7.48 – 7.34 (m, 8H), 7.32 – 7.24 (m, 2H), 3.40 (s, 3H), 1.85 (s, 3H).
^{13}C NMR (75 MHz, $CDCl_3$) δ 156.5 (d, J = 3.5 Hz) (C_q), 142.5 (d, J = 7.4 Hz) (C_q), 134.3 (CH), 133.5 (d, J = 95.1 Hz) (C_q), 132.1 (d, J = 9.8 Hz) (CH), 131.9 (d, J = 2.7 Hz) (CH), 131.5 (d, J = 102.5 Hz) (C_q), 131.4 (d, J = 2.7 Hz) (CH), 129.5 (d, J = 9.5 Hz) (CH), 128.2 (d, J = 12.3 Hz) (CH), 127.8 (d, J = 12.3 Hz) (CH), 61.2 (CH_3), 16.6 (CH_3).
^{31}P NMR (121 MHz, $CDCl_3$) δ 30.23.
HRMS (ESI) m/z: $[M+H]^+$ Calcd for $C_{21}H_{21}NO_2P$ 350.1304; Found 350.1314.
IR (ATR): 1436, 1312, 1181, 1037, 875, 692, 529 cm^{-1}.

Diphenyl(2-(pyrazin-2-yl)phenyl)phosphine oxide (**3j**)

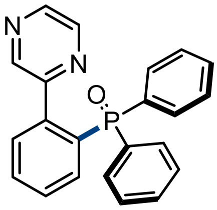

Prepared according to standard procedure A using [Ru(OAc)$_2$(*p*-cymene)] (10 mol %) on a 0.3 mmol scale, purification by column chromatography on silica gel (DCM/MeOH = 20/1, R_f = 0.38) yielded **3j** (29 mg, 27%) as a colorless solid. M.p.: 160-161 °C
^{1}H NMR (300 MHz, $CDCl_3$) δ 8.88 – 8.82 (m, 1H), 8.28 (d, J = 2.4 Hz, 1H), 8.23 – 8.19 (m, 1H), 7.68 – 7.56 (m, 6H), 7.50 – 7.34 (m, 8H).
^{13}C NMR (75 MHz, $CDCl_3$) δ 153.8 (d, J = 4.0 Hz) (C_q), 145.8 (CH), 143.1 (CH), 142.9 (CH), 142.3 (d, J = 7.5 Hz) (C_q), 134.3 (d, J = 11.5 Hz) (CH), 132.9 (d, J = 105.2 Hz) (C_q), 132.1 (d, J = 100.2 Hz) (C_q),

132.0 (d, J = 2.5 Hz) (CH), 131.7 (d, J = 9.6 Hz) (CH), 131.5 (d, J = 2.8 Hz) (CH), 128.5 (CH), 128.4 (CH), 128.3 (d, J = 12.2 Hz) (CH).

^{31}P NMR (121 MHz, $CDCl_3$) δ 28.76.

HRMS (ESI) m/z: $[M+H]^+$ Calcd for $C_{22}H_{18}N_2OP$ 357.1151; Found 357.1146.

IR (ATR): 3053, 1437, 1196, 1104, 1019, 715, 540 cm^{-1}.

Diphenyl(2-(3-phenylpyrazin-2-yl)phenyl)phosphine oxide (**3k**)

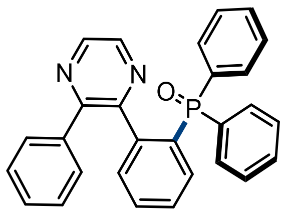

Prepared according to standard procedure A using [Ru(OAc)$_2$(*p*-cymene)] (10 mol %) on a 0.3 mmol scale, purification by column chromatography on silica gel (DCM/MeOH = 20/1, R_f = 0.22) yielded **3k** (44.1 mg, 34%) as a colorless sticky oil.

^{1}H NMR (400 MHz, $CDCl_3$) δ 8.51 (d, J = 2.4 Hz, 1H), 8.20 (d, J = 2.4 Hz, 1H), 7.53 – 7.41 (m, 10H), 7.40 – 7.33 (m, 5H), 7.25 – 7.16 (m, 4H).

^{13}C NMR (101 MHz, $CDCl_3$) δ 153.0 (d, J = 3.4 Hz) (C_q), 152.7 (C_q), 144.1 (d, J = 6.6 Hz) (C_q), 142.6 (CH), 140.8 (CH), 138.1 (C_q), 134.2 (d, J = 11.4 Hz) (CH), 133.6 (d, J = 105.1 Hz) (C_q), 131.8 (d, J = 9.8 Hz) (CH), 131.7 (CH), 131.4 (d, J = 2.7 Hz) (CH), 131.2 (d, J = 2.5 Hz) (CH), 130.4 (d, J = 92.9 Hz) (C_q), 129.9 (CH), 128.3 (CH), 128.2 (d, J = 12.2 Hz) (CH), 127.8 (CH), 127.6 (d, J = 12.2 Hz) (CH).

^{31}P NMR (162 MHz, $CDCl_3$) δ 29.83.

HRMS (ESI) m/z: $[M+H]^+$ Calcd for $C_{28}H_{22}N_2OP$ 433.1464; Found 433.1460.

IR (ATR): 3055, 1389, 1118, 1018, 856, 694, 542 cm^{-1}.

Diphenyl(2-(pyridin-2-yl)phenyl)phosphine oxide (**3l**)

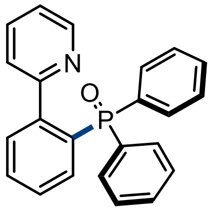

Prepared according to standard procedure A using [Ru(OAc)$_2$(*p*-cymene)] (10 mol %) on a 0.3 mmol scale, purification by column chromatography on silica gel (DCM/MeOH = 20/1, R_f = 0.40) yielded **3l** (54.2 mg, 51%) as a colorless solid. M.p.: 176-177 °C

^{1}H NMR (300 MHz, $CDCl_3$) δ 8.18 – 8.15 (m, 1H), 7.73 (d, J = 7.9 Hz, 1H), 7.56 – 7.49 (m, 6H), 7.41 – 7.35 (m, 2H), 7.33 – 7.27 (m, 3H), 7.26 – 7.19 (m, 4H), 6.88 – 6.83 (m, 1H).

^{13}C NMR (75 MHz, $CDCl_3$) δ 157.5 (d, J = 4.1 Hz) (C_q), 148.6 (CH), 145.7 (d, J = 7.9 Hz) (C_q), 135.3 (CH), 134.3 (d, J = 11.8 Hz) (CH), 133.1 (d, J = 105.2 Hz) (C_q), 131.9 (d, J = 2.6 Hz) (CH), 131.5 (d, J = 9.5 Hz) (CH), 131.3 (CH), 131.1 (d, J = 2.8 Hz) (CH), 131.1 (d, J = 101.3 Hz) (C_q), 128.0 (d, J = 12.2 Hz) (CH), 127.6 (d, J = 12.3 Hz) (CH), 125.6 (CH), 121.9 (CH).

^{31}P NMR (121 MHz, $CDCl_3$) δ 29.12.
HRMS (ESI) m/z: $[M+H]^+$ Calcd for $C_{23}H_{19}NOP$ 356.1199; Found 356.1204.
IR (ATR): 3057, 2142, 1584, 1437, 1181, 1104, 694, 540 cm^{-1}.

Diphenyl(2-(pyridin-2-yl)thiophen-3-yl)phosphine oxide (**3m**)

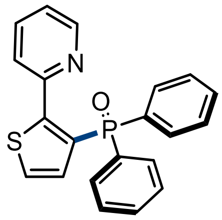

Prepared according to standard procedure A using [Ru(OAc)$_2$(*p*-cymene)] (10 mol %) on a 0.3 mmol scale, purification by column chromatography on silica gel (DCM/MeOH = 20/1, R_f = 0.25) yielded **3m** (96.4 mg, 89%) as a colorless sticky oil.
^{1}H NMR (300 MHz, $CDCl_3$) δ 8.41 – 8.36 (m, 1H), 8.26 – 8.21 (m, 1H), 7.71 – 7.63 (m, 4H), 7.43 – 7.30 (m, 8H), 7.01 – 6.94 (m, 1H), 6.72 (td, *J* = 5.2, 1.1 Hz, 1H).
^{13}C NMR (75 MHz, $CDCl_3$) δ 154.0 (d, *J* = 11.0 Hz) (C_q), 150.9 (d, *J* = 2.1 Hz) (C_q), 148.8 (CH), 136.2 (CH), 134.0 (d, *J* = 16.6 Hz) (CH), 133.0 (d, *J* = 107.4 Hz) (C_q), 131.6 (d, *J* = 2.9 Hz) (CH), 131.5 (d, *J* = 9.9 Hz) (CH), 129.4 (d, *J* = 103.0 Hz) (C_q), 128.3 (d, *J* = 12.4 Hz) (CH), 126.5 (d, *J* = 16.4 Hz) (CH), 124.2 (CH), 122.6 (CH).
^{31}P NMR (121 MHz, $CDCl_3$) δ 22.02.
HRMS (ESI) m/z: $[M+H]^+$ Calcd for $C_{21}H_{17}NOPS$ 362.0763; Found 362.0770.
IR (ATR): 3050, 1582, 1436, 1162, 996, 696, 537 cm^{-1}.

(2-(1-methyl-1H-benzo[d]imidazol-2-yl)phenyl)diphenylphosphine oxide (**3n**)

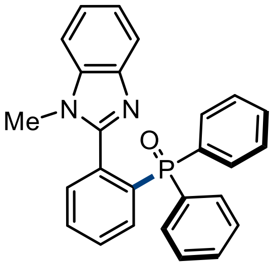

Prepared according to standard procedure A using [Ru(OAc)$_2$(*p*-cymene)] (10 mol %) on a 0.3 mmol scale, purification by column chromatography on silica gel (DCM/MeOH = 20/1, R_f = 0.34) yielded **3n** (50.3 mg, 41%) as a colorless sticky oil.
^{1}H NMR (300 MHz, $CDCl_3$) δ 7.99 – 7.89 (m, 1H), 7.64 – 7.56 (m, 2H), 7.54 – 7.46 (m, 4H), 7.43 – 7.38 (m, 2H), 7.23 – 7.07 (m, 8H), 7.01 – 6.96 (m, 1H), 3.25 (s, 3H).
^{13}C NMR (75 MHz, $CDCl_3$) δ 151.2 (d, *J* = 4.1 Hz) (C_q), 141.9 (C_q), 134.8 (C_q), 134.3 (d, *J* = 9.5 Hz) (CH), 134.0 (d, *J* = 100.0 Hz) (C_q), 133.5 (d, *J* = 8.2 Hz) (C_q), 131.8 (CH), 131.7 (d, *J* = 10.1 Hz) (CH), 131.5 (CH), 131.4 (d, *J* = 106.0 Hz) (C_q), 131.3 (d, *J* = 3.0 Hz) (CH), 129.9 (d, *J* = 11.2 Hz) (CH), 127.8 (d, *J* = 12.5 Hz) (CH), 122.7 (CH), 122.1 (CH), 119.6 (CH), 109.4 (CH), 30.9 (CH_3).
^{31}P NMR (121 MHz, $CDCl_3$) δ 28.70.

HRMS (ESI) m/z: $[M+H]^+$ Calcd for $C_{26}H_{22}N_2OP$ 409.1464; Found 409.1460.
IR (ATR): 3057, 1716, 1422, 1118, 906, 689, 535, 449 cm^{-1}.

(2-(9-Isopropyl-9H-purin-6-yl)phenyl)di(naphthalen-2-yl)phosphine oxide (**3o**)

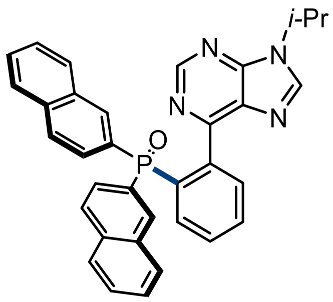

Prepared according to standard procedure A using $[Ru(OAc)_2$(*p*-cymene)] (10 mol %) on a 0.3 mmol scale, purification by column chromatography on silica gel (DCM/MeOH = 20/1, $R_f = 0.18$) yielded **3o** (116.0 mg, 72%) as a colorless sticky oil.
^{1}H NMR (300 MHz, $CDCl_3$) δ 8.53 (s, 1H), 8.28 (d, *J* = 13.9 Hz, 2H), 7.97 – 7.89 (m, 2H), 7.84 (s, 1H), 7.76 – 7.63 (m, 9H), 7.57 – 7.51 (m, 1H), 7.49 – 7.38 (m, 4H), 4.44 (h, *J* = 6.7 Hz, 1H), 1.32 (d, *J* = 6.8 Hz, 6H).
^{13}C NMR (75 MHz, $CDCl_3$) δ 156.3 (d, *J* = 3.2 Hz) (C_q), 150.9 (CH), 150.7 (C_q), 141.9 (CH), 139.8 (d, *J* = 7.2 Hz) (C_q), 135.5 (d, *J* = 10.6 Hz) (CH), 134.0 (d, *J* = 2.3 Hz) (C_q), 133.2 (d, *J* = 9.2 Hz) (CH), 132.3 (d, *J* = 101.4 Hz) (C_q), 132.0 (C_q), 131.9 (C_q), 131.7 (CH), 131.5 (CH), 130.9 (d, *J* = 106.6 Hz) (C_q), 129.0 (d, *J* = 12.0 Hz) (CH), 128.7 (CH), 127.5 (d, *J* = 17.2 Hz) (CH), 127.3 (d, *J* = 12.1 Hz) (CH), 126.7 (d, *J* = 10.5 Hz) (CH), 126.3 (CH), 47.0 (CH), 22.0 (CH_3).
^{31}P NMR (121 MHz, $CDCl_3$) δ 28.59.
HRMS (ESI) m/z: $[M+H]^+$ Calcd for $C_{34}H_{28}N_4OP$ 539.1995; Found 539.1989.
IR (ATR): 3054, 1576, 1391, 1188, 1085, 931, 814, 645, 477 cm^{-1}.

(2-(9-Isopropyl-9H-purin-6-yl)phenyl)di-p-tolylphosphine oxide (**3p**)

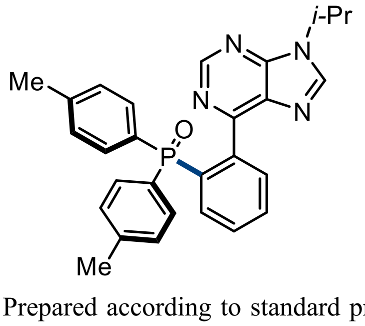

Prepared according to standard procedure A using $[Ru(OAc)_2$(*p*-cymene)] (10 mol %) on a 0.3 mmol scale, purification by column chromatography on silica gel (DCM/MeOH = 20/1, $R_f = 0.15$) yielded **3p** (102.1 mg, 73%) as a colorless sticky oil.
^{1}H NMR (400 MHz, $CDCl_3$) δ 8.56 (s, 1H), 8.03 (s, 1H), 7.88 (dd, *J* = 7.3, 3.9 Hz, 1H), 7.77 (dd, *J* = 13.5, 7.7 Hz, 1H), 7.64 (t, *J* = 7.5 Hz, 1H), 7.51 – 7.45 (m, 5H), 7.06 – 7.01 (m, 4H), 4.85 – 4.75 (m, 1H), 2.26 (s, 6H), 1.58 (d, *J* = 6.8 Hz, 6H).
^{13}C NMR (101 MHz, $CDCl_3$) δ 156.7 (d, *J* = 3.2 Hz) (C_q), 151.0 (CH), 151.0 (C_q), 141.9 (CH), 141.0 (d,

J = 2.8 Hz) (C_q), 139.7 (d, J = 6.9 Hz) (C_q), 135.2 (d, J = 10.6 Hz) (CH), 132.7 (d, J = 101.0 Hz) (C_q), 131.7 (C_q), 131.6 (d, J = 4.8 Hz) (CH), 131.5 (d, J = 3.7 Hz) (CH), 131.3 (d, J = 2.5 Hz) (CH), 130.6 (d, J = 108.9 Hz) (C_q), 128.8 (d, J = 12.0 Hz) (CH), 128.4 (d, J = 12.7 Hz) (CH), 47.1 (CH), 22.3 (CH_3), 21.4 (CH_3), 21.4 (CH_3).
^{31}P NMR (162 MHz, $CDCl_3$) δ 29.37.
HRMS (ESI) m/z: $[M+H]^+$ Calcd for $C_{28}H_{28}N_4OP$ 467.1995; Found 467.2006.
IR (ATR): 2978, 1580, 1331, 1100, 931, 733, 536 cm^{-1}.

Bis(4-chlorophenyl)(2-(9-isopropyl-9H-purin-6-yl)phenyl)phosphine oxide (**3q**)

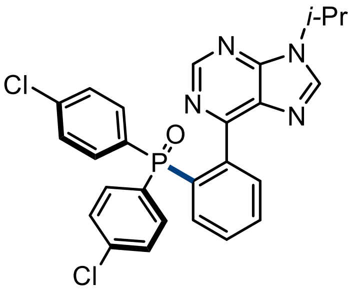

Prepared according to standard procedure A using [Ru(OAc)$_2$(p-cymene)] (10 mol %) on a 0.3 mmol scale, purification by column chromatography on silica gel (DCM/MeOH = 20/1, R_f = 0.38) yielded **3q** (77.4 mg, 51%) as a colorless solid. M.p.: 198-199 °C
^{1}H NMR (300 MHz, $CDCl_3$) δ 8.50 (s, 1H), 7.99 (s, 1H), 7.91 – 7.78 (m, 2H), 7.68 – 7.62 (m, 1H), 7.55 – 7.44 (m, 5H), 7.17 – 7.11 (m, 4H), 4.86 – 4.69 (m, 1H), 1.56 (d, J = 6.8 Hz, 6H).
^{13}C NMR (75 MHz, $CDCl_3$) δ 156.1 (d, J = 3.3 Hz) (C_q), 151.1 (C_q), 150.8 (CH), 142.2 (CH), 139.5 (d, J = 7.4 Hz) (C_q), 137.5 (d, J = 3.4 Hz) (C_q), 135.3 (d, J = 10.3 Hz) (CH), 133.0 (d, J = 10.8 Hz) (CH), 132.0 (d, J = 2.6 Hz) (CH), 132.0 (d, J = 108.3 Hz) (C_q), 131.8 (d, J = 9.4 Hz) (CH), 131.5 (C_q), 131.4 (d, J = 103.2 Hz) (C_q), 129.2 (d, J = 12.2 Hz) (CH), 128.0 (d, J = 13.0 Hz) (CH), 47.4 (CH), 22.3 (CH_3).
^{31}P NMR (121 MHz, $CDCl_3$) δ 27.88.
HRMS (ESI) m/z: $[M+H]^+$ Calcd for $C_{26}H_{22}Cl_2N_4OP$ 507.0903; Found 507.0920.
IR (ATR): 2978, 1582, 1390, 1193, 1086, 1013, 751, 559 cm^{-1}.

(2-(9-Isopropyl-9H-purin-6-yl)phenyl)bis(4-methoxyphenyl)phosphine oxide (**3r**)

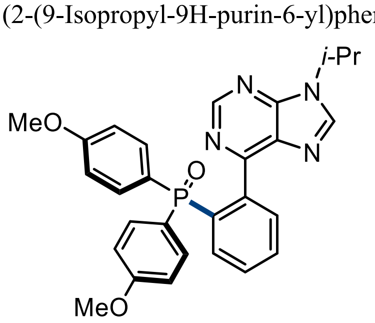

Prepared according to standard procedure A using [Ru(OAc)$_2$(p-cymene)] (10 mol %) on a 0.3 mmol scale, purification by column chromatography on silica gel (DCM/MeOH = 20/1, R_f = 0.13) yielded **3r** (129.9 mg, 87%) as a colorless sticky oil.
^{1}H NMR (300 MHz, $CDCl_3$) δ 8.56 (s, 1H), 7.97 (s, 1H), 7.83 (dd, J = 13.3, 7.6 Hz, 1H), 7.72 – 7.67 (m,

1H), 7.58 (t, J = 7.5 Hz, 1H), 7.49 – 7.40 (m, 5H), 6.65 (dd, J = 8.7, 2.0 Hz, 4H), 4.78 – 4.69 (m, 1H), 3.66 (s, 6H), 1.52 (d, J = 6.8 Hz, 6H).

^{13}C NMR (75 MHz, CDCl$_3$) δ 172.9 (C$_q$), 161.3 (d, J = 2.9 Hz) (C$_q$), 156.8 (d, J = 3.4 Hz) (C$_q$), 150.8 (CH), 150.7 (C$_q$), 141.9 (CH), 139.3 (d, J = 7.5 Hz) (C$_q$), 134.9 (d, J = 10.1 Hz) (CH), 133.3 (d, J = 11.3 Hz) (CH), 131.8 (d, J = 45.0 Hz) (C$_q$), 131.2 (d, J = 2.1 Hz) (CH), 131.0 (CH), 128.7 (d, J = 11.8 Hz) (CH), 124.5 (d, J = 113.1 Hz) (C$_q$), 113.0 (d, J = 13.4 Hz) (CH), 54.9 (CH$_3$), 47.1 (CH), 22.1 (CH$_3$).

^{31}P NMR (121 MHz, CDCl$_3$) δ 29.04.

HRMS (ESI) m/z: [M+H]$^+$ Calcd for $C_{28}H_{28}N_4O_3P$ 499.1894; Found 499.1900.

IR (ATR): 2978, 1596, 1248, 1101, 1024, 931, 734, 532 cm^{-1}.

Di([1,1'-biphenyl]-4-yl)(2-(9-isopropyl-9H-purin-6-yl)phenyl)phosphine oxide (**3s**)

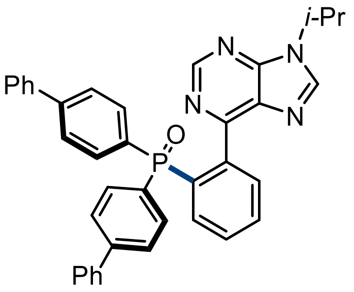

Prepared according to standard procedure A using [Ru(OAc)$_2$(p-cymene)] (10 mol %) on a 0.3 mmol scale, purification by column chromatography on silica gel (DCM/MeOH = 20/1, R$_f$ = 0.15) yielded **3s** (88.6 mg, 50%) as a colorless sticky oil.

^{1}H NMR (300 MHz, CDCl$_3$) δ 8.65 (s, 1H), 8.04 – 7.96 (m, 2H), 7.92 – 7.88 (m, 1H), 7.78 – 7.70 (m, 5H), 7.62 – 7.52 (m, 5H), 7.49 – 7.44 (m, 5H), 7.42 – 7.34 (m, 5H), 4.69 – 4.56 (m, 1H), 1.33 (d, J = 6.7 Hz, 6H).

^{13}C NMR (101 MHz, CDCl$_3$) δ 173.2 (C$_q$), 156.6 (d, J = 3.3 Hz) (C$_q$), 151.0 (CH), 151.0 (C$_q$), 143.4 (d, J = 2.7 Hz) (C$_q$), 141.9 (CH), 139.7 (C$_q$), 139.6 (d, J = 7.4 Hz) (C$_q$), 135.4 (d, J = 10.3 Hz) (CH), 132.4 (d, J = 101.9 Hz) (C$_q$), 132.2 (d, J = 10.2 Hz) (CH), 132.1 (d, J = 107.8 Hz) (C$_q$), 131.6 (CH), 131.6 (CH), 129.1 (d, J = 12.0 Hz) (CH), 128.8 (CH), 127.9 (CH), 127.0 (CH), 126.2 (d, J = 12.7 Hz) (CH), 47.1 (CH), 22.0 (CH$_3$).

^{31}P NMR (121 MHz, CDCl$_3$) δ 29.01.

HRMS (ESI) m/z: [M+H]$^+$ Calcd for $C_{38}H_{32}N_4OP$ 591.2308; Found 591.2320.

IR (ATR): 2978, 1597, 1391, 1177, 1102, 1007, 733, 529 cm^{-1}.

Ethyl (2-(9-isopropyl-9H-purin-6-yl)phenyl)(phenyl)phosphinate (**3t**)

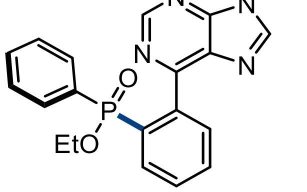

Prepared according to standard procedure A using [Ru(OAc)$_2$(p-cymene)] (10 mol %) on a 0.3 mmol

scale, purification by column chromatography on silica gel (DCM/MeOH = 20/1, R_f = 0.20) yielded **3t** (37.6 mg, 31%) as a colorless solid. M.p.: 153-154 °C

^{1}H NMR (300 MHz, $CDCl_3$) δ 8.74 (s, 1H), 8.22 – 8.13 (m, 1H), 7.94 (s, 1H), 7.77 – 7.72 (m, 1H), 7.61 – 7.52 (m, 4H), 7.26 – 7.17 (m, 3H), 4.91 – 4.79 (m, 1H), 3.96 – 3.83 (m, 2H), 1.58 (d, *J* = 6.8 Hz, 6H), 1.10 (t, *J* = 7.0 Hz, 3H).

^{13}C NMR (75 MHz, $CDCl_3$) δ 157.1 (d, *J* = 3.6 Hz) (C_q), 151.2 (C_q), 151.0 (CH), 142.1 (CH), 141.3 (C_q), 139.6 (d, *J* = 9.1 Hz) (C_q), 134.3 (d, *J* = 8.0 Hz) (CH), 133.1 (d, *J* = 140.9 Hz) (C_q), 132.0 (CH), 131.7 (d, *J* = 2.6 Hz) (CH), 131.2 (d, *J* = 10.5 Hz) (CH), 131.0 (d, *J* = 2.9 Hz) (CH), 131.0 (d, *J* = 133.7 Hz) (C_q), 129.0 (d, *J* = 12.0 Hz) (CH), 127.7 (d, *J* = 13.6 Hz) (CH), 60.7 (d, *J* = 5.7 Hz) (CH_2), 47.3 (CH), 22.5 (CH_3), 22.5 (CH_3), 16.1 (d, *J* = 6.9 Hz) (CH_3).

^{31}P NMR (121 MHz, $CDCl_3$) δ 29.94.

HRMS (ESI) m/z: $[M+H]^+$ Calcd for $C_{22}H_{24}N_4O_2P$ 407.1631; Found 407.1634.

IR (ATR): 2977, 1581, 1330, 1214, 1024, 930, 735, 648, 558 cm^{-1}.

Isopropyl(2-(9-isopropyl-9H-purin-6-yl)phenyl)(phenyl)phosphine oxide (**3u**)

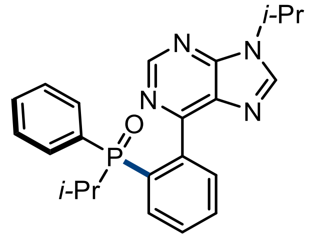

Prepared according to standard procedure A using [Ru(OAc)$_2$(*p*-cymene)] (10 mol %) on a 0.3 mmol scale, purification by column chromatography on silica gel (DCM/MeOH = 20/1, R_f = 0.16) yielded **3u** (72.2 mg, 60%) as a colorless sticky oil.

^{1}H NMR (400 MHz, $CDCl_3$) δ 8.79 (s, 1H), 8.45 – 8.34 (m, 1H), 7.84 (s, 1H), 7.64 – 7.54 (m, 3H), 7.22 – 7.14 (m, 2H), 6.93 – 6.85 (m, 3H), 4.84 – 4.70 (m, 1H), 3.17 – 3.03 (m, 1H), 1.56 (dd, *J* = 6.8, 4.4 Hz, 6H), 1.31 – 1.15 (m, 6H).

^{13}C NMR (101 MHz, $CDCl_3$) δ 157.2 (d, *J* = 3.2 Hz) (C_q), 150.8 (C_q), 150.6 (CH), 142.0 (CH), 138.3 (d, *J* = 7.8 Hz) (C_q), 134.3 (d, *J* = 6.9 Hz) (CH), 132.9 (d, *J* = 95.7 Hz) (C_q), 132.3 (d, *J* = 90.5 Hz) (C_q), 131.7 (C_q), 131.0 (d, *J* = 9.1 Hz) (CH), 130.9 (d, *J* = 2.4 Hz) (CH), 130.2 (d, *J* = 8.9 Hz) (CH), 129.7 (d, *J* = 2.6 Hz) (CH), 128.8 (d, *J* = 10.4 Hz) (CH), 127.1 (d, *J* = 11.3 Hz) (CH), 47.2 (CH), 26.8 (d, *J* = 73.3 Hz) (CH), 22.4 (CH_3), 22.2 (CH_3), 16.0 (d, *J* = 3.4 Hz) (CH_3), 15.9 (d, *J* = 2.6 Hz) (CH_3). ^{31}P NMR (162 MHz, $CDCl_3$) δ 39.61.

HRMS (ESI) m/z: $[M+H]^+$ Calcd for $C_{23}H_{26}N_4OP$ 405.1839; Found 405.1841.

IR (ATR): 2975, 1582, 1393, 1215, 1176, 1026, 734, 537 cm^{-1}.

(2-(9-Isopropyl-9H-purin-6-yl)phenyl)di(thiophen-2-yl)phosphine oxide (**3v**)

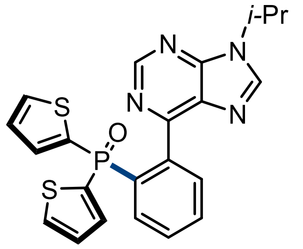

Prepared according to standard procedure A using [Ru(OAc)$_2$(*p*-cymene)] (10 mol %) on a 0.3 mmol scale, purification by column chromatography on silica gel (DCM/MeOH = 20/1, R_f = 0.26) yielded **3v** (28.3 mg, 21%) as a colorless sticky oil.

^{1}H NMR (300 MHz, CDCl$_3$) δ 8.72 (s, 1H), 8.09 – 8.01 (m, 3H), 7.74 – 7.67 (m, 1H), 7.61 – 7.47 (m, 5H), 7.00 – 6.96 (m, 2H), 4.96 – 4.82 (m, 1H), 1.63 (d, *J* = 6.8 Hz, 6H).

^{13}C NMR (75 MHz, CDCl$_3$) δ 156.1 (d, *J* = 3.4 Hz) (C$_q$), 151.2 (C$_q$), 151.1 (CH), 142.2 (CH), 139.4 (d, *J* = 7.3 Hz) (C$_q$), 136.2 (d, *J* = 10.8 Hz) (CH), 135.8 (d, *J* = 123.5 Hz) (C$_q$), 134.8 (d, *J* = 10.9 Hz) (CH), 133.1 (d, *J* = 113.4 Hz) (C$_q$), 133.1 (d, *J* = 5.5 Hz) (CH), 132.0 (d, *J* = 2.7 Hz) (CH), 131.8 (d, *J* = 10.0 Hz) (CH), 131.7 (C$_q$), 129.2 (d, *J* = 12.9 Hz) (CH), 127.5 (d, *J* = 14.9 Hz) (CH), 47.2 (CH), 22.5 (CH$_3$).

^{31}P NMR (121 MHz, CDCl$_3$) δ 14.49.

HRMS (ESI) m/z: [M+H]$^+$ Calcd for $C_{22}H_{20}N_4OPS_2$ 451.0811; Found 451.0817.

IR (ATR): 2977, 1576, 1331, 1190, 1004, 931, 715, 527 cm^{-1}.

(2-(9-Isopropyl-9H-purin-6-yl)phenyl)dimethylphosphine oxide (**3w**)

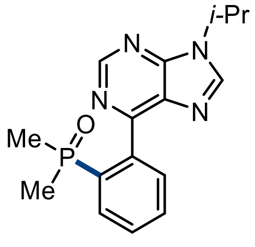

Prepared according to standard procedure A using [Ru(OAc)$_2$(*p*-cymene)] (10 mol %) on a 0.3 mmol scale, purification by column chromatography on silica gel (DCM/MeOH = 20/1, R_f = 0.04) yielded **3w** (55.6 mg, 59%) as a colorless solid. M.p.: 210-211 °C

^{1}H NMR (300 MHz, CDCl$_3$) δ 8.97 (s, 1H), 8.34 – 8.23 (m, 1H), 8.16 (s, 1H), 8.05 – 7.97 (m, 1H), 7.69 – 7.59 (m, 2H), 5.06 – 4.90 (m, 1H), 1.73 – 1.64 (m, 12H).

^{13}C NMR (75 MHz, CDCl$_3$) δ 157.3 (d, *J* = 2.6 Hz) (C$_q$), 151.8 (C$_q$), 150.9 (CH), 142.8 (CH), 137.4 (d, *J* = 7.2 Hz) (C$_q$), 134.5 (d, *J* = 91.6 Hz) (C$_q$), 133.5 (d, *J* = 7.9 Hz) (CH), 132.3 (C$_q$), 132.1 (d, *J* = 9.0 Hz) (CH), 131.2 (d, *J* = 2.6 Hz) (CH), 129.6 (d, *J* = 10.9 Hz) (CH), 47.5 (CH), 22.5 (CH$_3$), 19.5 (d, *J* = 73.1 Hz) (CH$_3$).

^{31}P NMR (121 MHz, CDCl$_3$) δ 36.29.

HRMS (ESI) m/z: [M+H]$^+$ Calcd for $C_{16}H_{20}N_4OP$ 315.1369; Found 315.1371.

IR (ATR): 2977, 1582, 1331, 1172, 932, 734, 649, 445 cm^{-1}.

7-Chloro-5-(2-(diphenylphosphoryl)phenyl)-1-methyl-1,3-dihydro-2H-benzo[e][1,4]diazepin-2-one (**3x**)

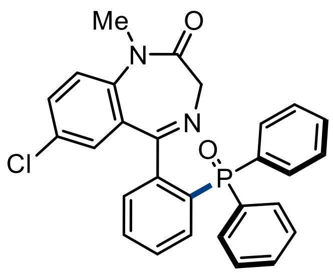

Prepared according to standard procedure A using [Ru(OAc)$_2$(*p*-cymene)] (10 mol %) on a 0.3 mmol scale, purification by column chromatography on silica gel (DCM/MeOH = 20/1, R_f = 0.18) yielded **3x** (130.6 mg, 90%) as a colorless solid. M.p.: 238-239 °C

^{1}H NMR (300 MHz, $CDCl_3$) δ 7.54 – 7.48 (m, 1H), 7.42 – 7.25 (m, 12H), 7.24 – 7.17 (m, 1H), 7.12 – 7.03 (m, 2H), 6.69 (d, *J* = 2.1 Hz, 1H), 4.46 (d, *J* = 10.9 Hz, 1H), 3.49 (d, *J* = 11.2 Hz, 1H), 3.38 (s, 3H).

^{13}C NMR (75 MHz, $CDCl_3$) δ 169.7 (d, *J* = 3.6 Hz) (C_q), 168.3 (C_q), 143.7 (d, *J* = 6.4 Hz) (C_q), 142.3 (C_q), 134.1 (d, *J* = 11.6 Hz) (CH), 132.0 (d, *J* = 100.1 Hz) (C_q), 131.6 (CH), 131.6 (CH), 131.5 (CH), 130.9 (CH), 129.5 (d, *J* = 168.7 Hz) (C_q), 129.4 (CH), 128.6 (d, *J* = 12.2 Hz) (CH), 128.2 (d, *J* = 12.3 Hz) (CH), 122.4 (CH), 56.3 (CH_2), 35.4 (CH_3).

^{31}P NMR (121 MHz, $CDCl_3$) δ 30.64.

HRMS (ESI) m/z: $[M+H]^+$ Calcd for $C_{28}H_{23}ClN_2O_2P$ 485.1180; Found 485.1190.

IR (ATR): 3057, 1666, 1485, 1402, 1116, 918, 717, 526 cm^{-1}.

7-Chloro-5-(2-(dimethylphosphoryl)phenyl)-1-methyl-1,3-dihydro-2H-benzo[e][1,4]diazepin-2-one (**3y**)

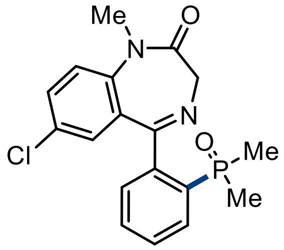

Prepared according to standard procedure A using [Ru(OAc)$_2$(*p*-cymene)] (10 mol %) on a 0.3 mmol scale, purification by column chromatography on silica gel (DCM/MeOH = 20/1, R_f = 0.11) yielded **3y** (96.6 mg, 89%) as a colorless sticky oil.

^{1}H NMR (300 MHz, $CDCl_3$) δ 7.82 (dd, *J* = 11.9, 7.5 Hz, 1H), 7.51 – 7.37 (m, 3H), 7.22 (d, *J* = 8.8 Hz, 1H), 7.16 – 7.09 (m, 1H), 7.03 (d, *J* = 2.4 Hz, 1H), 4.74 (d, *J* = 10.3 Hz, 1H), 3.77 (d, *J* = 10.2 Hz, 1H), 3.37 (s, 3H), 1.69 (dd, *J* = 48.9, 12.9 Hz, 6H).

^{13}C NMR (75 MHz, $CDCl_3$) δ 170.0 (C_q), 168.6 (C_q), 142.2 (C_q), 142.1 (C_q), 134.8 (d, *J* = 93.9 Hz) (C_q), 131.7 (CH), 131.5 (d, *J* = 3.5 Hz) (CH), 131.2 (d, *J* = 8.7 Hz) (CH), 131.0 (d, *J* = 1.8 Hz) (CH), 130.0 (d, *J* = 117.6 Hz) (C_q), 129.6 (CH), 129.4 (CH), 129.3 (C_q), 122.5 (CH), 56.5 (CH_2), 35.1 (CH_3), 19.8 (d, *J* = 29.6 Hz) (CH_3), 18.8 (d, *J* = 28.1 Hz) (CH_3).

^{31}P NMR (162 MHz, $CDCl_3$) δ 36.45.

HRMS (ESI) m/z: $[M+H]^+$ Calcd for $C_{18}H_{19}ClN_2O_2P$ 361.0867; Found 361.0870.

IR (ATR): 2915, 1665, 1470, 1300, 1168, 931, 722, 433 cm^{-1}.

Methyl 3-(4-(2-(diphenylphosphoryl)phenyl)-5-phenyloxazol-2-yl)propanoate (**3z**)

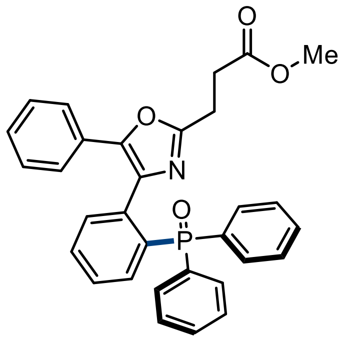

Prepared according to standard procedure A using [Ru(OAc)$_2$(*p*-cymene)] (10 mol %) on a 0.3 mmol scale, purification by column chromatography on silica gel (DCM/MeOH = 20/1, R_f = 0.24) yielded **3z** (71.5 mg, 47%) as a colorless sticky oil.

^{1}H NMR (300 MHz, CDCl$_3$) δ 8.07 – 7.96 (m, 1H), 7.58 – 7.46 (m, 6H), 7.33 – 7.25 (m, 3H), 7.20 – 7.08 (m, 7H), 7.06 – 6.99 (m, 2H), 3.61 (s, 3H), 2.82 (t, *J* = 7.6 Hz, 2H), 2.58 (t, *J* = 7.6 Hz, 2H).

^{13}C NMR (101 MHz, CDCl$_3$) δ 172.4 (C_q), 159.9 (C_q), 146.1 (C_q), 136.3 (d, *J* = 8.3 Hz) (C_q), 134.9 (d, *J* = 9.4 Hz) (CH), 133.3 (C_q), 133.0 (d, *J* = 100.4 Hz) (C_q), 132.7 (d, *J* = 103.7 Hz) (C_q), 132.2 (CH), 132.0 (d, *J* = 9.6 Hz) (CH), 131.8 (d, *J* = 10.1 Hz) (CH), 131.3 (d, *J* = 2.7 Hz) (CH), 128.8 (d, *J* = 11.6 Hz) (CH), 128.2 (CH), 127.8 (C_q), 127.7 (CH), 127.6 (CH), 125.0 (CH), 51.8 (CH_3), 30.1 (CH_2), 22.9 (CH_2).

^{31}P NMR (121 MHz, CDCl$_3$) δ 28.61.

HRMS (ESI) m/z: $[M+H]^+$ Calcd for $C_{31}H_{27}NO_4P$ 508.1672; Found 508.1677.

IR (ATR): 3052, 1737, 1437, 1331, 1179, 1118, 692, 545 cm^{-1}.

(2-(9-((3aR,4R,6R,6aR)-6-(Hydroxymethyl)-2,2-dimethyltetrahydrofuro[3,4-d][1,3]dioxol-4-yl)-9H-purin-6-yl)phenyl)diphenylphosphine oxide (**3aa**)

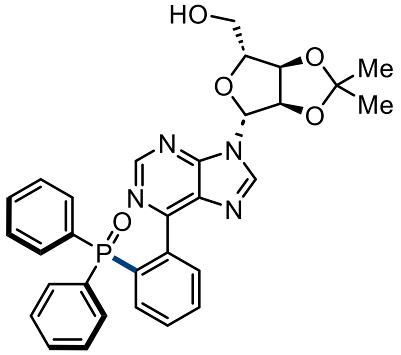

Prepared according to standard procedure A using [Ru(OAc)$_2$(*p*-cymene)] (10 mol %) on a 0.3 mmol scale, purification by column chromatography on silica gel (DCM/MeOH = 20/1, R_f = 0.16) yielded **3aa** (73.3 mg, 43%) as a colorless sticky oil.

^{1}H NMR (300 MHz, CDCl$_3$) δ 8.50 (s, 1H), 8.02 (s, 1H), 7.84 – 7.71 (m, 2H), 7.66 – 7.59 (m, 1H), 7.57 – 7.45 (m, 5H), 7.32 – 7.24 (m, 2H), 7.22 – 7.14 (m, 4H), 5.84 (d, *J* = 4.5 Hz, 1H), 5.49 (s, 1H), 5.12 – 4.99 (m, 2H), 4.47 – 4.40 (m, 1H), 3.83 (dd, *J* = 12.6, 1.5 Hz, 1H), 3.68 (d, *J* = 11.0 Hz, 1H), 1.58 (s, 3H), 1.32 (s, 3H).

^{13}C NMR (75 MHz, CDCl$_3$) δ 157.9 (d, *J* = 3.2 Hz) (C_q), 150.7 (CH), 150.1 (C_q), 144.1 (CH), 139.2 (d, *J* = 6.9 Hz) (C_q), 135.3 (d, *J* = 10.4 Hz) (CH), 134.2 (d, *J* = 4.6 Hz) (C_q), 132.5 (d, *J* = 100.7 Hz) (C_q),

132.3 (d, J = 97.3 Hz) (C_q), 131.7 (CH), 131.6 (CH), 131.1 (d, J = 2.7 Hz) (CH), 129.3 (d, J = 12.0 Hz) (CH), 127.9 (d, J = 2.1 Hz) (CH), 127.7 (d, J = 2.2 Hz) (CH), 114.1 (C_q), 93.7 (CH), 86.2 (CH), 82.9 (CH), 81.5 (CH), 63.2 (CH_2), 27.5 (CH_3), 25.2 (CH_3).

^{31}P NMR (121 MHz, $CDCl_3$) δ 29.19.

HRMS (ESI) m/z: $[M+H]^+$ Calcd for $C_{31}H_{30}N_4O_5P$ 569.1948; Found 569.1966.

IR (ATR): 2989, 1579, 1384, 1171, 1070, 849, 719, 535 cm^{-1}.

(4-(9-Isopropyl-9H-purin-6-yl)phenyl)diphenylphosphine oxide (**4a**)

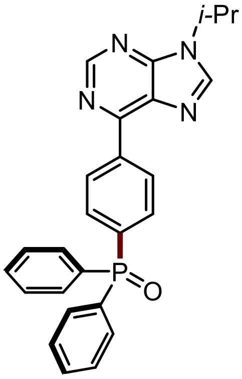

Prepared according to standard procedure B using [$RuCl_3 \cdot 3H_2O$] (10 mol %) on a 0.3 mmol scale, purification by column chromatography on silica gel (DCM/MeOH = 20/1, R_f = 0.44) yielded **4a** (107.7 mg, 82%) as a colorless solid. M.p.: 185-186 °C

^{1}H NMR (400 MHz, $CDCl_3$) δ 8.87 (s, 1H), 8.79 (dd, J = 8.3, 2.5 Hz, 2H), 8.10 (s, 1H), 7.78 (dd, J = 11.6, 8.3 Hz, 2H), 7.65 – 7.54 (m, 4H), 7.42 – 7.36 (m, 2H), 7.34 – 7.28 (m, 4H), 4.85 – 4.74 (m, 1H), 1.49 (d, J = 6.8 Hz, 6H).

^{13}C NMR (101 MHz, $CDCl_3$) δ 152.5 (C_q), 151.9 (C_q), 151.5 (CH), 142.4 (CH), 138.8 (d, J = 2.9 Hz) (C_q), 134.3 (d, J = 102.6 Hz) (C_q), 131.9 (d, J = 104.3 Hz) (C_q), 131.8 (d, J = 10.0 Hz) (CH), 131.6 (d, J = 4.5 Hz) (CH), 131.6 (d, J = 2.6 Hz) (CH), 131.3 (C_q), 129.1 (d, J = 12.0 Hz) (CH), 128.1 (d, J = 12.1 Hz) (CH), 47.0 (CH), 22.0 (CH_3).

^{31}P NMR (162 MHz, $CDCl_3$) δ 28.57.

HRMS (ESI) m/z: $[M+H]^+$ Calcd for $C_{26}H_{24}N_4OP$ 439.1682; Found 439.1687.

IR (ATR): 3058, 2978, 1576, 1326, 1184, 1117, 805, 537 cm^{-1}.

(4-(9-Methyl-9H-purin-6-yl)phenyl)diphenylphosphine oxide (**4b**)

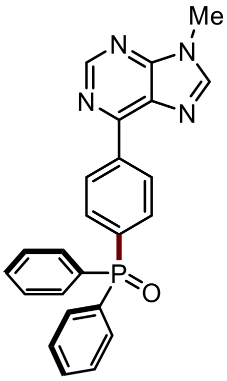

Prepared according to standard procedure B using [$RuCl_3 \cdot 3H_2O$] (10 mol %) on a 0.3 mmol scale, purification by column chromatography on silica gel (DCM/MeOH = 20/1, R_f = 0.40) yielded **4b** (52.9 mg, 43%) as a colorless sticky oil.

^{1}H NMR (300 MHz, $CDCl_3$) δ 9.04 (s, 1H), 8.85 (dd, *J* = 8.4, 2.6 Hz, 2H), 8.12 (s, 1H), 7.90 – 7.83 (m, 2H), 7.74 – 7.66 (m, 4H), 7.56 – 7.52 (m, 2H), 7.49 – 7.44 (m, 4H), 3.94 (s, 3H).

^{13}C NMR (75 MHz, $CDCl_3$) δ 153.4 (d, *J* = 0.9 Hz) (C_q), 153.0 (C_q), 152.4 (CH), 145.4 (CH), 139.0 (d, *J* = 2.9 Hz) (C_q), 134.7 (d, *J* = 102.8 Hz) (C_q), 132.4 (d, *J* = 10.1 Hz) (CH), 132.2 (CH), 132.1 (d, *J* = 104.6 Hz) (C_q), 132.1 (d, *J* = 2.9 Hz) (CH), 131.2 (C_q), 129.6 (d, *J* = 12.1 Hz) (CH), 128.5 (d, *J* = 12.2 Hz) (CH), 29.9 (CH_3).

^{31}P NMR (121 MHz, $CDCl_3$) δ 29.17.

HRMS (ESI) m/z: $[M+H]^+$ Calcd for $C_{24}H_{20}N_4OP$ 411.1369; Found 411.1368.

IR (ATR): 3059, 1578, 1437, 1180, 1121, 927, 733, 537 cm^{-1}.

(4-(9-Benzyl-9H-purin-6-yl)phenyl)diphenylphosphine oxide (**4c**)

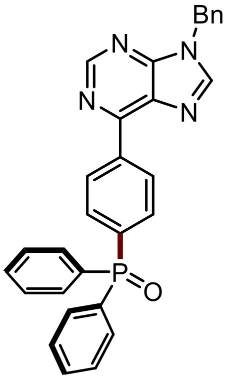

Prepared according to standard procedure B using [$RuCl_3 \cdot 3H_2O$] (10 mol %) on a 0.3 mmol scale, purification by column chromatography on silica gel (DCM/MeOH = 20/1, R_f = 0.38) yielded **4c** (76.0 mg, 52%) as a colorless sticky oil.

^{1}H NMR (400 MHz, $CDCl_3$) δ 9.06 (s, 1H), 8.86 (dd, *J* = 8.2, 2.6 Hz, 2H), 8.10 (s, 1H), 7.86 (dd, *J* = 11.6, 8.2 Hz, 2H), 7.73 – 7.67 (m, 4H), 7.57 – 7.52 (m, 2H), 7.50 – 7.44 (m, 4H), 7.38 – 7.30 (m, 5H), 5.47 (s, 2H).

^{13}C NMR (101 MHz, $CDCl_3$) δ 153.5 (C_q), 152.70 (C_q), 152.6 (CH), 144.6 (CH), 138.9 (d, *J* = 2.9 Hz) (C_q), 134.9 (d, *J* = 102.7 Hz) (C_q), 135.0 (Cq), 132.3 (d, *J* = 10.0 Hz) (CH), 132.2 (d, *J* = 104.4 Hz) (C_q), 132.1 (CH), 132.0 (d, *J* = 3.9 Hz) (CH), 131.2 (C_q), 129.6 (d, *J* = 12.0 Hz) (CH), 129.1 (CH), 128.6 (d, *J* = 4.4 Hz) (CH), 128.4 (CH), 127.8 (CH), 47.3 (CH_2).

^{31}P NMR (162 MHz, $CDCl_3$) δ 28.91.

HRMS (ESI) m/z: $[M+H]^+$ Calcd for $C_{30}H_{24}N_4OP$ 487.1682; Found 487.1695.

IR (ATR): 3061, 1577, 1325, 1119, 908, 722, 645, 536 cm^{-1}.

(2R,3R,4R,5R)-2-(Acetoxymethyl)-5-(6-(4-(diphenylphosphoryl)phenyl)-9H-purin-9-yl)tetrahydrofuran-3,4-diyl diacetate (**4d**)

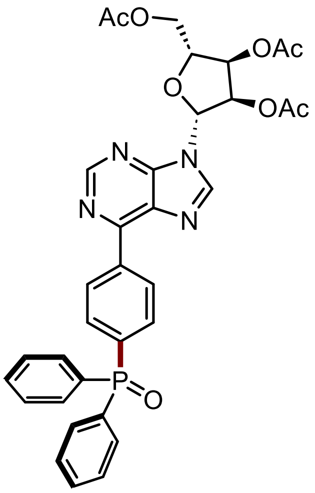

Prepared according to standard procedure B using [$RuCl_3 \cdot 3H_2O$] (10 mol %) on a 0.3 mmol scale, purification by column chromatography on silica gel (DCM/MeOH = 20/1, R_f = 0.40) yielded **4d** (45.3 mg, 23%) as a colorless sticky oil.

^{1}H NMR (300 MHz, $CDCl_3$) δ 9.05 (s, 1H), 8.84 (dd, *J* = 8.6, 2.6 Hz, 2H), 8.29 (s, 1H), 7.91 – 7.84 (m, 2H), 7.75 – 7.67 (m, 4H), 7.59 – 7.53 (m, 2H), 7.51 – 7.45 (m, 4H), 6.29 (d, *J* = 5.2 Hz, 1H), 6.00 (t, *J* = 5.4 Hz, 1H), 5.73 – 5.66 (m, 1H), 4.51 – 4.37 (m, 3H), 2.18 – 2.07 (m, 9H).

^{13}C NMR (75 MHz, $CDCl_3$) δ 170.3 (C_q), 169.6 (C_q), 169.4 (C_q), 154.1 (C_q), 152.7 (CH), 152.2 (C_q), 143.1 (CH), 138.6 (d, *J* = 2.9 Hz) (C_q), 135.2 (d, *J* = 102.4 Hz) (C_q), 132.4 (d, *J* = 10.0 Hz) (CH), 132.2 (d, *J* = 104.5 Hz) (C_q), 132.1 (d, *J* = 10.0 Hz) (CH), 132.0 (CH), 132.1 (C_q), 129.6 (d, *J* = 12.0 Hz), (CH) 128.6 (d, *J* = 12.2 Hz) (CH), 86.5 (CH), 80.4 (CH), 73.1 (CH), 70.6 (CH), 63.0 (CH_2), 20.8 (CH_3), 20.5 (CH_3), 20.4 (CH_3).

^{31}P NMR (121 MHz, $CDCl_3$) δ 28.83.

HRMS (ESI) m/z: $[M+H]^+$ Calcd for $C_{34}H_{32}N_4O_8P$ 655.1952; Found 655.1965.

IR (ATR): 2925, 1746, 1580, 1366, 1214, 1046, 739, 537 cm^{-1}.

(2-Fluoro-4-(9-isopropyl-9H-purin-6-yl)phenyl)diphenylphosphine oxide (**4e**)

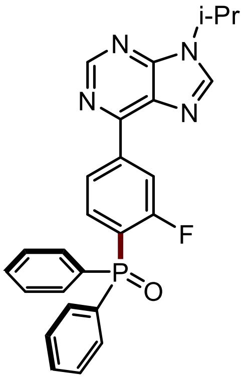

Prepared according to standard procedure A using [$Ru(OAc)_2$(*p*-cymene)] (10 mol %) on a 0.3 mmol scale, purification by column chromatography on silica gel (DCM/MeOH = 20/1, R_f = 0.40) yielded **4e** (93.1 mg, 68%) as a colorless sticky oil.

^{1}H NMR (300 MHz, $CDCl_3$) δ 9.03 (s, 1H), 8.83 – 8.72 (m, 1H), 8.64 – 8.52 (m, 1H), 8.22 (s, 1H), 8.15 – 8.03 (m, 1H), 7.85 – 7.73 (m, 4H), 7.61 – 7.45 (m, 6H), 5.07 – 4.90 (m, 1H), 1.68 (d, *J* = 6.8 Hz, 6H).

^{13}C NMR (75 MHz, $CDCl_3$) δ 162.9 (d, $J_{C\text{-}F}$ = 249.8 Hz) (C_q), 152.5 (C_q), 151.9 (CH), 151.8 (d, *J* = 2.5

Hz) (C_q), 142.8 (CH), 142.4 (dd, J = 8.4, 2.3 Hz) (C_q), 135.1 – 134.5 (m) (CH), 132.2 (d, J = 2.8 Hz) (CH), 131.9 (d, J = 108.2 Hz) (C_q), 131.7 (dd, J = 10.6, 1.8 Hz) (CH), 128.5 (d, J = 12.8 Hz) (CH), 125.6 (dd, J = 10.6, 3.3 Hz) (CH), 122.1 (dd, J = 19.1 Hz, 99.0 Hz) (C_q), 116.9 (dd, J = 25.1, 5.7 Hz) (CH), 47.5 (CH), 22.5 (CH_3).

^{31}P NMR (121 MHz, $CDCl_3$) δ 24.44.

^{19}F NMR (282 MHz, $CDCl_3$) δ -98.88.

HRMS (ESI) m/z: $[M+H]^+$ Calcd for $C_{26}H_{23}FN_4OP$ 457.1588; Found 457.1600.

IR (ATR): 3057, 2978, 1574, 1436, 1104, 897, 690, 521 cm^{-1}.

(4-(9-Isopropyl-9H-purin-6-yl)-2-methylphenyl)diphenylphosphine oxide (**4f**)

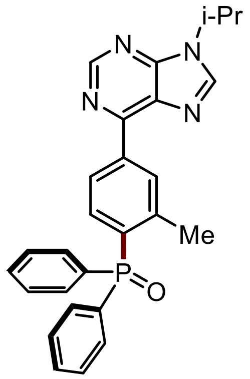

Prepared according to standard procedure A using $[Ru(OAc)_2(p\text{-cymene})]$ (10 mol %) on a 0.3 mmol scale, purification by column chromatography on silica gel (DCM/MeOH = 20/1, R_f = 0.43) yielded **4f** (50.2 mg, 37%) as a colorless sticky oil.

^{1}H NMR (300 MHz, $CDCl_3$) δ 9.04 (s, 1H), 8.67 – 8.51 (m, 2H), 8.20 (s, 1H), 7.76 – 7.66 (m, 4H), 7.61 – 7.54 (m, 2H), 7.53 – 7.45 (m, 4H), 7.24 (dd, J = 13.8, 8.1 Hz, 1H), 5.08 – 4.91 (m, 1H), 2.61 (s, 3H), 1.68 (d, J = 6.8 Hz, 6H).

^{13}C NMR (75 MHz, $CDCl_3$) δ 152.9 (d, J = 94.3 Hz) (C_q), 152.0 (CH), 143.7 (d, J = 8.1 Hz) (C_q), 142.4 (CH), 139.1 (d, J = 2.6 Hz) (C_q), 133.8 (d, J = 13.6 Hz) (CH), 132.4 (d, J = 103.8 Hz) (C_q), 132.4 (C_q), 132.2 (d, J = 10.3 Hz) (CH), 132.0 (CH), 131.9 (d, J = 2.6 Hz) (CH), 131.8 (C_q), 128.6 (d, J = 12.1 Hz) (CH), 126.6 (d, J = 12.8 Hz) (CH), 47.4 (CH), 22.5(CH_3), 21.8 (d, J = 4.4 Hz) (CH_3).

^{31}P NMR (121 MHz, $CDCl_3$) δ 31.59.

HRMS (ESI) m/z: $[M+H]^+$ Calcd for $C_{27}H_{26}N_4OP$ 453.1839; Found 453.1828.

IR (ATR): 2977, 2226, 1573, 1325, 1116, 807, 693, 547 cm^{-1}.

Bis(4-chlorophenyl)(4-(9-isopropyl-9H-purin-6-yl)phenyl)phosphine oxide (**4g**)

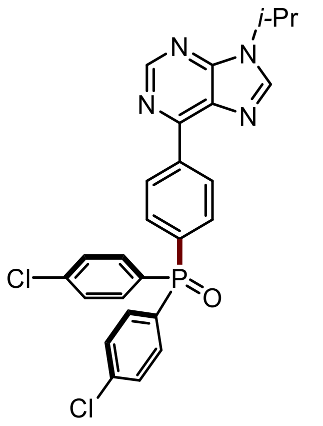

Prepared according to standard procedure B using [$RuCl_3 \cdot 3H_2O$] (10 mol %) on a 0.3 mmol scale, purification by column chromatography on silica gel (DCM/MeOH = 20/1, R_f = 0.44) yielded **4g** (94.2 mg, 62%) as a colorless sticky oil.

^{1}H NMR (300 MHz, $CDCl_3$) δ 8.99 (s, 1H), 8.85 (dd, J = 8.5, 2.7 Hz, 2H), 8.18 (s, 1H), 7.83 – 7.75 (m, 2H), 7.63 – 7.55 (m, 4H), 7.45 – 7.40 (m, 4H), 4.96 (h, J = 6.8 Hz, 1H), 1.64 (d, J = 6.8 Hz, 6H).

^{13}C NMR (75 MHz, $CDCl_3$) δ 152.9 (d, J = 1.0 Hz) (C_q), 152.3 (C_q), 152.0 (CH), 142.6 (CH), 139.5 (d, J = 2.9 Hz) (C_q), 138.9 (d, J = 3.4 Hz) (C_q), 133.5 (d, J = 104.7 Hz) (C_q), 133.3 (d, J = 10.9 Hz) (CH), 132.1 (d, J = 10.2 Hz) (CH), 131.7 (C_q), 130.3 (d, J = 106.0 Hz) (C_q), 129.7 (d, J = 12.2 Hz) (CH), 129.0 (d, J = 12.8 Hz) (CH), 47.4 (CH), 22.4 (CH_3).

^{31}P NMR (121 MHz, $CDCl_3$) δ 27.54.

HRMS (ESI) m/z: $[M+H]^+$ Calcd for $C_{26}H_{22}Cl_2N_4OP$ 507.0903; Found 507.0913.

IR (ATR): 2977, 1576, 1388, 1177, 1084, 1013, 930, 729, 562 cm^{-1}.

(4-(9-Isopropyl-9H-purin-6-yl)phenyl)di-p-tolylphosphine oxide (**4h**)

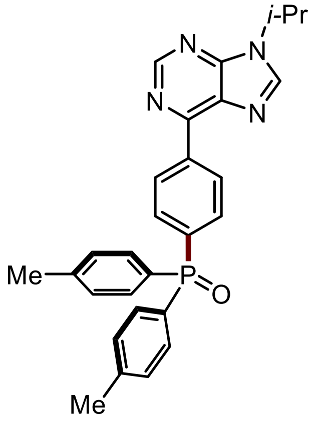

Prepared according to standard procedure B using [$RuCl_3 \cdot 3H_2O$] (10 mol %) on a 0.3 mmol scale, purification by column chromatography on silica gel (DCM/MeOH = 20/1, R_f = 0.42) yielded **4h** (62.8 mg, 45%) as a colorless sticky oil.

^{1}H NMR (400 MHz, $CDCl_3$) δ 9.07 (s, 1H), 8.89 – 8.85 (m, 2H), 8.23 (s, 1H), 7.93 – 7.87 (m, 2H), 7.65 – 7.59 (m, 4H), 7.32 – 7.29 (m, 4H), 5.10 – 4.96 (m, 1H), 2.44 (s, 6H), 1.72 (d, J = 6.7 Hz, 6H).

^{13}C NMR (75 MHz, $CDCl_3$) δ 153.5 (C_q), 152.3 (C_q), 152.1 (CH), 142.5 (CH), 142.4 (C_q), 138.9 (d, J = 3.0 Hz) (C_q), 135.3 (d, J = 102.7 Hz) (C_q), 132.3 (d, J = 10.1 Hz) (CH), 132.1 (d, J = 10.1 Hz) (CH),

131.8 (C_q), 129.5 (d, J = 12.0 Hz) (CH), 129.2 (d, J = 12.5 Hz) (CH), δ 129.2 (d, J = 106.8 Hz) (C_q), 47.4 (CH), 22.5 (CH_3), 21.6 (CH_3).

^{31}P NMR (162 MHz, $CDCl_3$) δ 29.16.

HRMS (ESI) m/z: $[M+H]^+$ Calcd for $C_{28}H_{28}N_4OP$ 467.1995; Found 467.2016.

IR (ATR): 2904, 1577, 1327, 1183, 1116, 930, 731, 542 cm^{-1}.

(4-(9-Isopropyl-9H-purin-6-yl)phenyl)bis(4-methoxyphenyl)phosphine oxide (**4i**)

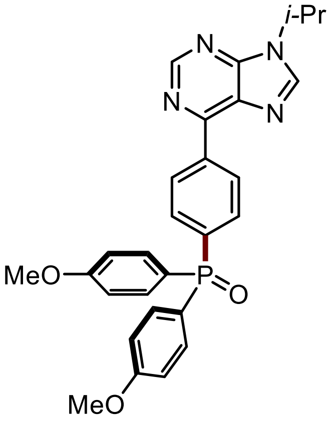

Prepared according to standard procedure B using [$RuCl_3 \cdot 3H_2O$] (10 mol %) on a 0.3 mmol scale, purification by column chromatography on silica gel (DCM/MeOH = 20/1, R_f = 0.33) yielded **4i** (96.6 mg, 65%) as a colorless sticky oil.

^{1}H NMR (300 MHz, $CDCl_3$) δ 8.99 (d, J = 1.2 Hz, 1H), 8.81 (dd, J = 8.2, 2.5 Hz, 2H), 8.17 (s, 1H), 7.90 – 7.81 (m, 2H), 7.62 – 7.53 (m, 4H), 6.95 – 6.91 (m, 4H), 5.02 – 4.87 (m, 1H), 3.80 (d, J = 1.3 Hz, 6H), 1.63 (d, J = 6.8 Hz, 6H).

^{13}C NMR (75 MHz, $CDCl_3$) δ 162.4 (d, J = 2.8 Hz) (C_q), 153.4 (C_q), 152.2 (C_q), 152.0 (CH), 142.5 (CH), 138.8 (d, J = 2.9 Hz) (C_q), 135.5 (d, J = 103.5 Hz) (C_q), 133.9 (d, J = 11.3 Hz) (CH), 132.2 (d, J = 10.1 Hz) (CH), 131.7 (C_q), 129.4 (d, J = 12.0 Hz) (CH), 123.6 (d, J = 111.2 Hz) (C_q), 114.0 (d, J = 13.2 Hz), 55.2 (CH), 47.3 (CH_3), 22.4 (CH_3).

^{31}P NMR (121 MHz, $CDCl_3$) δ 28.82.

HRMS (ESI) m/z: $[M+H]^+$ Calcd for $C_{28}H_{28}N_4O_3P$ 499.1894; Found 499.1895.

IR (ATR): 2977, 1569, 1250, 1118, 1026, 929, 731, 535 cm^{-1}.

(4-(9-Isopropyl-9H-purin-6-yl)phenyl)bis(4-(trifluoromethoxy)phenyl)phosphine oxide (**4j**)

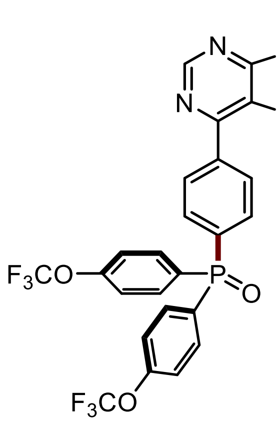

Prepared according to standard procedure B using [$RuCl_3$·$3H_2O$] (10 mol %) on a 0.3 mmol scale, purification by column chromatography on silica gel (DCM/MeOH = 20/1, R_f = 0.47) yielded **4j** (111.0 mg, 61%) as a colorless sticky oil.

^{1}H NMR (300 MHz, $CDCl_3$) δ 9.02 (s, 1H), 8.88 (dd, *J* = 8.0, 1.7 Hz, 2H), 8.19 (s, 1H), 7.87 – 7.70 (m, 6H), 7.32 (d, *J* = 8.1 Hz, 4H), 4.98 (p, *J* = 6.8 Hz, 1H), 1.66 (d, *J* = 6.8 Hz, 6H).

^{13}C NMR (75 MHz, $CDCl_3$) δ 153.0 (C_q), 152.4 (C_q), 152.2 (C_q), 152.1 (CH), 142.6 (CH), 139.7 (d, *J* = 2.4 Hz) (C_q), 134.1 (d, *J* = 11.0 Hz) (CH), 132.3 (d, *J* = 72.8 Hz) (C_q), 132.2 (d, *J* = 10.1 Hz) (CH), 130.5 (d, *J* = 105.8 Hz) (C_q), 129.8 (d, *J* = 12.1 Hz) (CH), 120.6 (d, *J* = 12.7 Hz) (CH), 120.2 (q, *J* = 259.0 Hz) (C_q), 47.5 (CH), 22.5 (CH_3).

^{31}P NMR (121 MHz, $CDCl_3$) δ 26.78.

HRMS (ESI) m/z: $[M+H]^+$ Calcd for $C_{28}H_{22}F_6N_4O_3P$ 607.1328; Found 607.1335.

IR (ATR): 2980, 1579, 1501, 1252, 1164, 1117, 748, 563 cm^{-1}.

Bis(3,5-dimethylphenyl)(4-(9-isopropyl-9H-purin-6-yl)phenyl)phosphine oxide (**4k**)

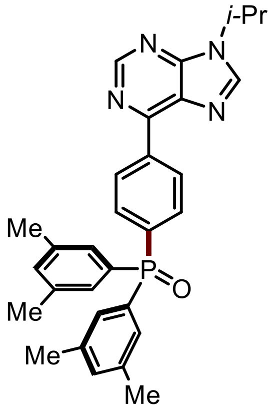

Prepared according to standard procedure B using [$RuCl_3$·$3H_2O$] (10 mol %) on a 0.3 mmol scale, purification by column chromatography on silica gel (DCM/MeOH = 20/1, R_f = 0.51) yielded **4k** (75.4 mg, 51%) as a colorless sticky oil.

^{1}H NMR (300 MHz, $CDCl_3$) δ 9.02 (s, 1H), 8.83 (dd, *J* = 8.4, 2.5 Hz, 2H), 8.18 (s, 1H), 7.86 (dd, *J* = 11.5, 8.4 Hz, 2H), 7.30 (d, *J* = 12.3 Hz, 4H), 7.15 (s, 2H), 4.97 (p, *J* = 6.8 Hz, 1H), 2.30 (s, 12H), 1.66 (d, *J* = 6.8 Hz, 6H).

^{13}C NMR (75 MHz, $CDCl_3$) δ 153.5 (C_q), 152.3 (C_q), 152.0 (CH), 142.4 (C_q), 138.8 (d, *J* = 2.8 Hz) (C_q), 138.1 (d, *J* = 12.8 Hz) (C_q), 135.3 (d, *J* = 101.7 Hz) (C_q), 133.7 (d, *J* = 2.8 Hz) (CH), 132.4 (d, *J* = 10.0

Hz) (CH), 132.1 (d, J = 103.5 Hz) (C_q), 131.7 (C_q), 129.6 (d, J = 9.9 Hz) (CH), 129.4 (d, J = 12.0 Hz) (CH), 47.4 (CH), 22.5 (CH_3), 21.2 (CH_3).

^{31}P NMR (121 MHz, $CDCl_3$) δ 29.43.

HRMS (ESI) m/z: $[M+H]^+$ Calcd for $C_{30}H_{32}N_4OP$ 495.2308; Found 495.2314.

IR (ATR): 2977, 1575, 1326, 1182, 849, 730, 569, 530 cm^{-1}.

(E)-(4-(1-(Methoxyimino)ethyl)phenyl)diphenylphosphine oxide (**4l**)

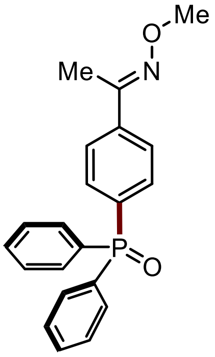

Prepared according to standard procedure A using [Ru(OAc)$_2$(p-cymene)] (10 mol %) on a 0.3 mmol scale, purification by column chromatography on silica gel (DCM/MeOH = 20/1, R_f = 0.30) yielded **4l** (31.8 mg, 30%) as a colorless sticky oil.

^{1}H NMR (400 MHz, $CDCl_3$) δ 7.80 – 7.76 (m, 2H), 7.74 – 7.67 (m, 6H), 7.61 – 7.57 (m, 2H), 7.53 – 7.48 (m, 4H), 4.05 (s, 3H), 2.26 (s, 3H).

^{13}C NMR (101 MHz, $CDCl_3$) δ 153.7 (C_q), 140.0 (d, J = 2.9 Hz) (C_q), 132.9 (d, J = 103.9 Hz) (C_q), 132.3 (d, J = 104.6 Hz) (C_q), 132.2 (d, J = 9.8 Hz) (CH), 132.1 (CH), 132.0 (CH), 128.5 (d, J = 12.0 Hz) (CH), 126.0 (d, J = 12.4 Hz) (CH), 62.2 (CH_3), 12.5 (CH_3).

^{31}P NMR (162 MHz, $CDCl_3$) δ 28.87.

HRMS (ESI) m/z: $[M+H]^+$ Calcd for $C_{21}H_{21}NO_2P$ 350.1304; Found 350.1310.

IR (ATR): 2935, 1436, 1181, 1118, 1046, 897, 709, 554 cm^{-1}.

(4-(1H-pyrazol-1-yl)phenyl)diphenylphosphine oxide (**4m**)

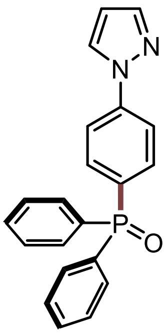

Prepared according to standard procedure B using [$RuCl_3 \cdot 3H_2O$] (10 mol %) on a 0.3 mmol scale, purification by column chromatography on silica gel (DCM/MeOH = 20/1, R_f = 0.53) yielded **4m** (33.0 mg, 32%) as a colorless sticky oil.

^{1}H NMR (300 MHz, $CDCl_3$) δ 7.98 (d, J = 2.5 Hz, 1H), 7.84 – 7.64 (m, 9H), 7.58 – 7.52 (m, 2H), 7.50

– 7.44 (m, 4H), 6.49 (dd, *J* = 2.5, 1.9 Hz, 1H).

^{13}C NMR (75 MHz, CDCl$_3$) δ 142.6 (d, *J* = 3.1 Hz) (C_q), 141.9 (CH), 133.6 (d, *J* = 10.7 Hz) (CH), 132.3 (d, *J* = 104.8 Hz) (C_q), 132.1 (d, *J* = 3.7 Hz) (CH), 132.0 (CH), 130.1 (d, *J* = 106.0 Hz) (C_q), 128.6 (d, *J* = 12.2 Hz) (CH), 126.8 (CH), 118.6 (d, *J* = 12.6 Hz) (CH), 108.4 (CH).

^{31}P NMR (121 MHz, CDCl$_3$) δ 28.52.

HRMS (ESI) m/z: $[M+H]^+$ Calcd for $C_{21}H_{18}N_2OP$ 345.1151; Found 345.1156.

IR (ATR): 3059, 1600, 1392, 1177, 1111, 933, 726, 548 cm^{-1}.

(4-(4,4-Dimethyl-4,5-dihydrooxazol-2-yl)phenyl)diphenylphosphine oxide (**4n**)

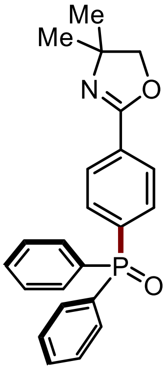

Prepared according to standard procedure B using [RuCl$_3$·3H$_2$O] (10 mol %) on a 0.3 mmol scale, purification by column chromatography on silica gel (DCM/MeOH = 20/1, R_f = 0.49) yielded **4n** (25.3 mg, 22%) as a colorless sticky oil.

^{1}H NMR (500 MHz, CDCl$_3$) δ 8.04 – 8.00 (m, 2H), 7.74 – 7.69 (m, 2H), 7.68 – 7.63 (m, 4H), 7.57 – 7.53 (m, 2H), 7.48 – 7.44 (m, 4H), 4.12 (s, 2H), 1.38 (s, 6H).

^{13}C NMR (126 MHz, CDCl$_3$) δ 161.2 (C_q), 135.5 (d, *J* = 102.3 Hz) (C_q), 132.1 (d, *J* = 104.6 Hz) (C_q), 132.1 (CH),132.0 (CH), 132.1 (CH), 131.4 (d, *J* = 2.8 Hz) (C_q), 128.6 (d, *J* = 12.3 Hz) (CH), 128.1 (d, *J* = 12.2 Hz) (CH), 79.3 (CH$_2$), 67.9 (C_q), 28.3 (CH$_3$).

^{31}P NMR (121 MHz, CDCl$_3$) δ 28.57.

HRMS (ESI) m/z: $[M+H]^+$ Calcd for $C_{23}H_{23}NO_2P$ 376.1461; Found 376.1469.

IR (ATR): 2965, 1649, 1437, 1197, 1118, 1063, 721, 547 cm^{-1}.

Diphenyl(4-(pyridin-2-yl)phenyl)phosphine oxide (**4o**)

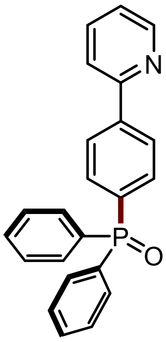

Prepared according to standard procedure B using [RuCl$_3$·3H$_2$O] (10 mol %) on a 0.3 mmol scale,

purification by column chromatography on silica gel (DCM/MeOH = 20/1, R_f = 0.56) yielded **4o** (47.8 mg, 45%) as a colorless sticky oil.

^{1}H NMR (300 MHz, $CDCl_3$) δ 8.71 (d, *J* = 4.6 Hz, 1H), 8.08 (dd, *J* = 8.4, 2.5 Hz, 2H), 7.82 – 7.67 (m, 8H), 7.58 – 7.53 (m, 2H), 7.50 – 7.44 (m, 4H), 7.31 – 7.26 (m, 1H).

^{13}C NMR (101 MHz, $CDCl_3$) δ 156.2 (C_q), 149.9 (CH), 142.8 (d, *J* = 2.8 Hz) (C_q), 136.9 (CH), 132.8 (d, *J* = 104.2 Hz) (C_q), 132.6 (d, *J* = 10.1 Hz) (CH), 132.4 (d, *J* = 104.3 Hz) (C_q), 132.1 (d, *J* = 10.0 Hz) (CH), 132.0 (d, *J* = 2.7 Hz) (CH), 128.5 (d, *J* = 12.2 Hz) (CH), 126.9 (d, *J* = 12.3 Hz) (CH), 122.9 (CH), 121.0 (CH).

^{31}P NMR (121 MHz, $CDCl_3$) δ 28.98.

HRMS (ESI) m/z: $[M+H]^+$ Calcd for $C_{23}H_{19}NOP$ 356.1199; Found 356.1203.

IR (ATR): 3055, 1586, 1436, 1182, 1119, 779, 705, 553 cm^{-1}.

(4-(5-Methylpyrimidin-2-yl)phenyl)diphenylphosphine oxide (**4p**)

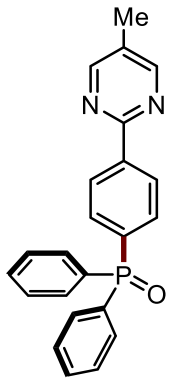

Prepared according to standard procedure B using [$RuCl_3 \cdot 3H_2O$] (10 mol %) on a 0.3 mmol scale, purification by column chromatography on silica gel (DCM/MeOH = 20/1, R_f = 0.31) yielded **4p** (50.9 mg, 46%) as a colorless solid. M.p.: 217-218 °C

^{1}H NMR (300 MHz, $CDCl_3$) δ 8.68 – 8.58 (m, 2H), 8.51 – 8.45 (m, 2H), 7.82 – 7.74 (m, 2H), 7.73 – 7.65 (m, 4H), 7.57 – 7.50 (m, 2H), 7.48 – 7.42 (m, 4H), 2.33 (s, 3H).

^{13}C NMR (75 MHz, $CDCl_3$) δ 161.4 (d, *J* = 1.0 Hz) (C_q), 157.4 (CH), 140.9 (d, *J* = 2.9 Hz) (C_q), 134.2 (d, *J* = 103.4 Hz) (C_q), 132.3 (d, *J* = 10.1 Hz) (CH), 132.4 (d, *J* = 104.3 Hz) (C_q), 132.0 (d, *J* = 10.0 Hz) (CH), 131.9 (CH), 129.1 (C_q), 128.5 (d, *J* = 12.2 Hz) (CH), 127.7 (d, *J* = 12.3 Hz) (CH), 15.5 (CH_3).

^{31}P NMR (121 MHz, $CDCl_3$) δ 28.94.

HRMS (ESI) m/z: $[M+H]^+$ Calcd for $C_{23}H_{20}N_2OP$ 371.1308; Found 371.1311.

IR (ATR): 3058, 1542, 1429, 1195, 1118, 791, 730, 545 cm^{-1}.

(4-(2-Methyl-2H-tetrazol-5-yl)phenyl)diphenylphosphine oxide (**4q**)

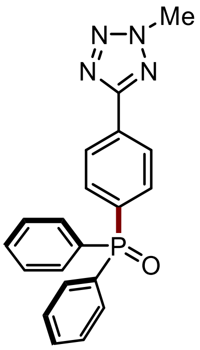

Prepared according to standard procedure B using [$RuCl_3 \cdot 3H_2O$] (10 mol %) on a 0.3 mmol scale, purification by column chromatography on silica gel (DCM/MeOH = 20/1, R_f = 0.51) yielded **4q** (38.9 mg, 36%) as a colorless solid. 175-176 °C

^{1}H NMR (400 MHz, $CDCl_3$) δ 8.27 – 8.20 (m, 2H), 7.83 – 7.77 (m, 2H), 7.72 – 7.66 (m, 4H), 7.59 – 7.54 (m, 2H), 7.51 – 7.45 (m, 4H), 4.41 (s, 3H).

^{13}C NMR (101 MHz, $CDCl_3$) δ 164.3 (C_q), 134.7 (d, *J* = 103.1 Hz) (C_q), 132.7 (d, *J* = 10.0 Hz) (CH), 132.1 (CH), 132.1 (d, *J* = 9.9 Hz) (CH), 131.1 (d, *J* = 99.6 Hz) (C_q), 130.6 (Cq), 128.6 (d, *J* = 12.2 Hz) (CH), 126.7 (d, *J* = 12.2 Hz) (CH), 39.6 (CH_3).

^{31}P NMR (162 MHz, $CDCl_3$) δ 28.61.

HRMS (ESI) m/z: $[M+H]^+$ Calcd for $C_{20}H_{18}N_4OP$ 361.1213; Found 361.1218.

IR (ATR): 3058, 1437, 1196, 1113, 1003, 844, 727, 562 cm^{-1}.

(4-(9-((3aR,4R,6R,6aR)-6-(Hydroxymethyl)-2,2-dimethyltetrahydrofuro[3,4-d][1,3]dioxol-4-yl)-9H-purin-6-yl)phenyl)diphenylphosphine oxide (**4r**)

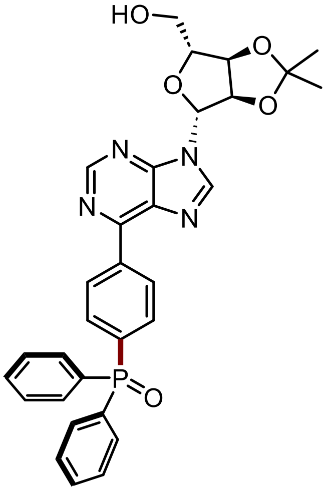

Prepared according to standard procedure B using [$RuCl_3 \cdot 3H_2O$] (10 mol %) on a 0.3 mmol scale, purification by column chromatography on silica gel (DCM/MeOH = 20/1, R_f = 0.40) yielded **4r** (47.6 mg, 28%) as a colorless sticky oil.

^{1}H NMR (300 MHz, $CDCl_3$) δ 8.99 (s, 1H), 8.83 (dd, *J* = 8.4, 2.5 Hz, 2H), 8.25 (s, 1H), 7.86 (dd, *J* = 11.6, 8.4 Hz, 2H), 7.73 – 7.66 (m, 4H), 7.58 – 7.52 (m, 2H), 7.50 – 7.44 (m, 4H), 6.02 (d, *J* = 4.6 Hz, 1H), 5.63 (d, *J* = 8.3 Hz, 1H), 5.30 – 5.23 (m, 1H), 5.13 (dd, *J* = 6.0, 1.2 Hz, 1H), 4.58 – 4.53 (m, 1H), 3.98 (dd, *J* = 12.6, 1.4 Hz, 1H), 3.88 – 3.77 (m, 1H), 1.66 (s, 3H), 1.38 (s, 3H).

^{13}C NMR (75 MHz, $CDCl_3$) δ 154.5 (d, *J* = 1.0 Hz) (C_q), 151.8 (CH), 151.4 (C_q), 144.4 (CH), 138.4 (d,

J = 2.9 Hz) (C_q), 135.3 (d, *J* = 102.3 Hz) (C_q), 132.9 (C_q), 132.4 (d, *J* = 10.0 Hz) (CH), 132.1 (d, *J* = 3.9 Hz) (CH), 132.1 (d, *J* = 104.5 Hz) (C_q), 132.0 (d, *J* = 3.3 Hz) (CH), 129.7 (d, *J* = 12.0 Hz) (CH), 128.6 (d, *J* = 12.2 Hz) (CH), 114.2 (C_q), 93.9 (CH), 86.3 (CH), 83.1 (CH), 81.6 (CH), 63.2 (CH_2), 27.6 (CH_3), 25.2 (CH_3).

^{31}P NMR (121 MHz, $CDCl_3$) δ 28.98.

HRMS (ESI) m/z: $[M+H]^+$ Calcd for $C_{31}H_{30}N_4O_5P$ 569.1948; Found 569.1959.

IR (ATR): 2938, 1581, 1437, 1173, 1083, 848, 718, 532 cm^{-1}.

9. NMR Spectroscopic Data

(2-(9-Isopropyl-9H-purin-6-yl)phenyl)diphenylphosphine oxide (**3a**)

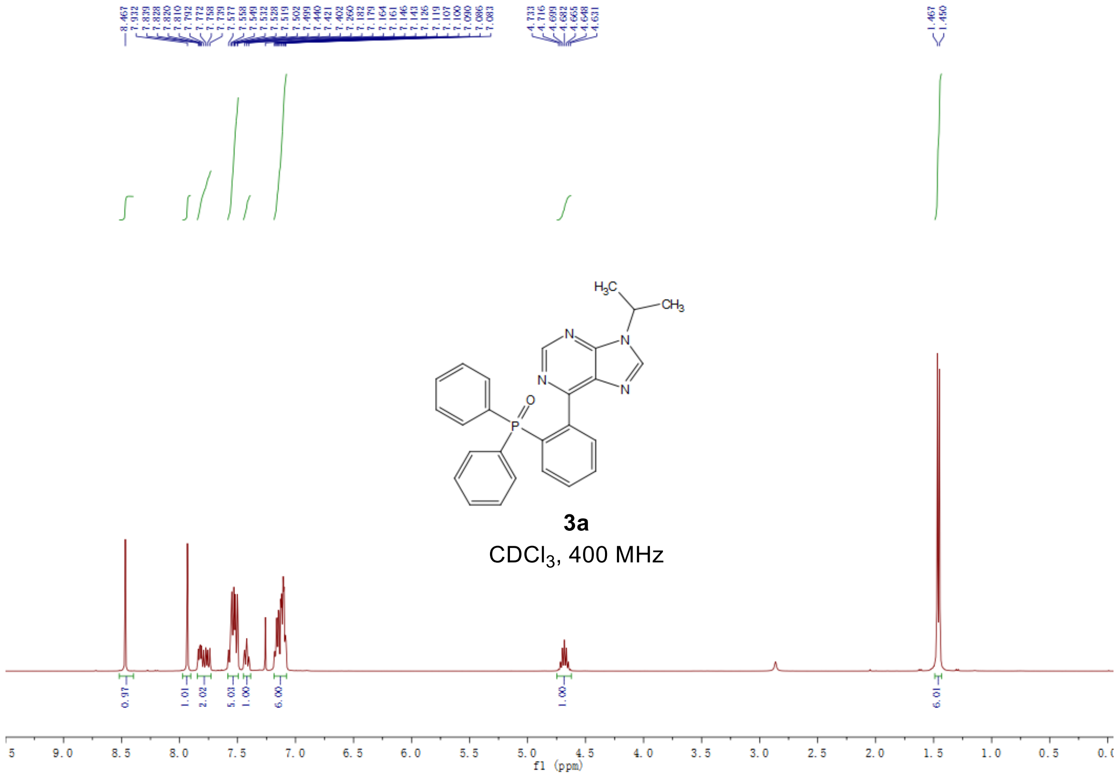

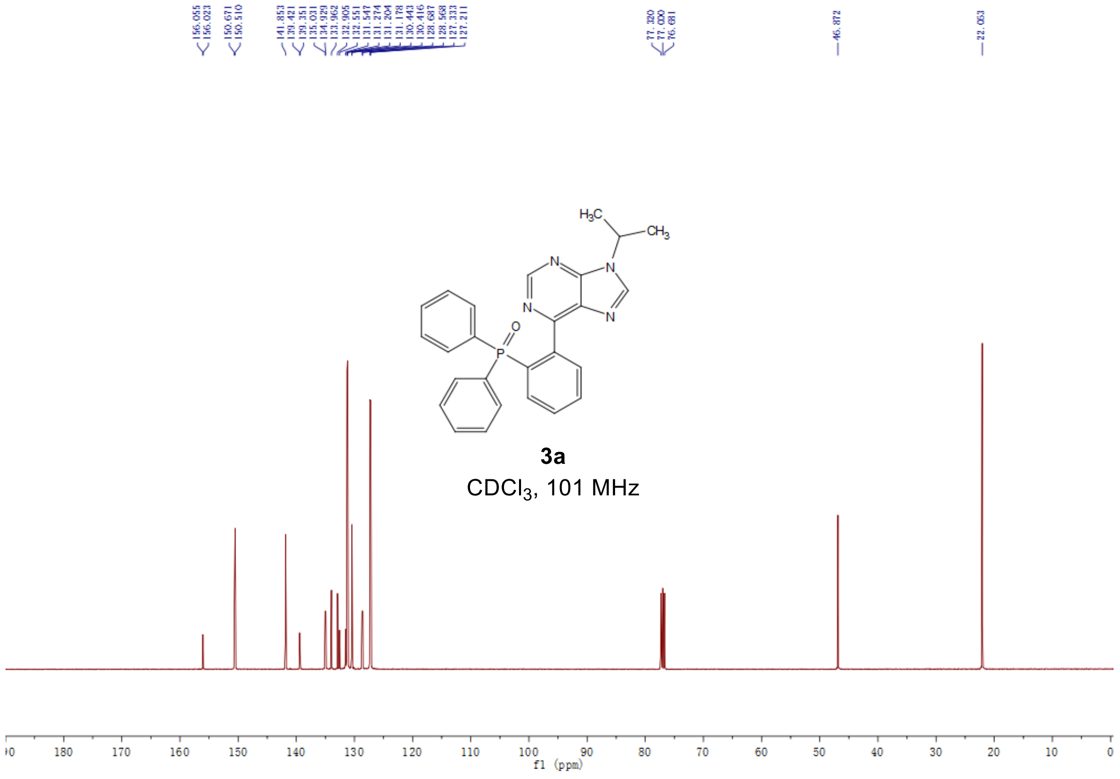

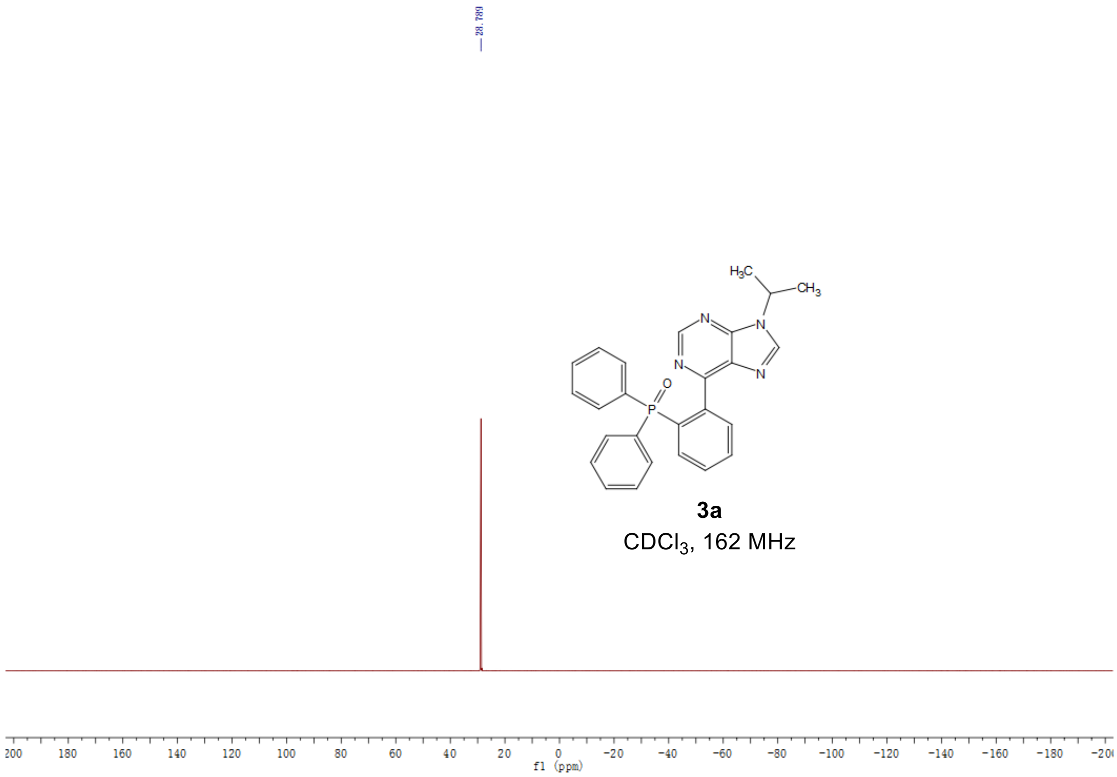

(2-(9-Methyl-9H-purin-6-yl)phenyl)diphenylphosphine oxide (**3b**)

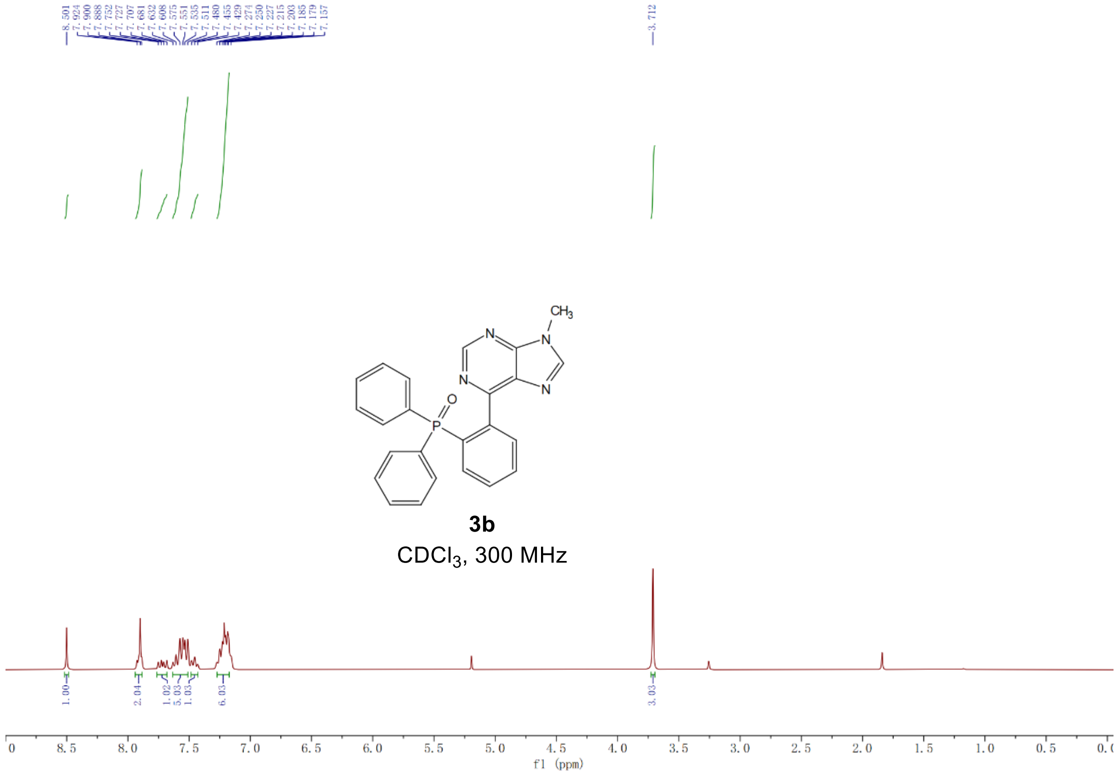

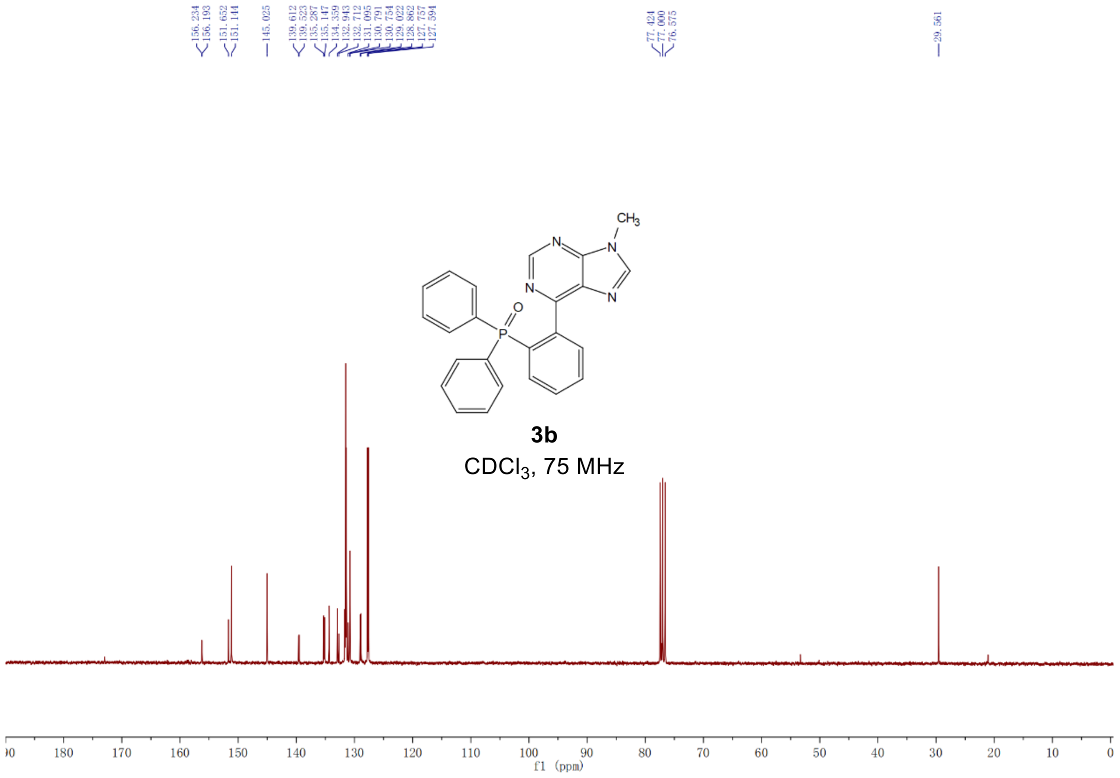
156.234
156.193
151.652
151.144
145.025
139.612
139.523
135.287
135.147
134.359
132.943
132.712
131.065
130.791
130.754
129.022
128.862
127.757
127.594
77.424
77.000
76.575
29.561
CH3
N
O
P
3b
CDCl3, 75 MHz
f1 (ppm)

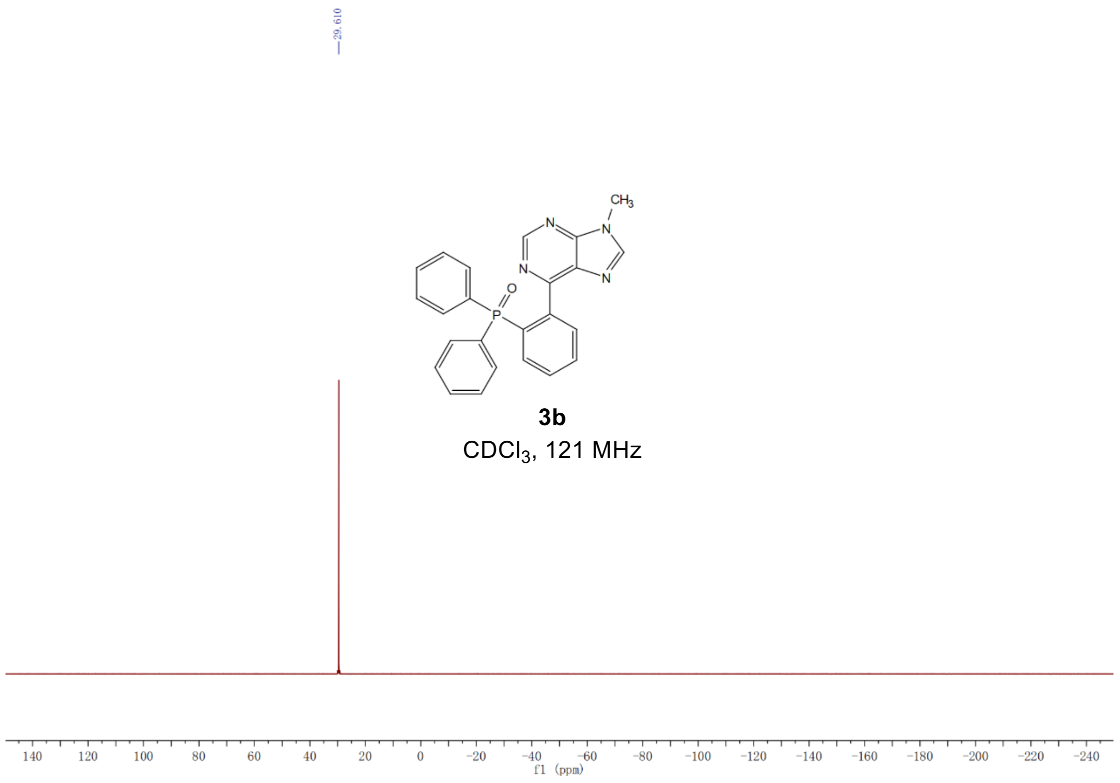
29.610
CH3
N
O
P
3b
CDCl3, 121 MHz
f1 (ppm)

(2-(9-Benzyl-9H-purin-6-yl)phenyl)diphenylphosphine oxide (**3c**)

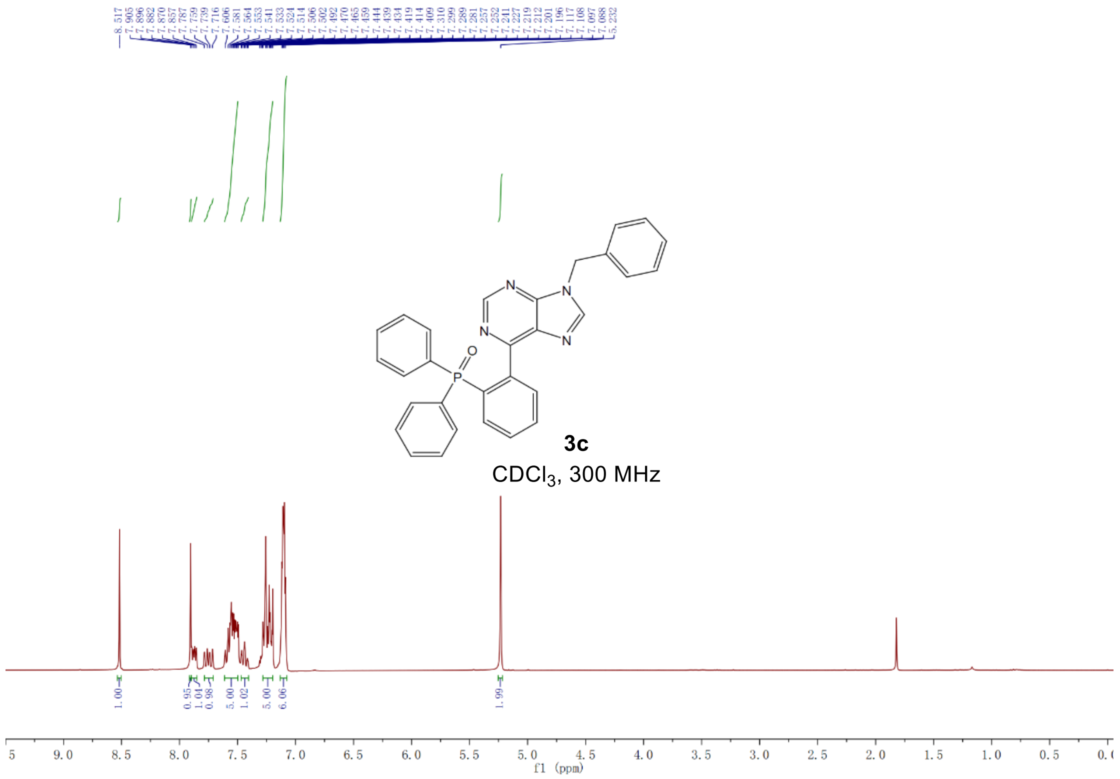

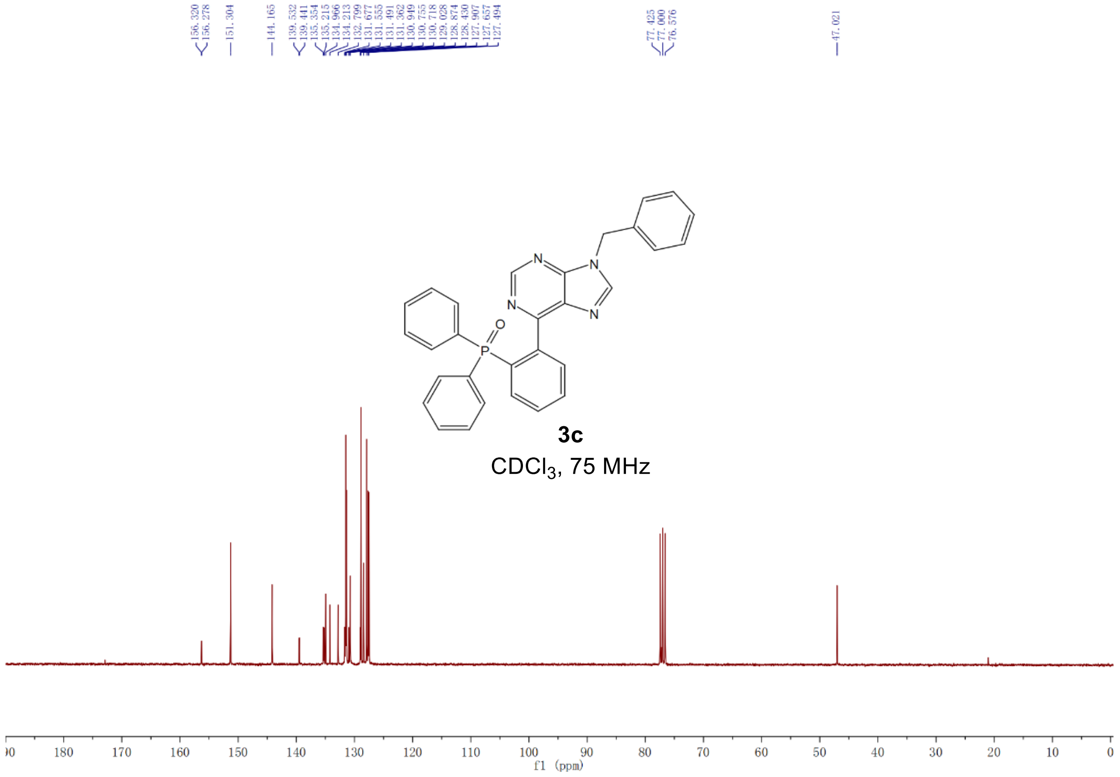

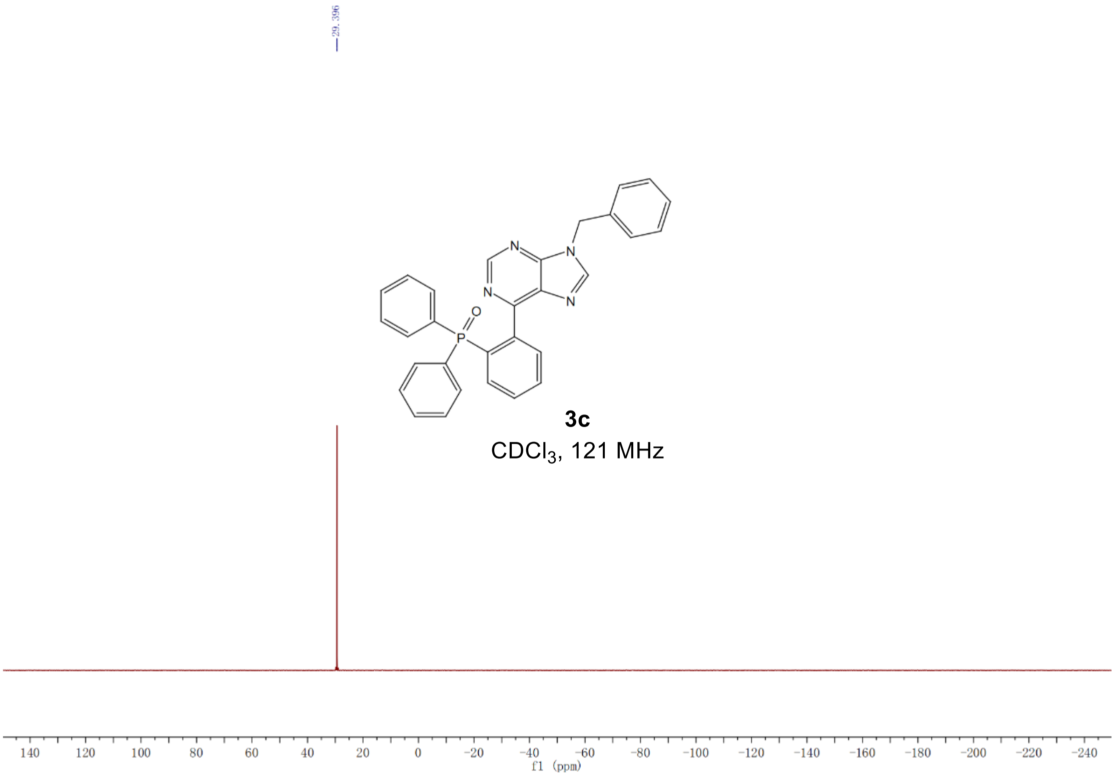

(2R,3R,4R,5R)-2-(Acetoxymethyl)-5-(6-(2-(diphenylphosphoryl)phenyl)-9H-purin-9-yl)tetrahydrofuran-3,4-diyl diacetate (**3d**)

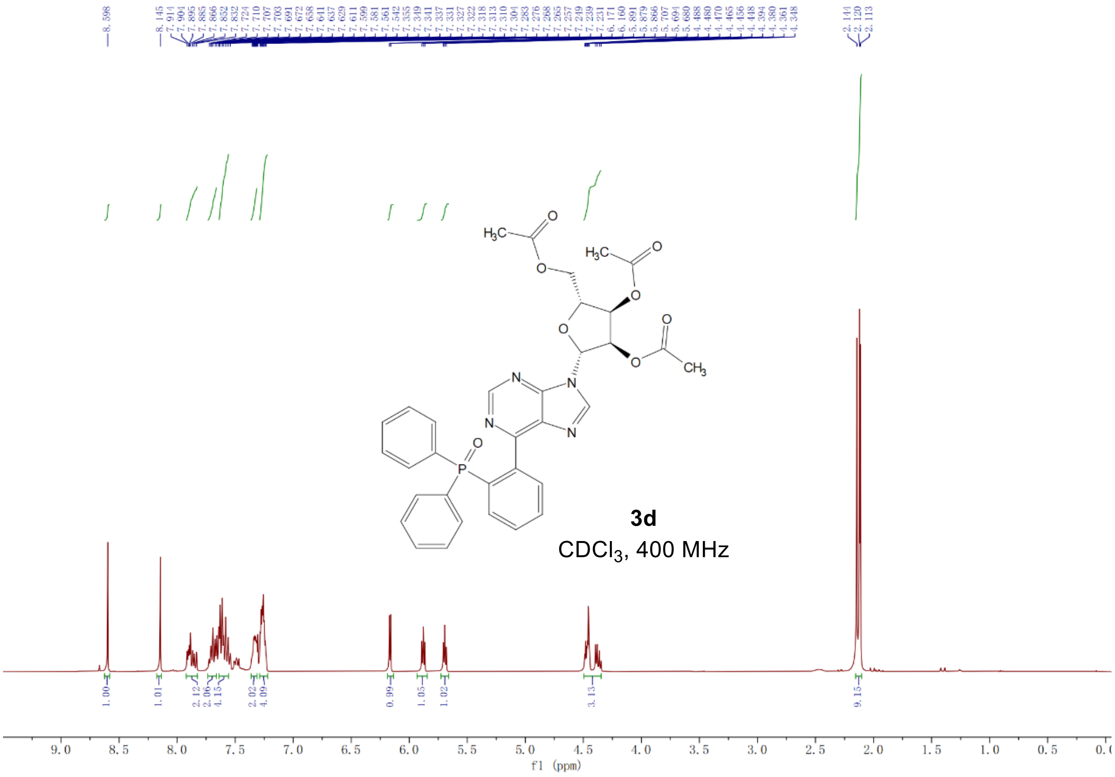

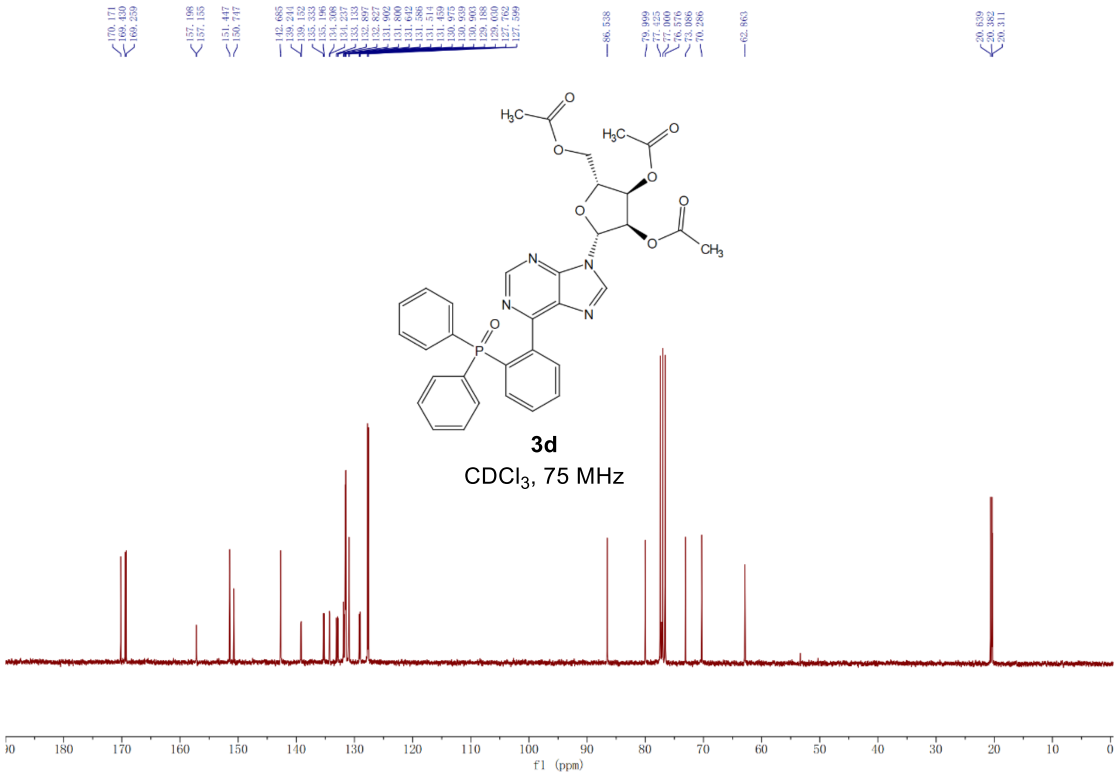
170.171
169.430
169.259
157.198
157.155
151.447
150.747
142.685
139.244
139.152
135.333
135.196
134.308
134.237
133.133
132.897
132.827
131.902
131.800
131.642
131.586
131.514
131.459
130.975
130.939
130.903
129.188
129.030
127.762
127.599
86.538
79.999
77.425
77.000
76.576
73.086
70.286
62.863
20.639
20.382
20.311
H_3C
O
N
P
CH_3
3d
$CDCl_3$, 75 MHz
180 170 160 150 140 130 120 110 100 90 80 70 60 50 40 30 20 10 0
f1 (ppm)

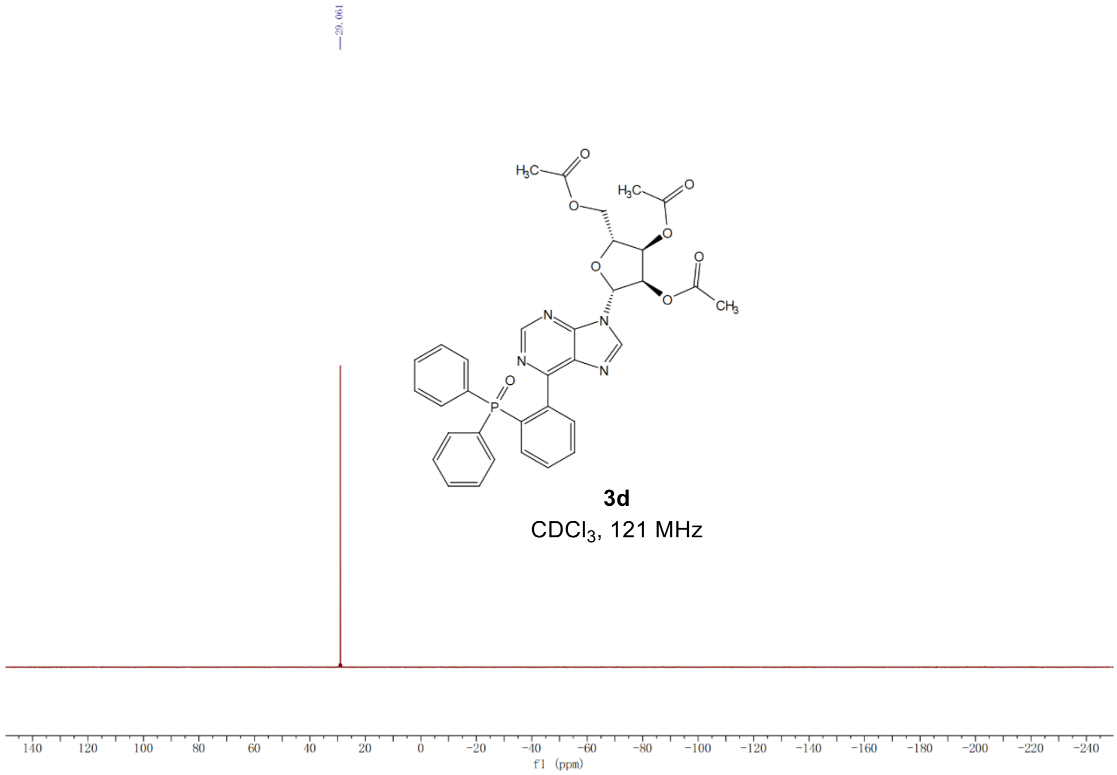
29.061
H_3C
O
N
P
CH_3
3d
$CDCl_3$, 121 MHz
140 120 100 80 60 40 20 0 −20 −40 −60 −80 −100 −120 −140 −160 −180 −200 −220 −240
f1 (ppm)

(4-Fluoro-2-(9-isopropyl-9H-purin-6-yl)phenyl)diphenylphosphine oxide (**3e**)

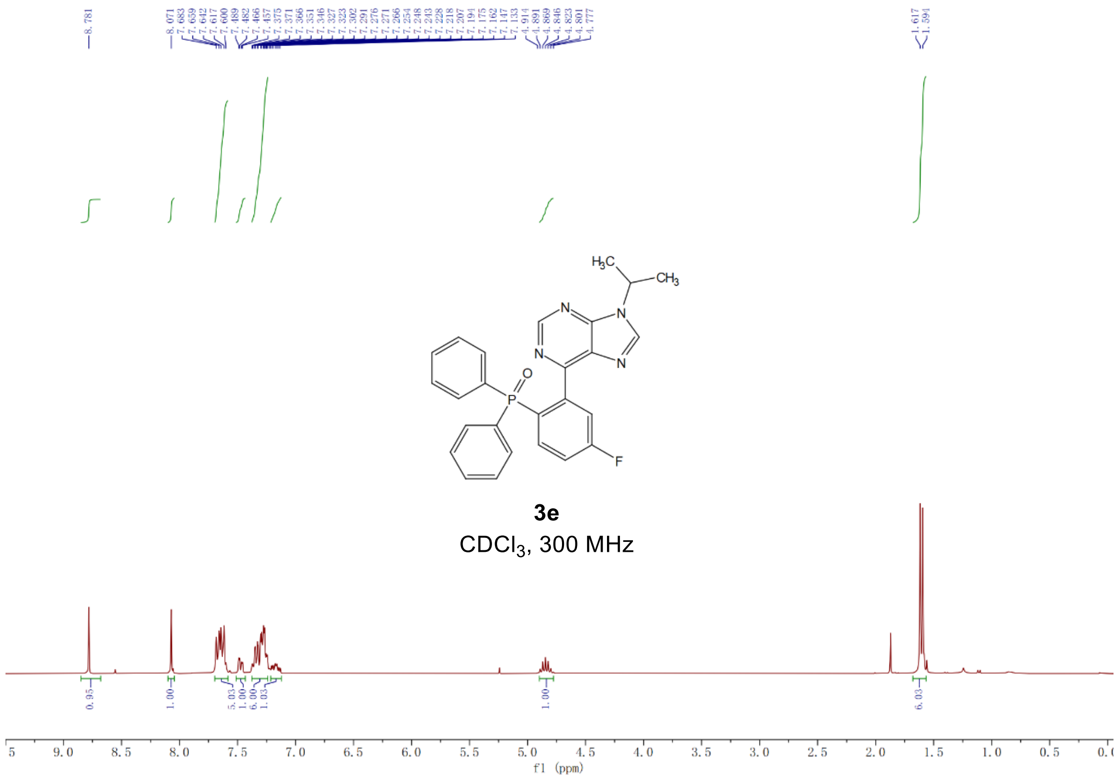

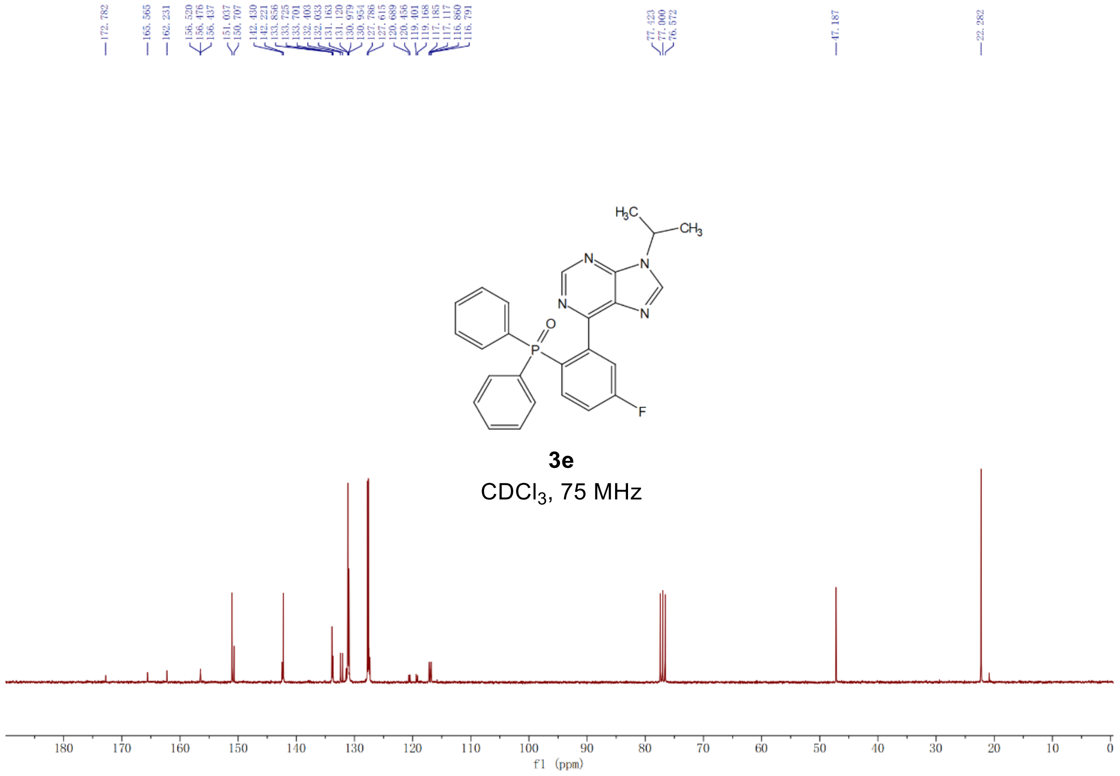

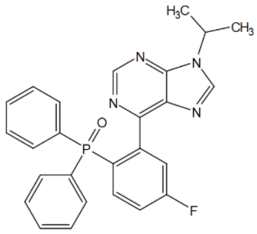

3e
$CDCl_3$, 121 MHz

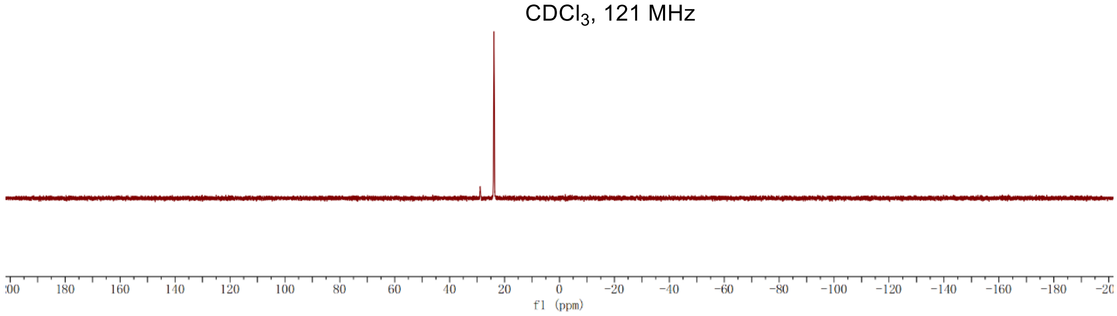

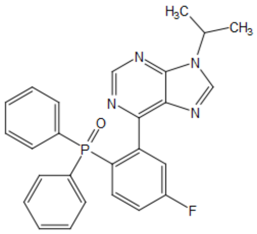

3e
$CDCl_3$, 282 MHz

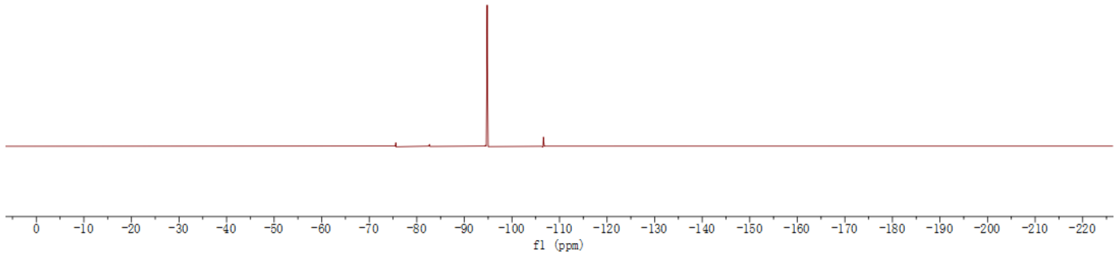

(2-(9-Isopropyl-9H-purin-6-yl)-4-methylphenyl)diphenylphosphine oxide (**3f**)

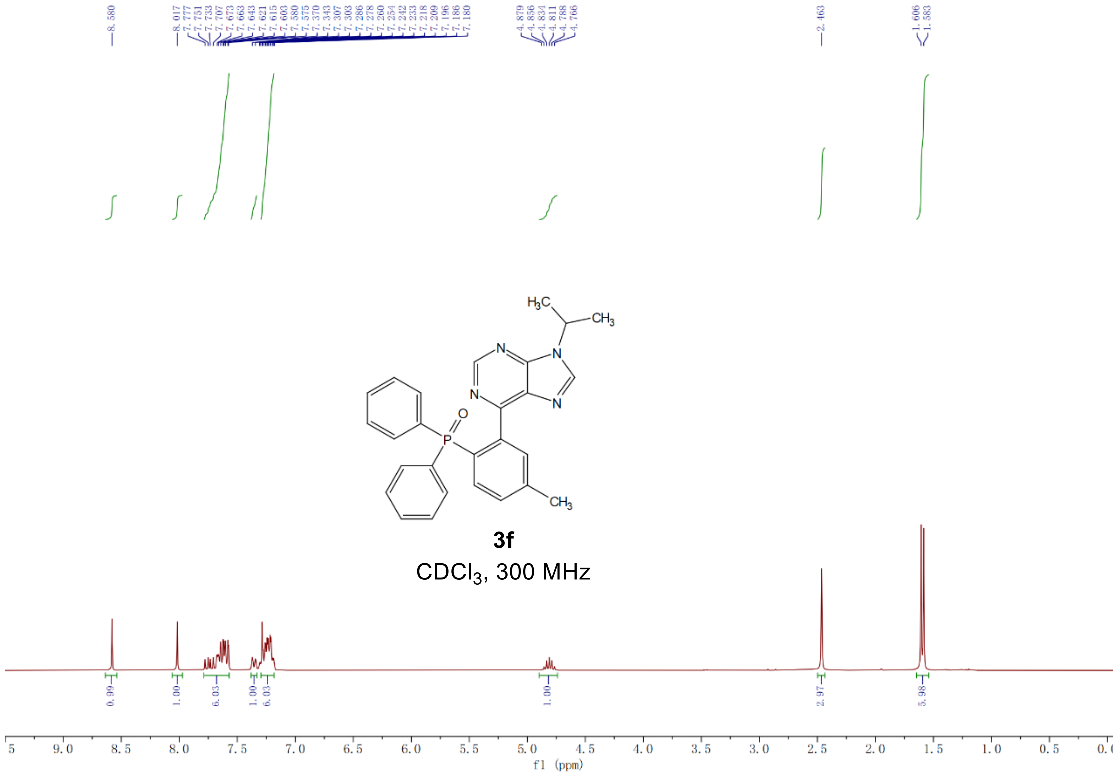

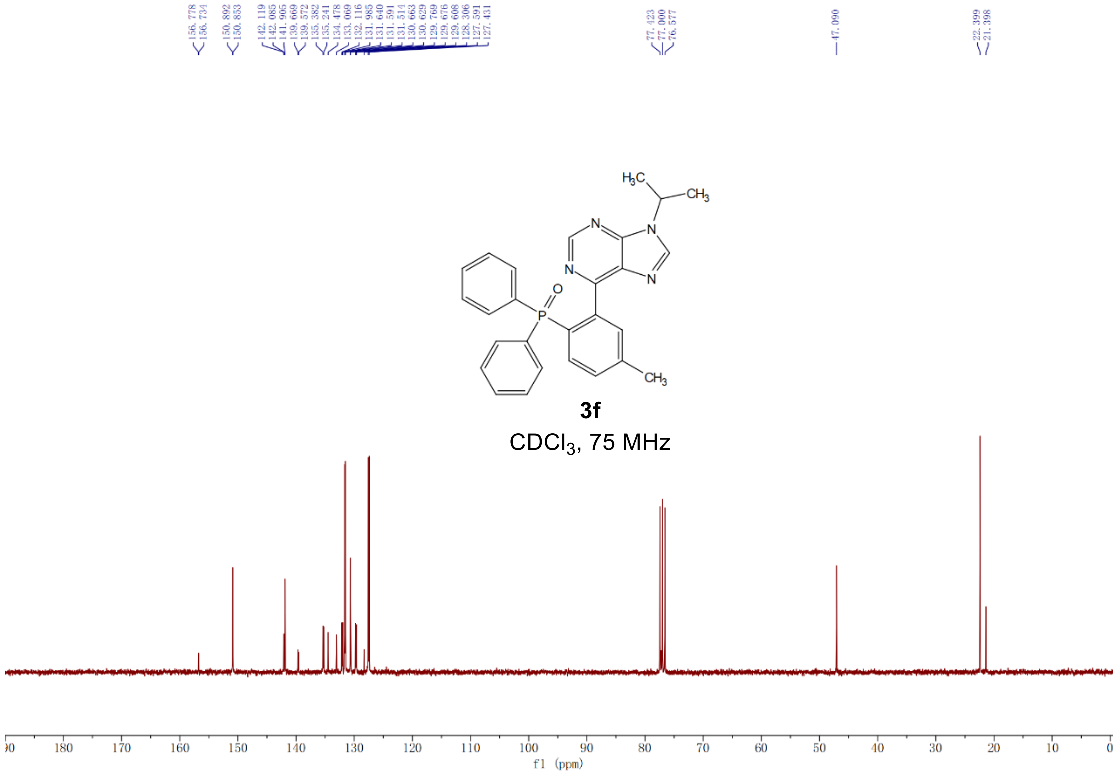

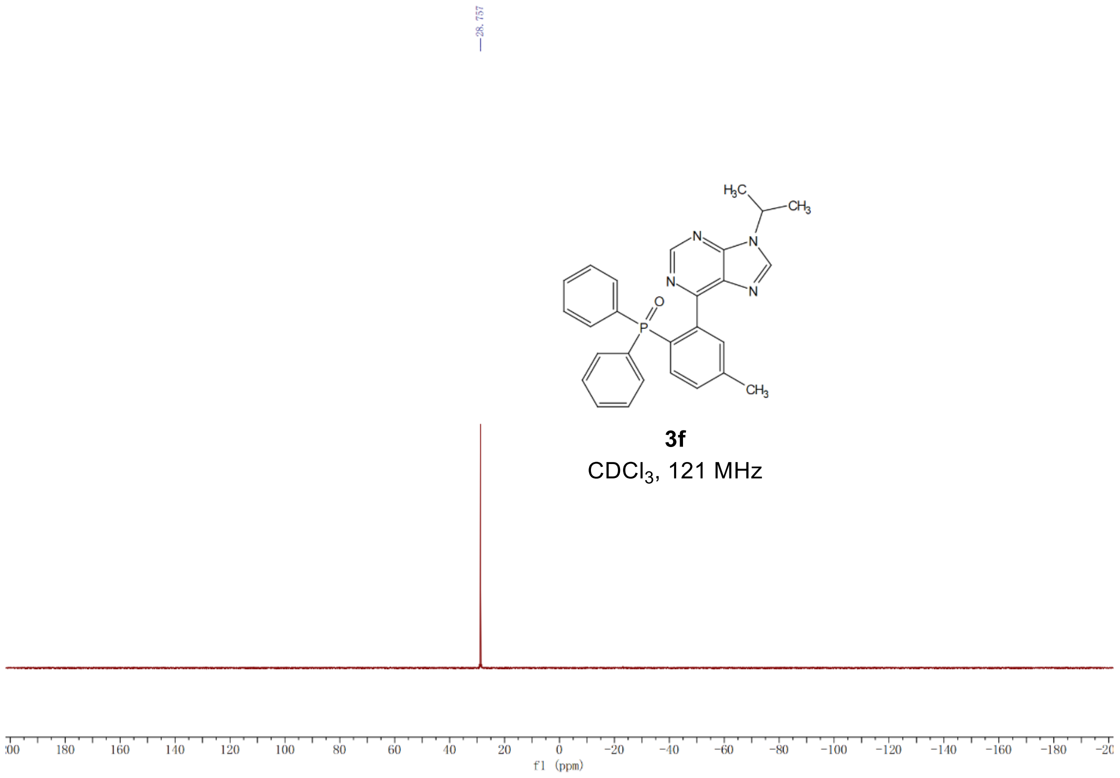

(2,5-Bis(3-methoxypyridin-2-yl)phenyl)diphenylphosphine oxide (**3g**)

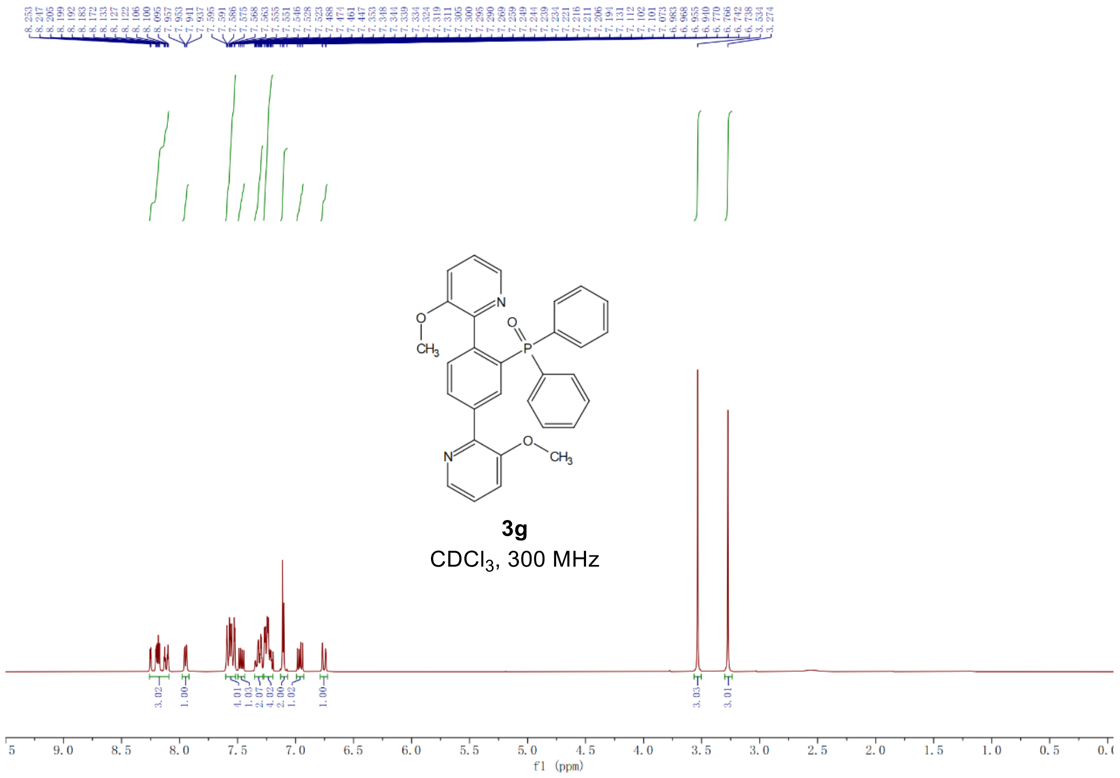

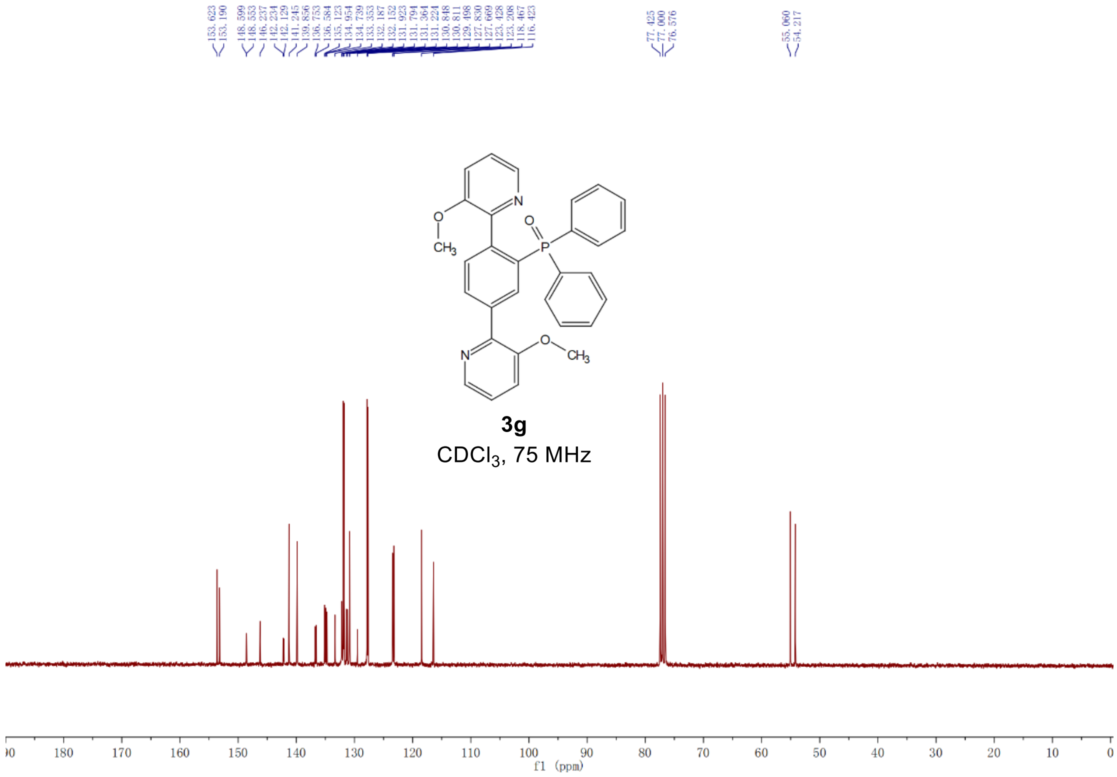
3g
$CDCl_3$, 75 MHz
f1 (ppm)

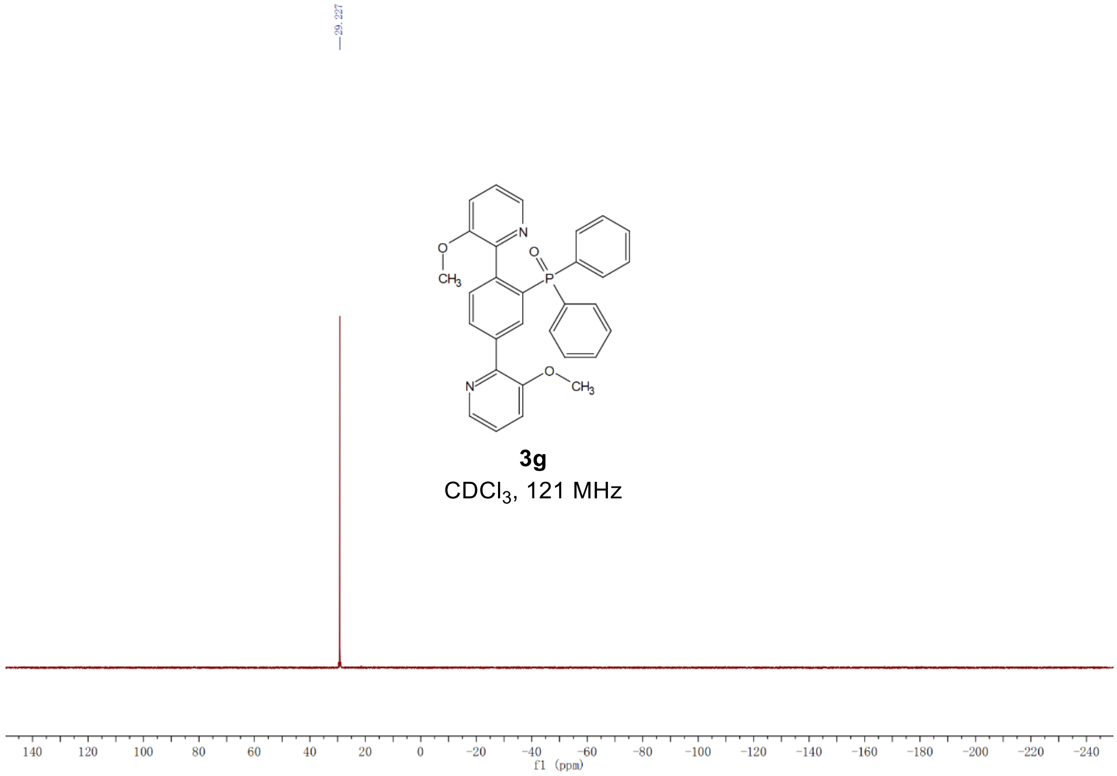
29.227
3g
$CDCl_3$, 121 MHz
f1 (ppm)

(2-(4,4-Dimethyl-4,5-dihydrooxazol-2-yl)phenyl)diphenylphosphine oxide (**3h**)

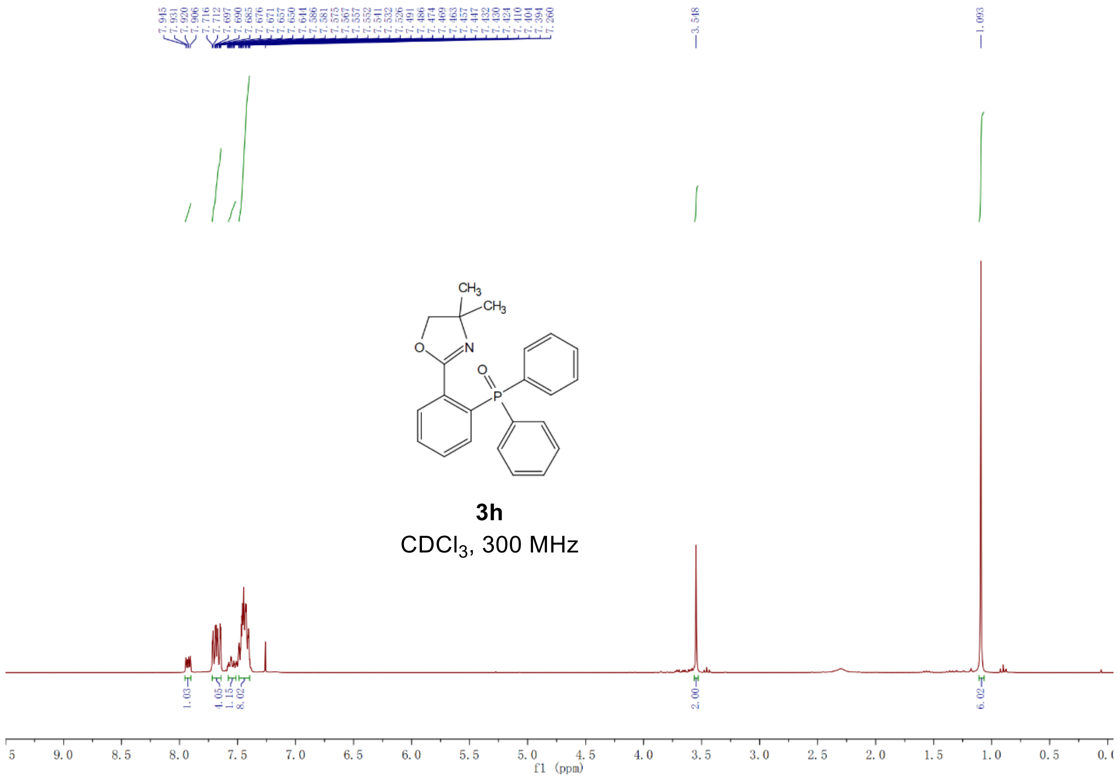

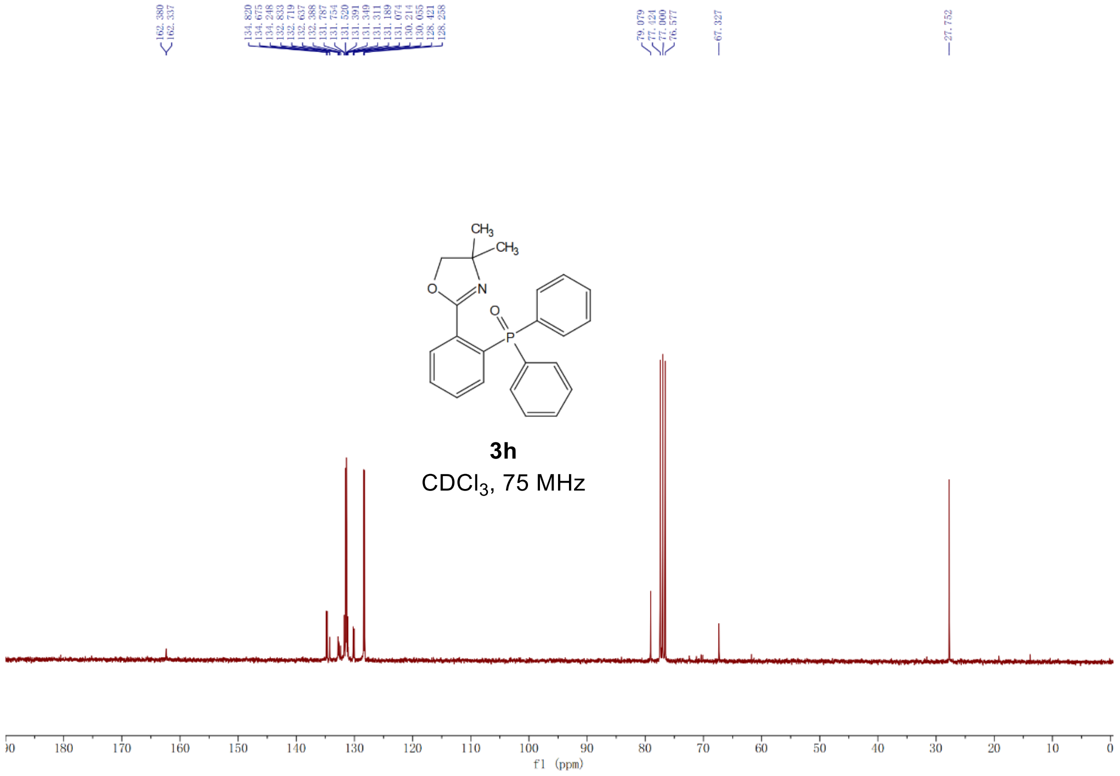

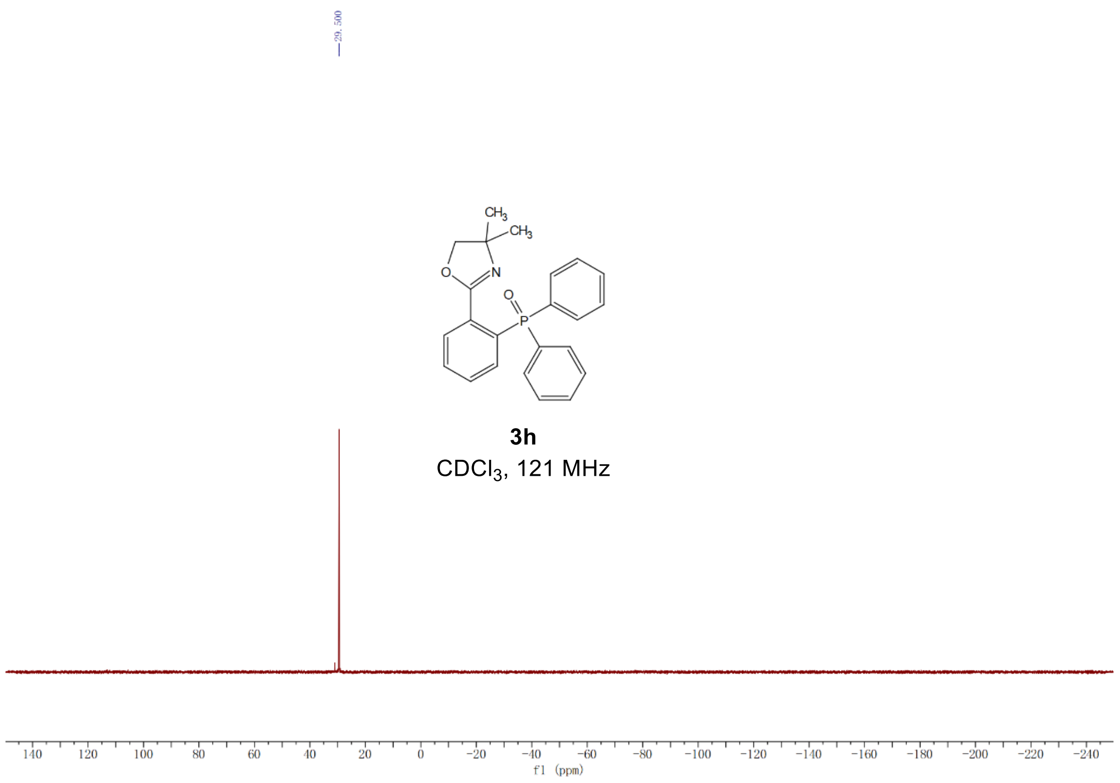

(E)-(2-(1-(Methoxyimino)ethyl)phenyl)diphenylphosphine oxide (3**i**)

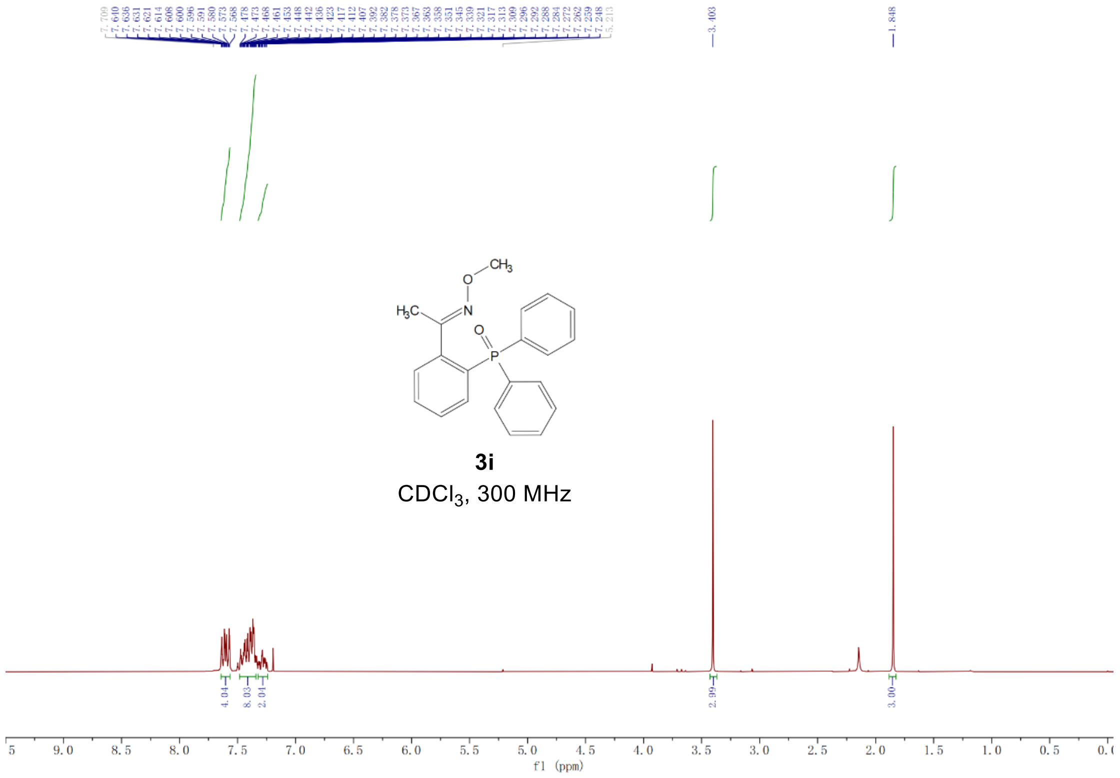

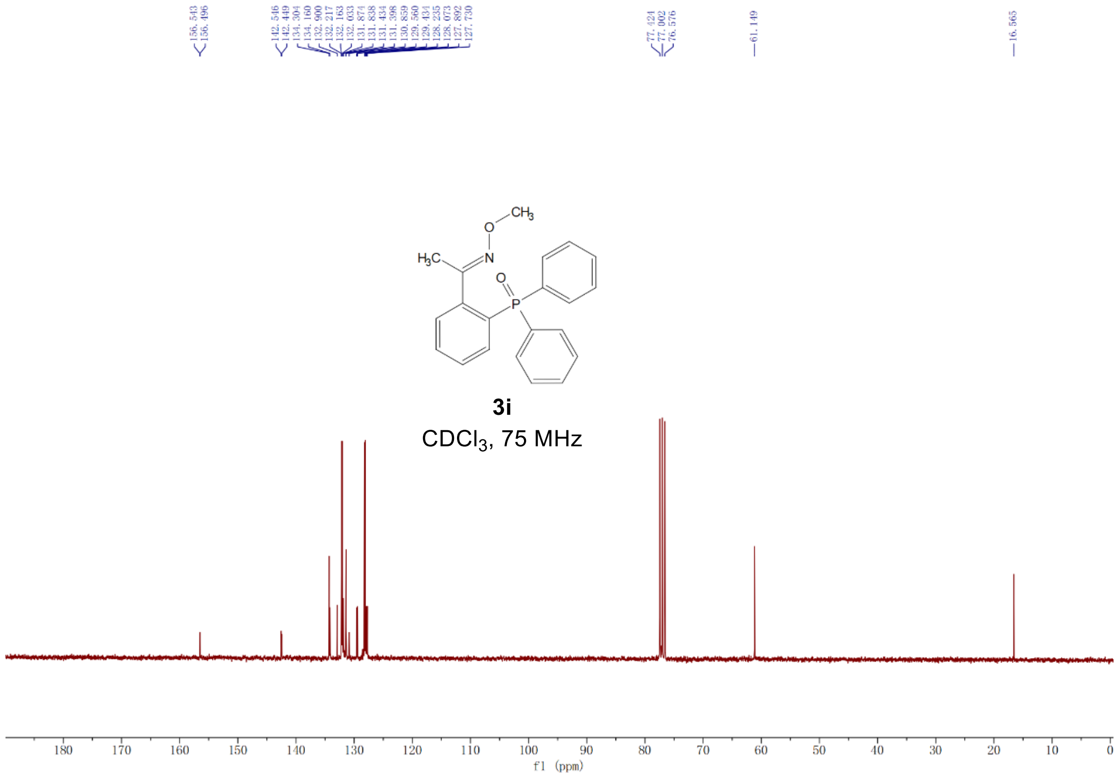

3i

$CDCl_3$, 75 MHz

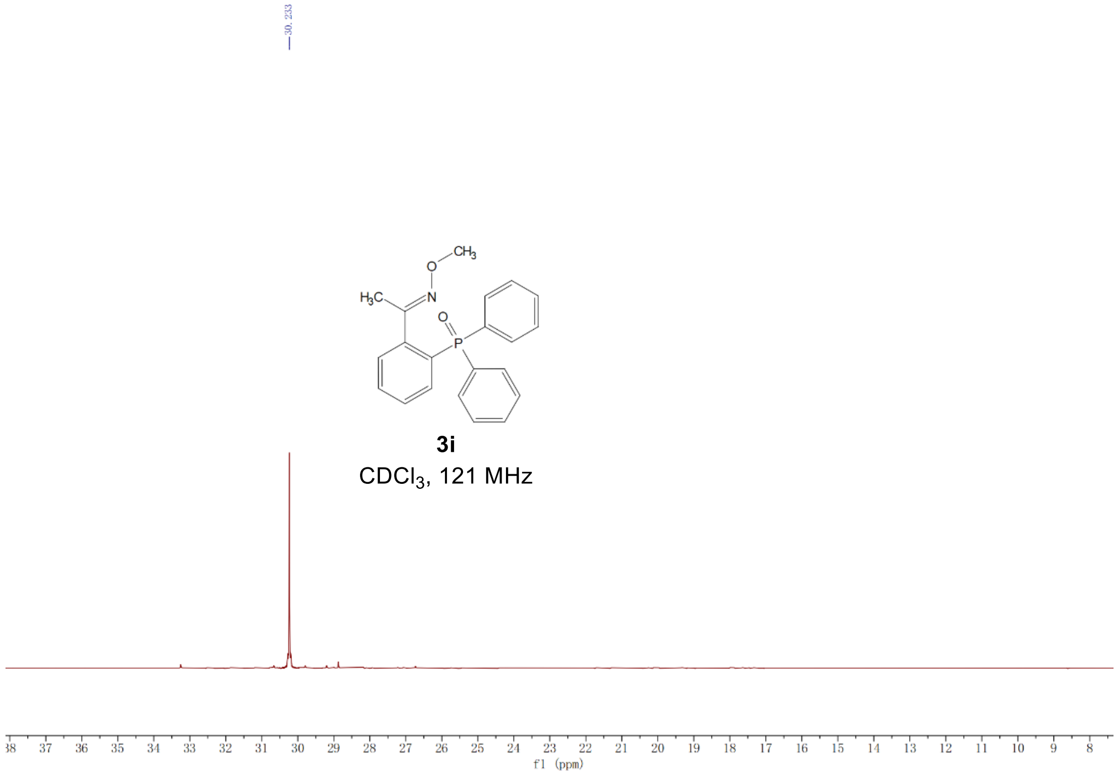

3i

$CDCl_3$, 121 MHz

Diphenyl(2-(pyrazin-2-yl)phenyl)phosphine oxide (**3j**)

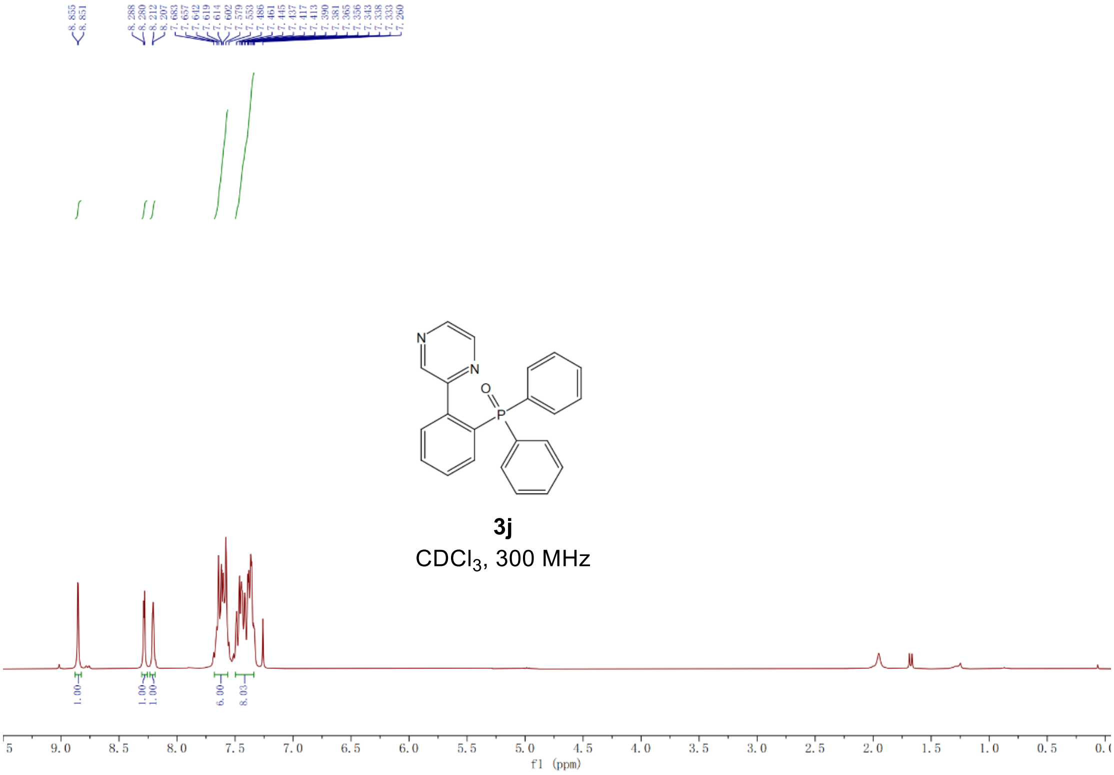

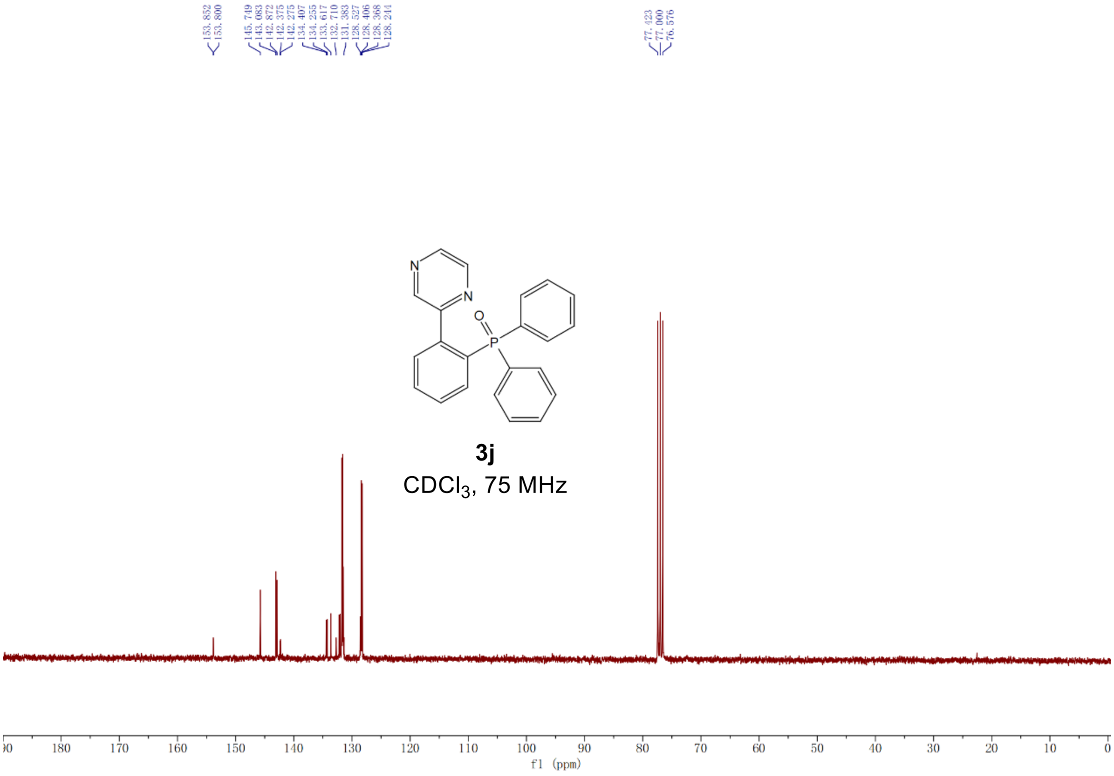

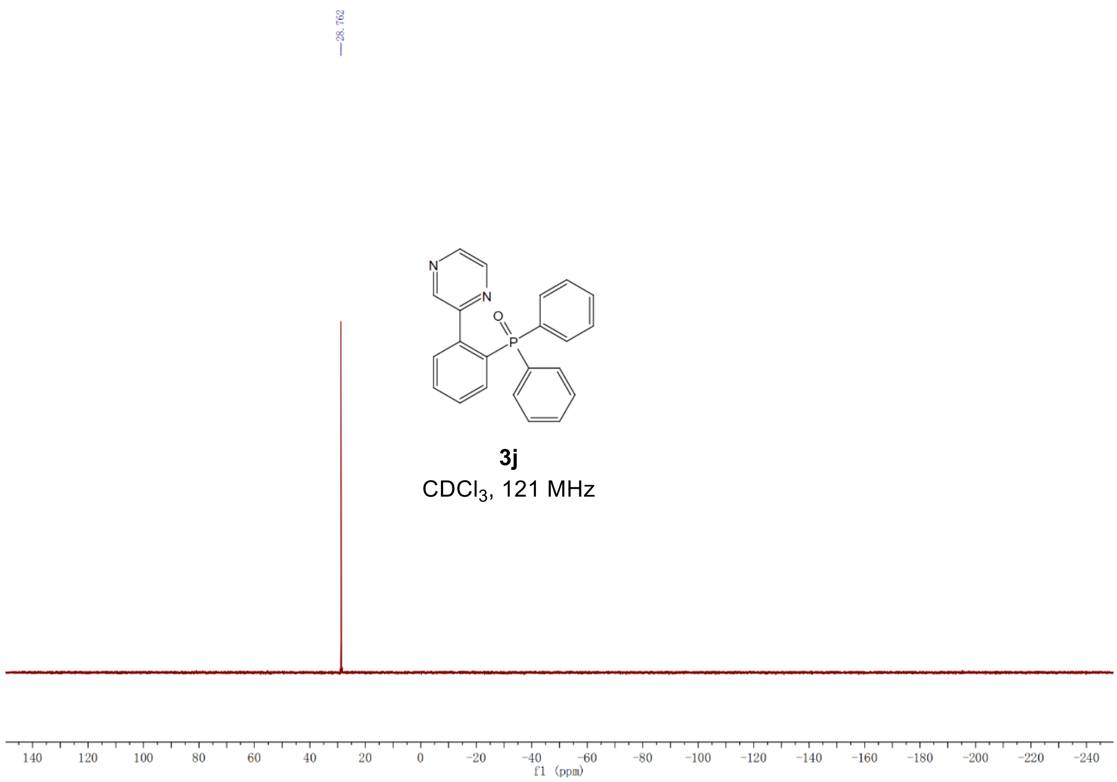

Diphenyl(2-(3-phenylpyrazin-2-yl)phenyl)phosphine oxide (**3k**)

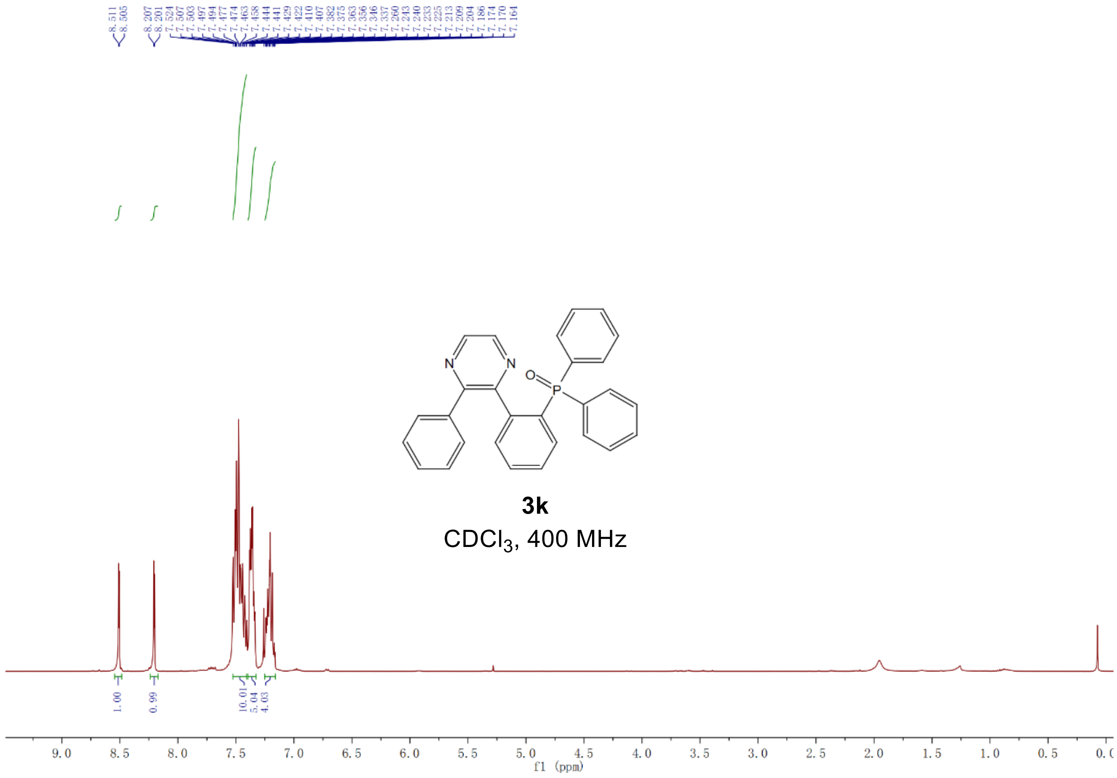

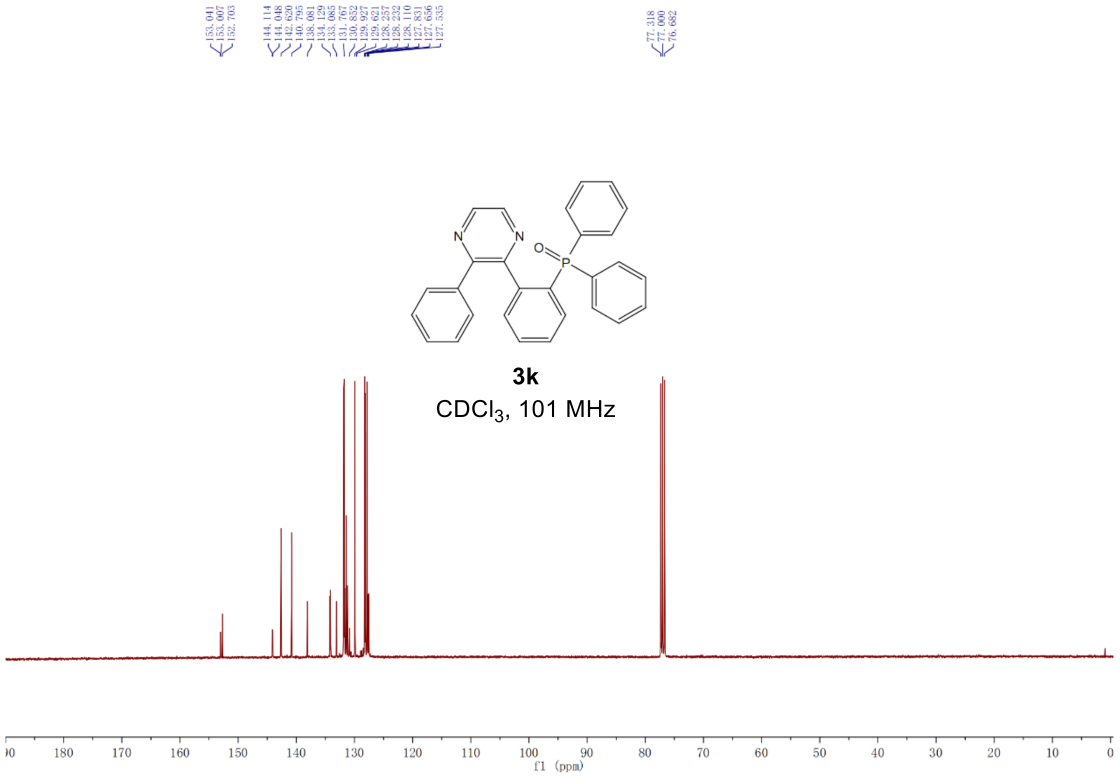
153.041
153.007
152.703
144.114
144.048
142.620
140.795
138.081
134.129
133.085
131.767
130.852
129.927
129.621
128.257
128.232
128.110
127.831
127.656
127.335
77.318
77.000
76.682
N
N
O
P
3k
CDCl3, 101 MHz
f1 (ppm)

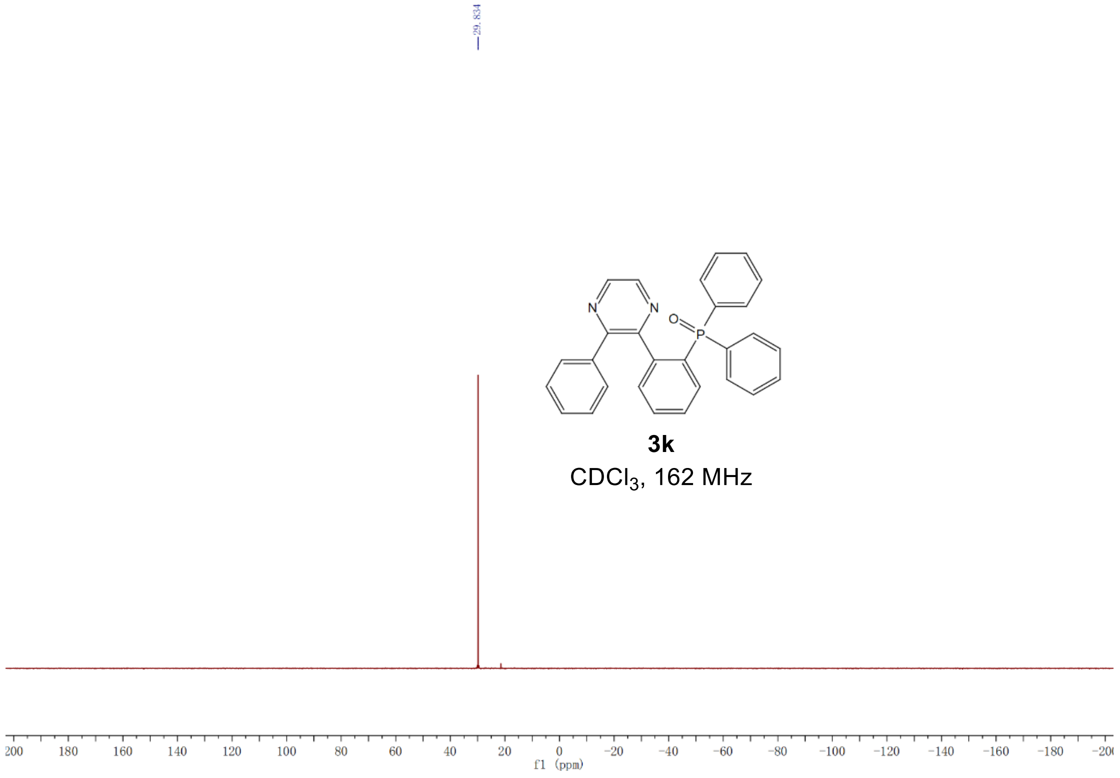
29.834
N
N
O
P
3k
CDCl3, 162 MHz
f1 (ppm)

Diphenyl(2-(pyridin-2-yl)phenyl)phosphine oxide (**3l**)

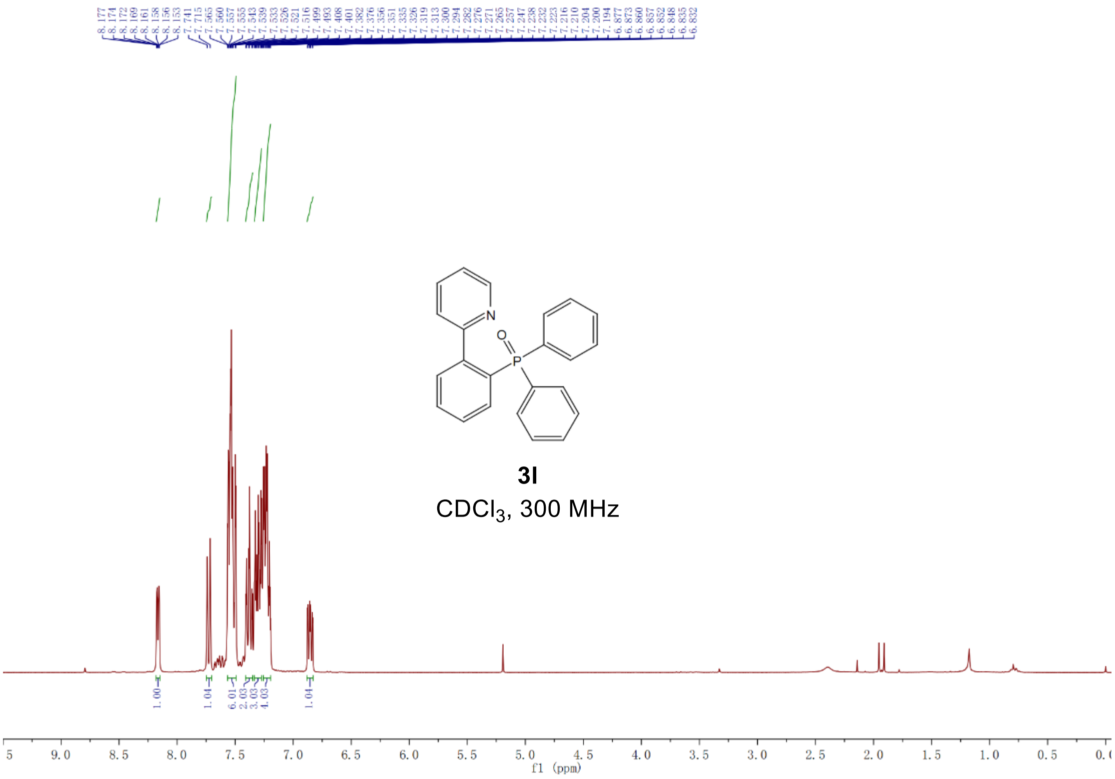

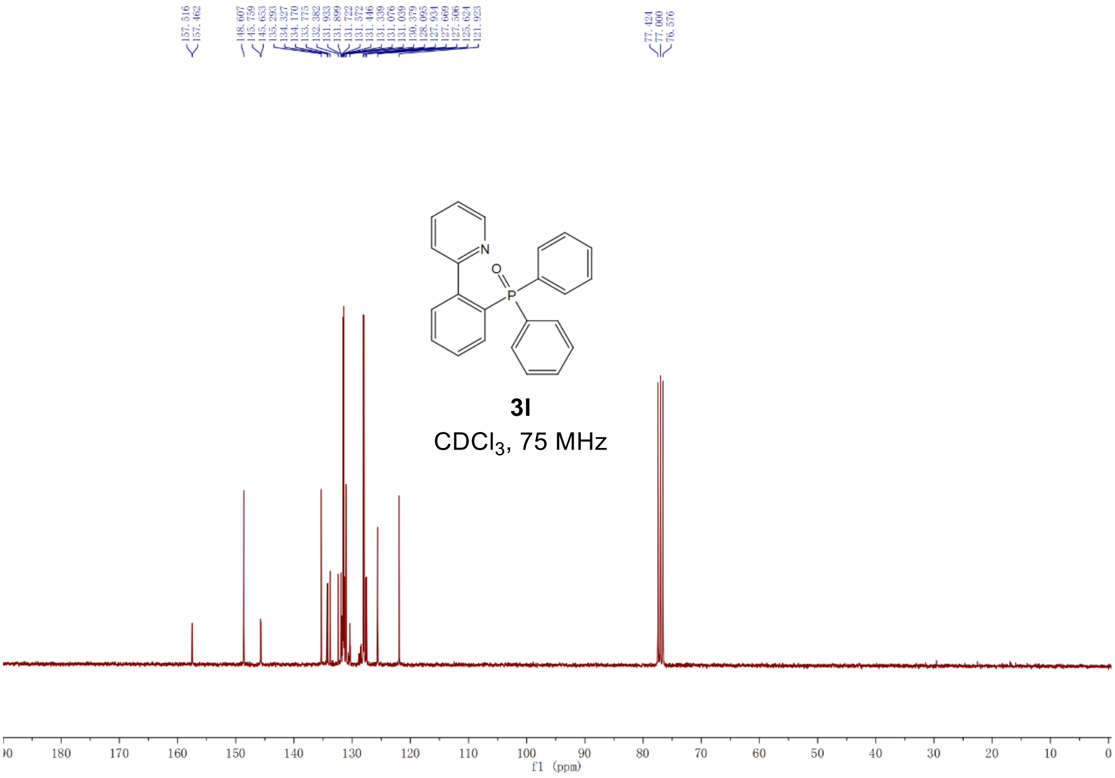

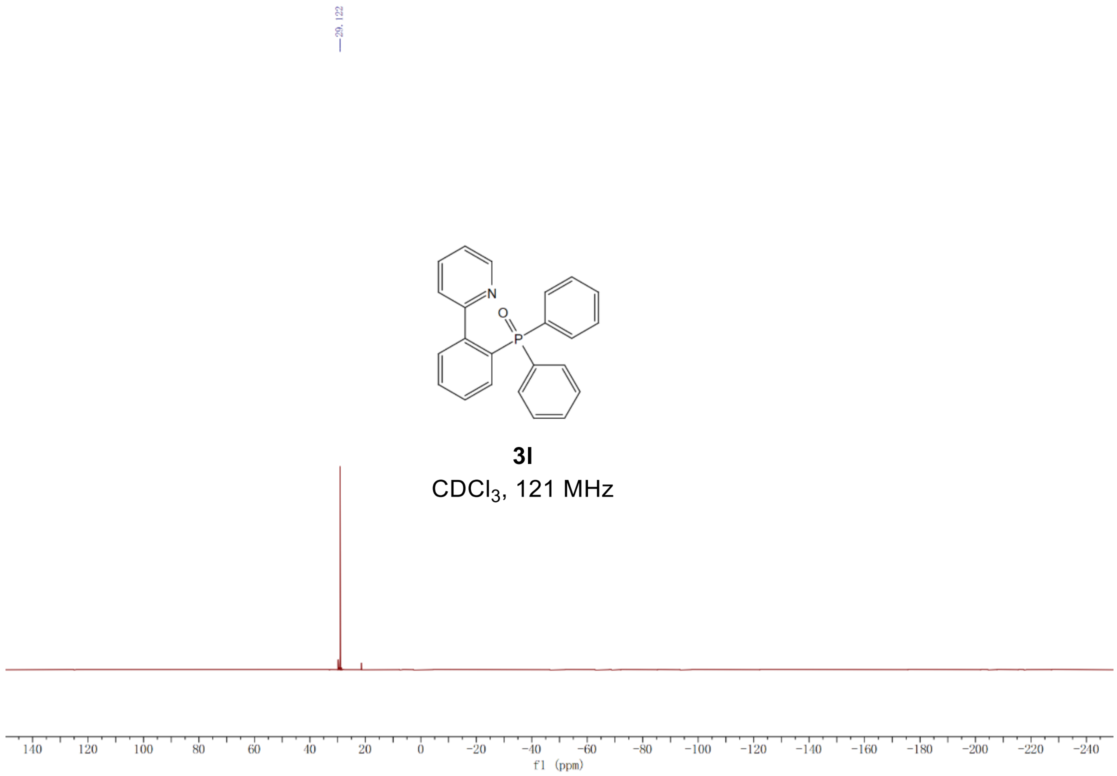

Diphenyl(2-(pyridin-2-yl)thiophen-3-yl)phosphine oxide (**3m**)

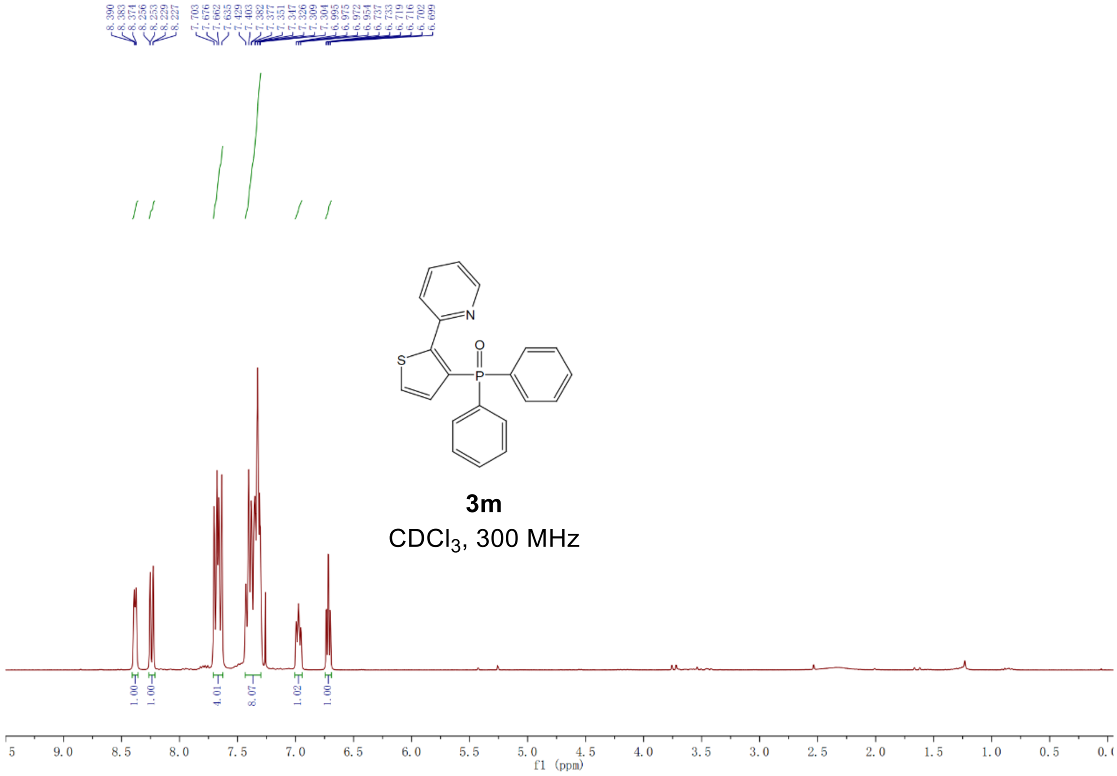

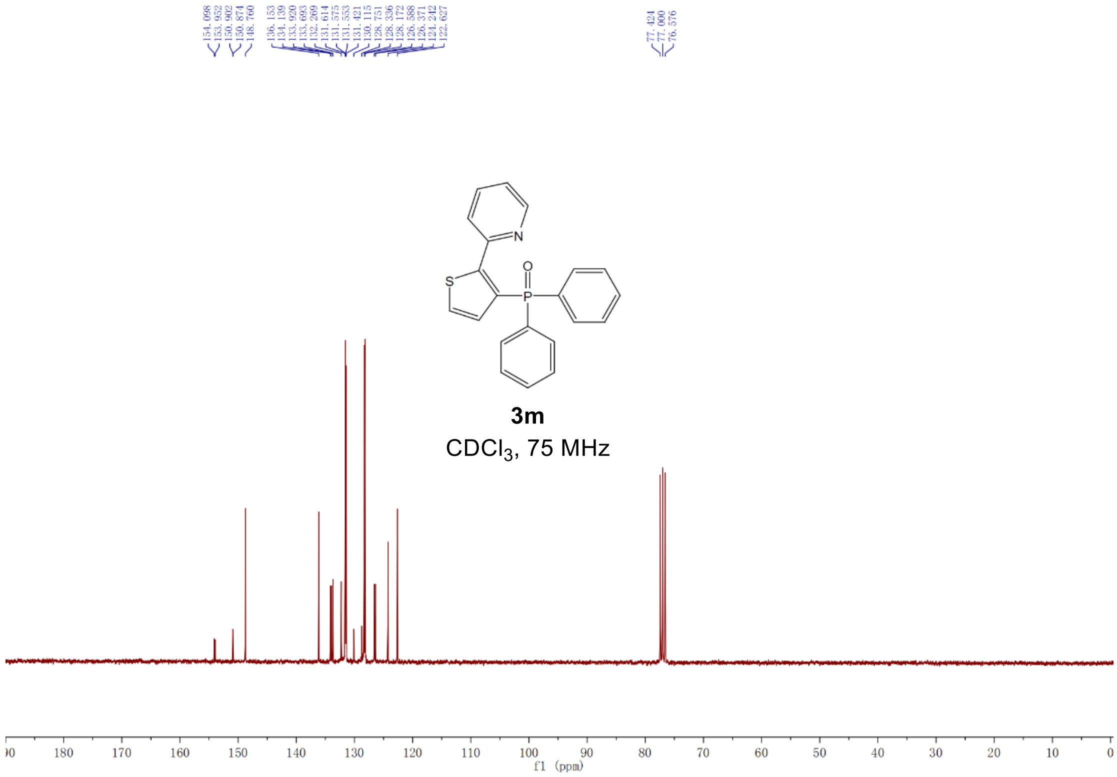
154.098
153.962
150.902
150.874
148.760
136.153
134.139
133.920
133.693
132.269
131.614
131.575
131.553
131.421
130.115
128.751
128.336
128.172
126.588
126.371
124.242
122.627
77.424
77.000
76.576
3m
CDCl3, 75 MHz
f1 (ppm)

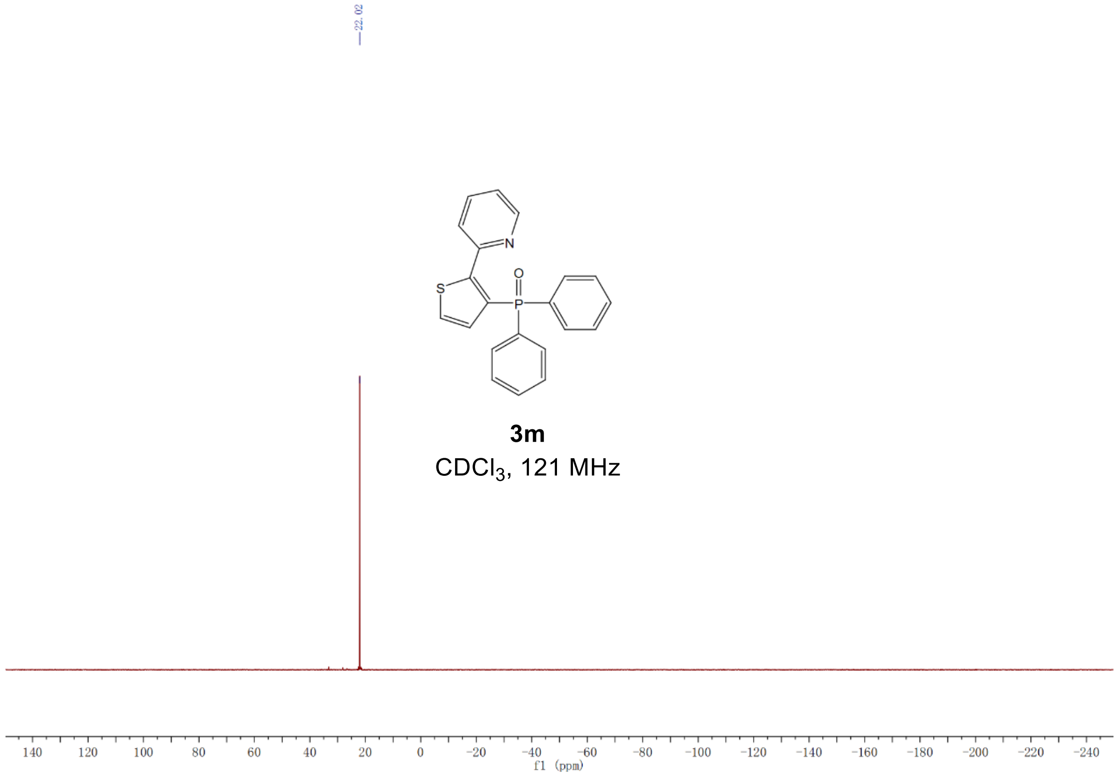
22.02
3m
CDCl3, 121 MHz
f1 (ppm)

(2-(1-methyl-1H-benzo[d]imidazol-2-yl)phenyl)diphenylphosphine oxide (**3n**)

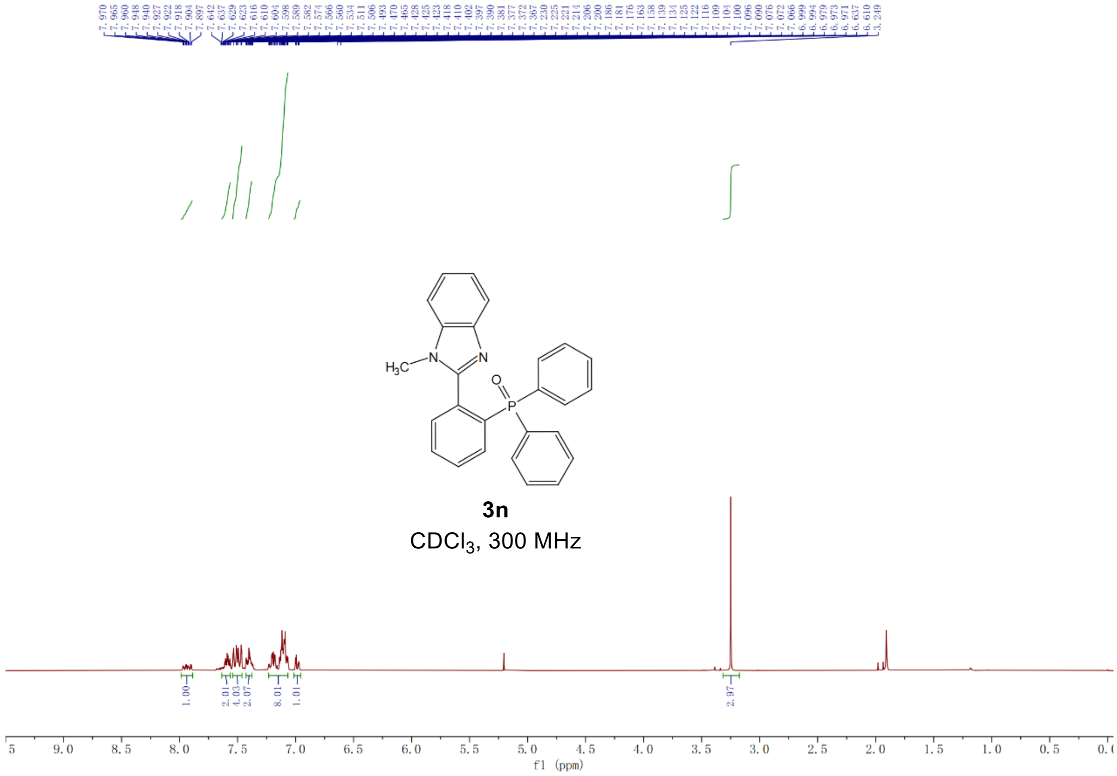

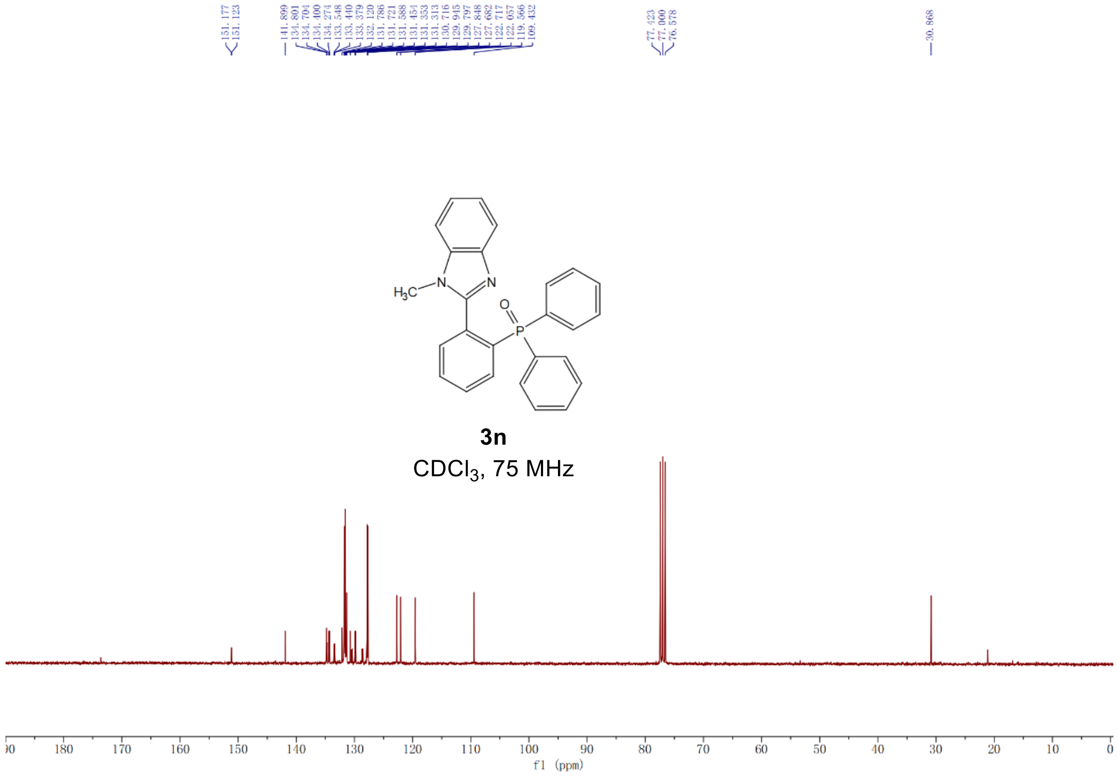

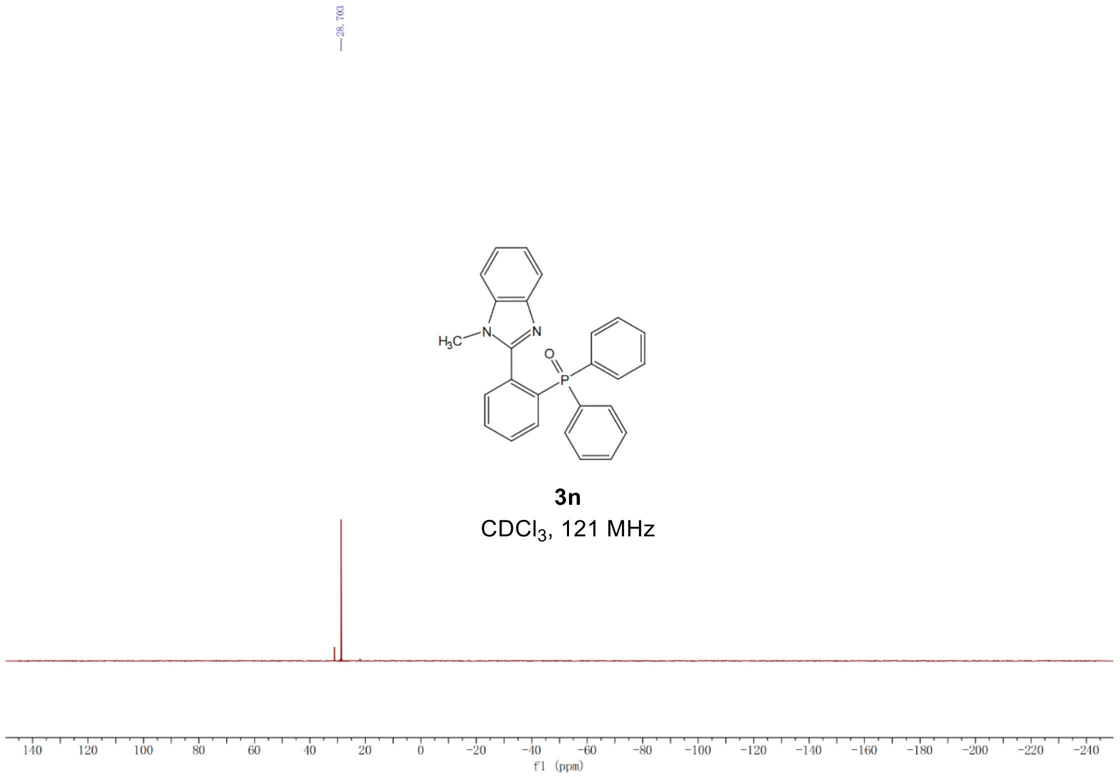

(2-(9-Isopropyl-9H-purin-6-yl)phenyl)di(naphthalen-2-yl)phosphine oxide (**3o**)

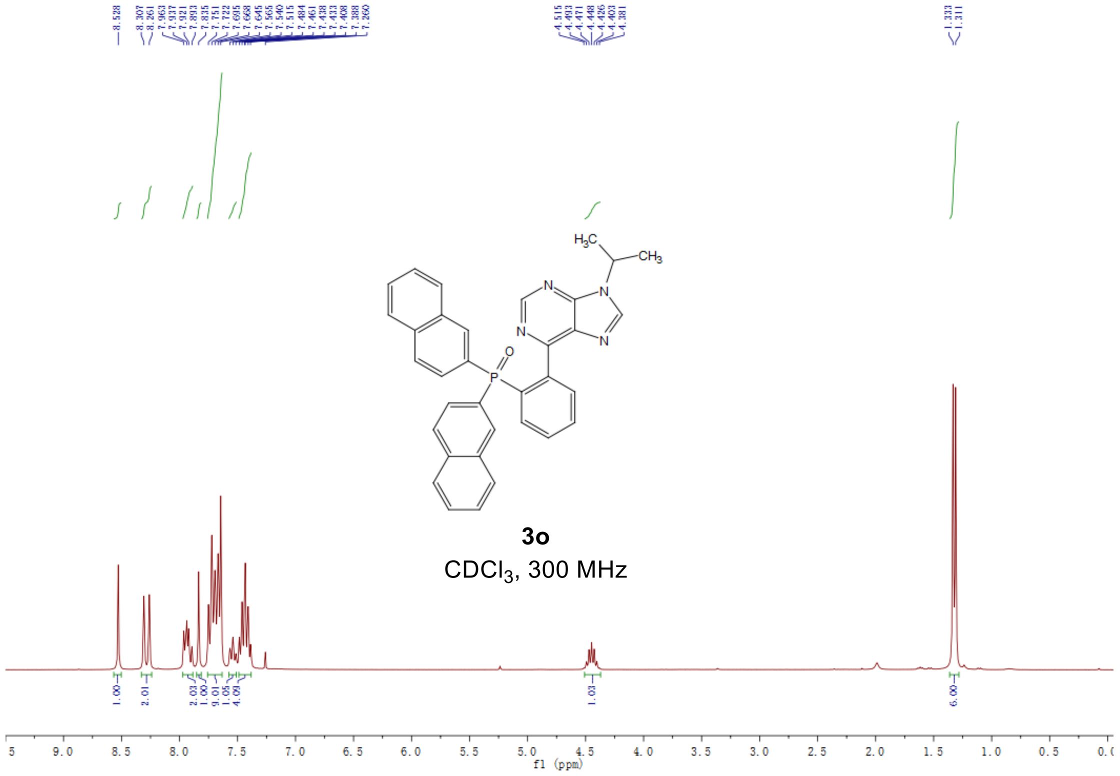

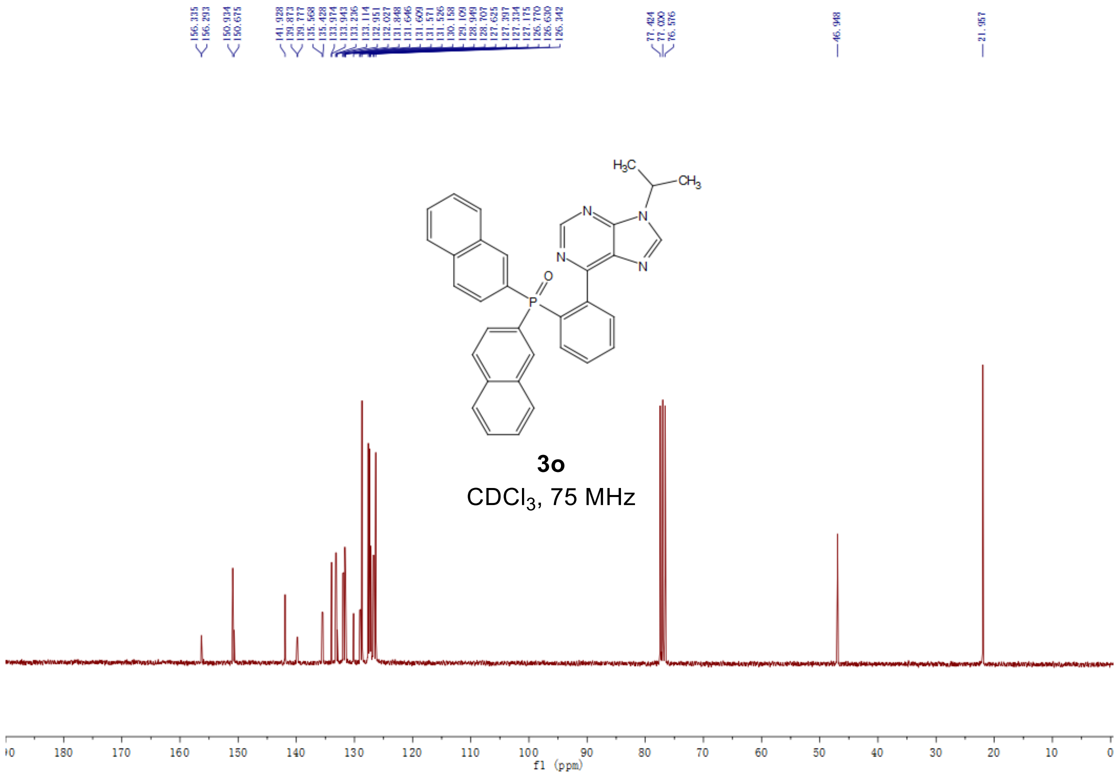
156.335
156.293
150.934
150.675
141.928
139.873
139.777
135.568
135.428
133.974
133.943
133.236
133.114
132.951
132.027
131.848
131.646
131.609
131.571
131.526
130.158
129.109
128.949
128.707
127.625
127.397
127.334
127.175
126.770
126.630
126.342
77.424
77.000
76.576
46.948
21.957
H3C
CH3
N
N
N
N
O
P
3o
CDCl3, 75 MHz
f1 (ppm)

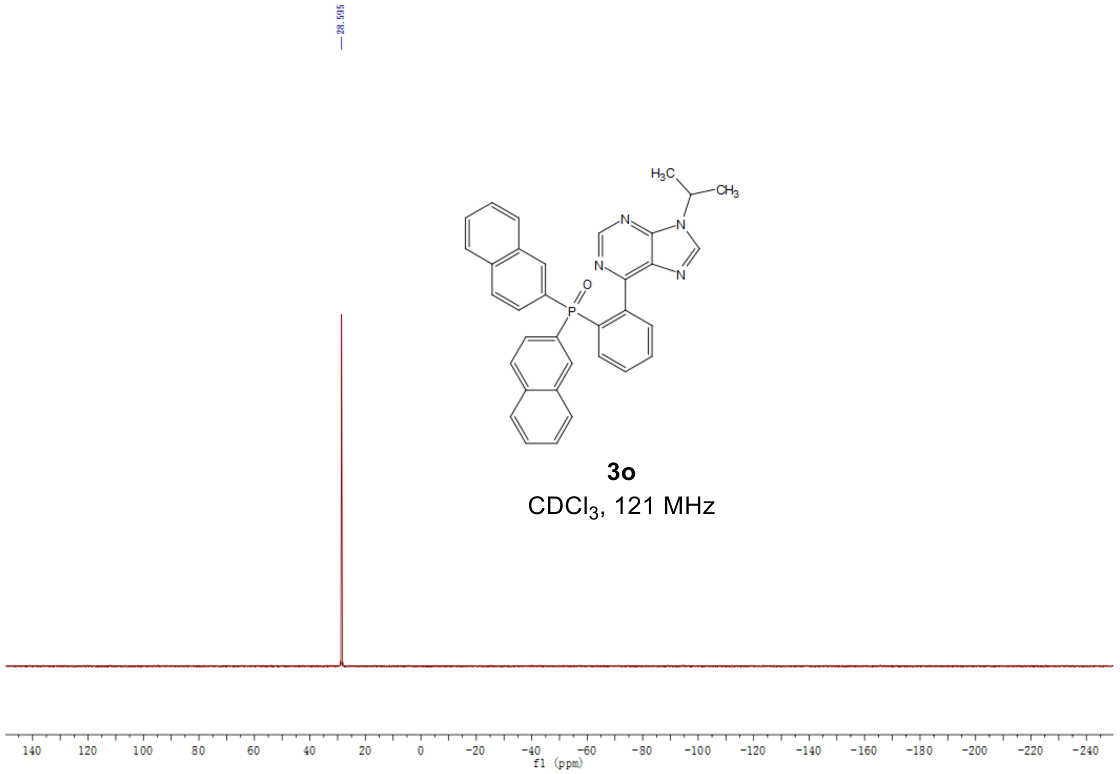
28.595
H3C
CH3
N
N
N
N
O
P
3o
CDCl3, 121 MHz
f1 (ppm)

(2-(9-Isopropyl-9H-purin-6-yl)phenyl)di-p-tolylphosphine oxide (**3p**)

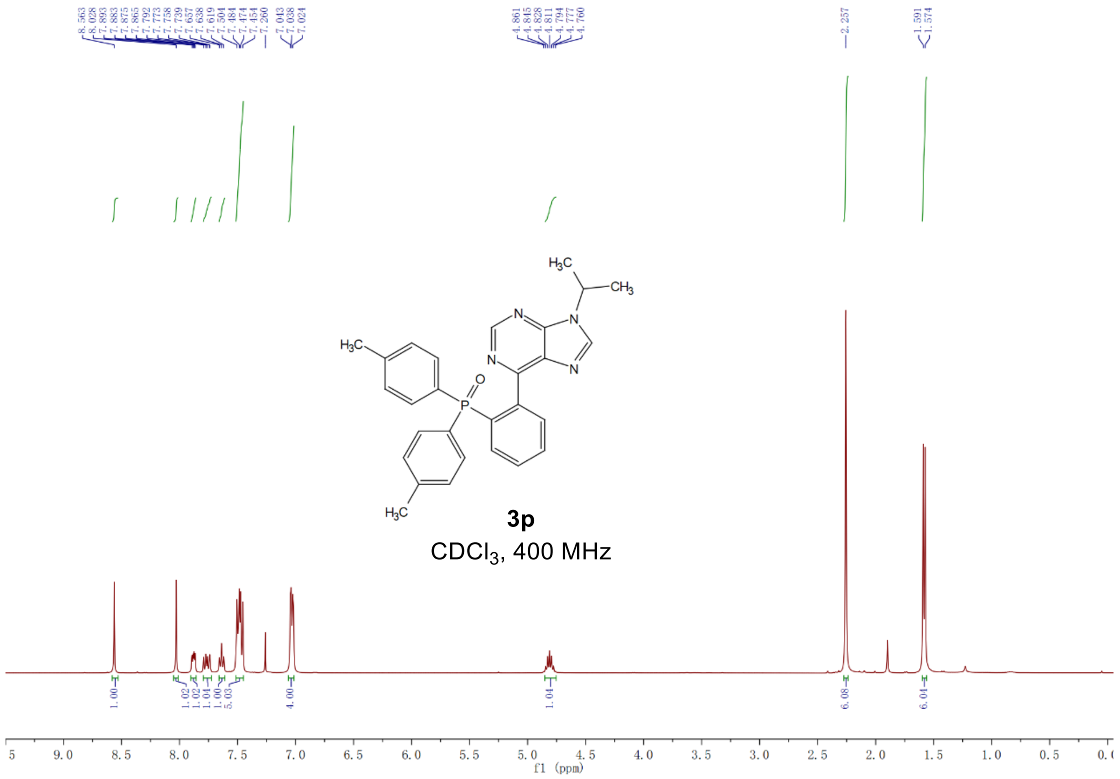

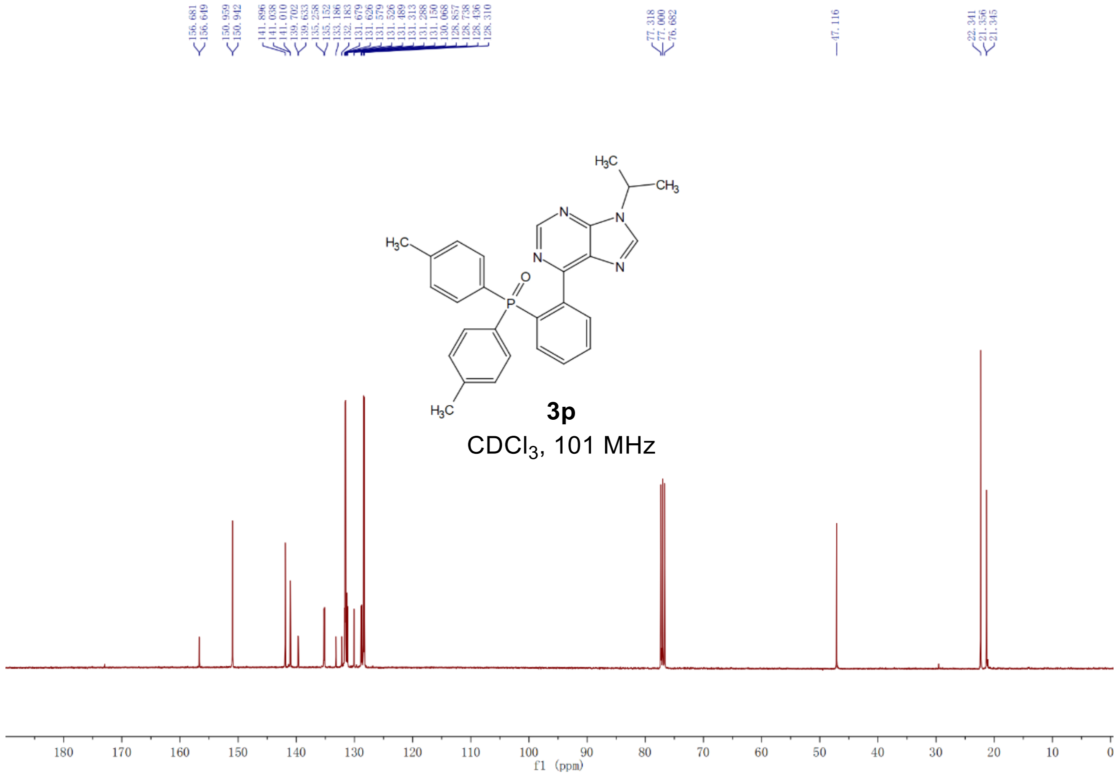

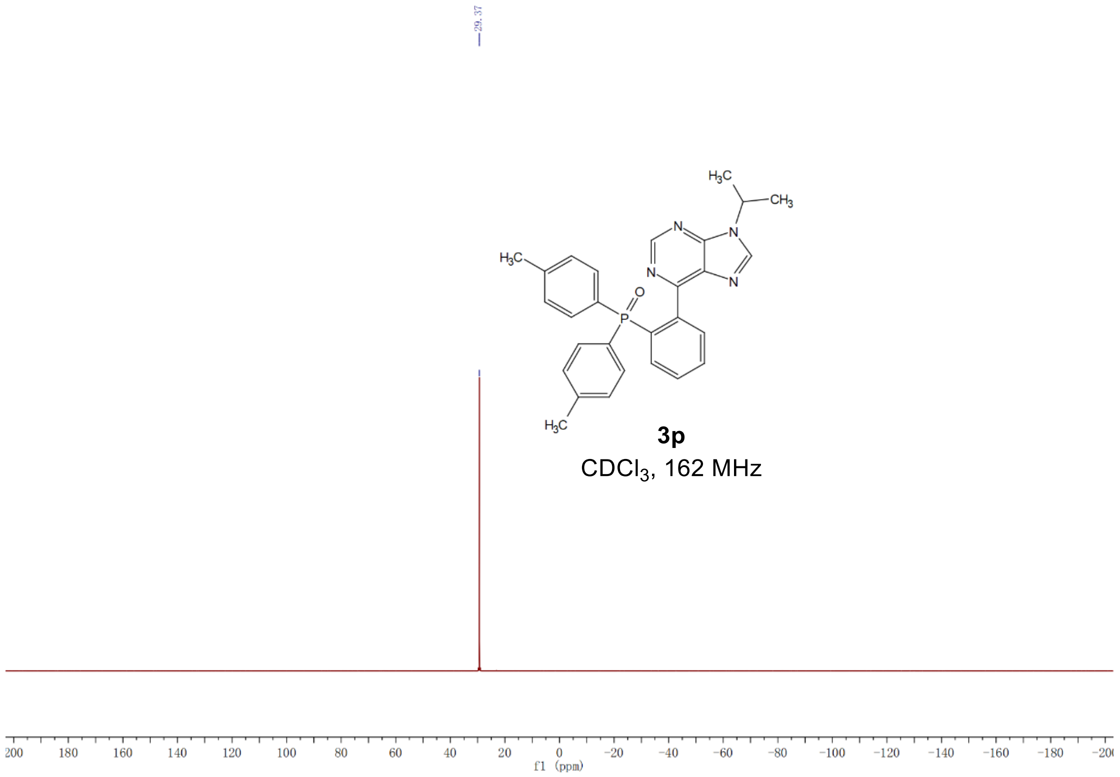

Bis(4-chlorophenyl)(2-(9-isopropyl-9H-purin-6-yl)phenyl)phosphine oxide (**3q**)

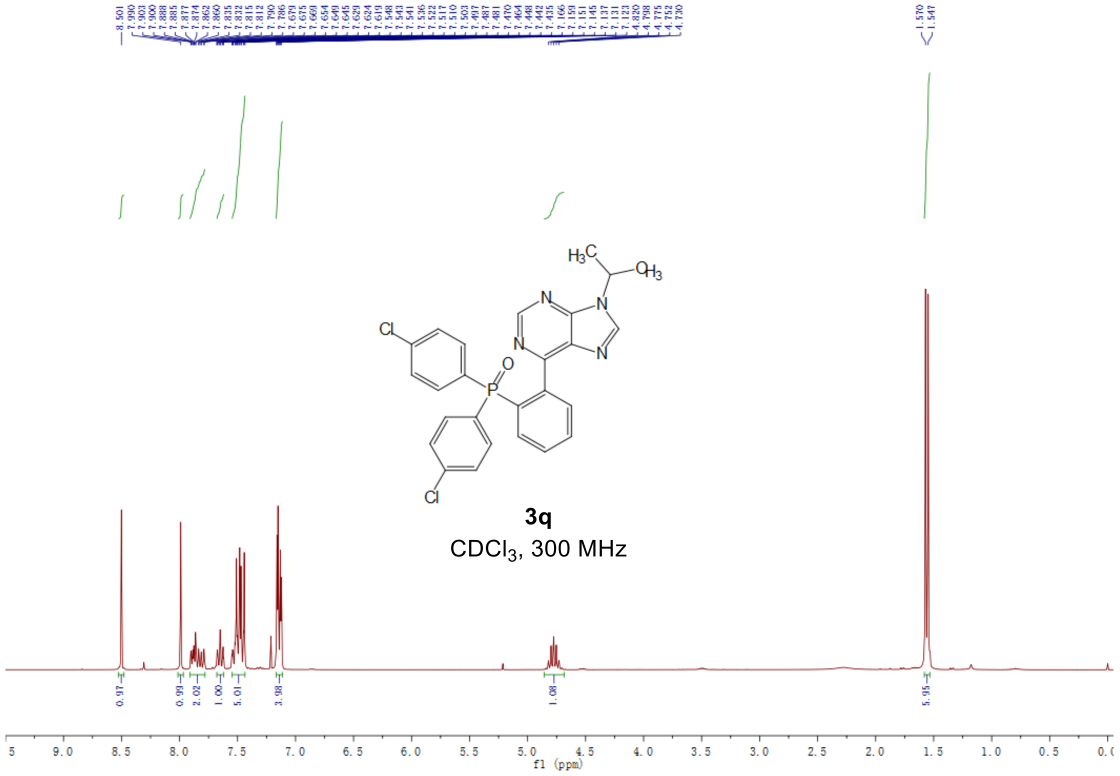

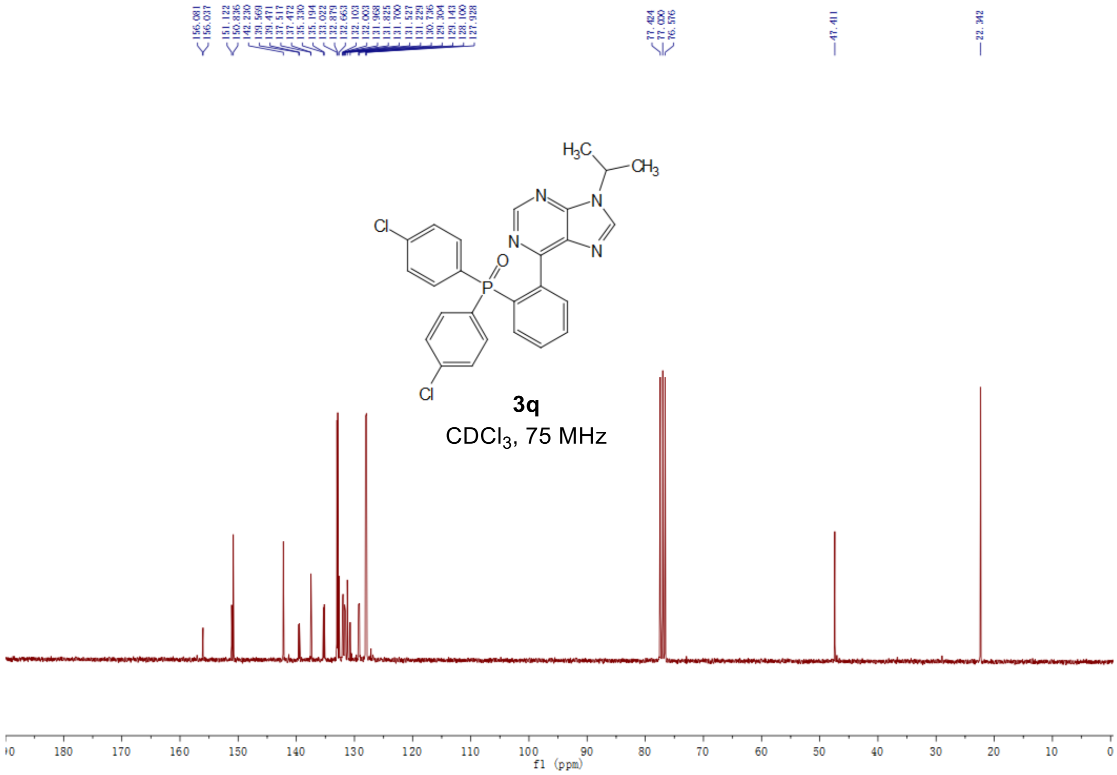

3q
$CDCl_3$, 75 MHz
f1 (ppm)

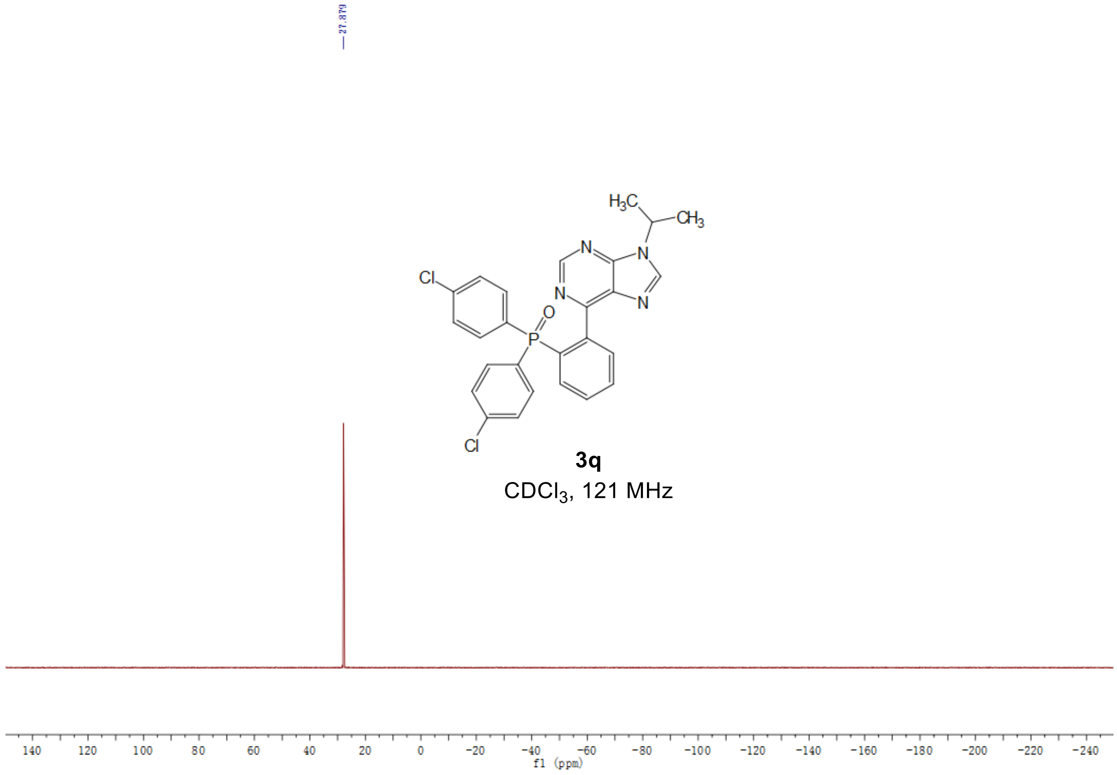

3q
$CDCl_3$, 121 MHz
f1 (ppm)

(2-(9-Isopropyl-9H-purin-6-yl)phenyl)bis(4-methoxyphenyl)phosphine oxide (**3r**)

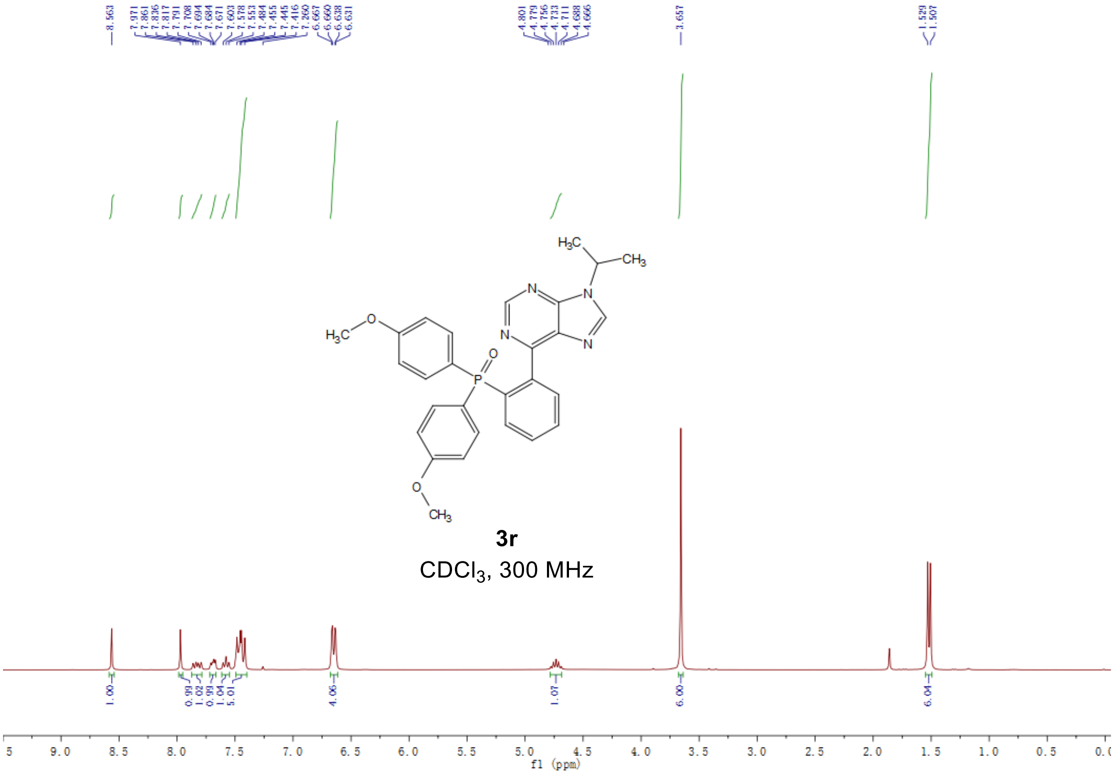

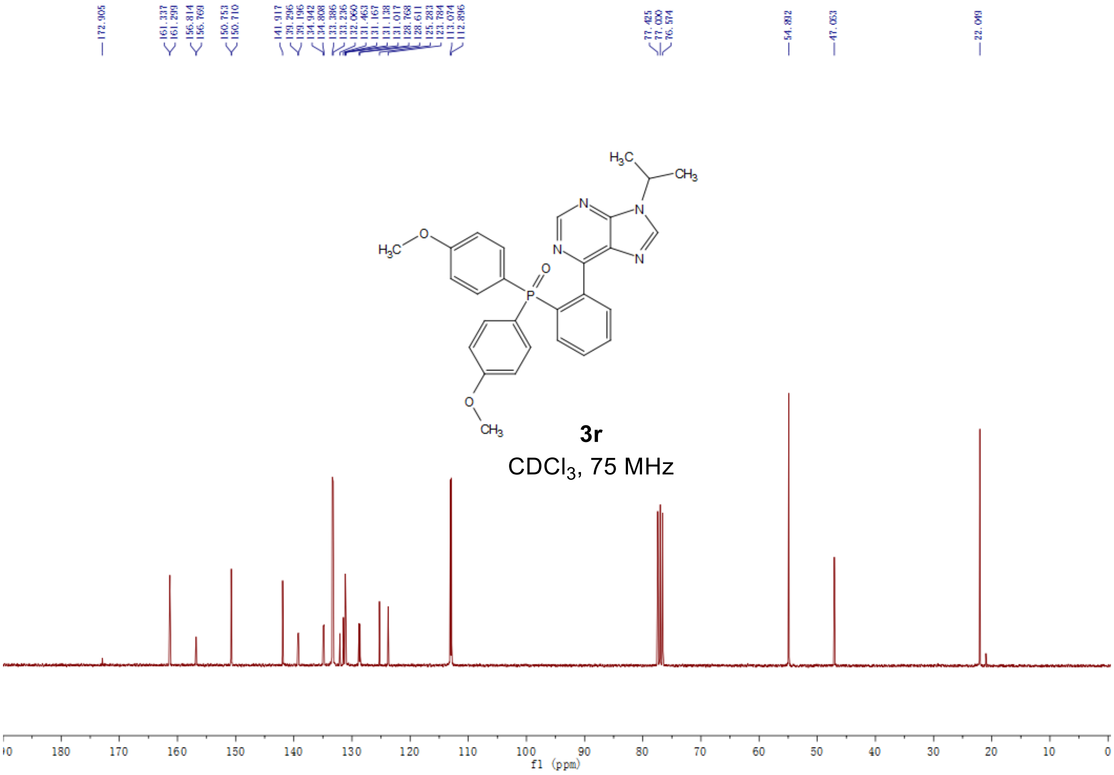

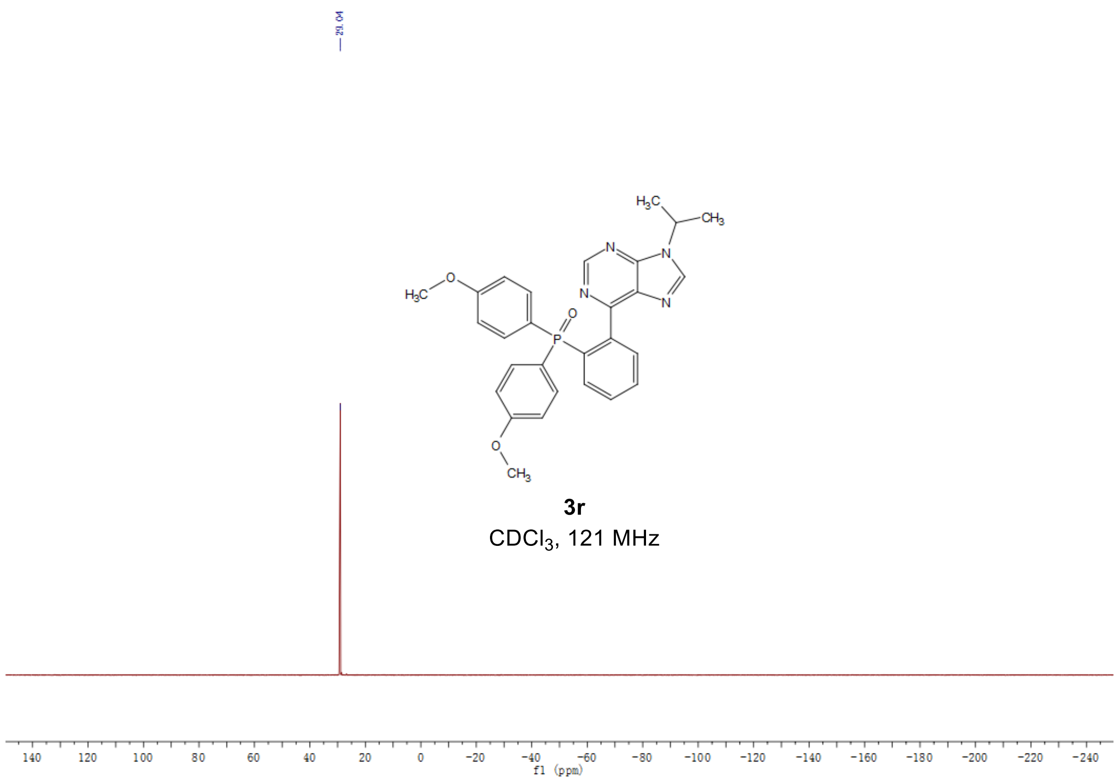

Di([1,1'-biphenyl]-4-yl)(2-(9-isopropyl-9H-purin-6-yl)phenyl)phosphine oxide (**3s**)

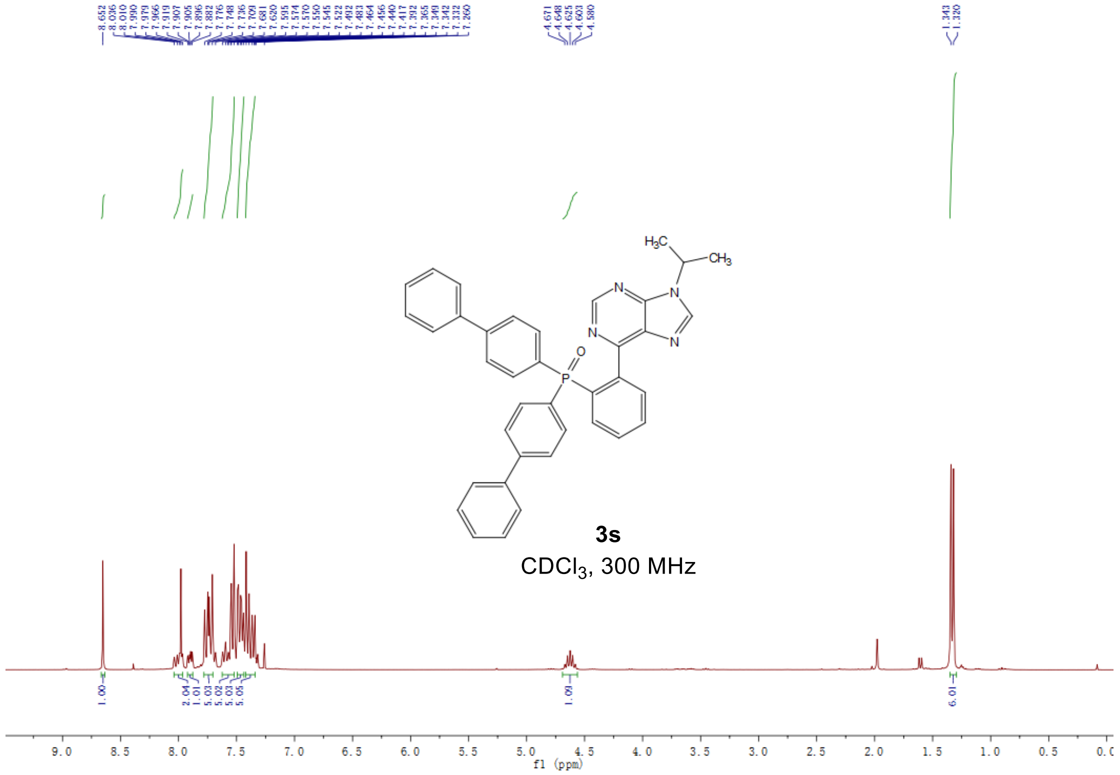

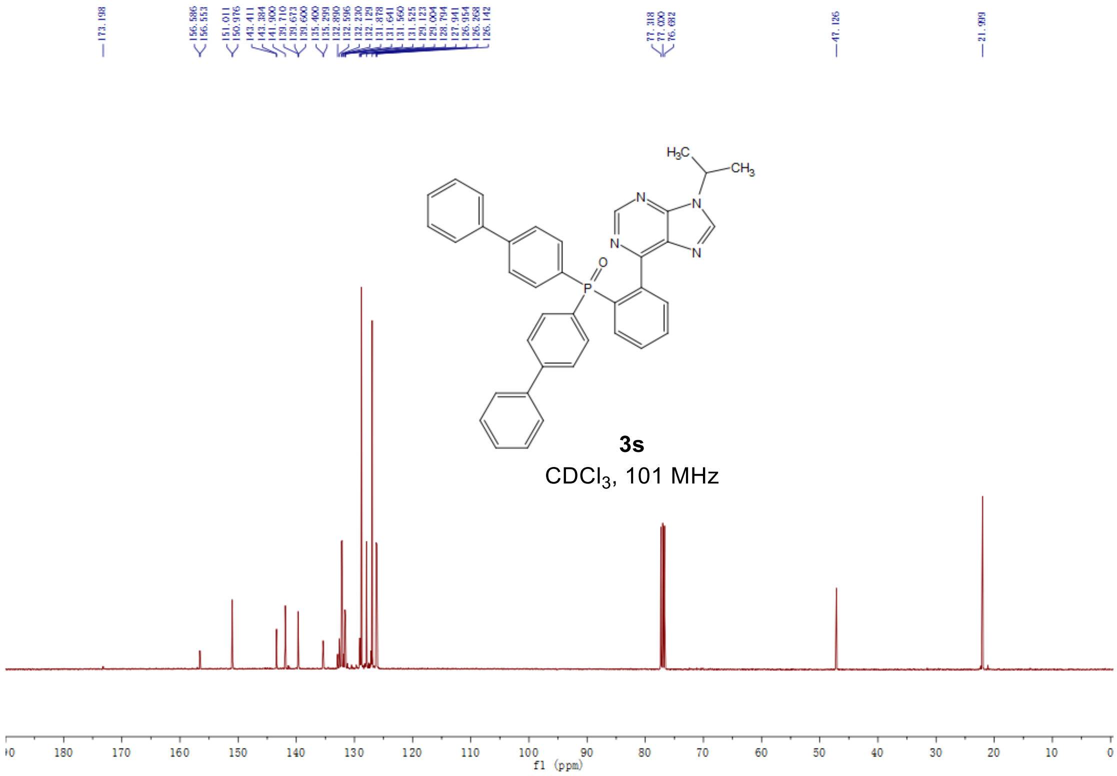
3s
CDCl$_3$, 101 MHz
f1 (ppm)

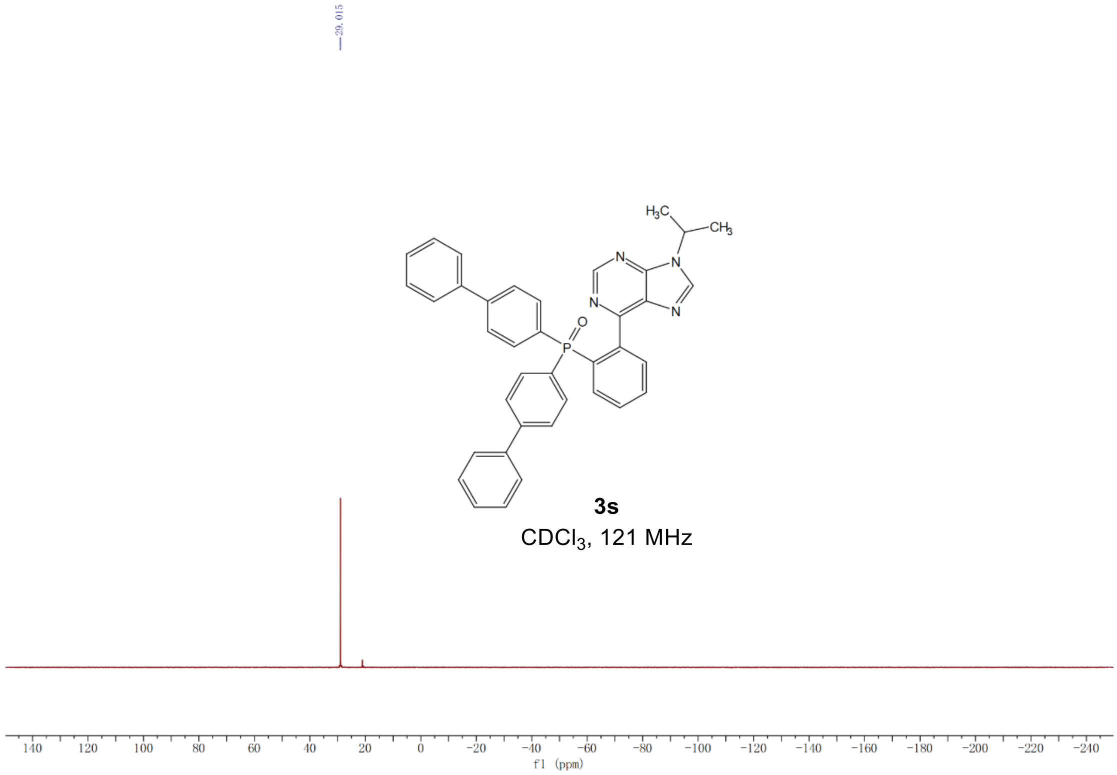
3s
CDCl$_3$, 121 MHz
f1 (ppm)

Eethyl (2-(9-isopropyl-9H-purin-6-yl)phenyl)(phenyl)phosphinate (**3t**)

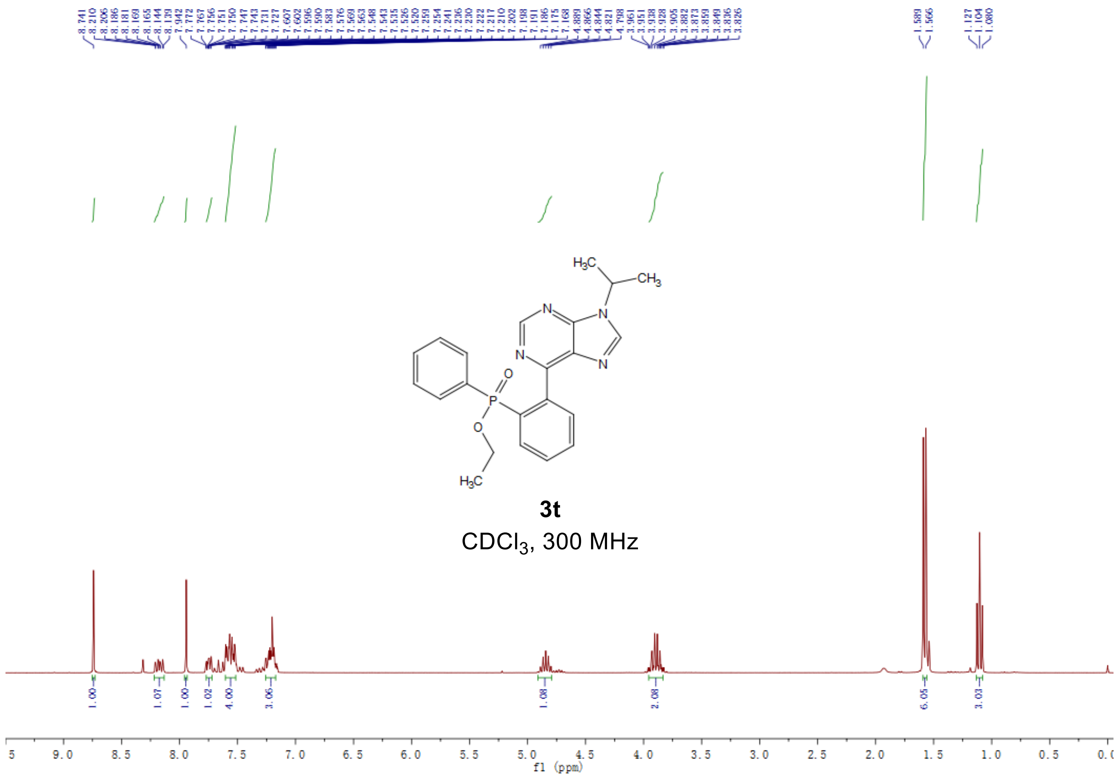

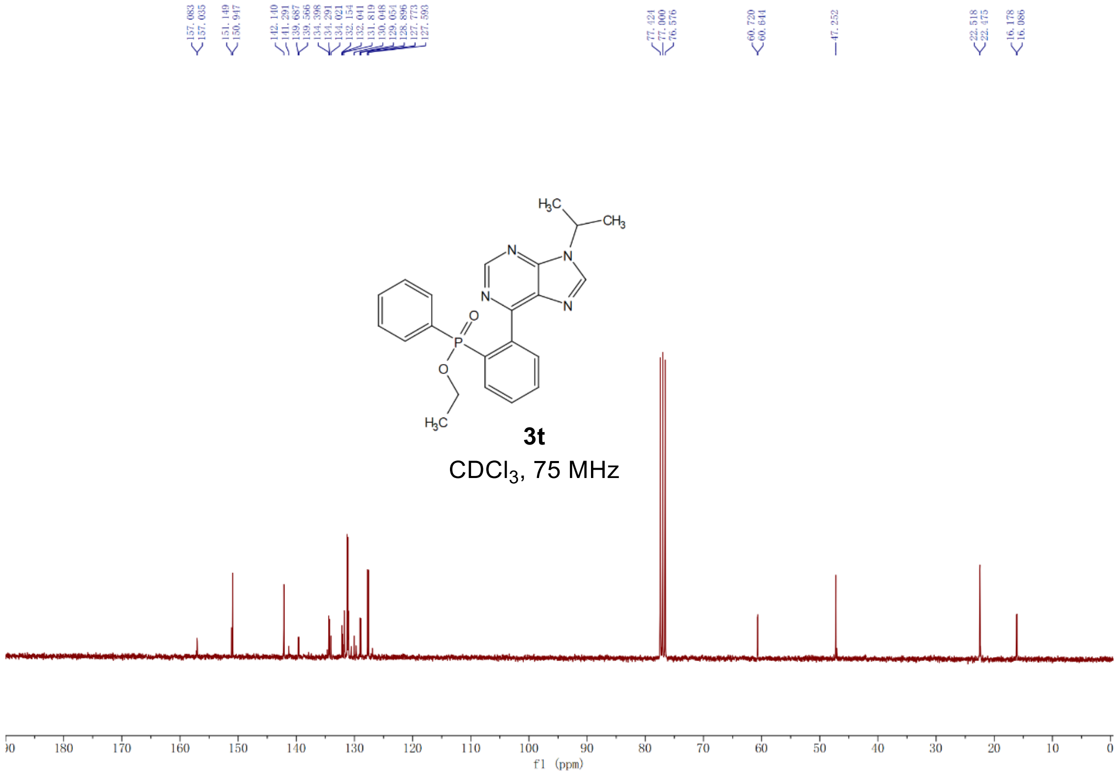

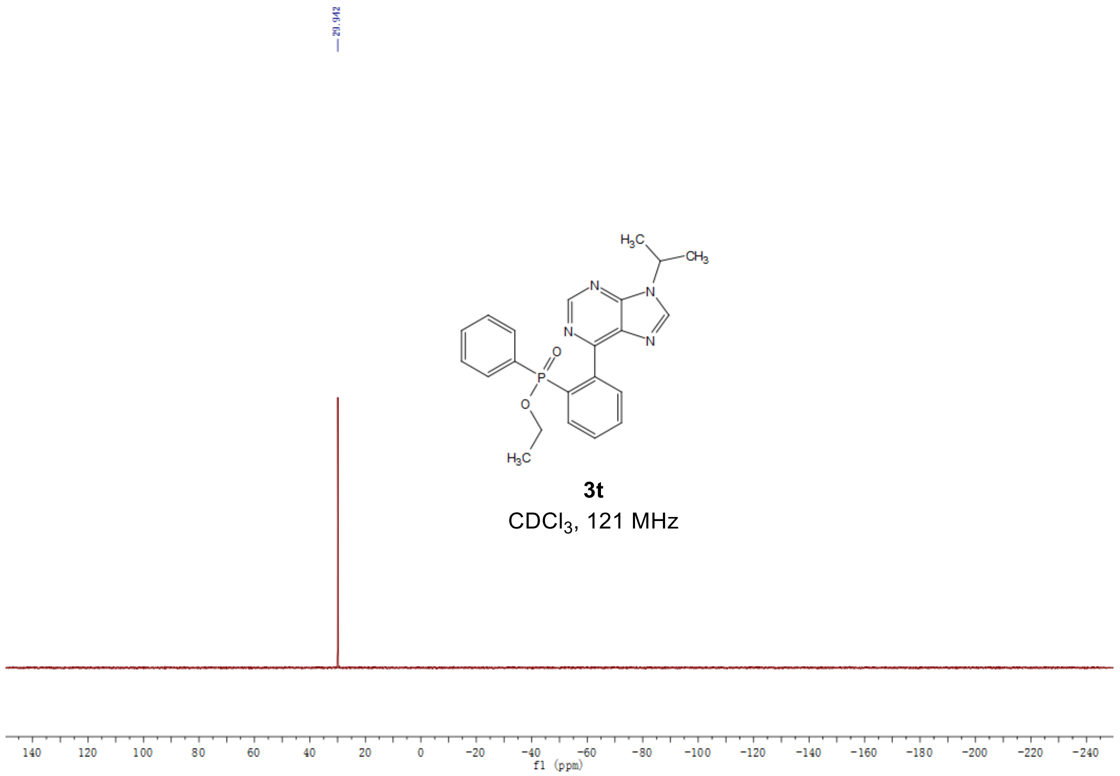

Isopropyl(2-(9-isopropyl-9H-purin-6-yl)phenyl)(phenyl)phosphine oxide (**3u**)

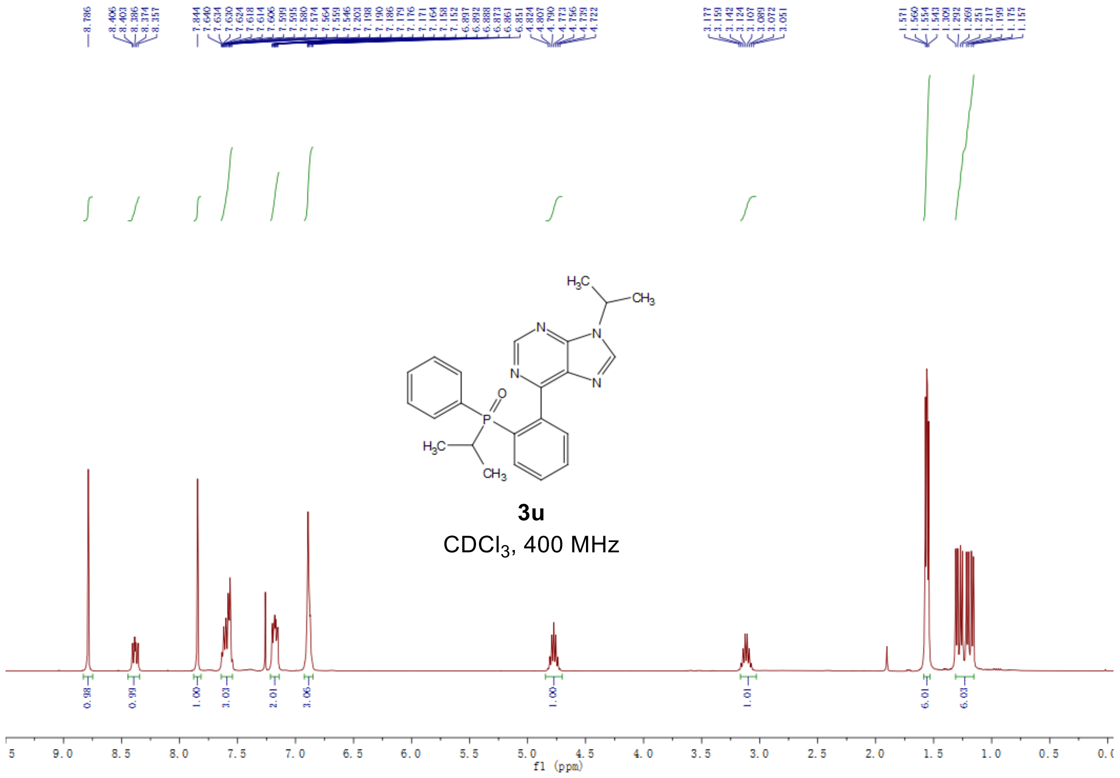

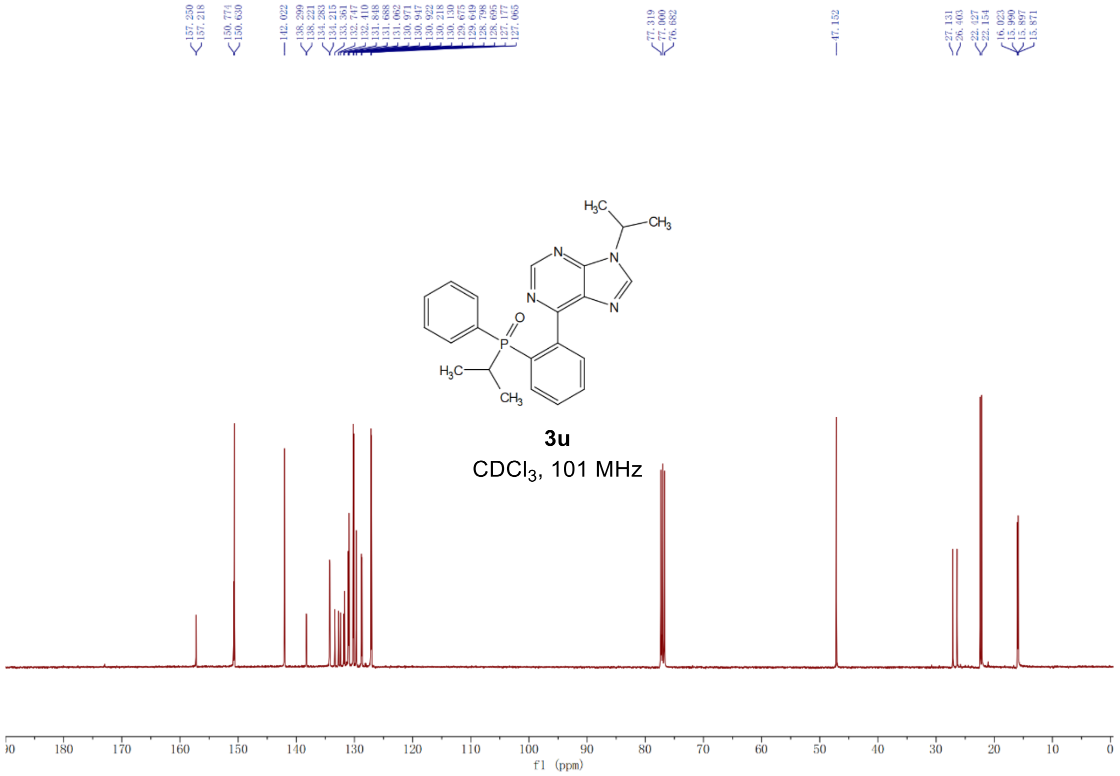
157.259
157.218
150.774
150.630
142.022
138.299
138.221
134.283
134.215
133.361
132.747
132.410
131.848
131.688
131.062
130.971
130.947
130.922
130.218
130.159
129.675
129.619
128.798
128.695
127.177
127.065
77.319
77.000
76.682
47.152
27.131
26.403
22.427
22.154
16.023
15.990
15.897
15.871
3u
CDCl3, 101 MHz
f1 (ppm)

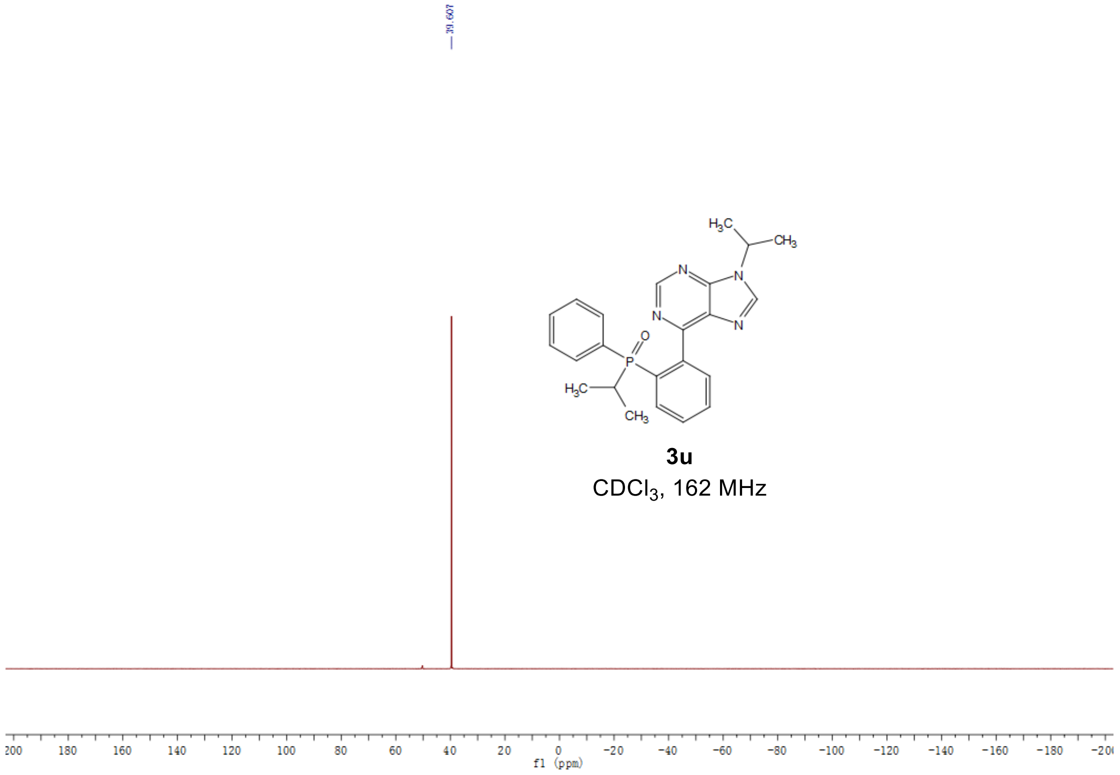
39.607
3u
CDCl3, 162 MHz
f1 (ppm)

(2-(9-Isopropyl-9H-purin-6-yl)phenyl)di(thiophen-2-yl)phosphine oxide (**3v**)

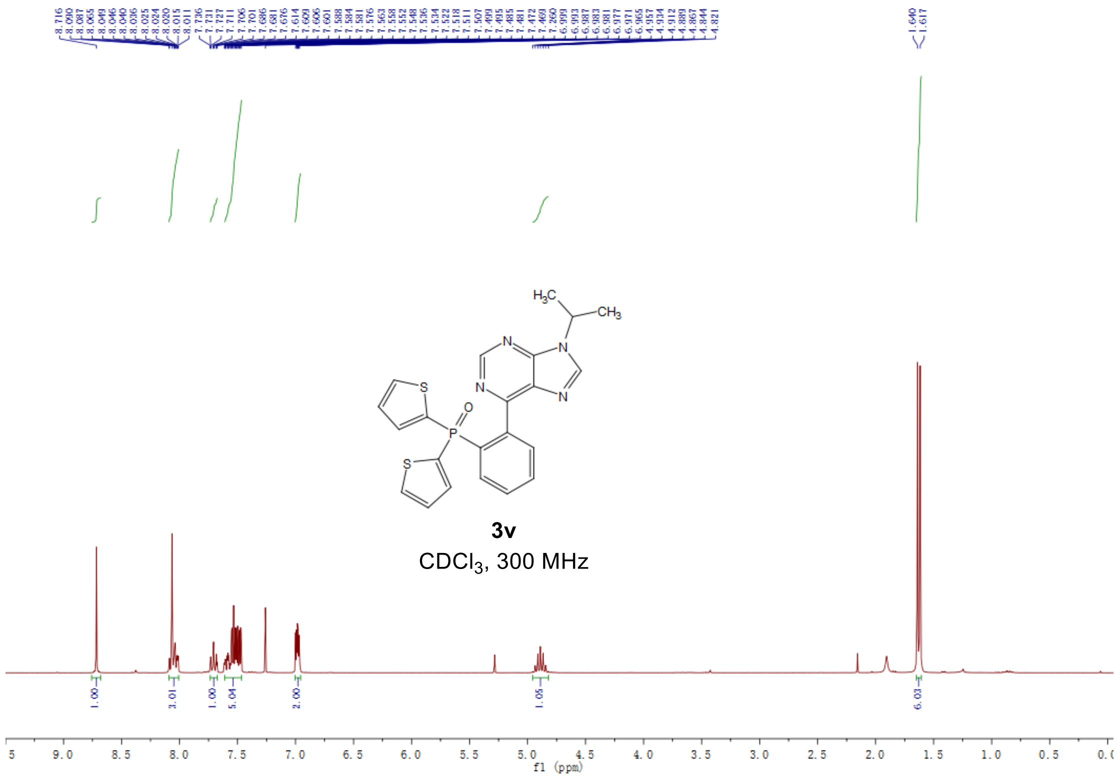

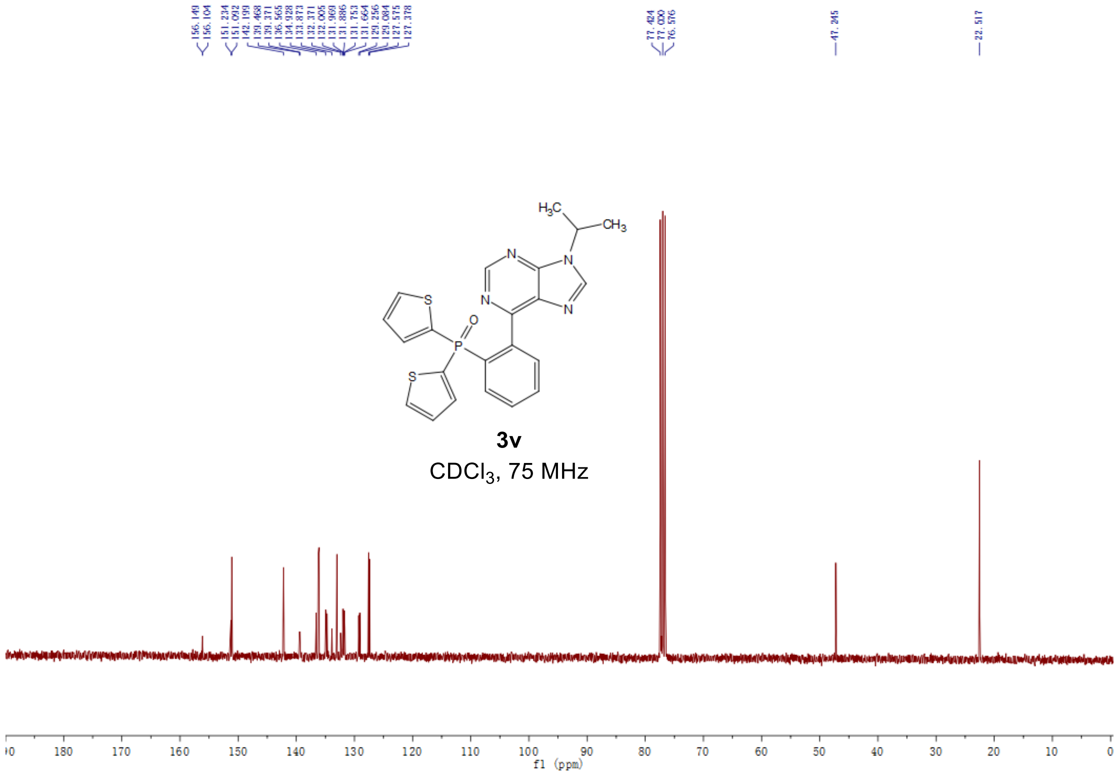

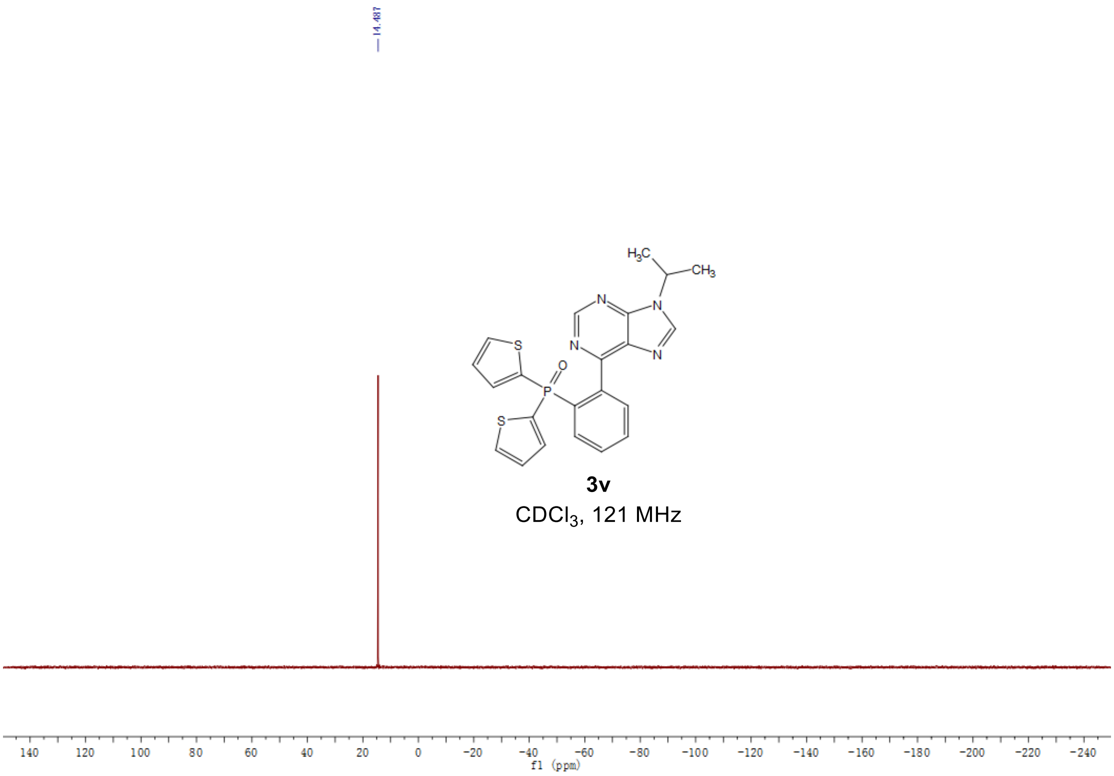

(2-(9-Isopropyl-9H-purin-6-yl)phenyl)dimethylphosphine oxide (**3w**)

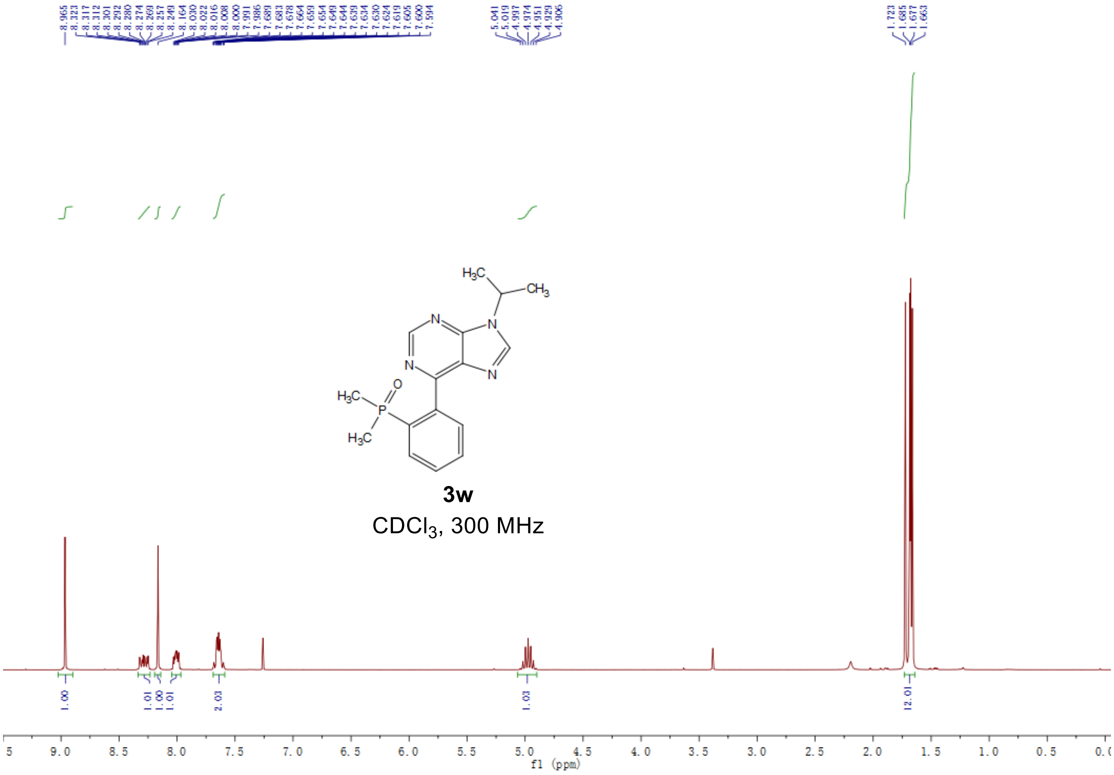

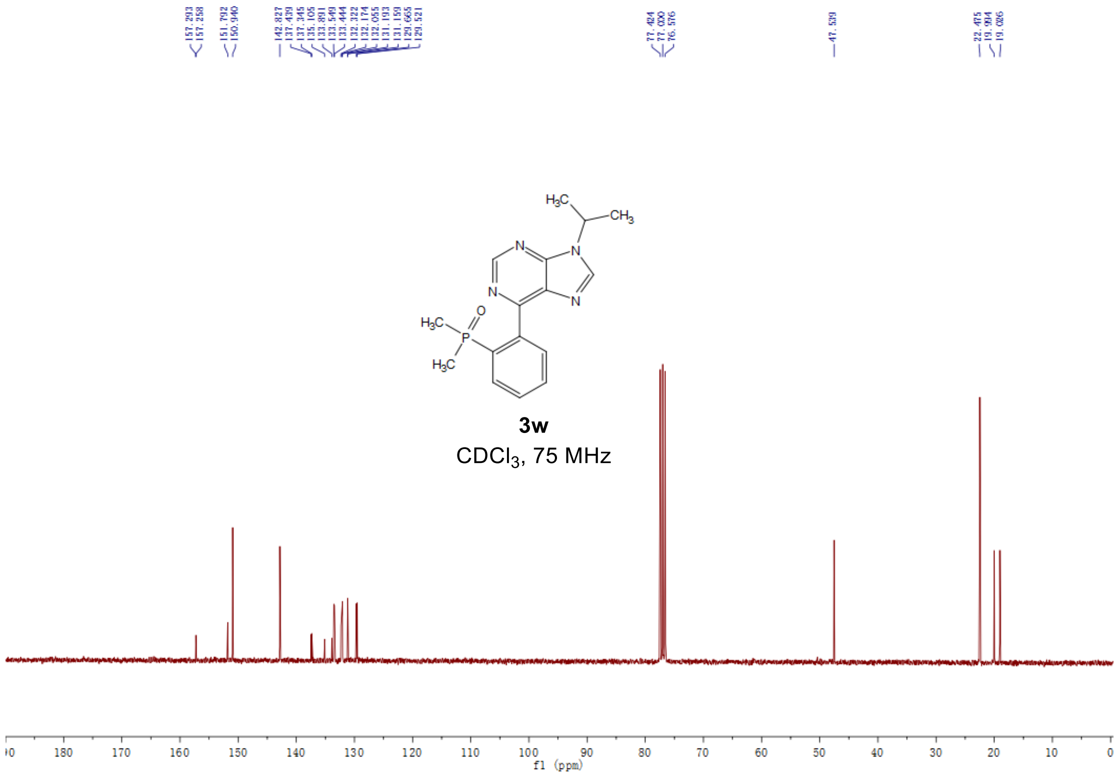
157.293
157.258
151.792
150.940
142.827
137.439
137.345
135.105
133.891
133.549
133.444
132.322
132.174
132.055
131.193
131.159
129.665
129.521
77.424
77.000
76.576
47.539
22.475
19.994
19.026
3w
CDCl3, 75 MHz
f1 (ppm)

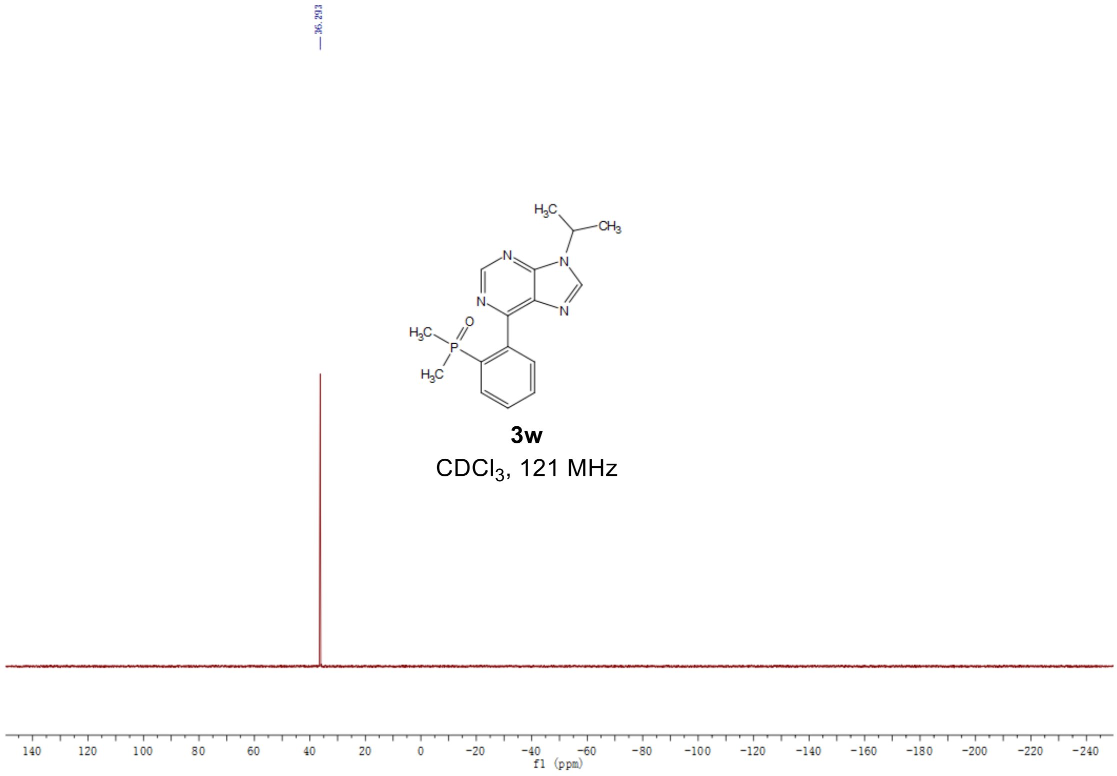
36.293
3w
CDCl3, 121 MHz
f1 (ppm)

7-Chloro-5-(2-(diphenylphosphoryl)phenyl)-1-methyl-1,3-dihydro-2H-benzo[e][1,4]diazepin-2-one (**3x**)

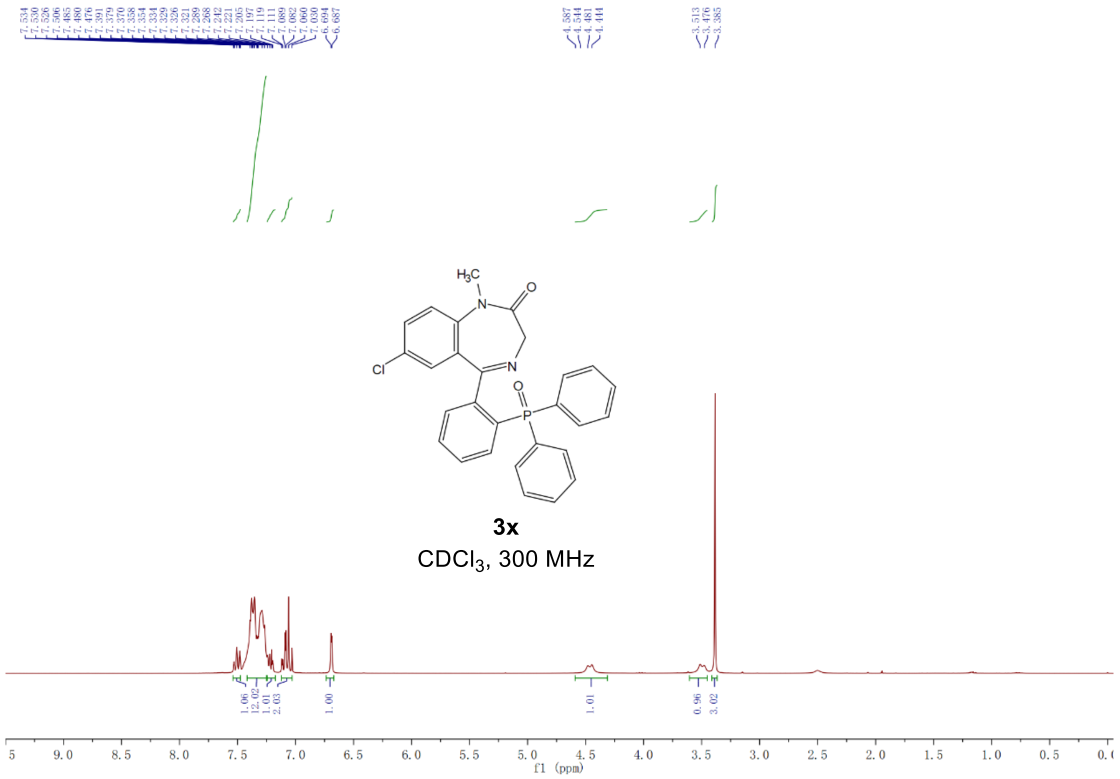

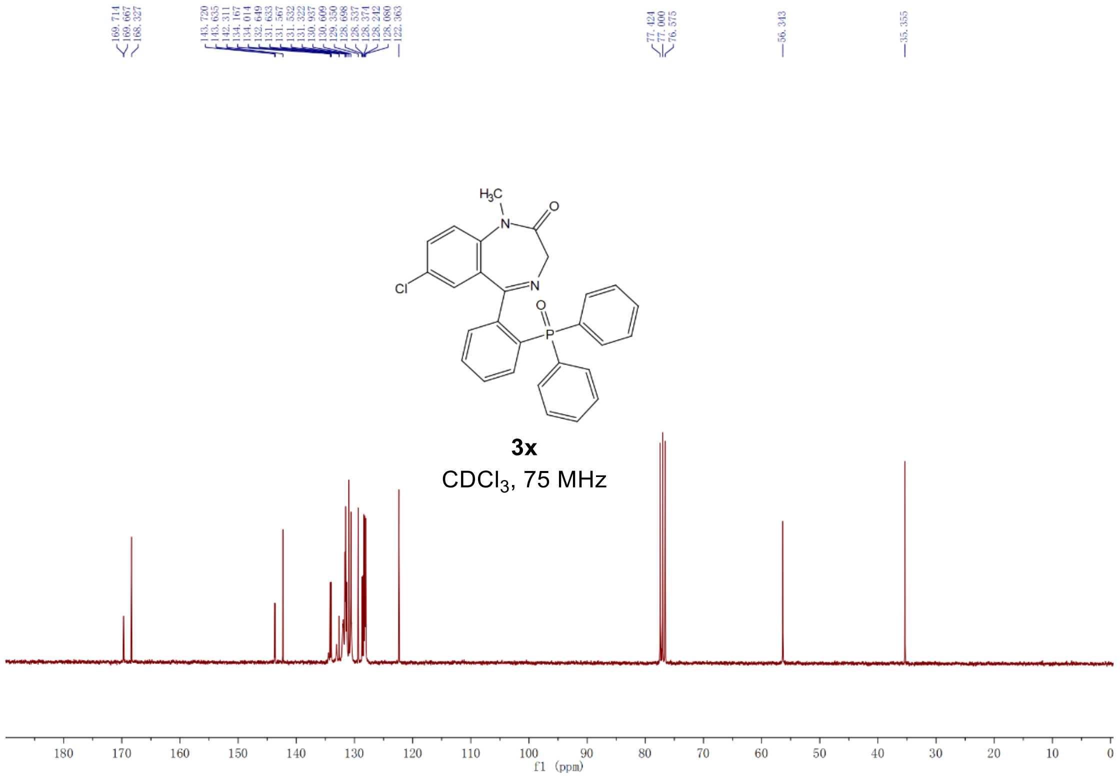

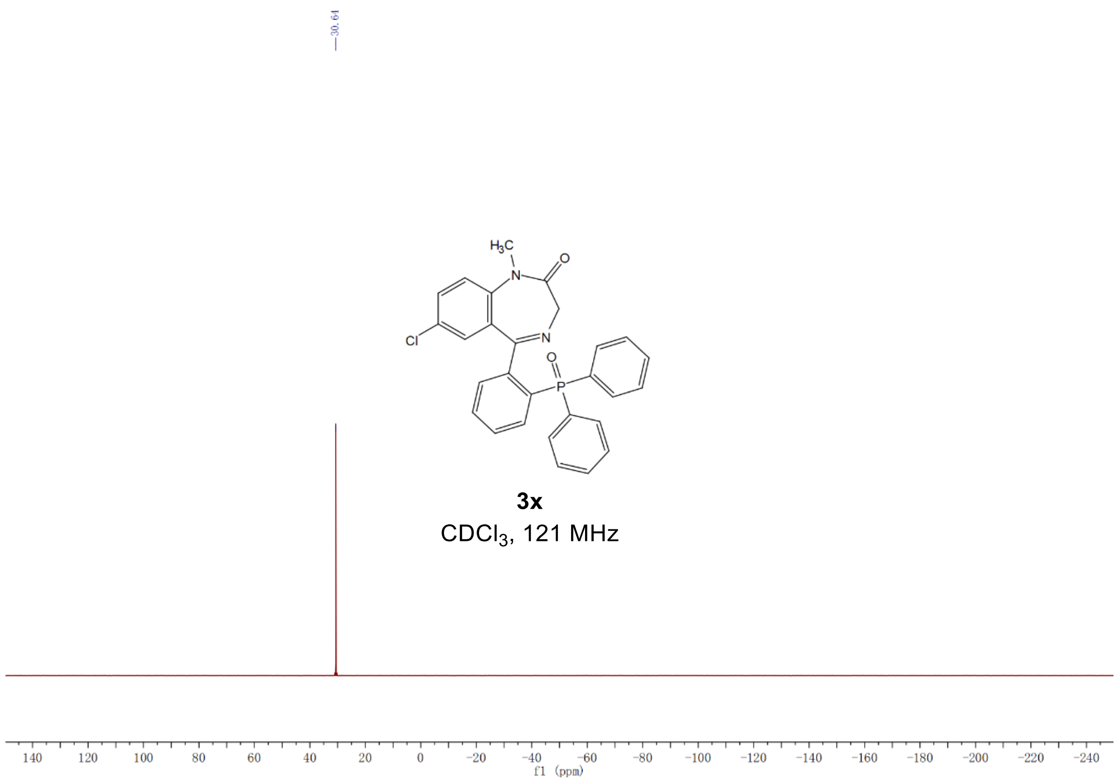

7-Chloro-5-(2-(dimethylphosphoryl)phenyl)-1-methyl-1,3-dihydro-2H-benzo[e][1,4]diazepin-2-one (**3y**)

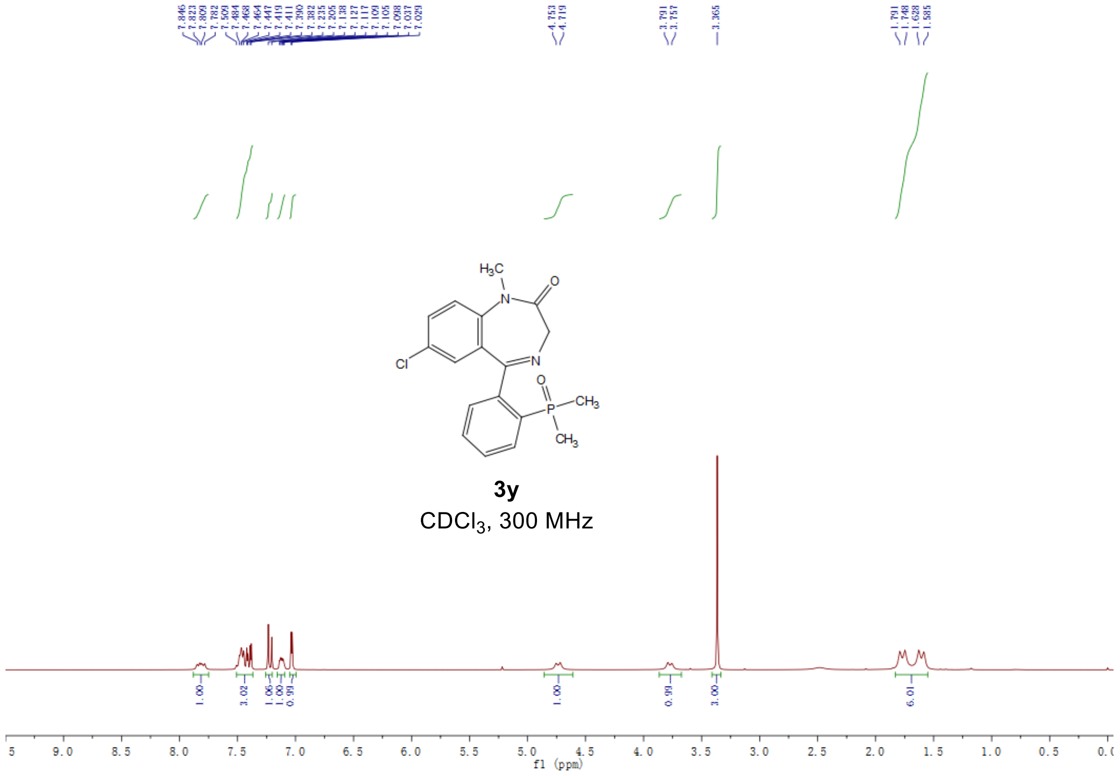

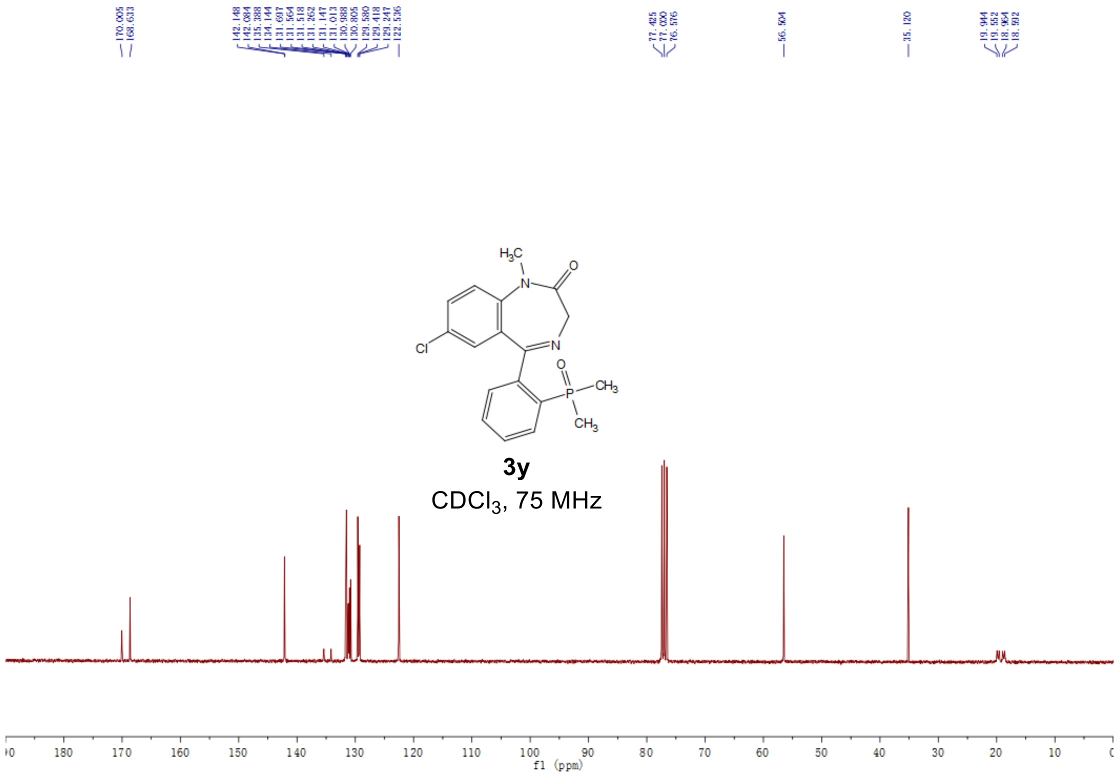
170.005
168.633
142.148
142.084
135.388
134.144
131.697
131.564
131.518
131.262
131.147
131.013
130.988
129.905
129.580
129.418
129.247
122.536
77.425
77.000
76.576
56.504
35.120
19.944
19.552
18.964
18.592
3y
CDCl3, 75 MHz
f1 (ppm)

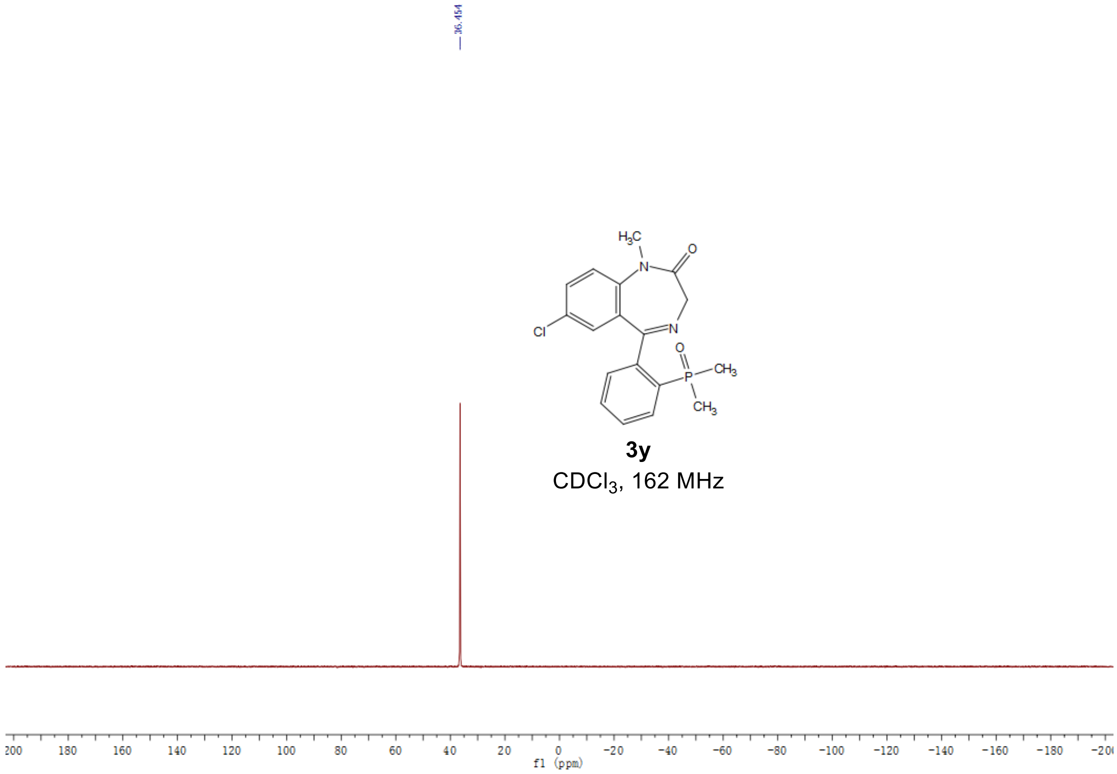
36.454
3y
CDCl3, 162 MHz
f1 (ppm)

Methyl 3-(4-(2-(diphenylphosphoryl)phenyl)-5-phenyloxazol-2-yl)propanoate (**3z**)

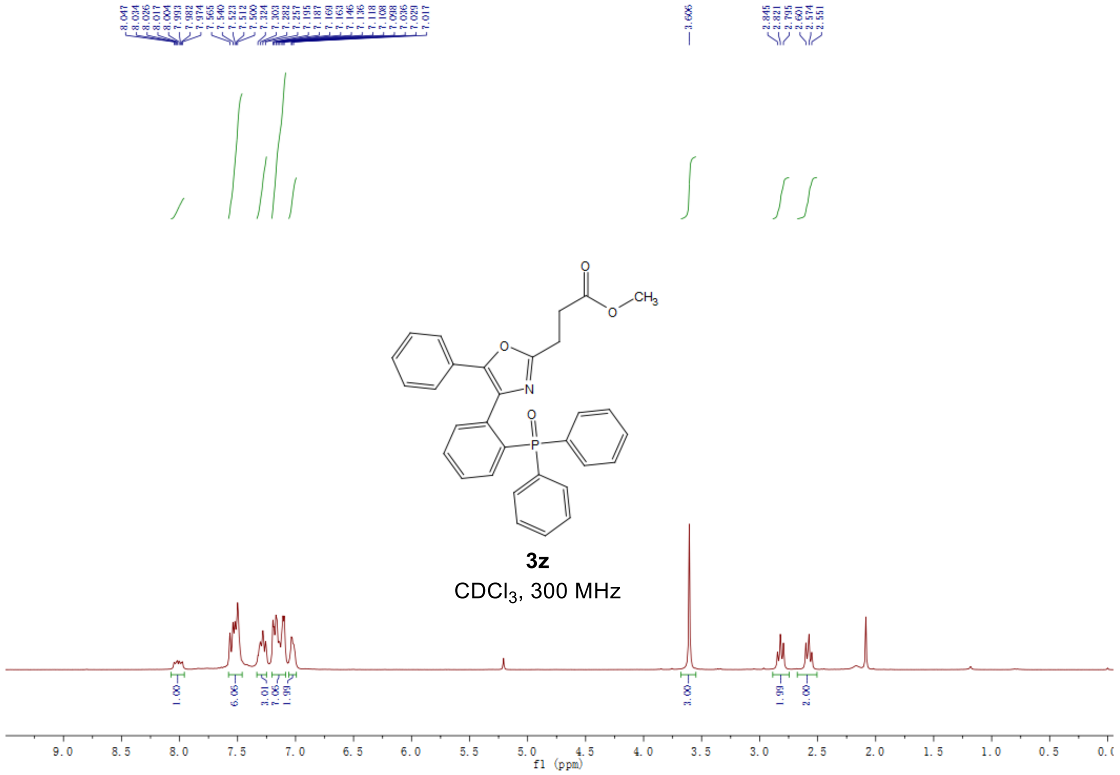

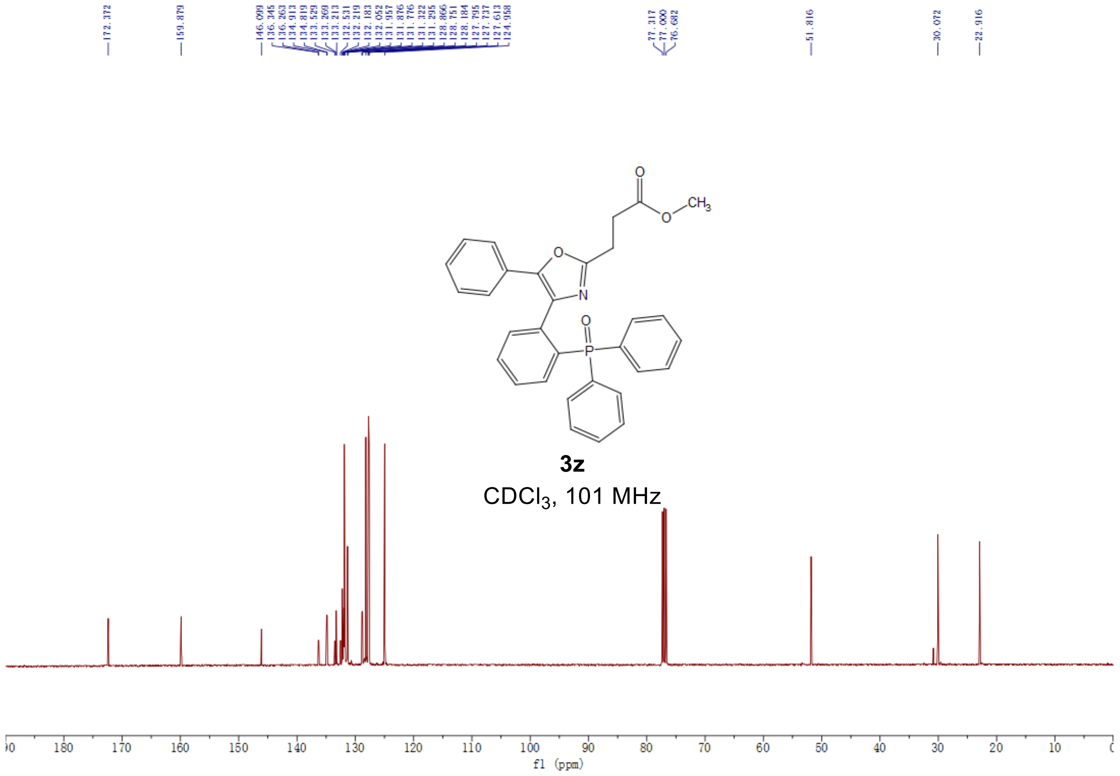

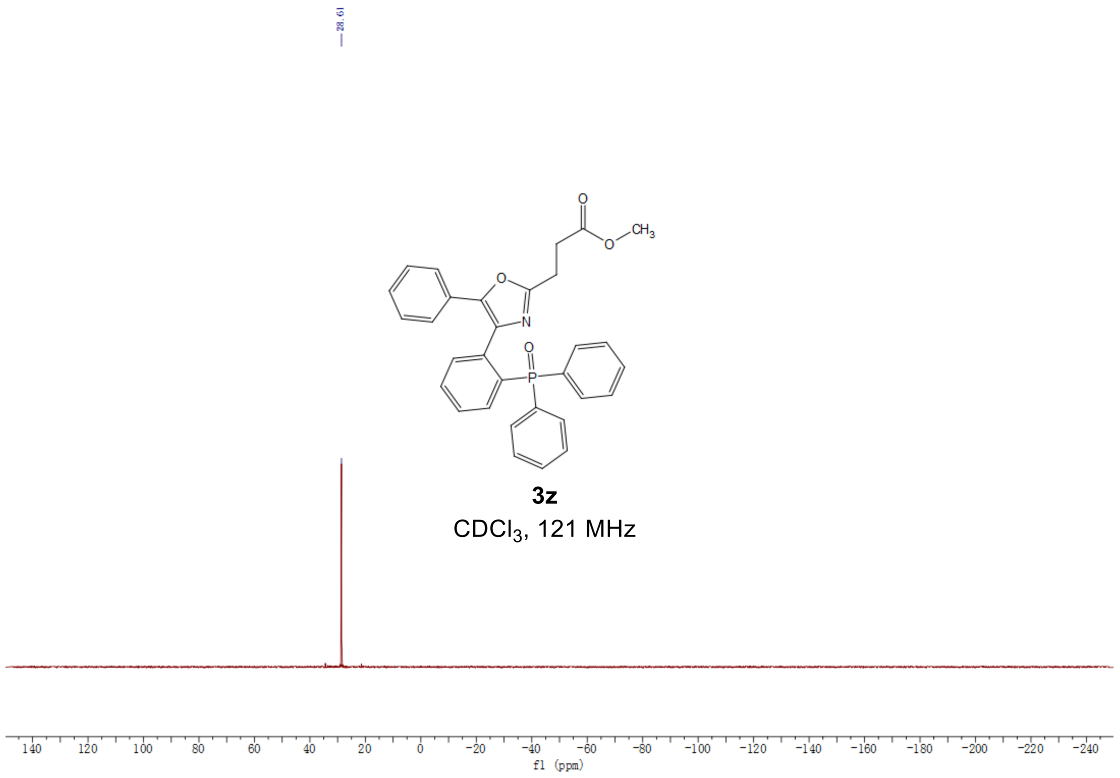

(2-(9-((3aR,4R,6R,6aR)-6-(Hydroxymethyl)-2,2-dimethyltetrahydrofuro[3,4-d][1,3]dioxol-4-yl)-9H-purin-6-yl)phenyl)diphenylphosphine oxide (**3aa**)

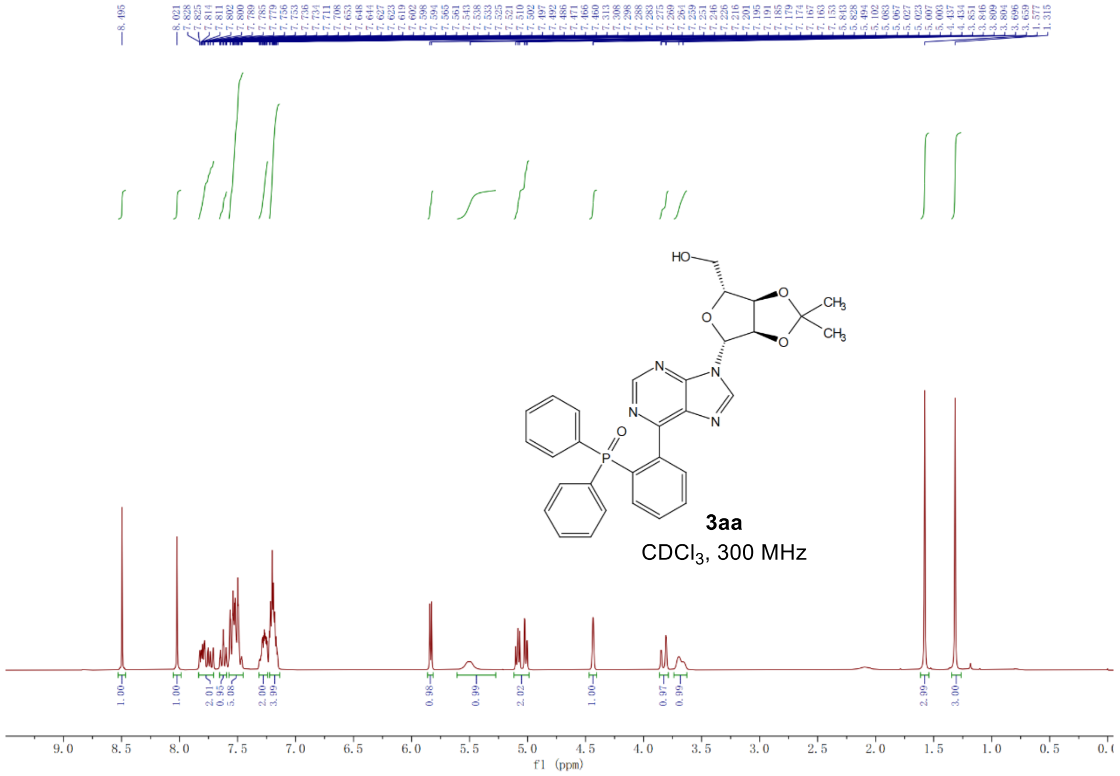

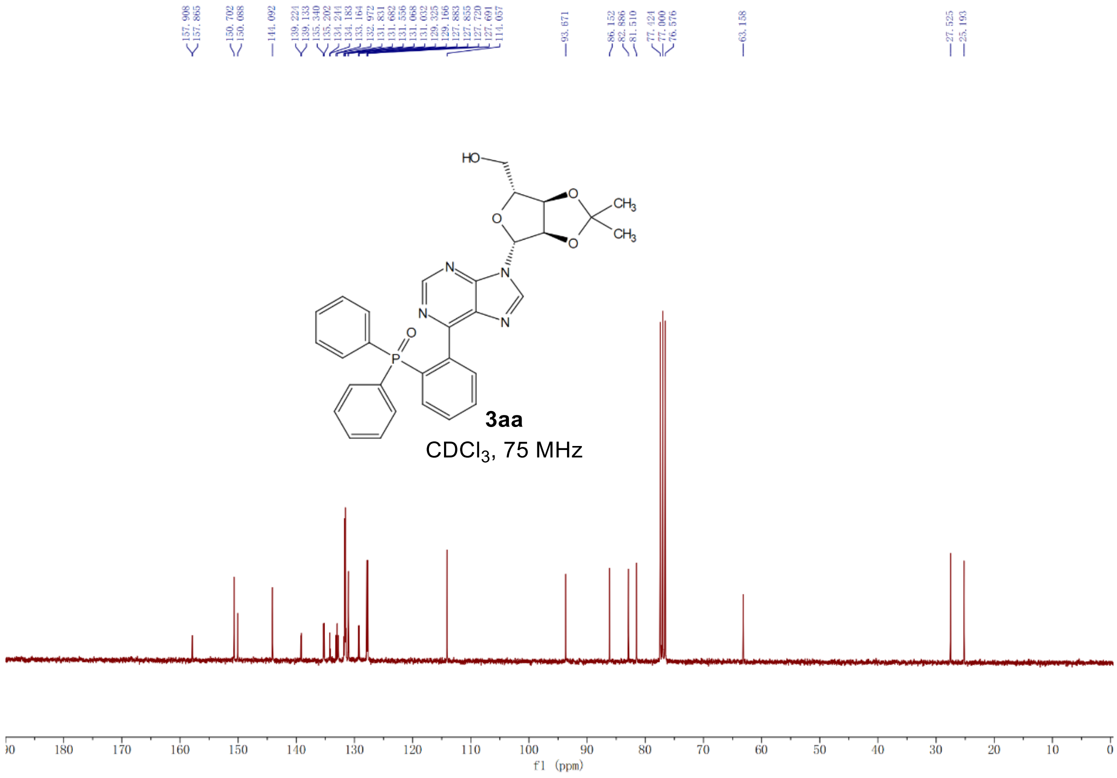
157.908
157.865
150.702
150.088
144.092
139.224
139.133
135.340
135.202
134.244
134.183
133.164
132.972
131.831
131.682
131.556
131.068
131.032
129.325
129.166
127.883
127.855
127.720
127.691
114.057
93.671
86.152
82.886
81.510
77.424
77.000
76.576
63.158
27.525
25.193
HO
O
O
O
CH3
CH3
N
N
N
N
O
P
3aa
CDCl3, 75 MHz
180 170 160 150 140 130 120 110 100 90 80 70 60 50 40 30 20 10 0
f1 (ppm)

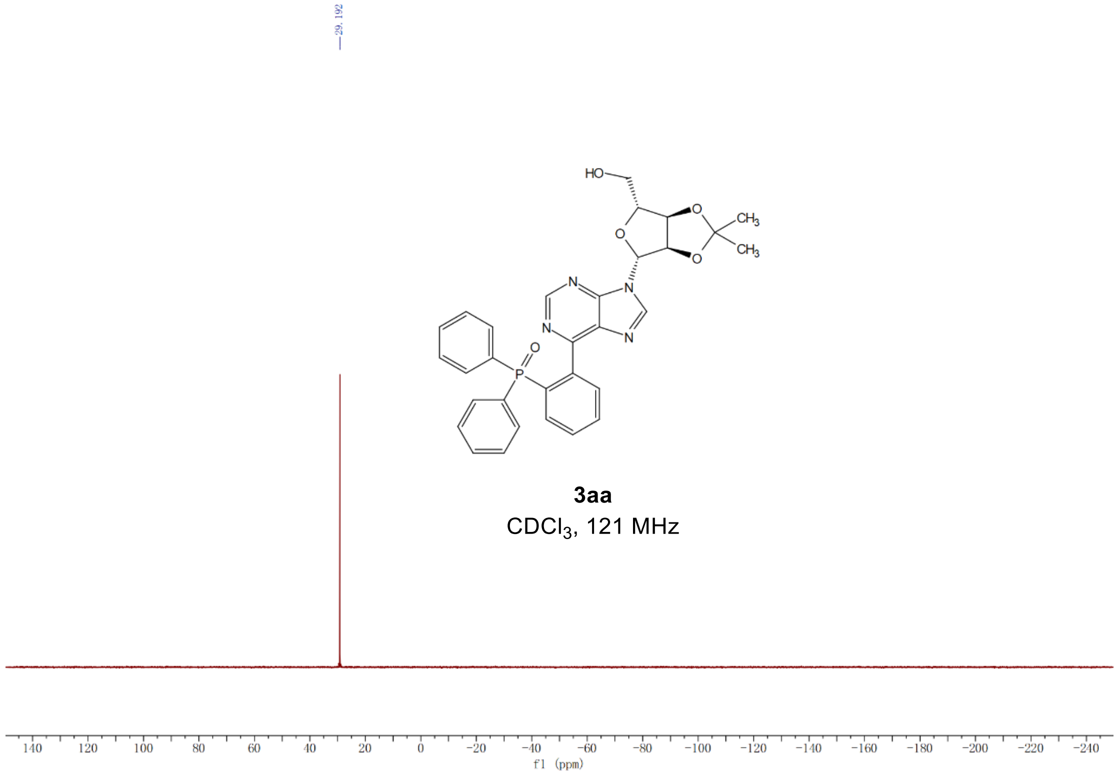
29.192
HO
O
O
O
CH3
CH3
N
N
N
N
O
P
3aa
CDCl3, 121 MHz
140 120 100 80 60 40 20 0 -20 -40 -60 -80 -100 -120 -140 -160 -180 -200 -220 -240
f1 (ppm)

(4-(9-Isopropyl-9H-purin-6-yl)phenyl)diphenylphosphine oxide (**4a**)

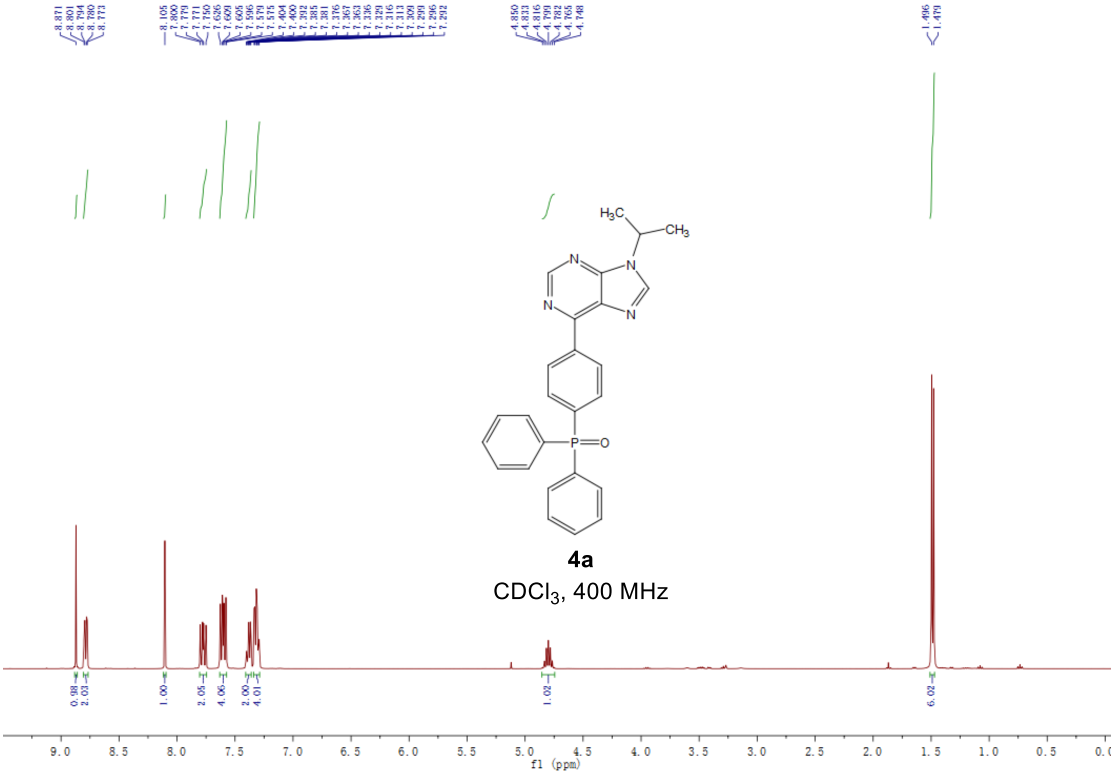

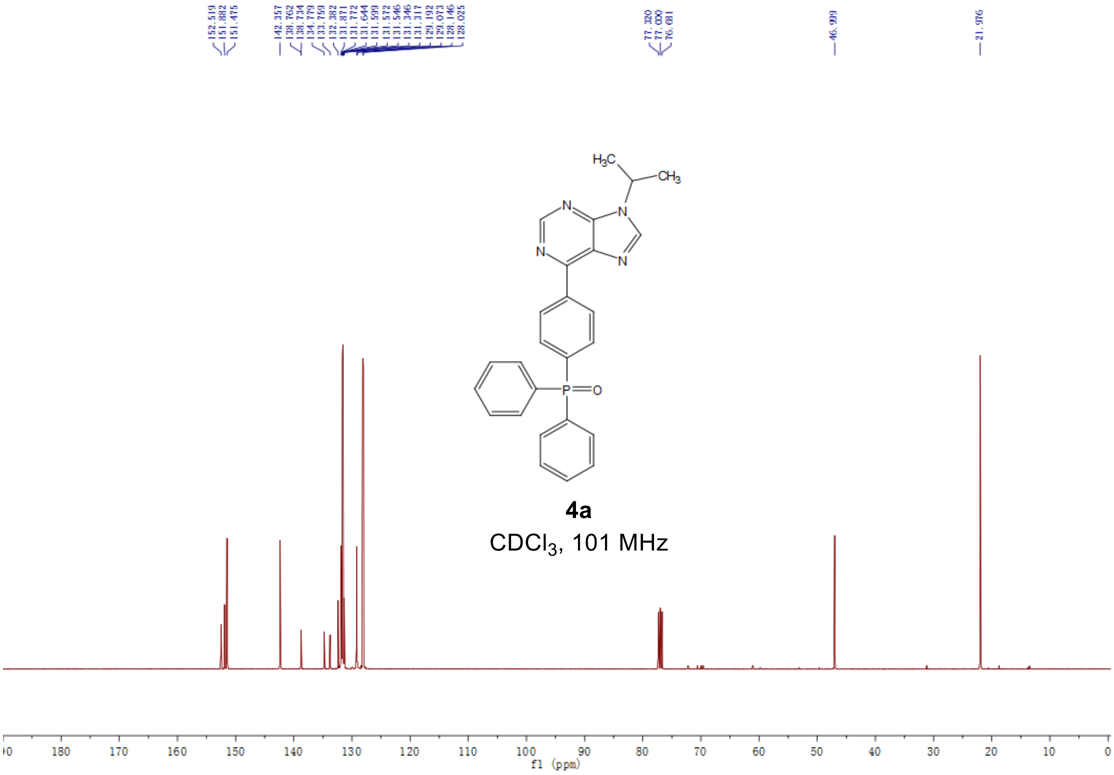

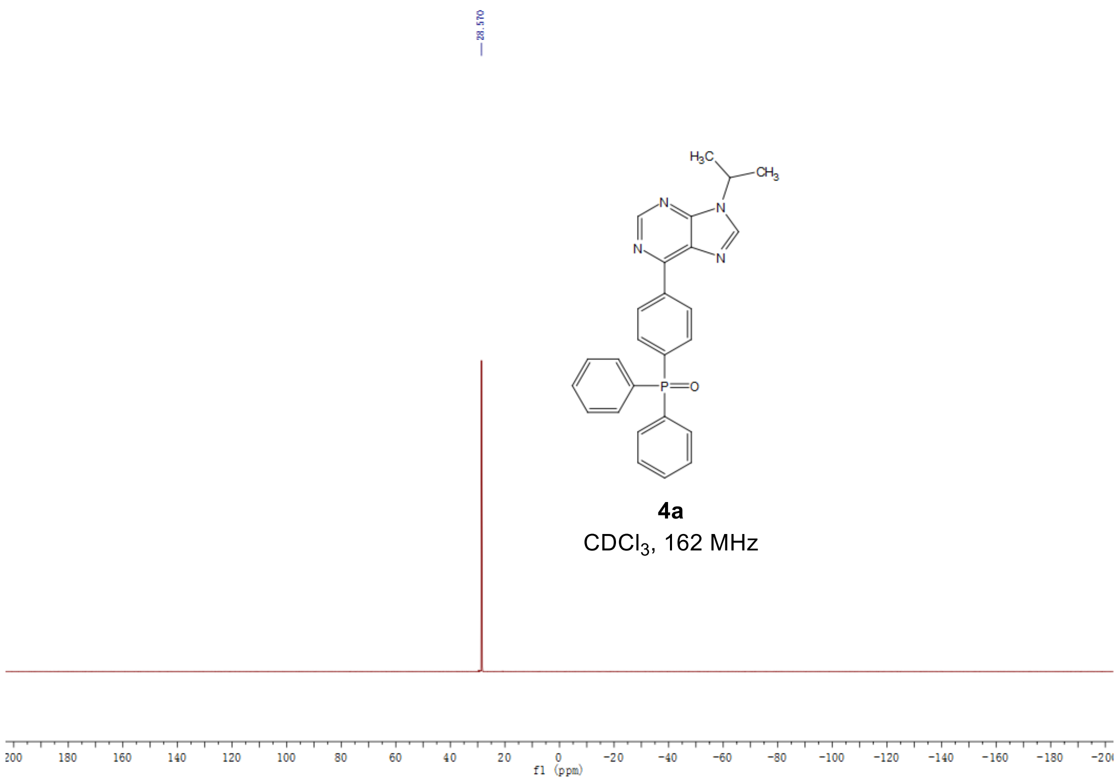

(4-(9-Methyl-9H-purin-6-yl)phenyl)diphenylphosphine oxide (**4b**)

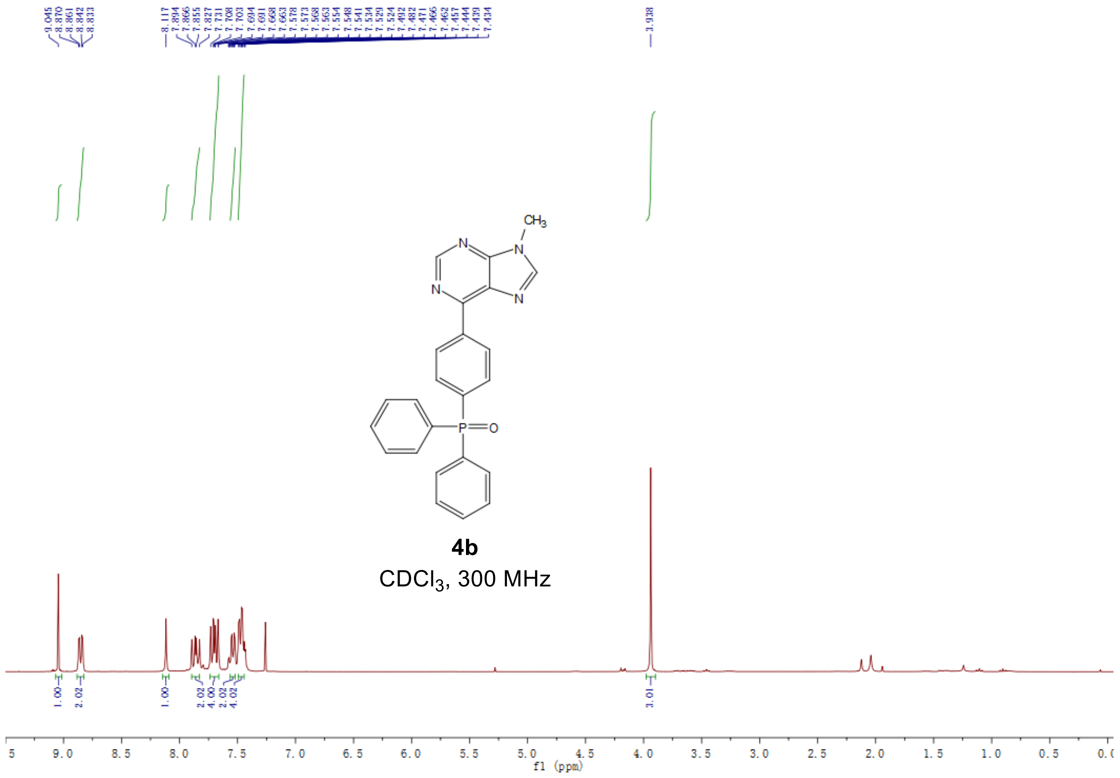

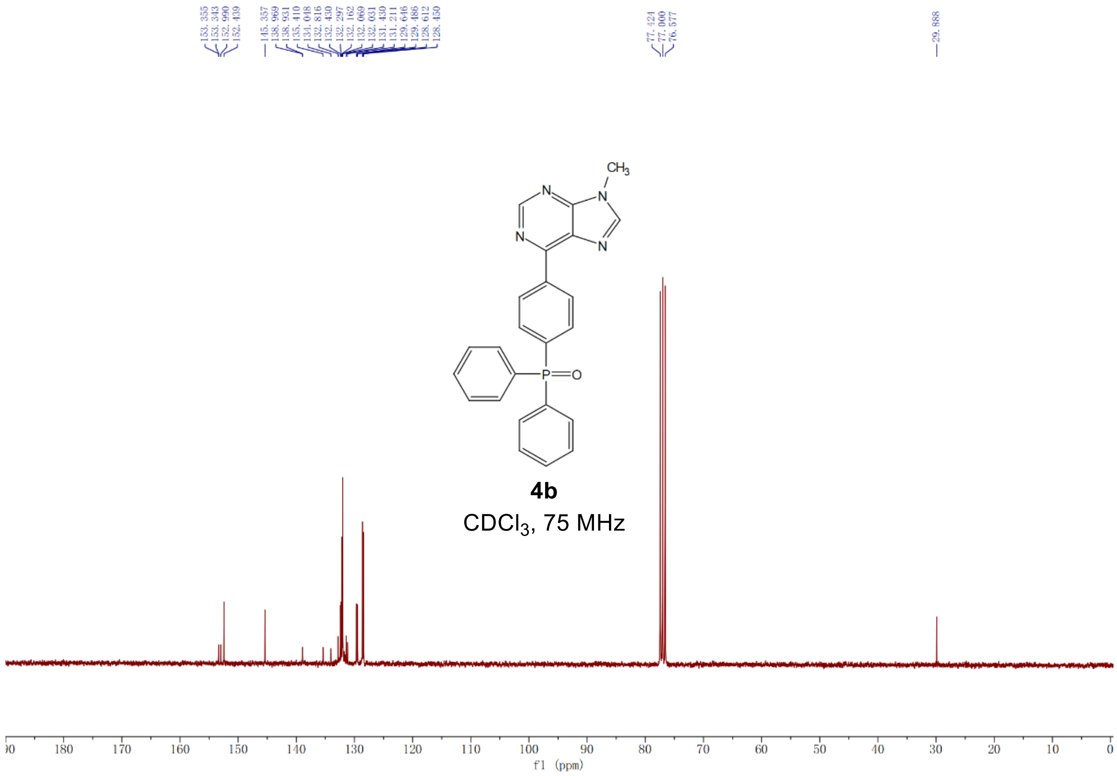
153.355
153.343
152.990
152.439
145.357
138.969
138.931
135.410
134.048
132.816
132.430
132.297
132.162
132.069
132.031
131.430
131.211
129.646
129.486
128.612
128.450
77.424
77.000
76.577
29.888
CH3
N
N
N
N
P
O
4b
CDCl3, 75 MHz
f1 (ppm)

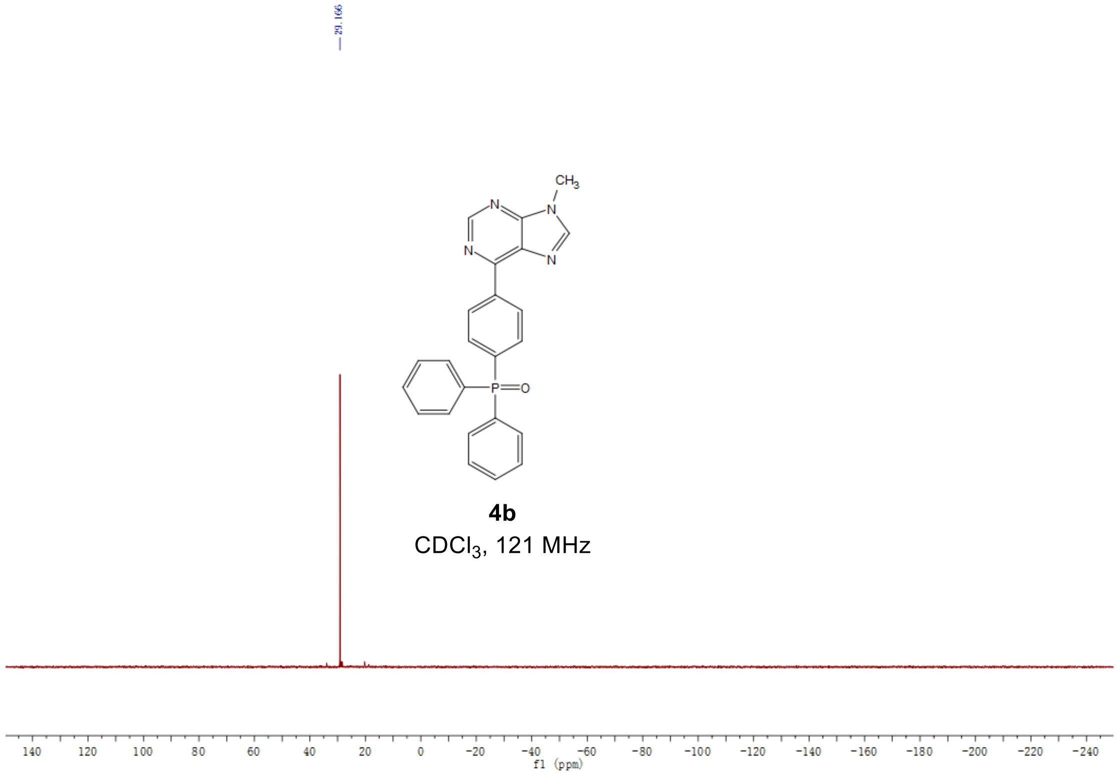
29.166
CH3
N
N
N
N
P
O
4b
CDCl3, 121 MHz
f1 (ppm)

(4-(9-Benzyl-9H-purin-6-yl)phenyl)diphenylphosphine oxide (**4c**)

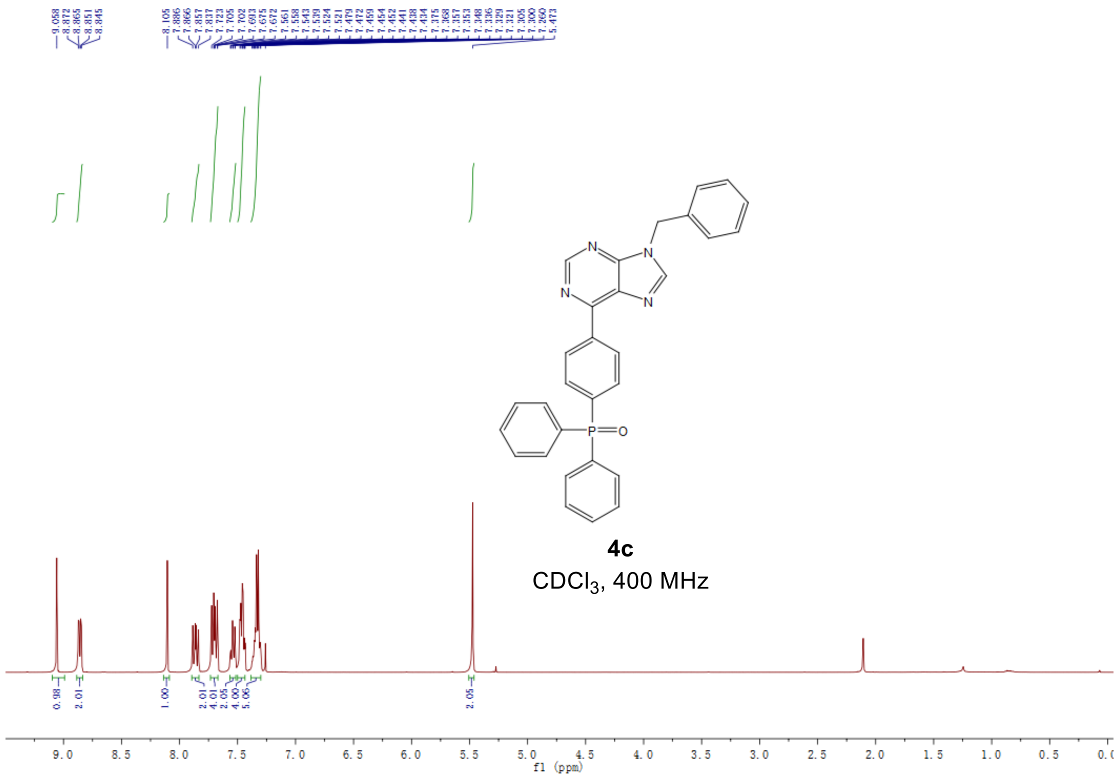

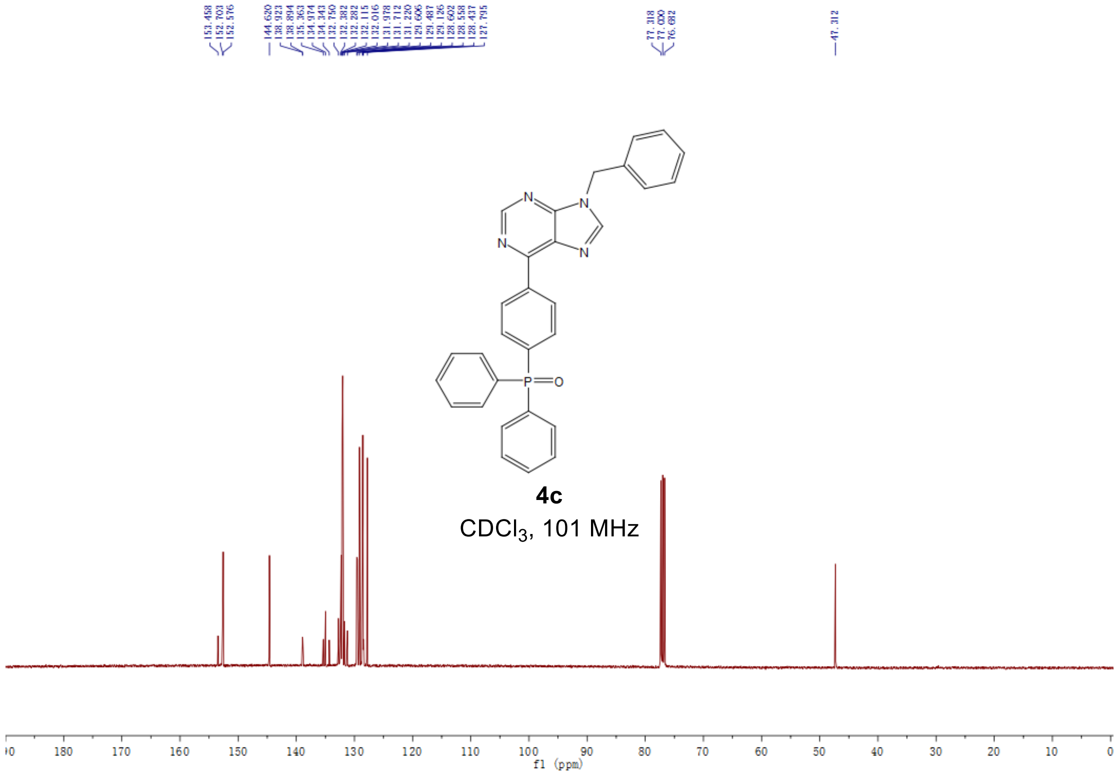

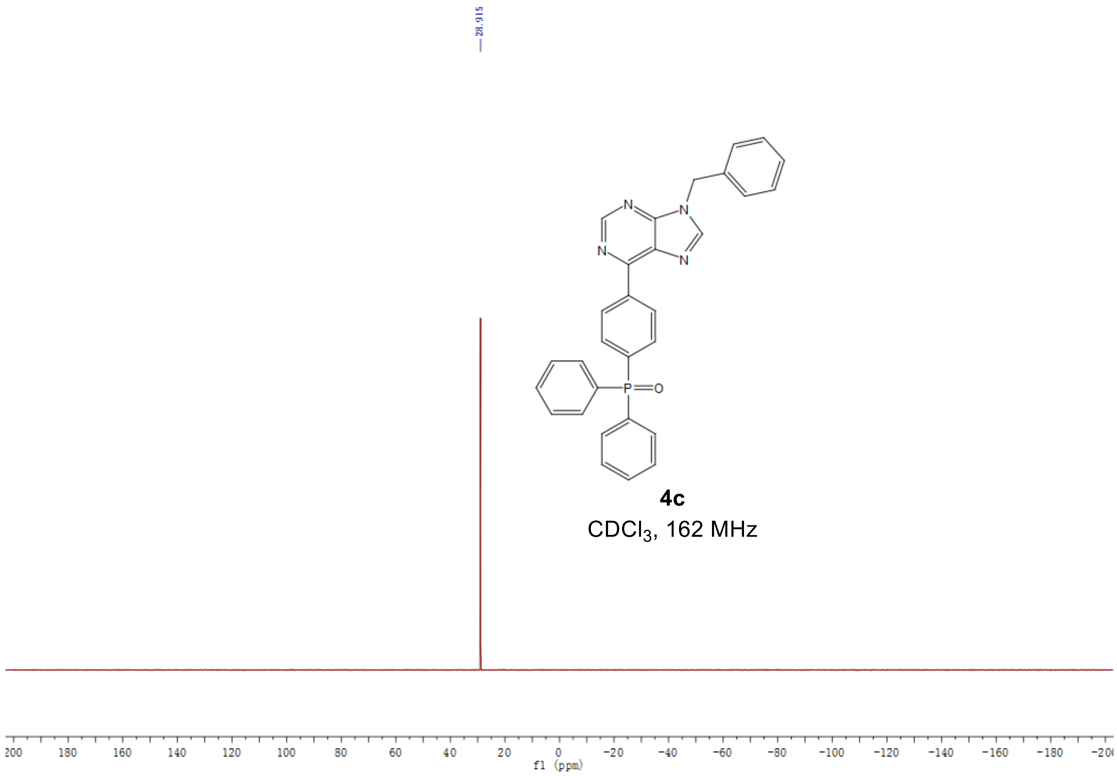

(2R,3R,4R,5R)-2-(Acetoxymethyl)-5-(6-(4-(diphenylphosphoryl)phenyl)-9H-purin-9-yl)tetrahydrofuran-3,4-diyl diacetate (**4d**)

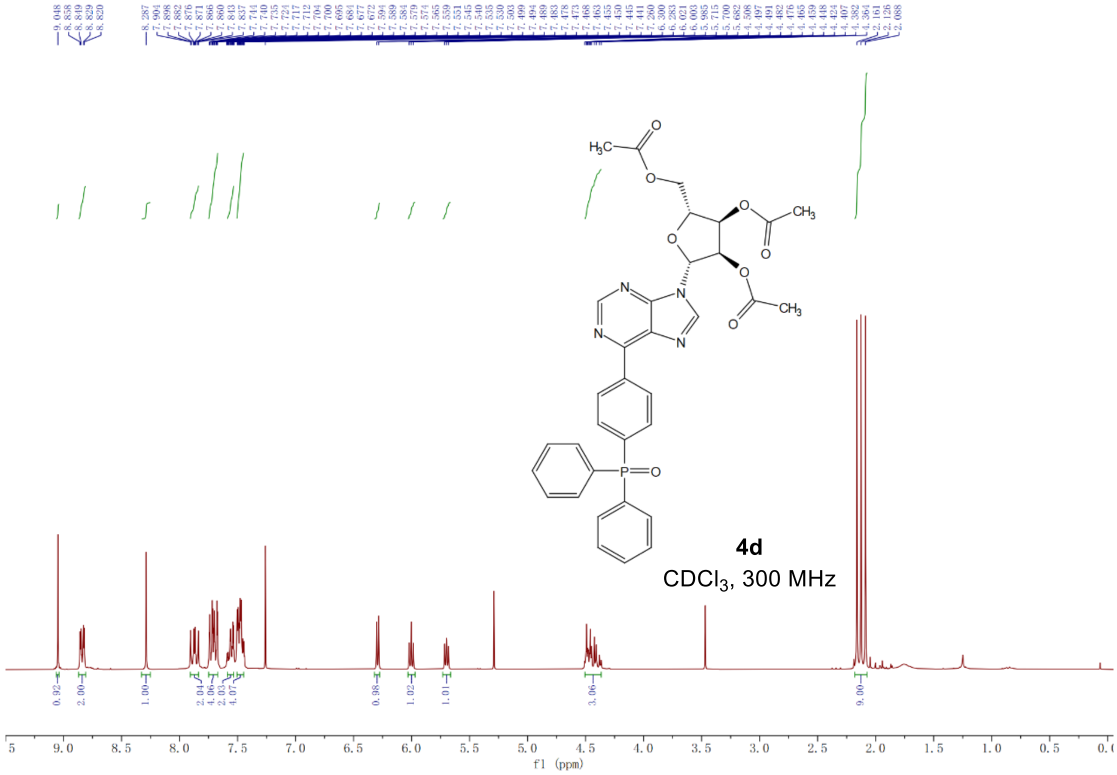

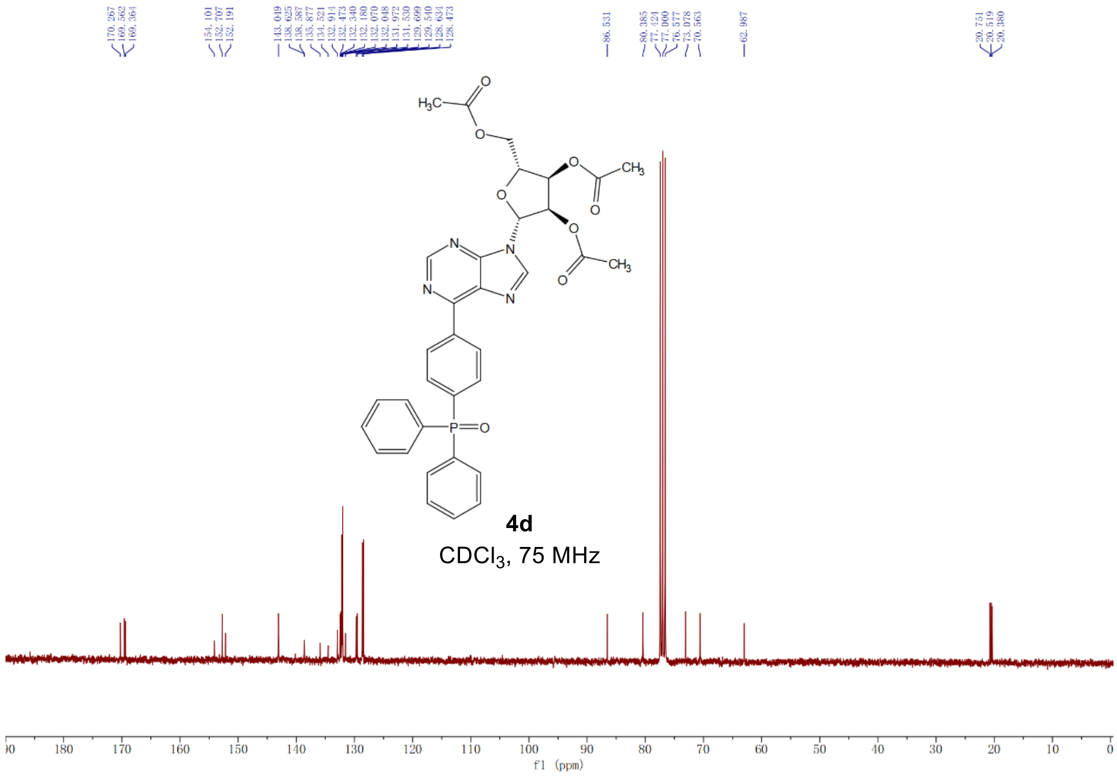
170.267
169.562
169.364
154.101
152.707
152.191
143.049
138.625
138.587
135.877
134.521
132.914
132.473
132.340
132.180
132.070
132.048
131.672
131.530
129.699
129.540
128.634
128.473
86.531
80.385
77.424
77.000
76.577
73.078
70.563
62.987
20.751
20.519
20.380
4d
CDCl3, 75 MHz
f1 (ppm)

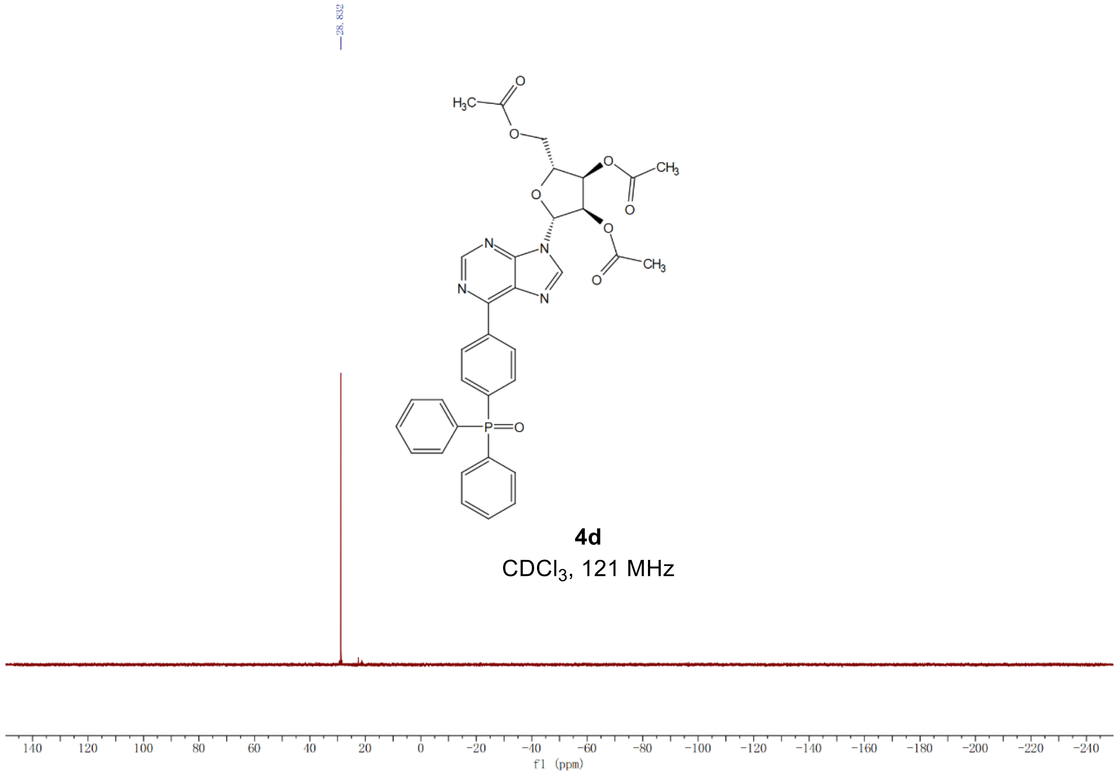
28.832
4d
CDCl3, 121 MHz
f1 (ppm)

(2-Fluoro-4-(9-isopropyl-9H-purin-6-yl)phenyl)diphenylphosphine oxide (**4e**)

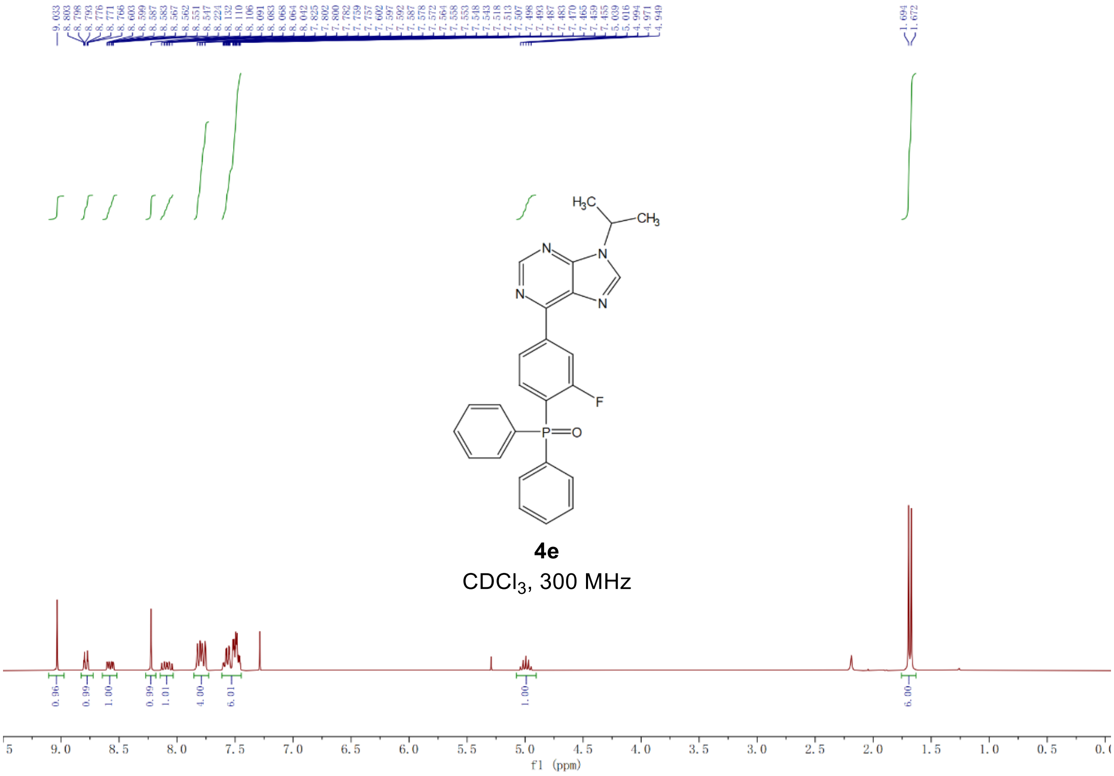

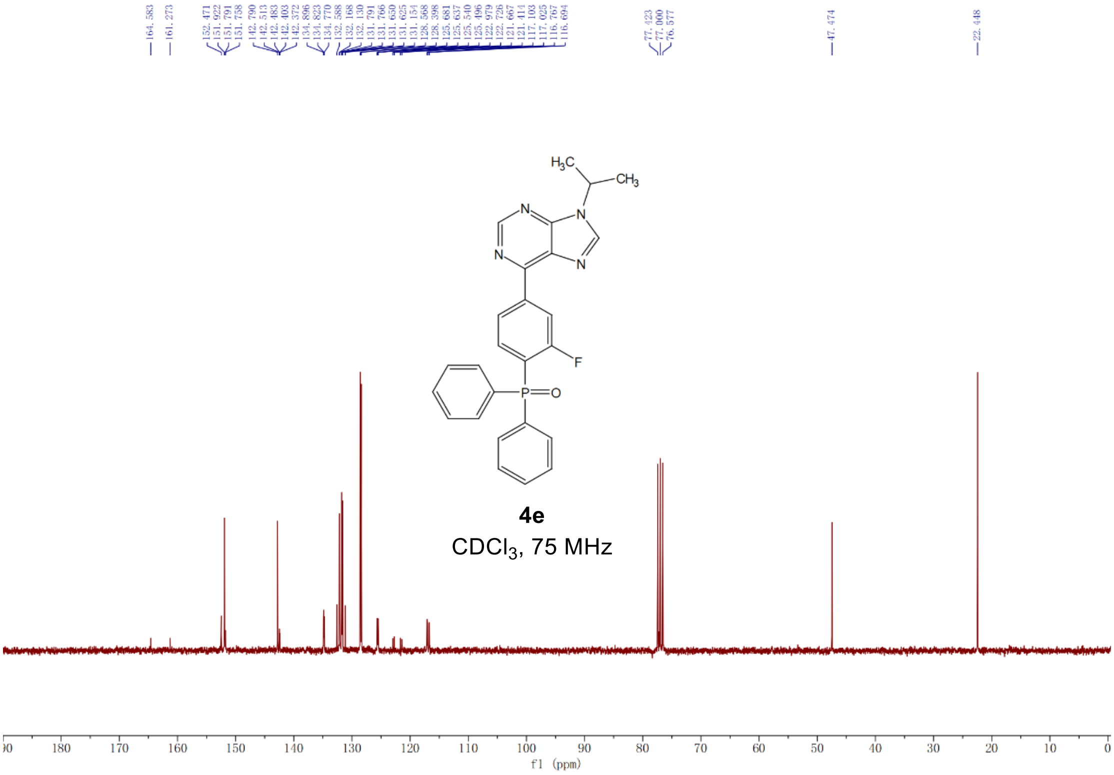

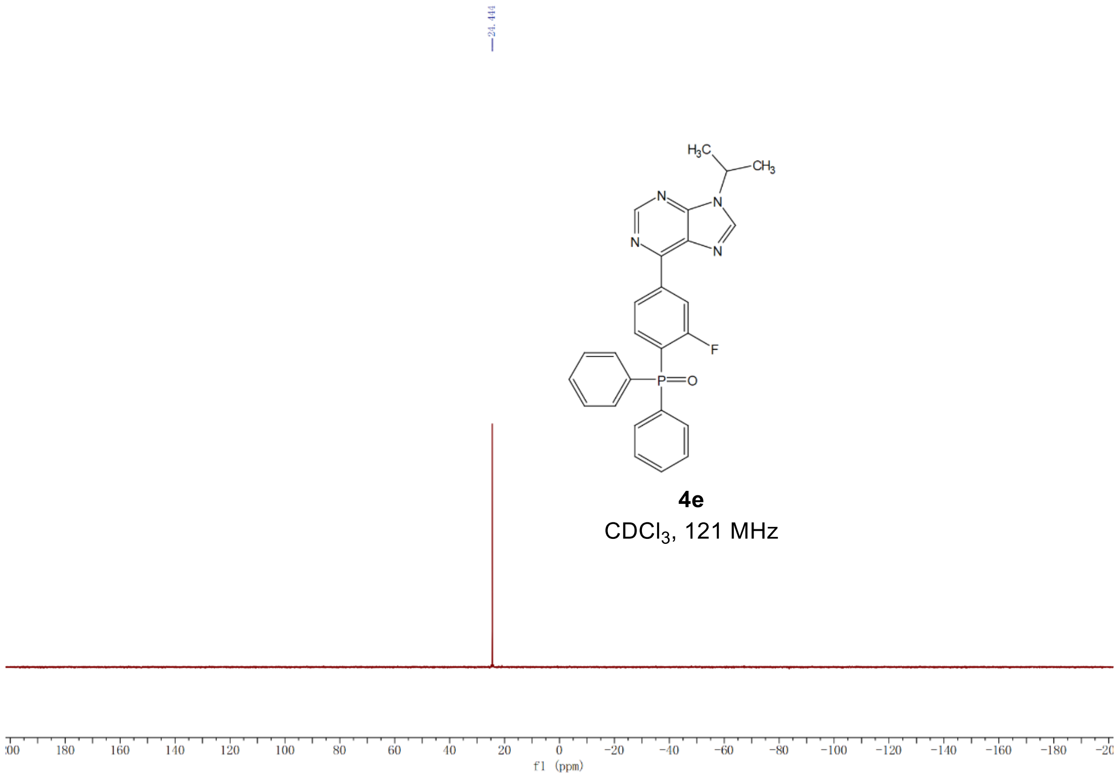
24.444
H3C
CH3
N
N
N
N
F
P
O
4e
CDCl3, 121 MHz
f1 (ppm)

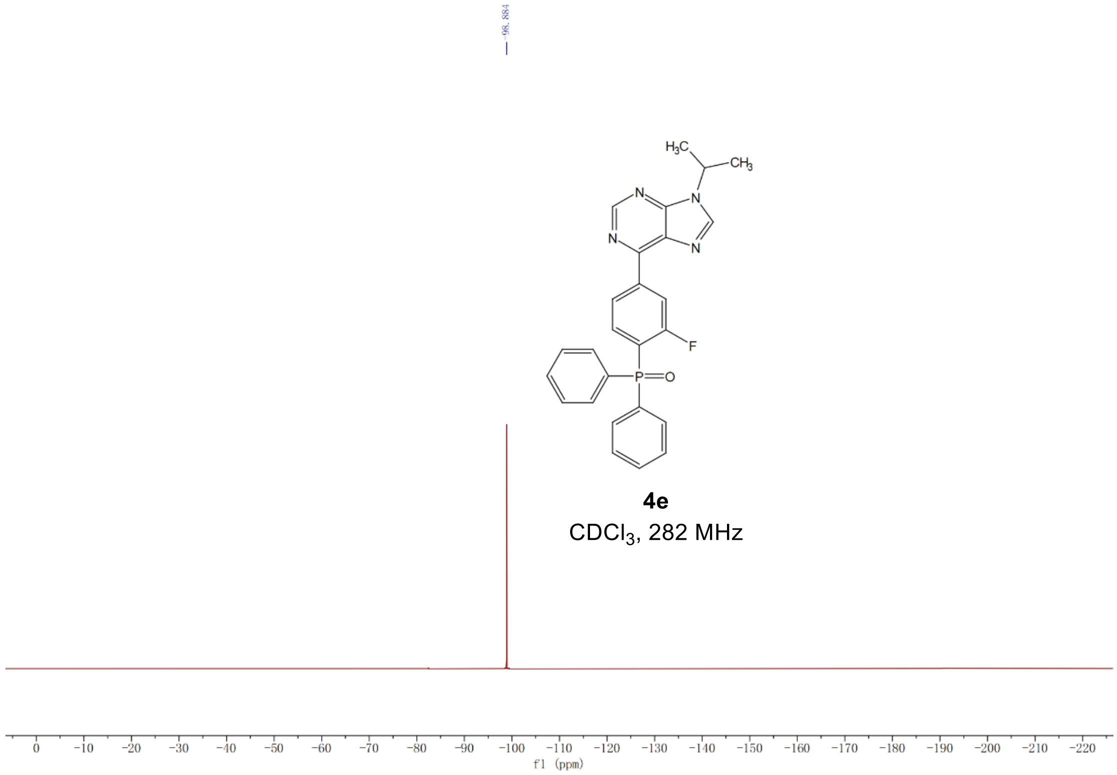
-98.884
H3C
CH3
N
N
N
N
F
P
O
4e
CDCl3, 282 MHz
f1 (ppm)

(4-(9-Isopropyl-9H-purin-6-yl)-2-methylphenyl)diphenylphosphine oxide (**4f**)

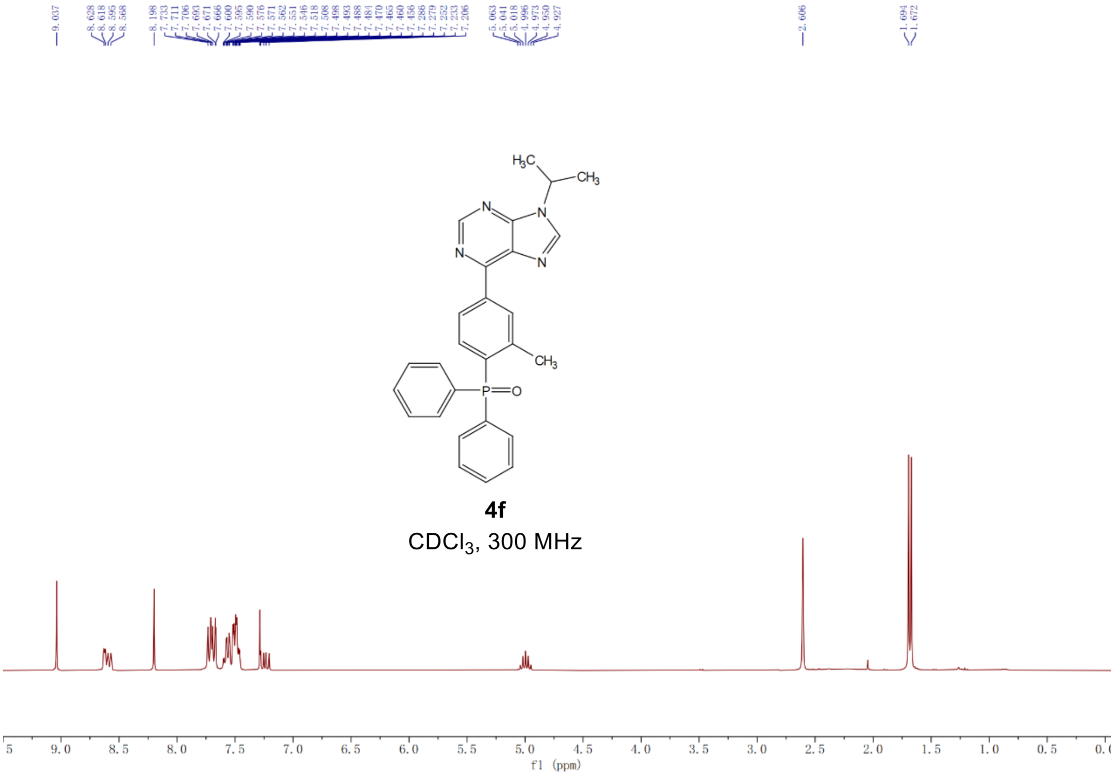

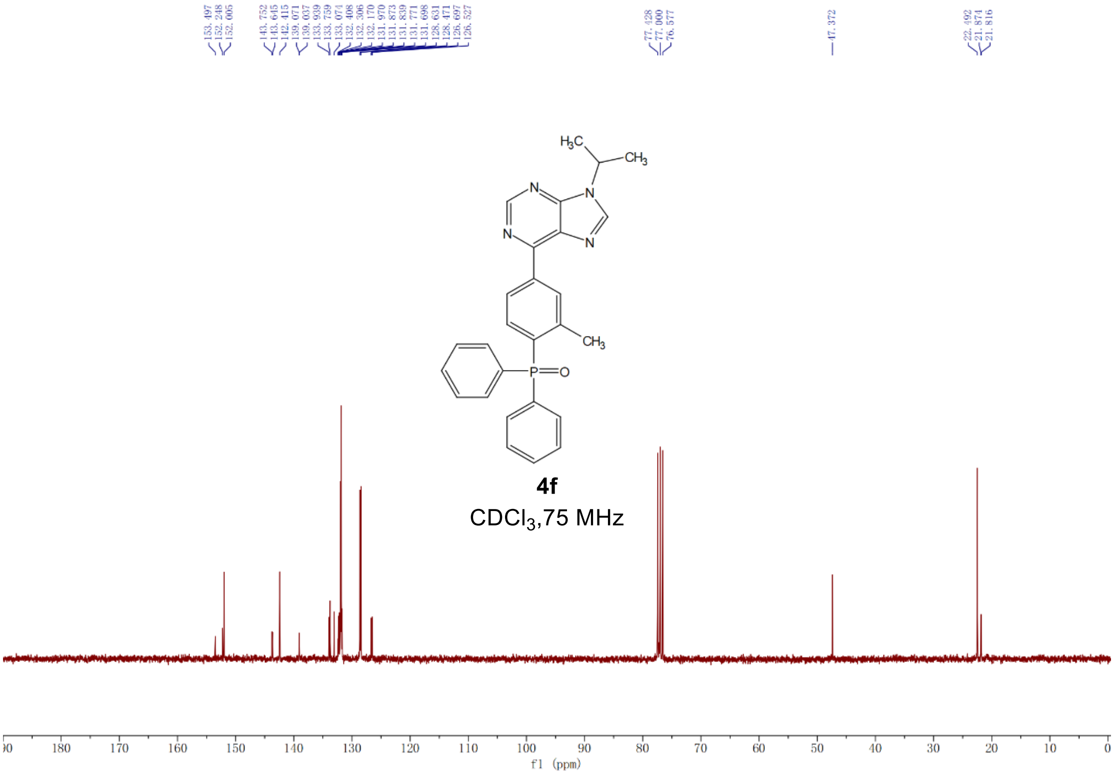

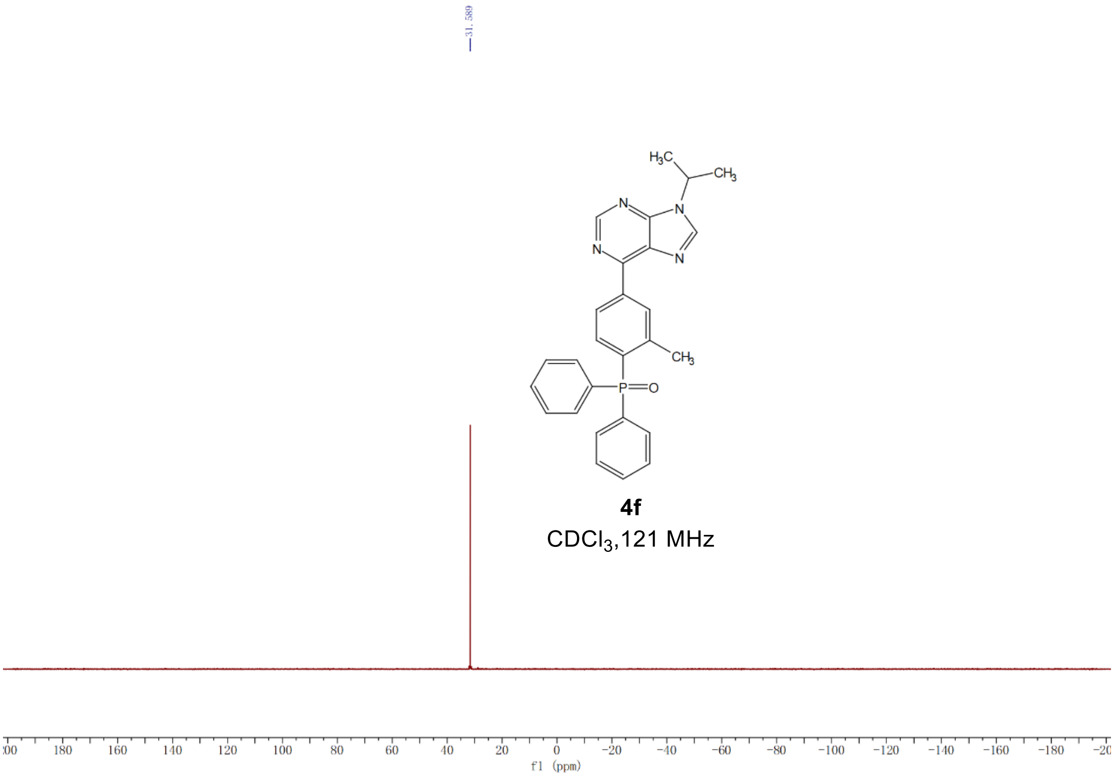

Bis(4-chlorophenyl)(4-(9-isopropyl-9H-purin-6-yl)phenyl)phosphine oxide (**4g**)

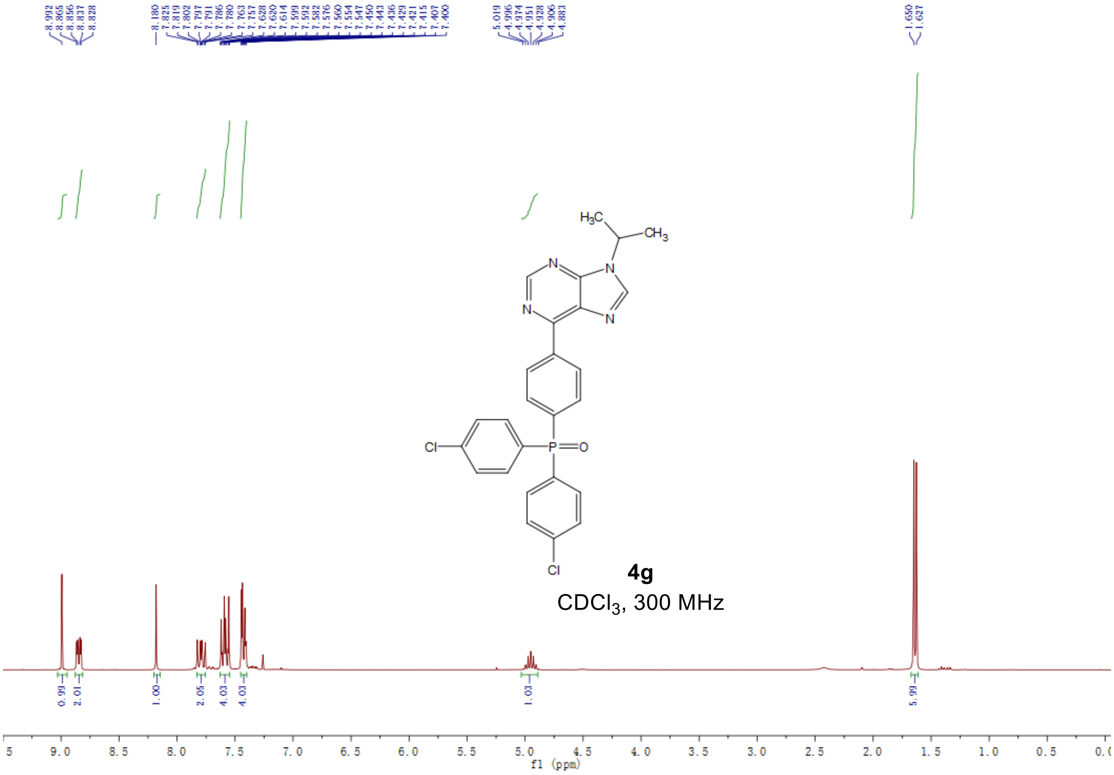

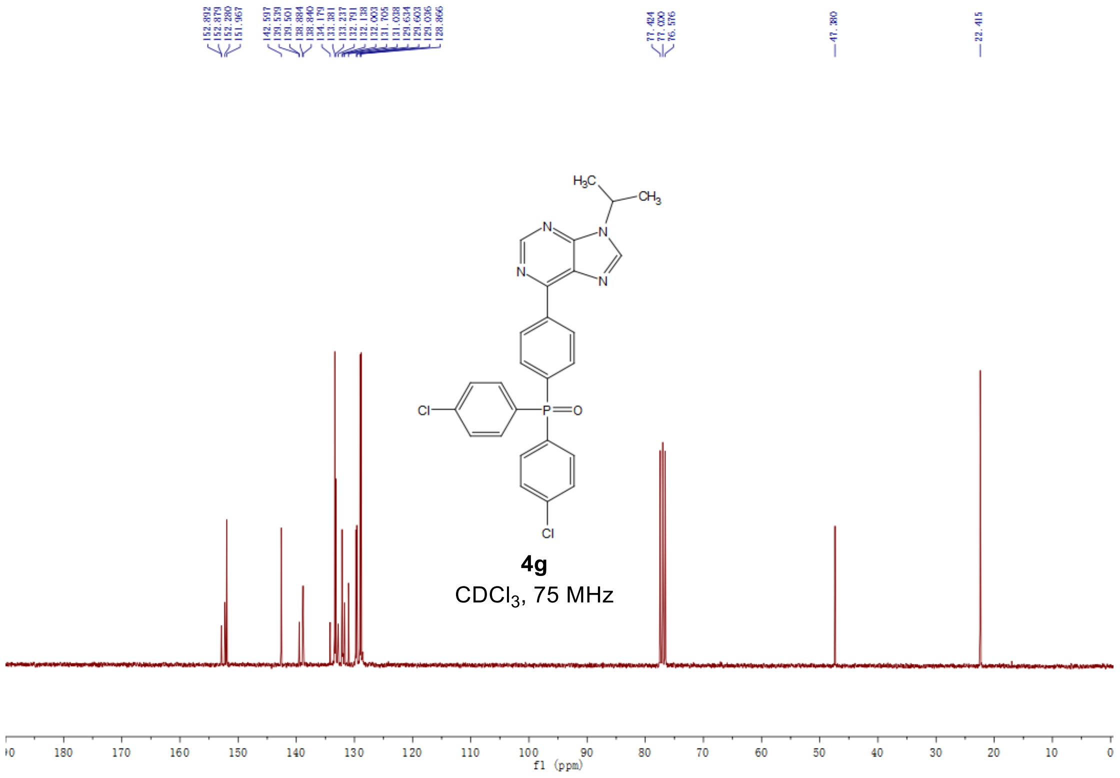

4g

$CDCl_3$, 75 MHz

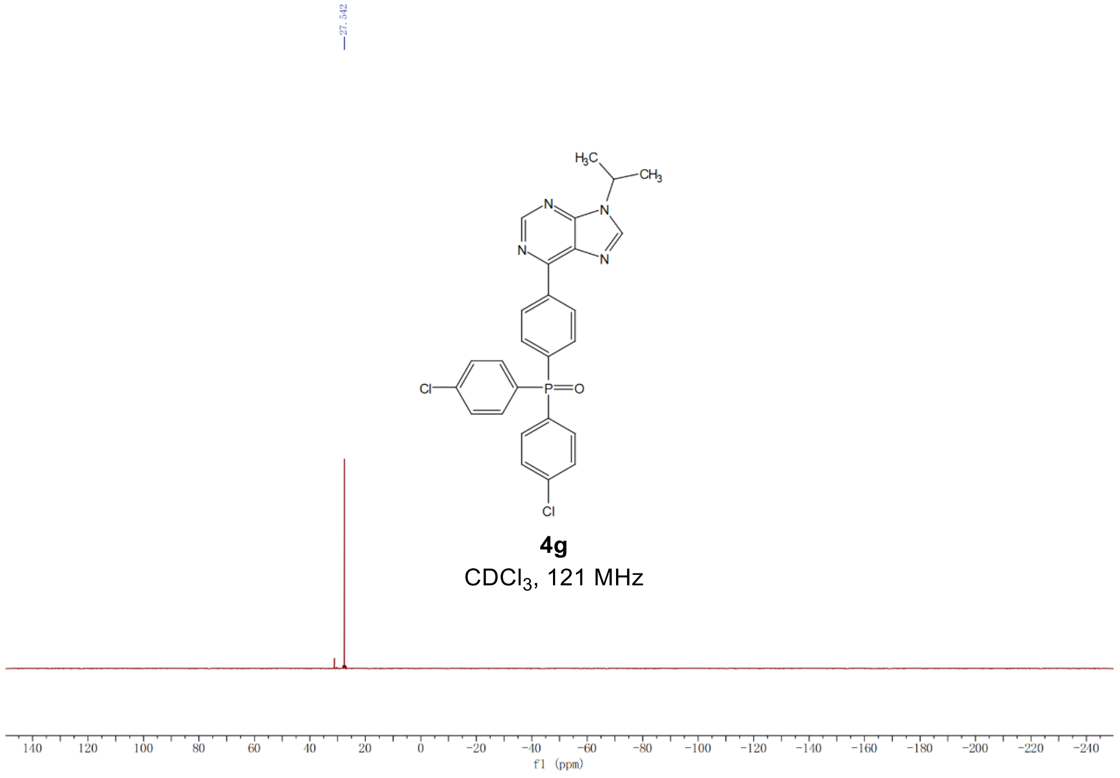

4g

$CDCl_3$, 121 MHz

(4-(9-Isopropyl-9H-purin-6-yl)phenyl)di-p-tolylphosphine oxide (**4h**)

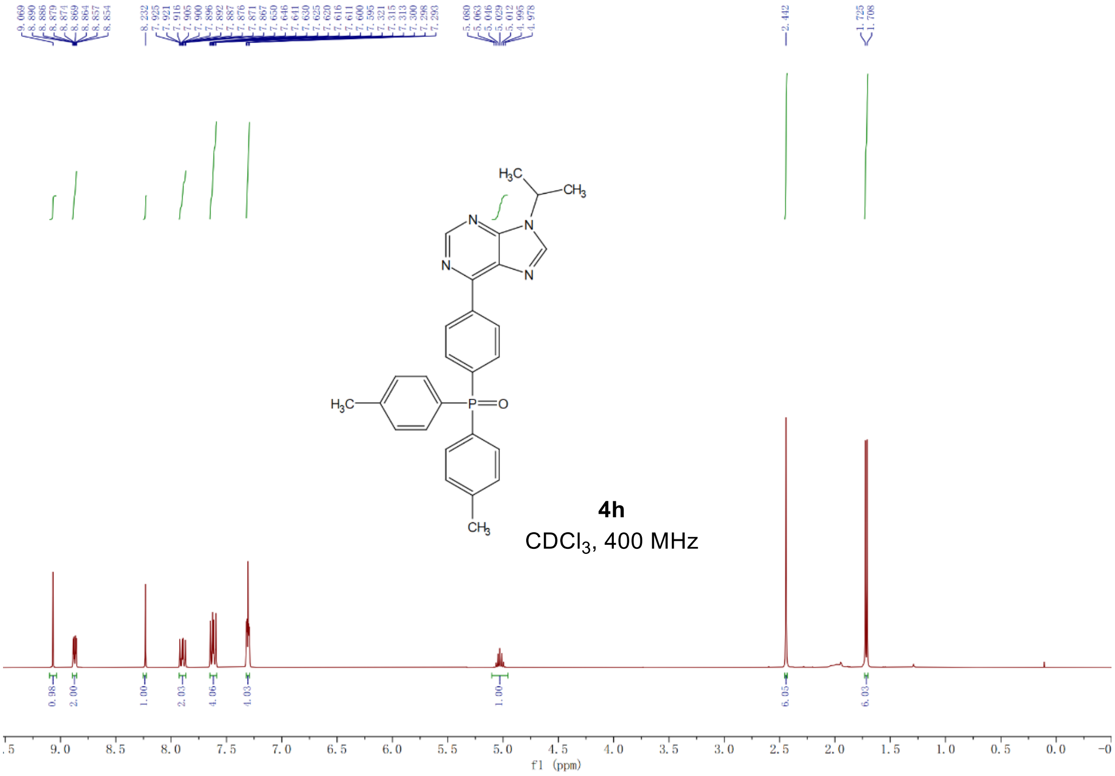

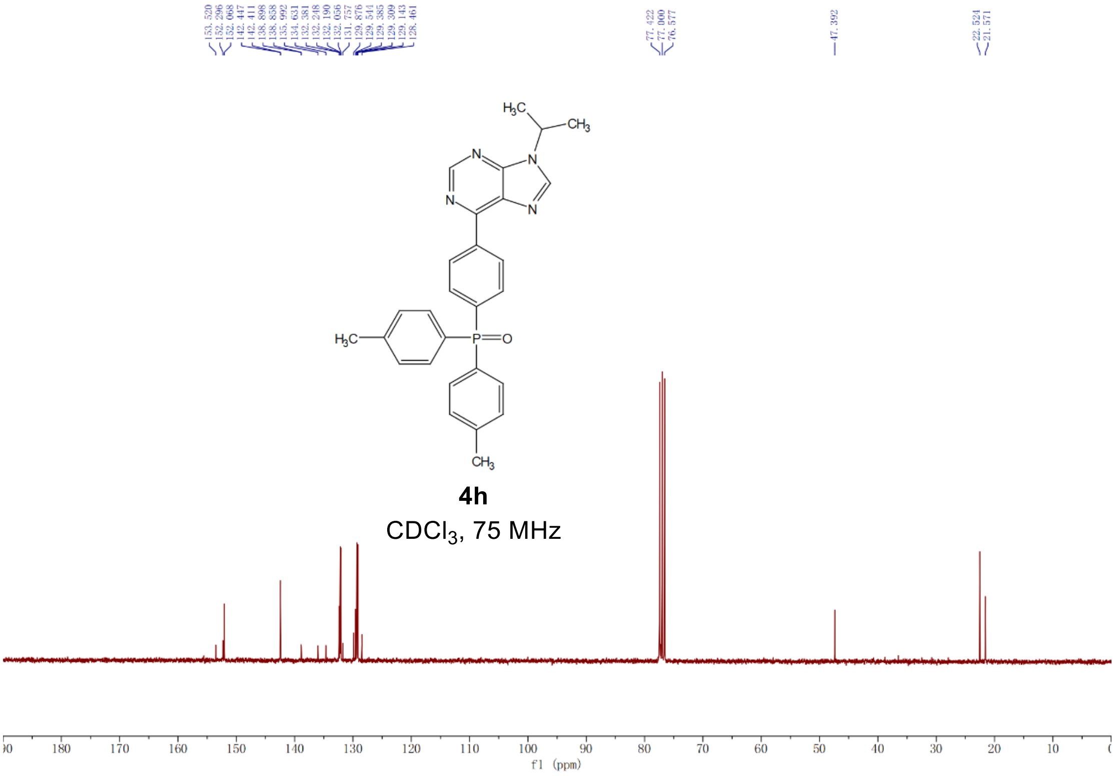

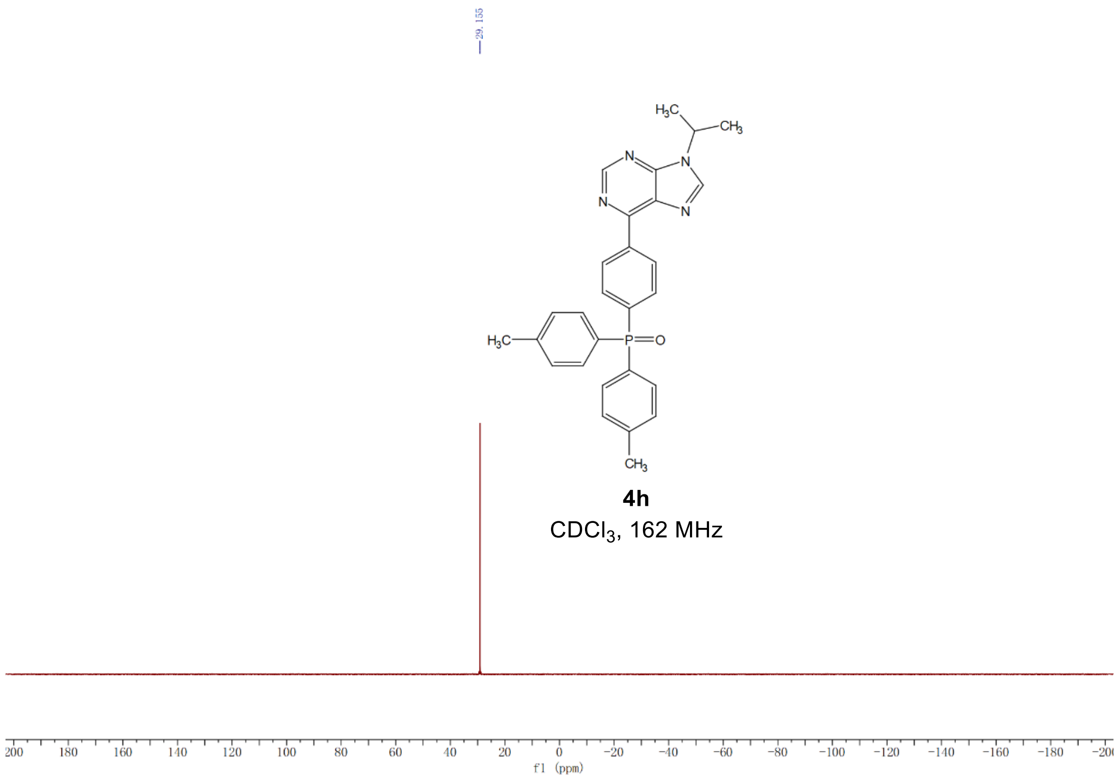

(4-(9-Isopropyl-9H-purin-6-yl)phenyl)bis(4-methoxyphenyl)phosphine oxide (**4i**)

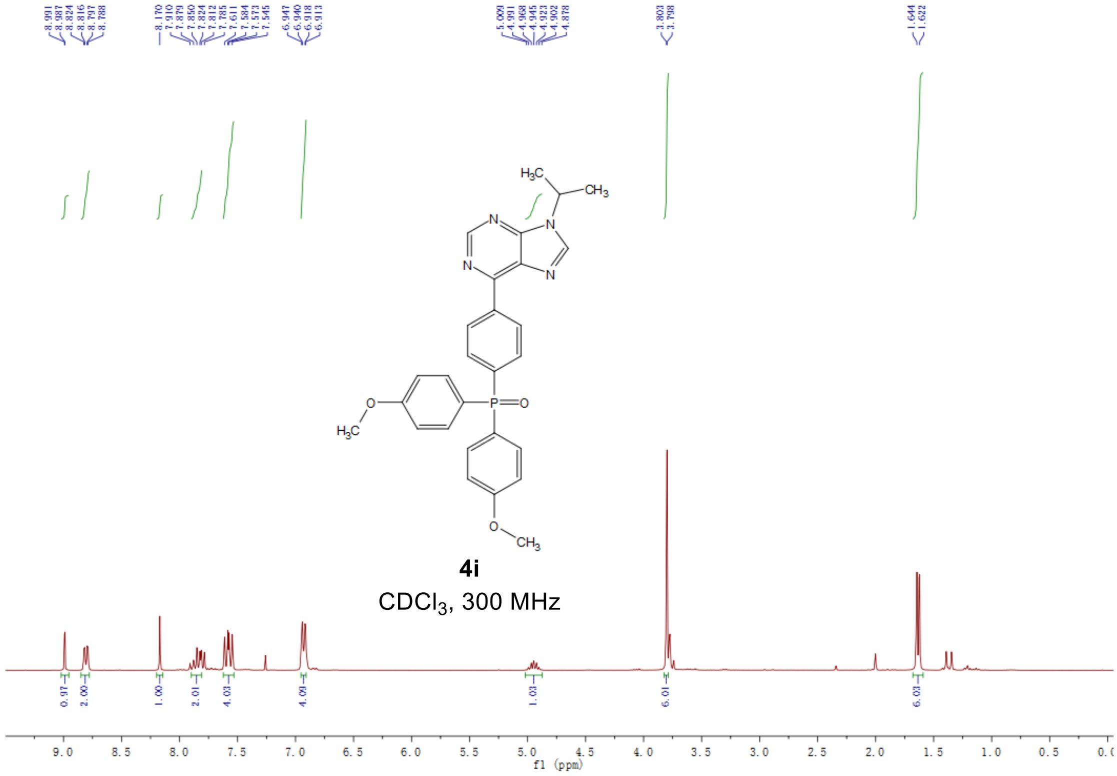

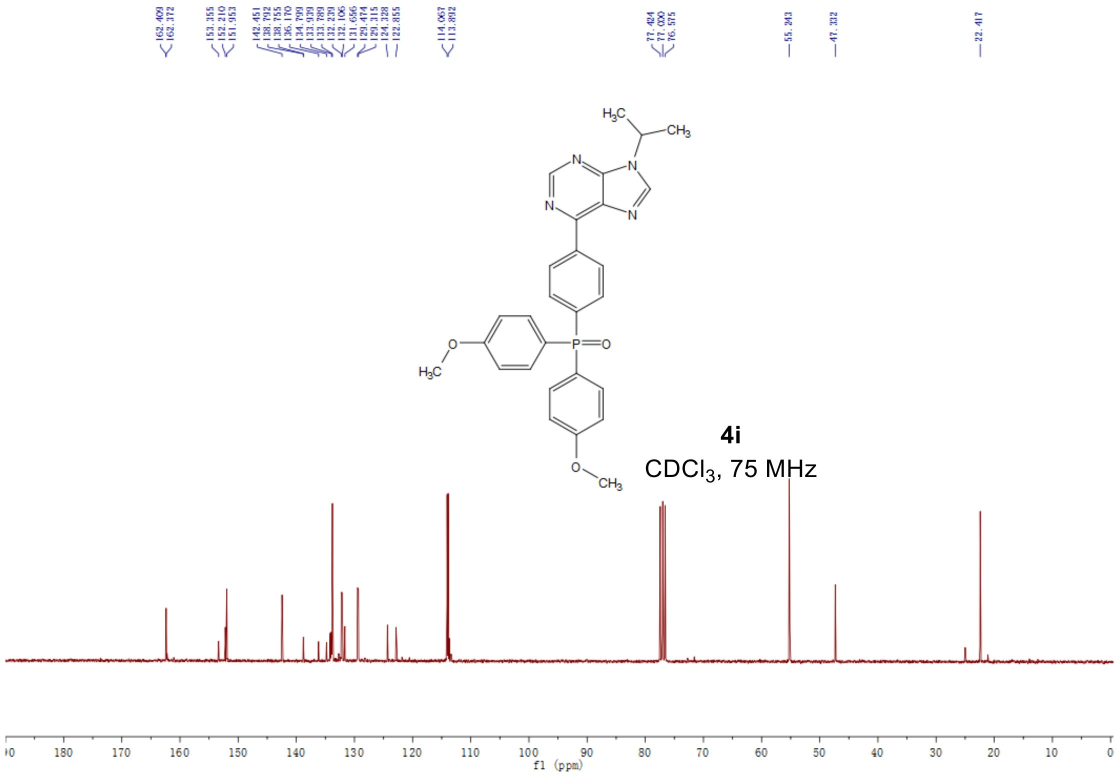
162.409
162.372
153.355
152.210
151.953
142.451
138.792
138.755
136.170
134.799
133.939
133.789
132.239
132.106
131.656
129.474
129.315
124.328
122.855
114.067
113.892
77.424
77.000
76.575
55.243
47.332
22.417
4i
CDCl3, 75 MHz
f1 (ppm)

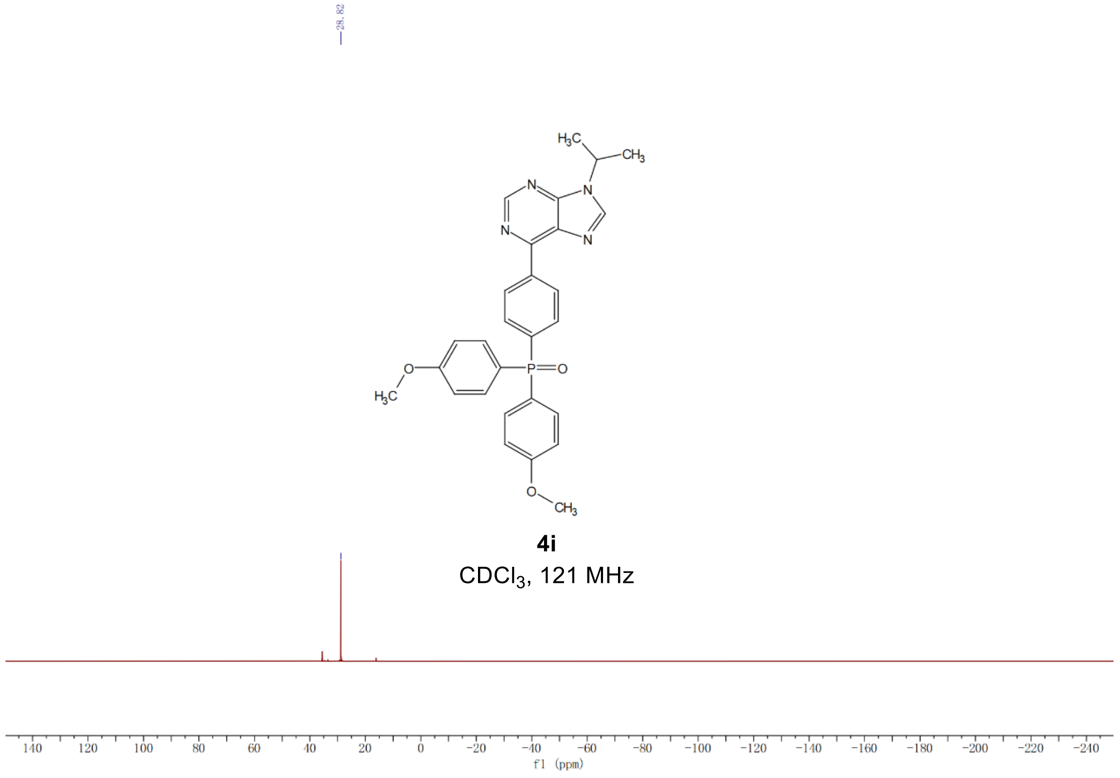
28.82
4i
CDCl3, 121 MHz
f1 (ppm)

(4-(9-Isopropyl-9H-purin-6-yl)phenyl)bis(4-(trifluoromethoxy)phenyl)phosphine oxide (**4j**)

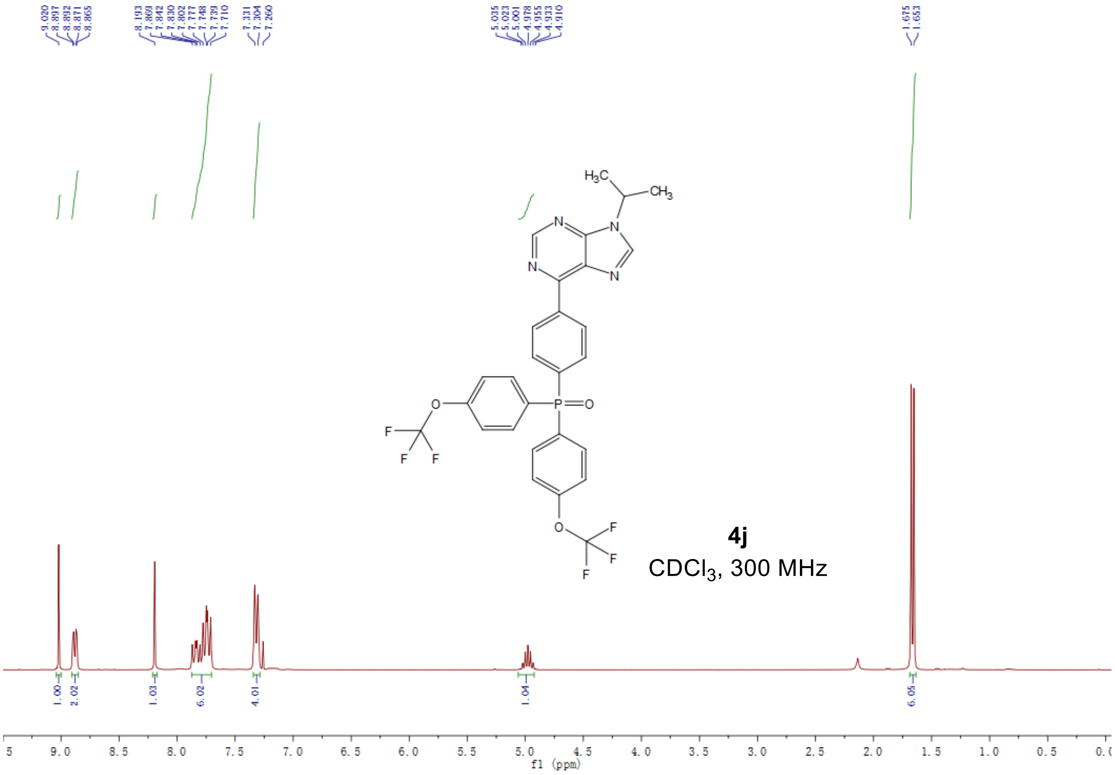

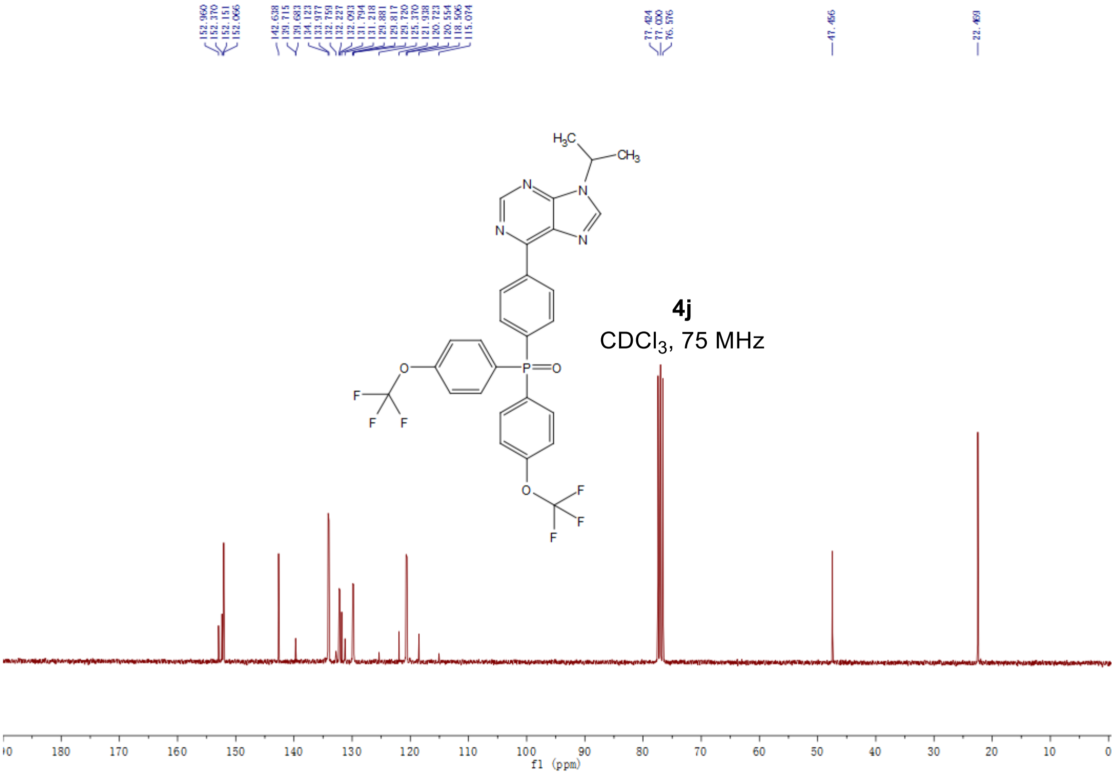

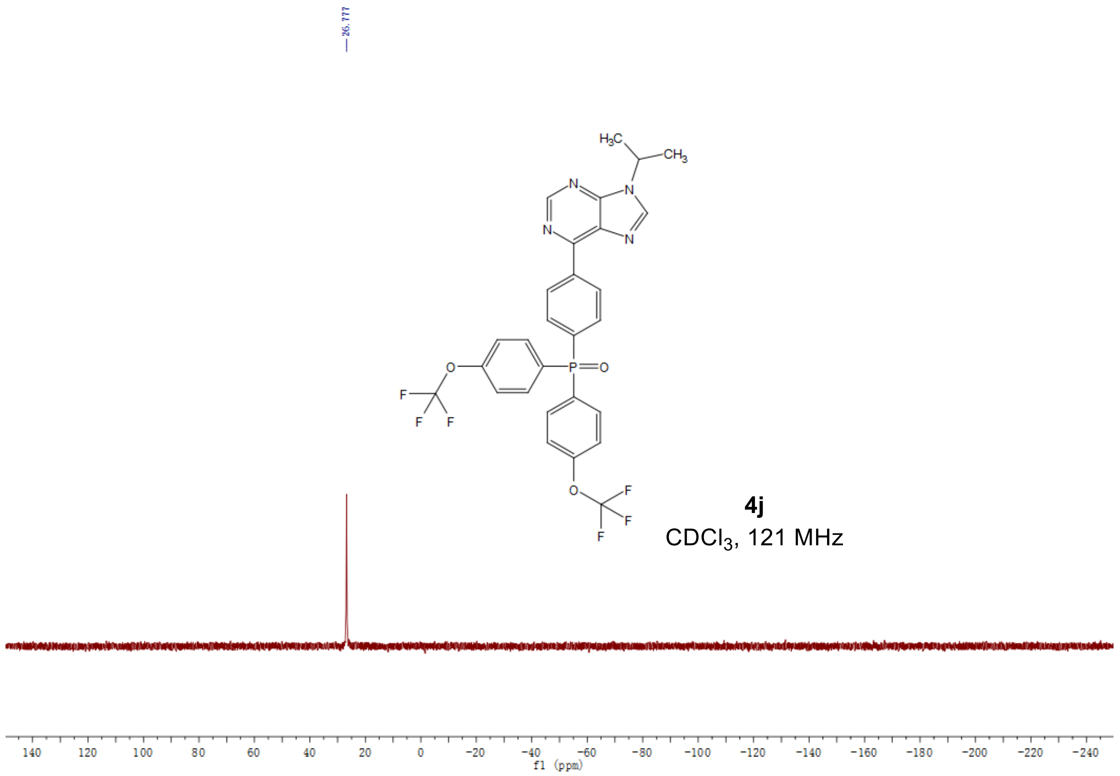

Bis(3,5-dimethylphenyl)(4-(9-isopropyl-9H-purin-6-yl)phenyl)phosphine oxide (**4k**)

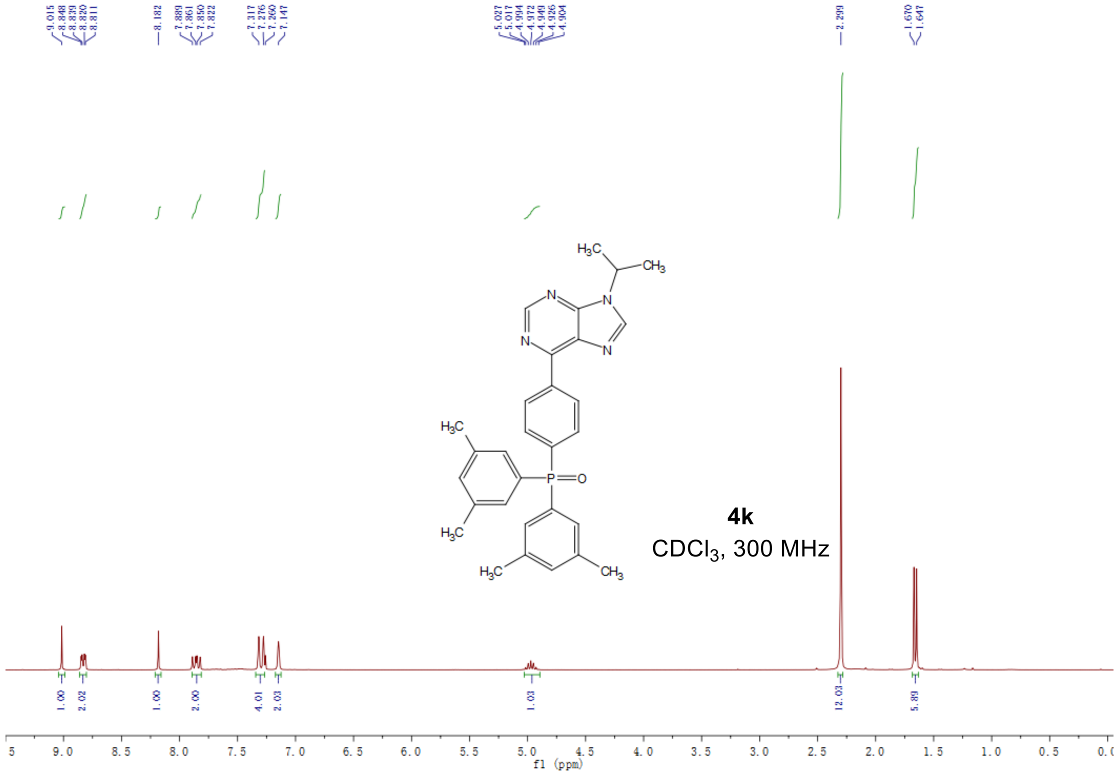

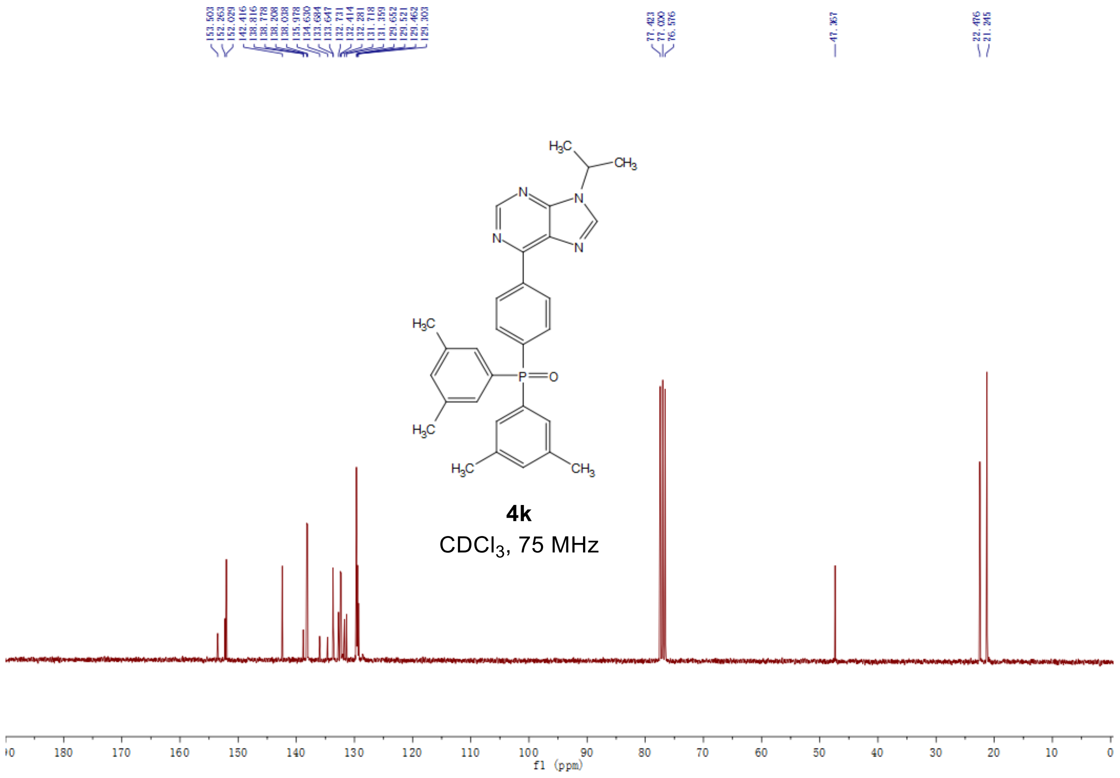

153.503
152.263
152.029
142.416
138.816
138.778
138.208
138.038
135.978
134.630
133.684
133.647
132.731
132.414
132.281
131.718
131.359
129.652
129.521
129.462
129.303
77.423
77.000
76.576
47.367
22.476
21.245
4k
CDCl3, 75 MHz
180
170
160
150
140
130
120
110
100
90
80
70
60
50
40
30
20
10
0
f1 (ppm)

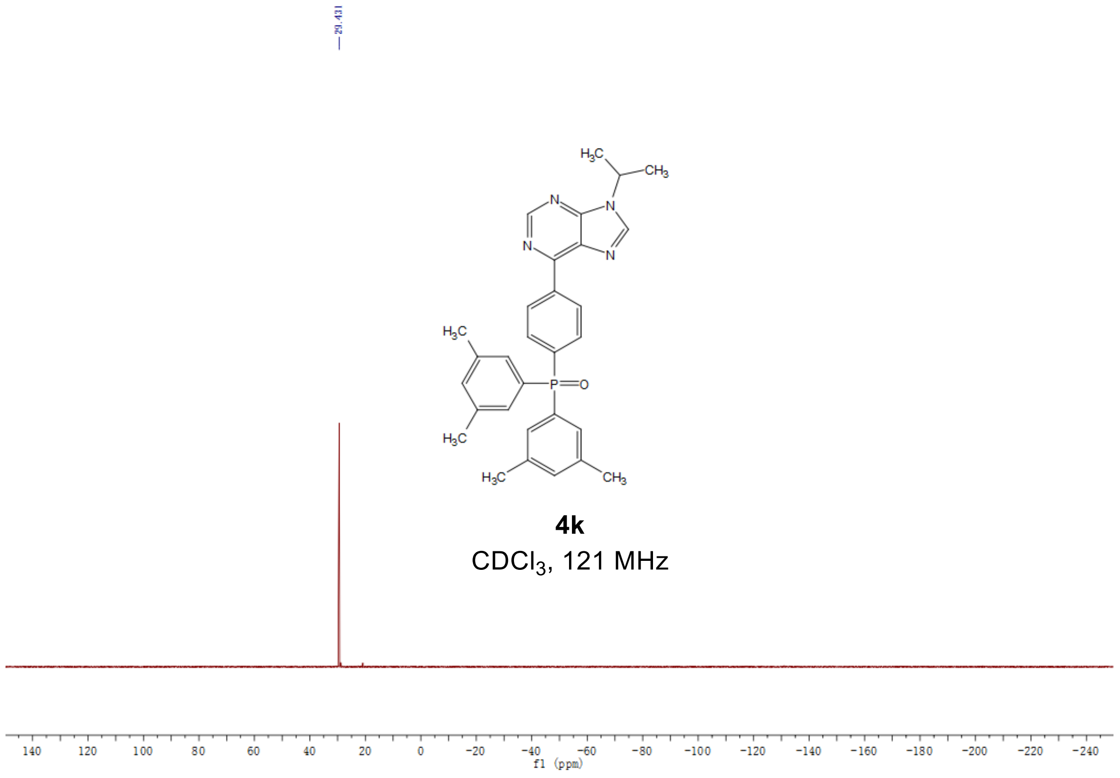

29.431
4k
CDCl3, 121 MHz
140
120
100
80
60
40
20
0
-20
-40
-60
-80
-100
-120
-140
-160
-180
-200
-220
-240
f1 (ppm)

(E)-(4-(1-(Methoxyimino)ethyl)phenyl)diphenylphosphine oxide (**4l**)

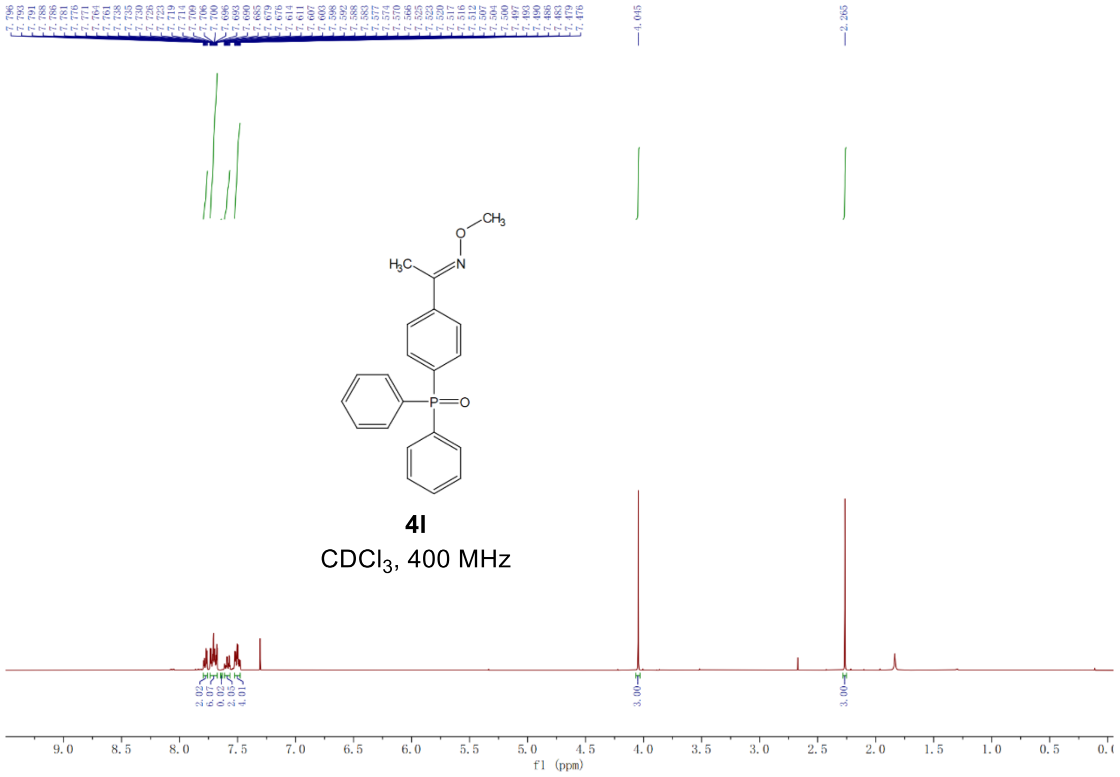

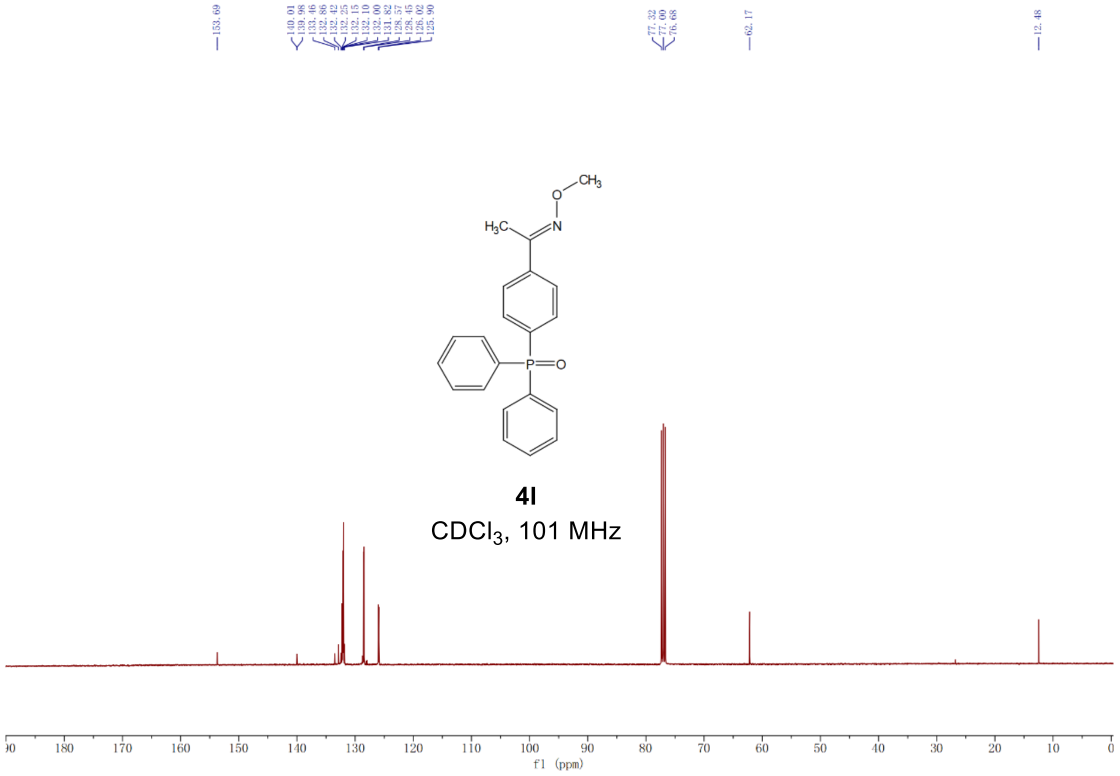

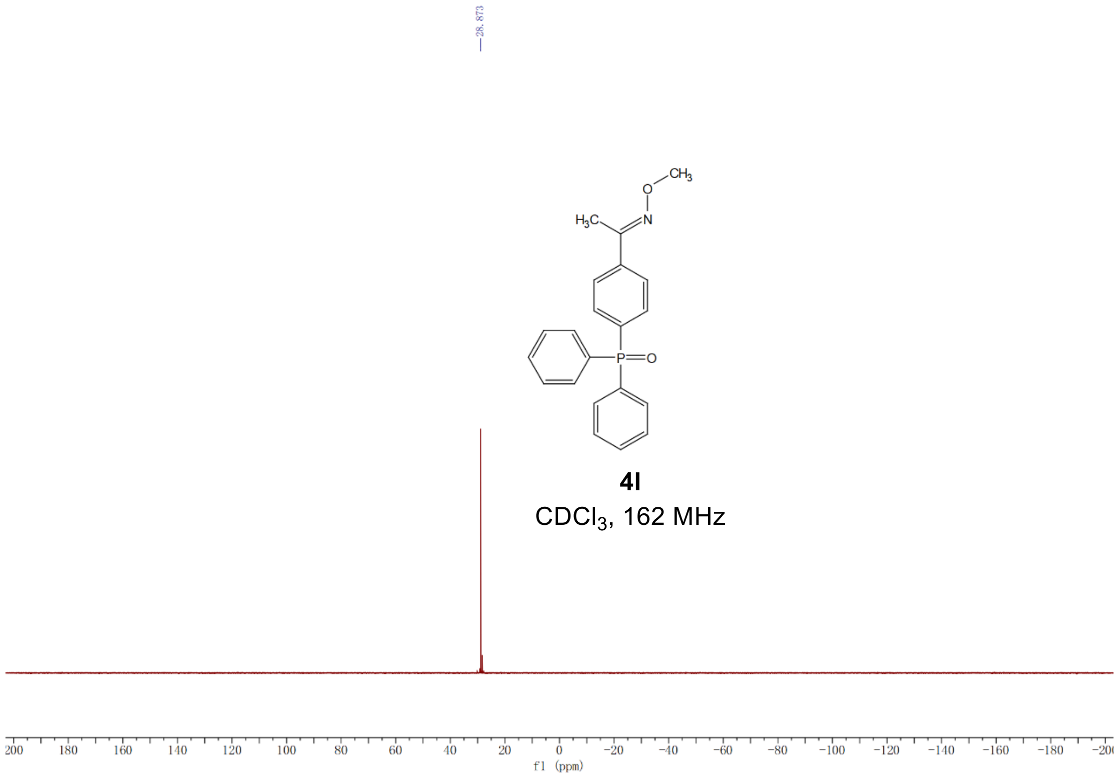

(4-(1H-pyrazol-1-yl)phenyl)diphenylphosphine oxide (**4m**)

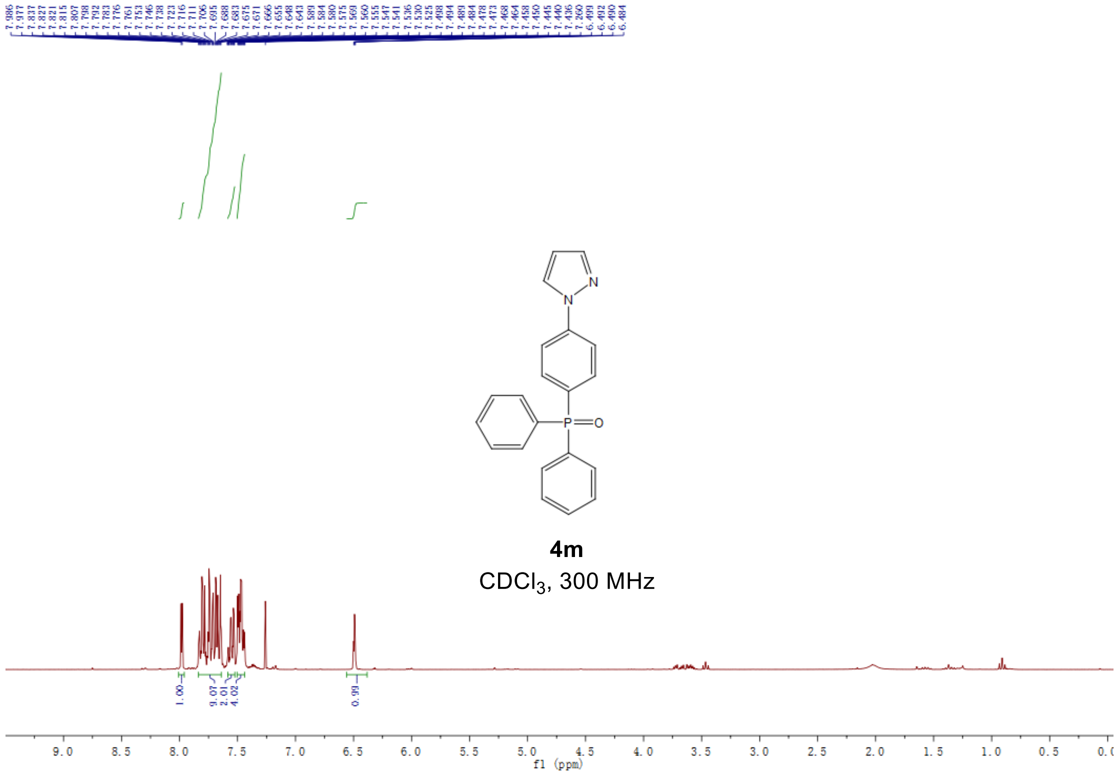

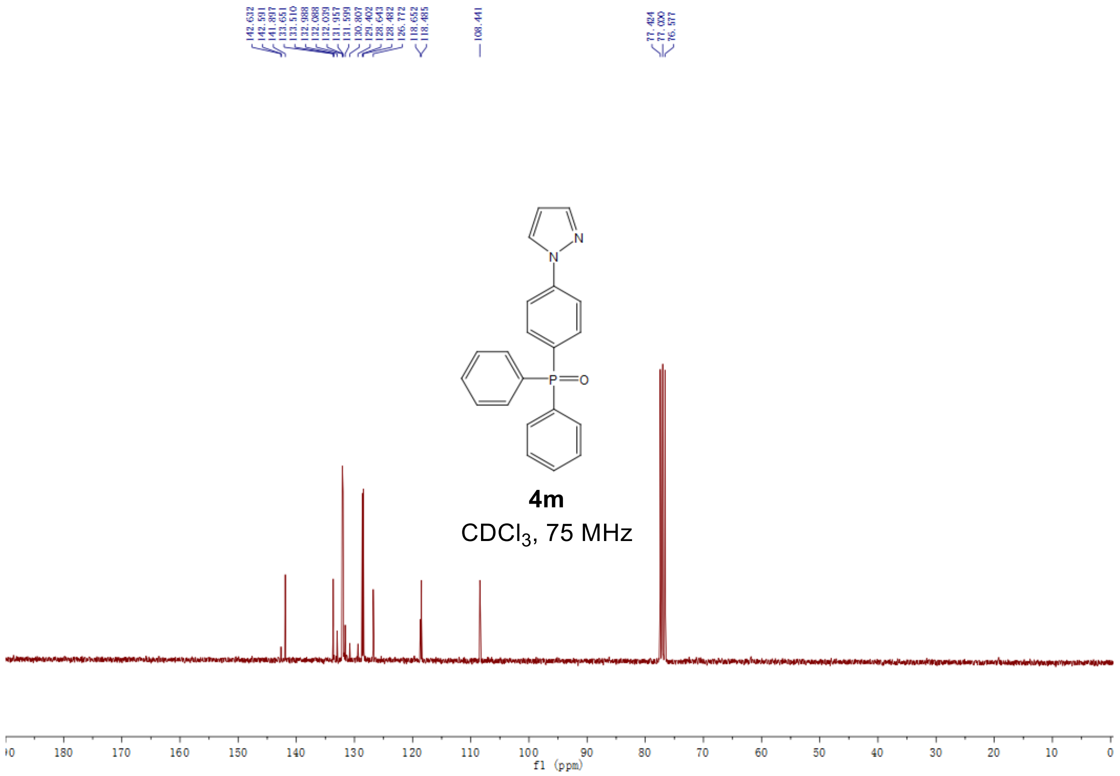
142.632
142.591
141.897
133.651
133.510
132.988
132.088
132.039
131.957
131.599
130.807
129.402
128.643
128.482
126.772
118.652
118.485
108.441
77.424
77.000
76.577
N
N
P
O
4m
CDCl3, 75 MHz
180 170 160 150 140 130 120 110 100 90 80 70 60 50 40 30 20 10 0
f1 (ppm)

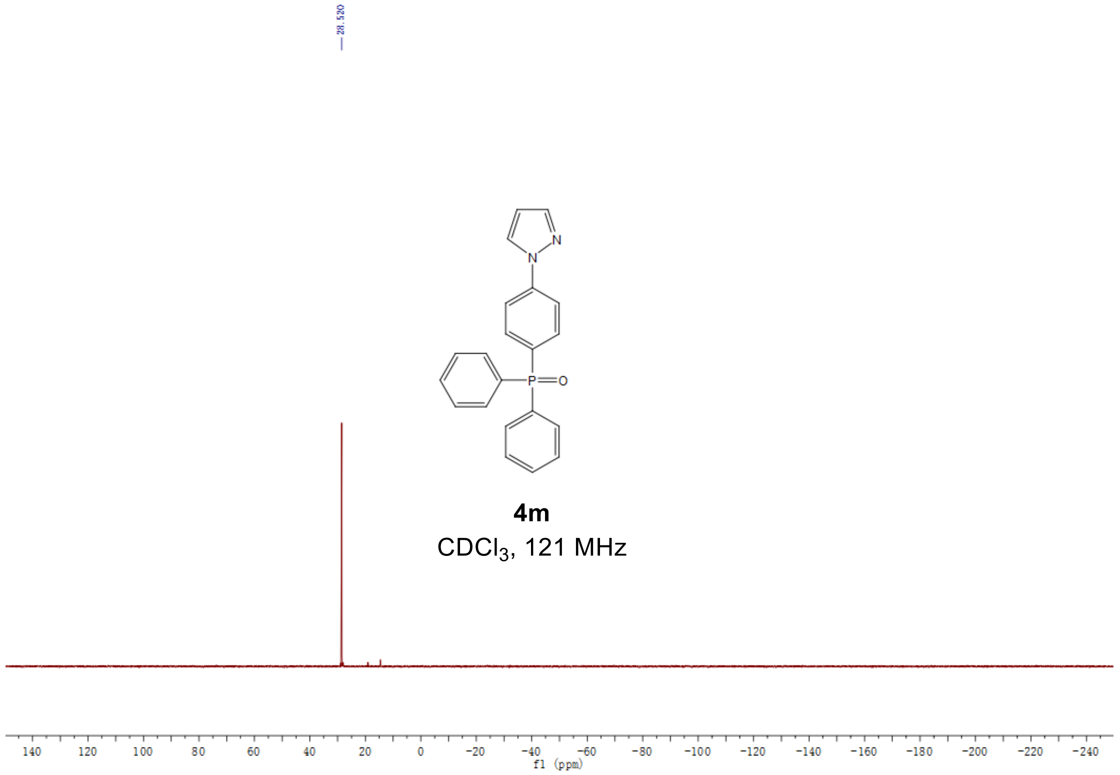
28.520
N
N
P
O
4m
CDCl3, 121 MHz
140 120 100 80 60 40 20 0 −20 −40 −60 −80 −100 −120 −140 −160 −180 −200 −220 −240
f1 (ppm)

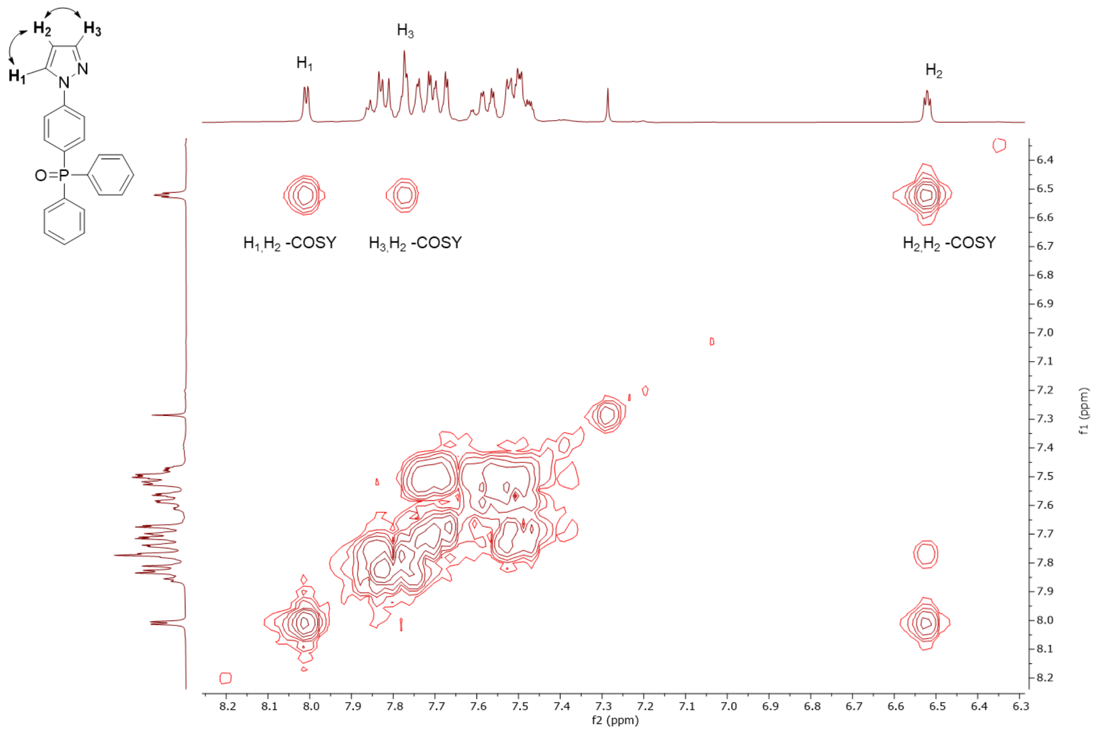

(4-(4,4-Dimethyl-4,5-dihydrooxazol-2-yl)phenyl)diphenylphosphine oxide (**4n**)

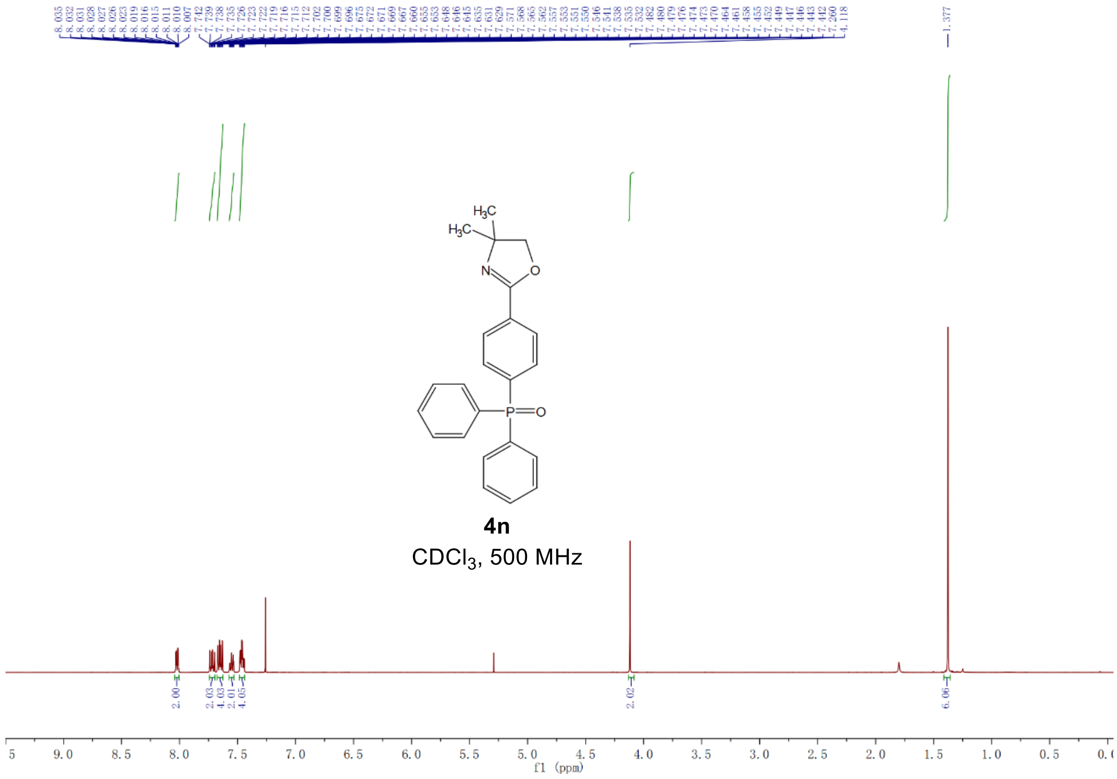

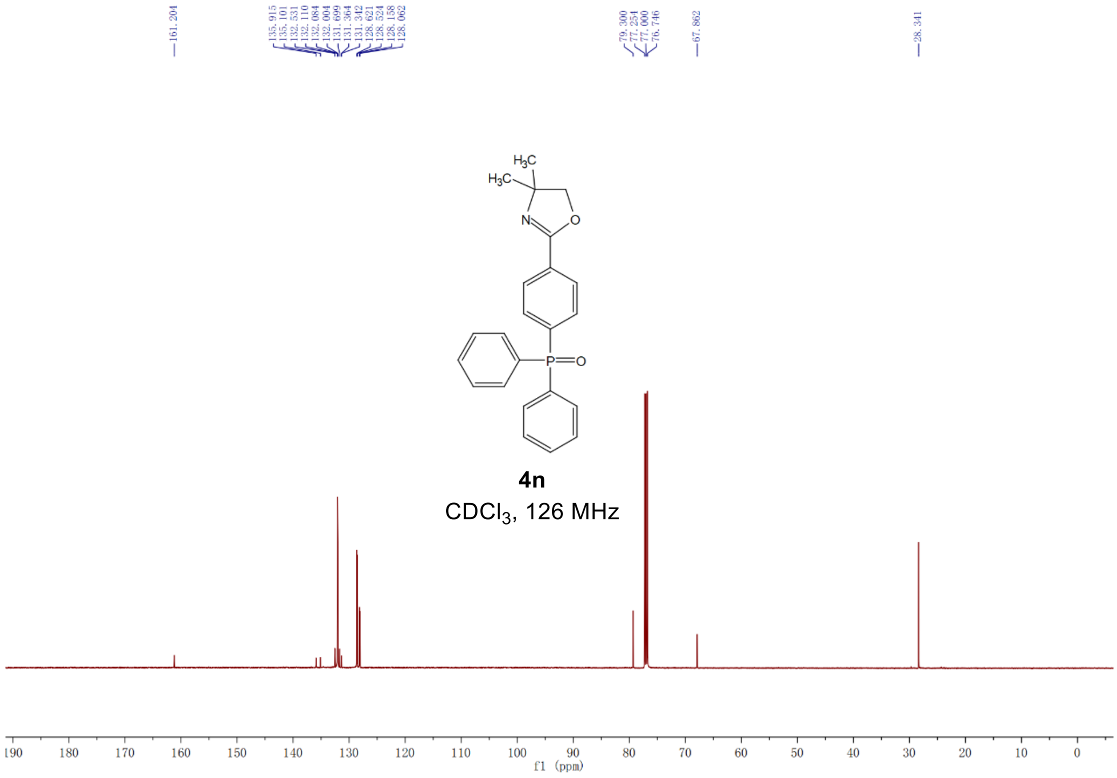
161.204
135.915
135.101
132.531
132.110
132.084
132.004
131.699
131.364
131.342
128.621
128.524
128.158
128.062
79.300
77.254
77.000
76.746
67.862
28.341
4n
CDCl3, 126 MHz
f1 (ppm)

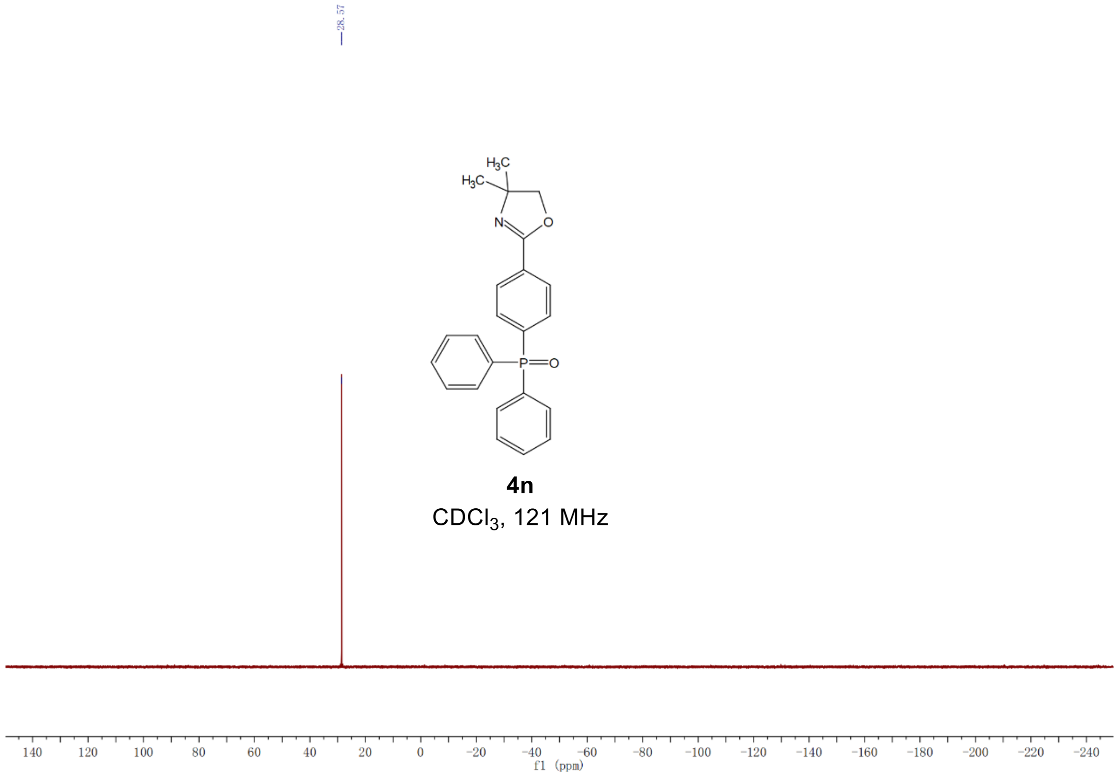
28.57
4n
CDCl3, 121 MHz
f1 (ppm)

Diphenyl(4-(pyridin-2-yl)phenyl)phosphine oxide (**4o**)

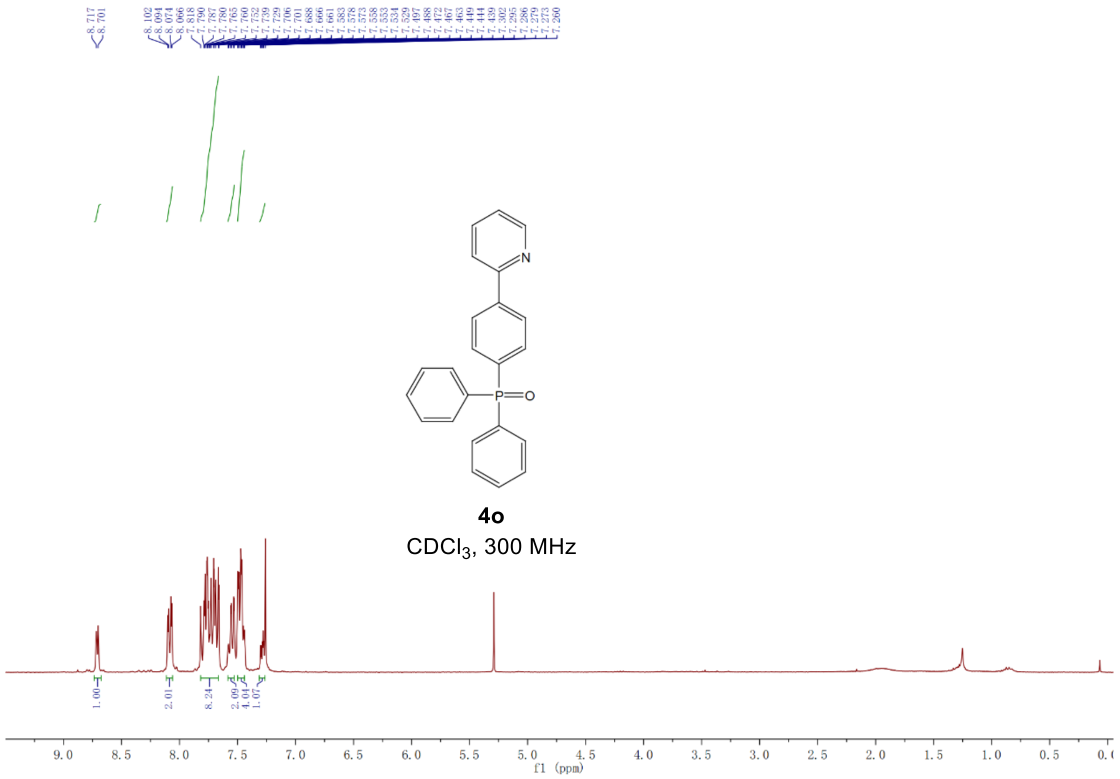

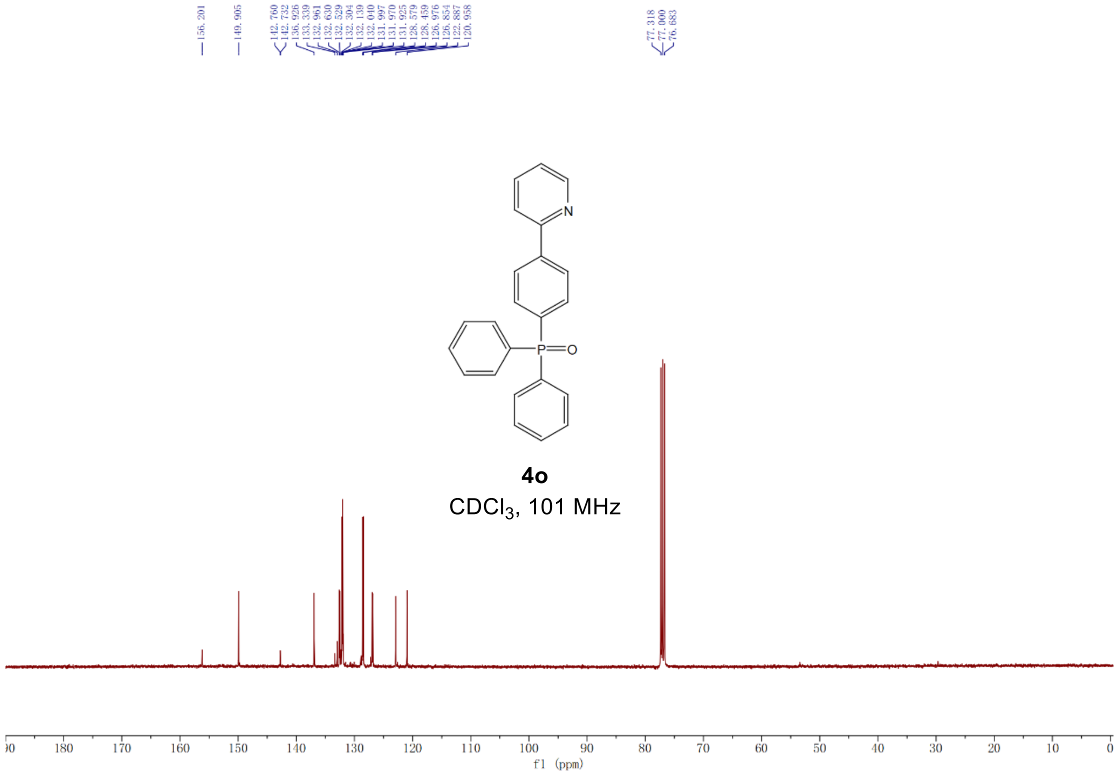

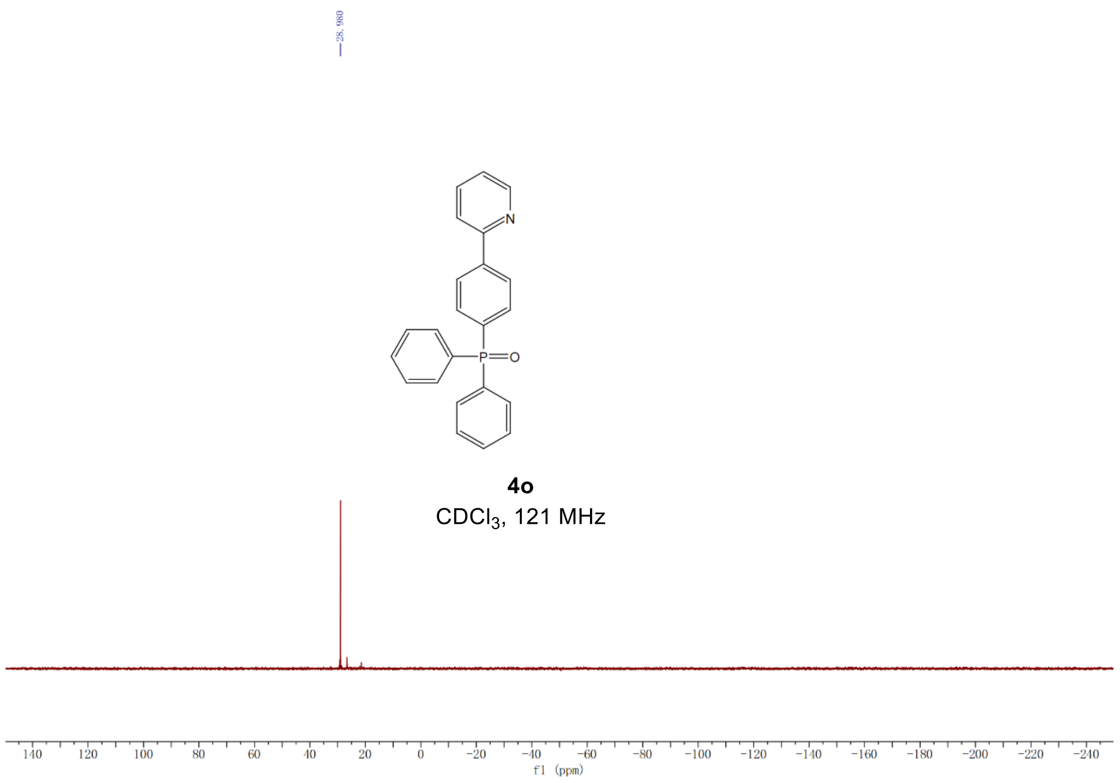

(4-(5-Methylpyrimidin-2-yl)phenyl)diphenylphosphine oxide (**4p**)

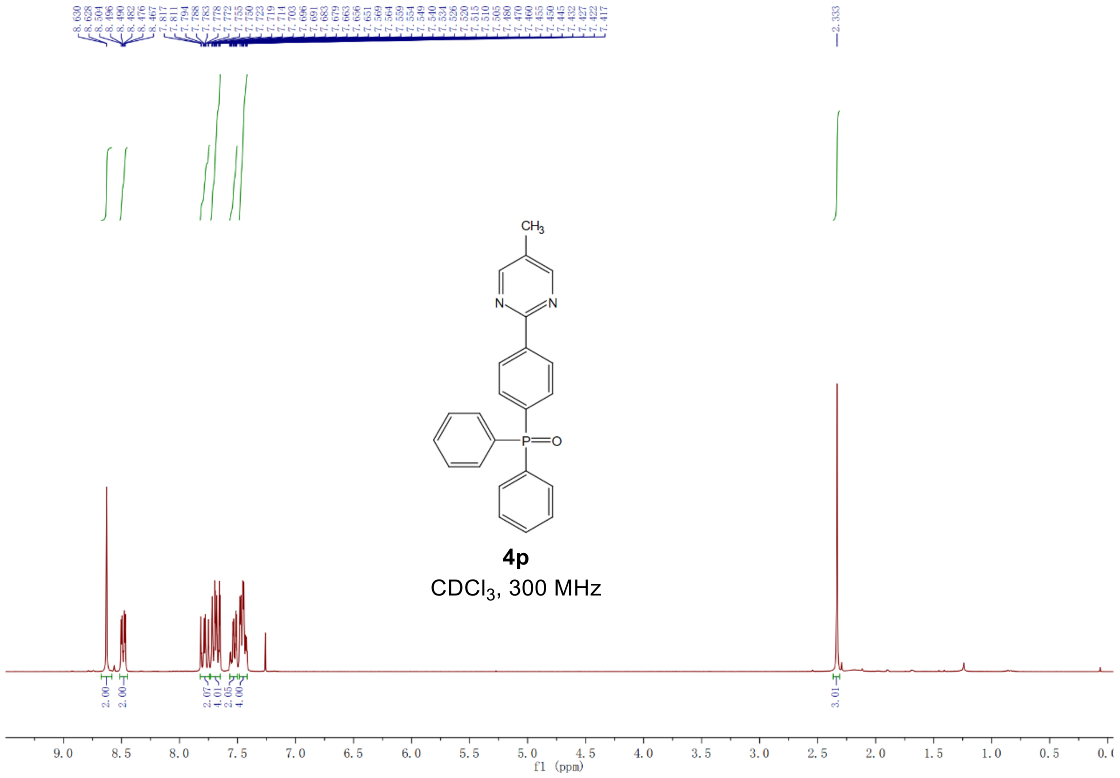

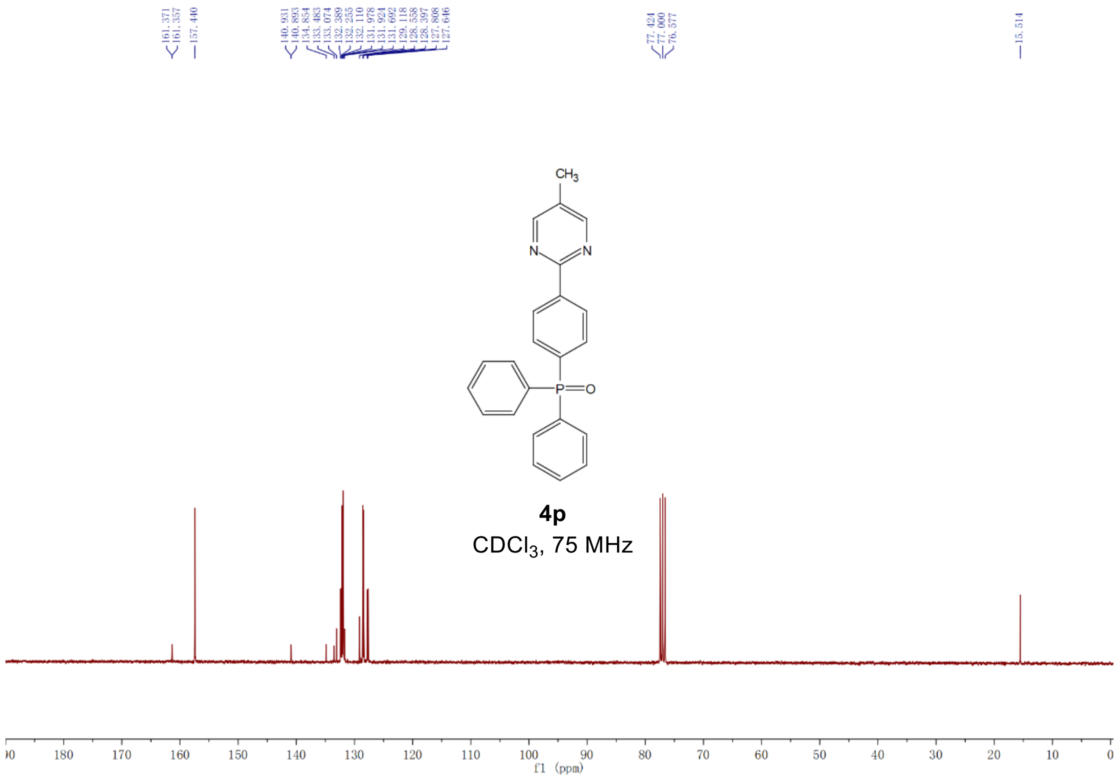
4p
CDCl3, 75 MHz
f1 (ppm)

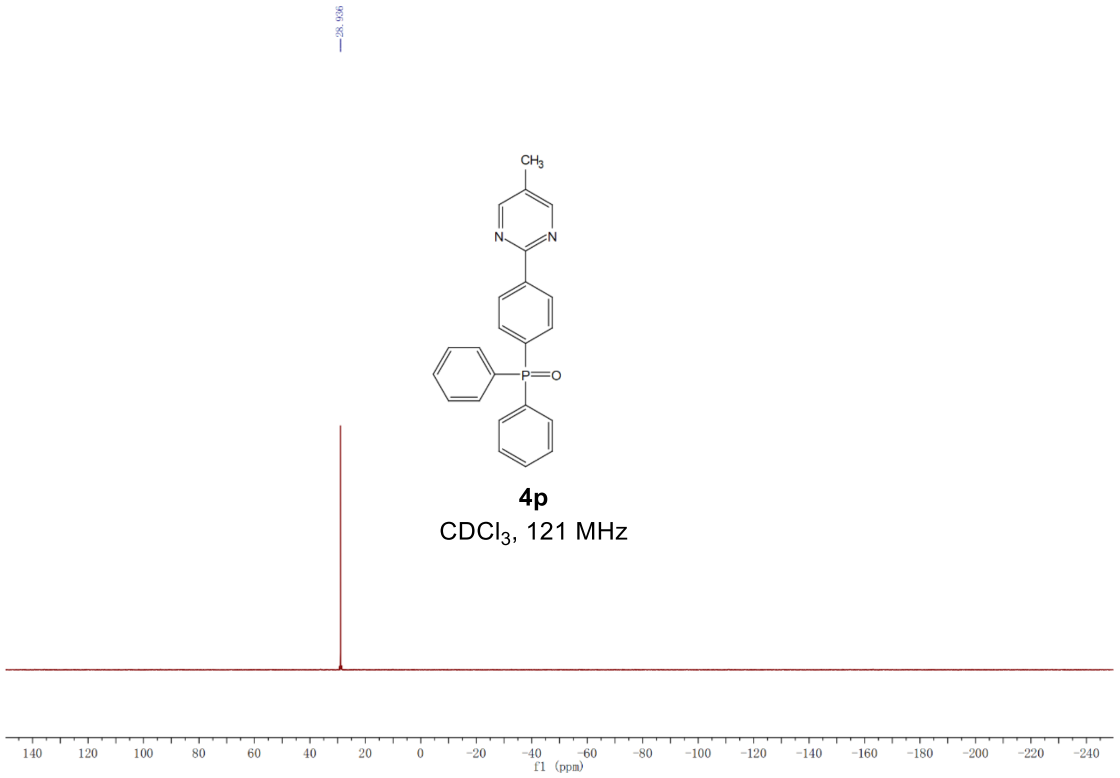
28.936
4p
CDCl3, 121 MHz
f1 (ppm)

(4-(2-Methyl-2H-tetrazol-5-yl)phenyl)diphenylphosphine oxide (**4q**)

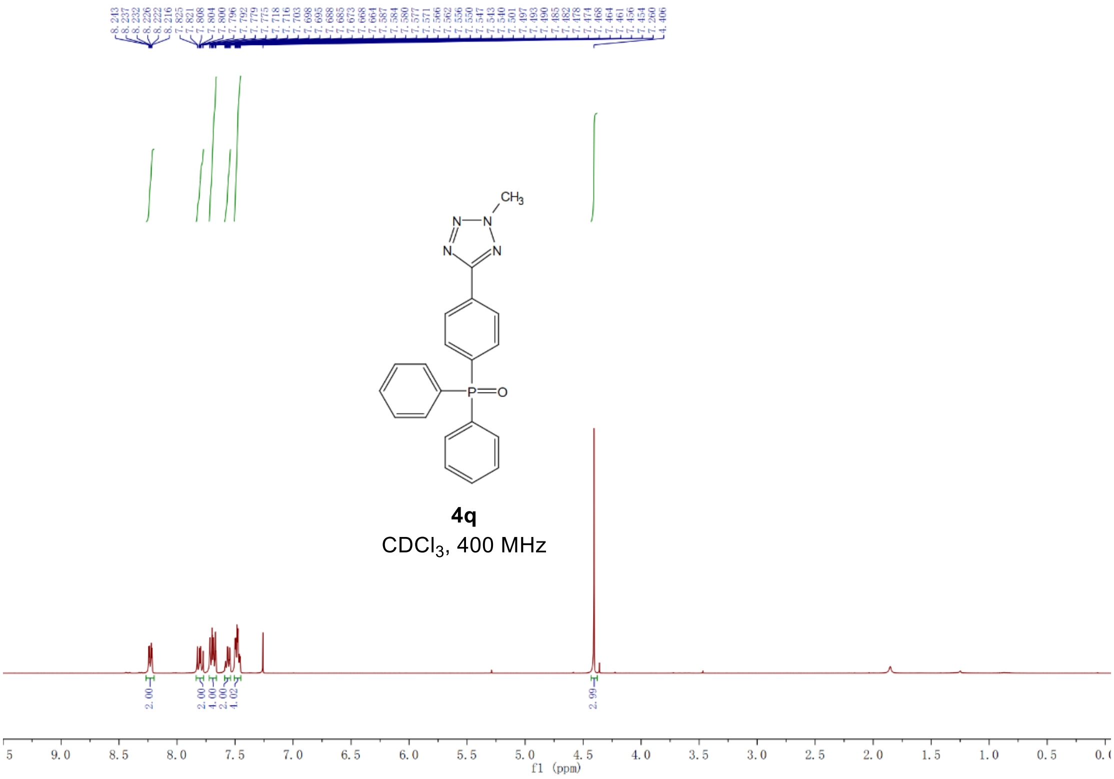

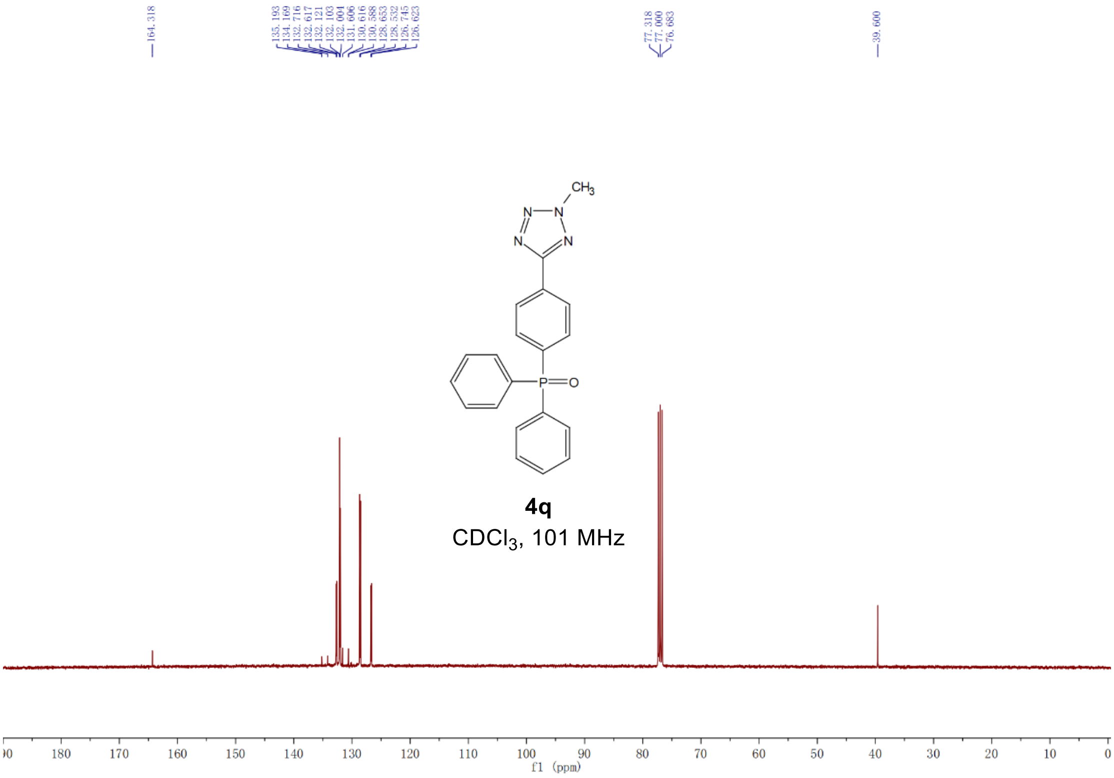

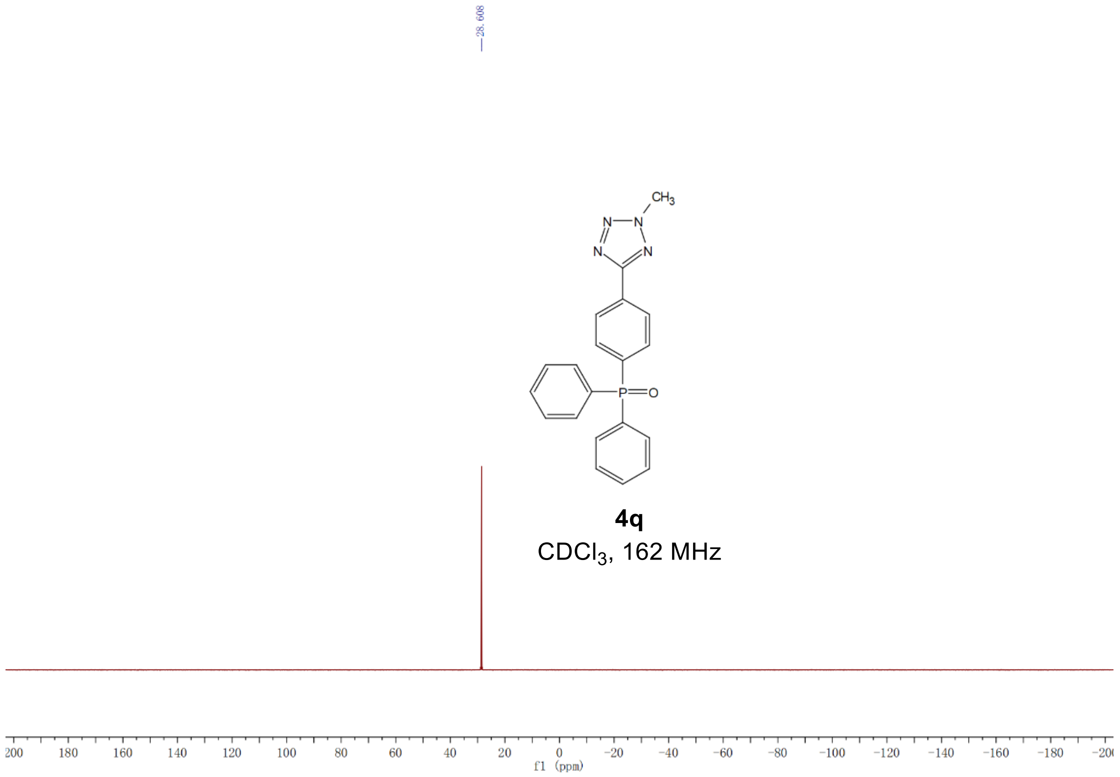

(4-(9-((3aR,4R,6R,6aR)-6-(Hydroxymethyl)-2,2-dimethyltetrahydrofuro[3,4-d][1,3]dioxol-4-yl)-9H-purin-6-yl)phenyl)diphenylphosphine oxide (**4r**)

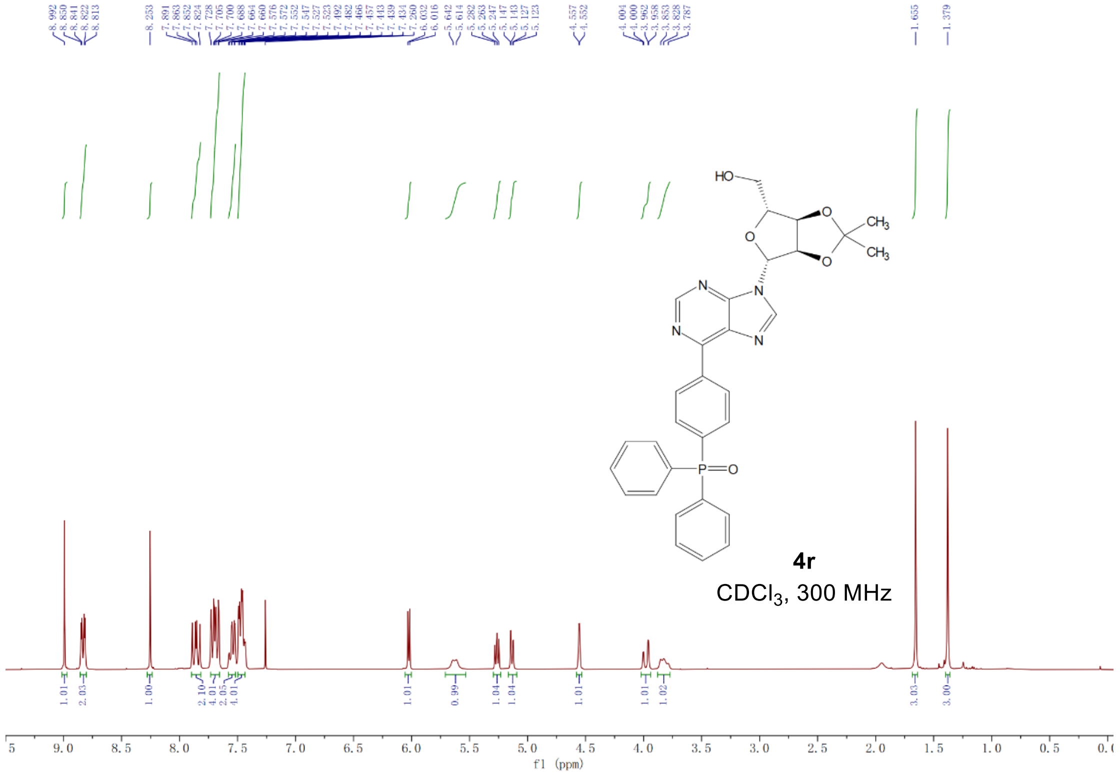

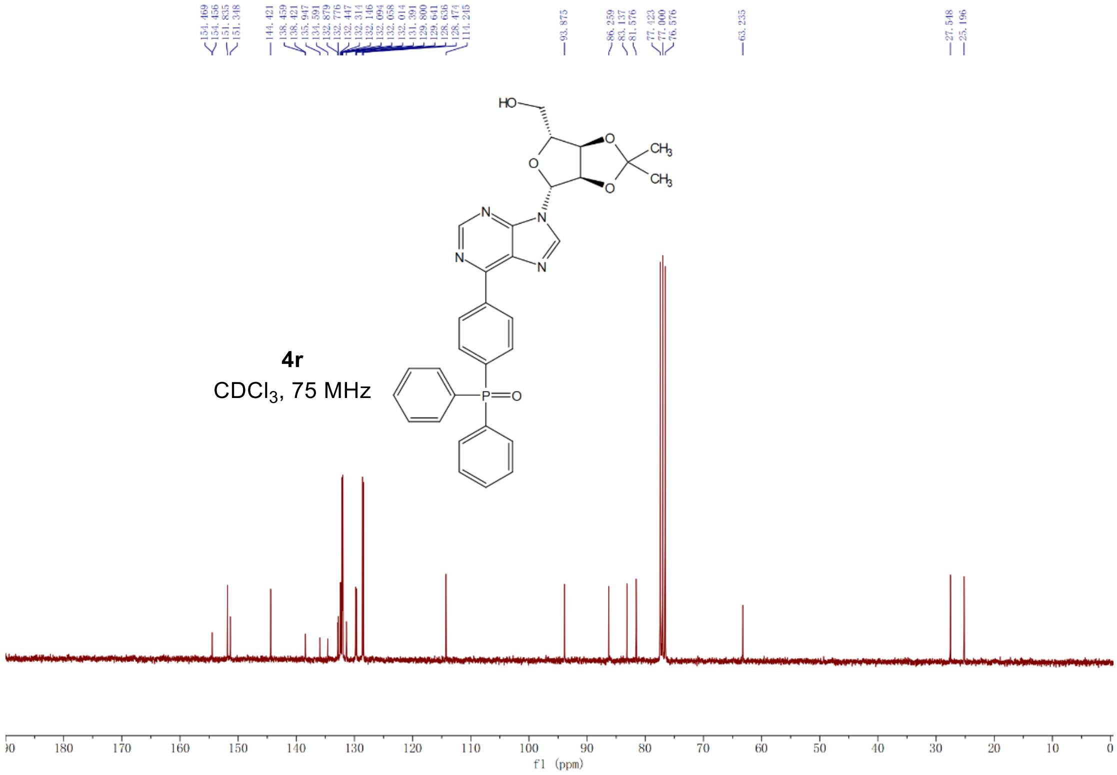
HO
O
O
O
CH3
CH3
N
N
N
N
P=O
4r
CDCl3, 75 MHz
f1 (ppm)

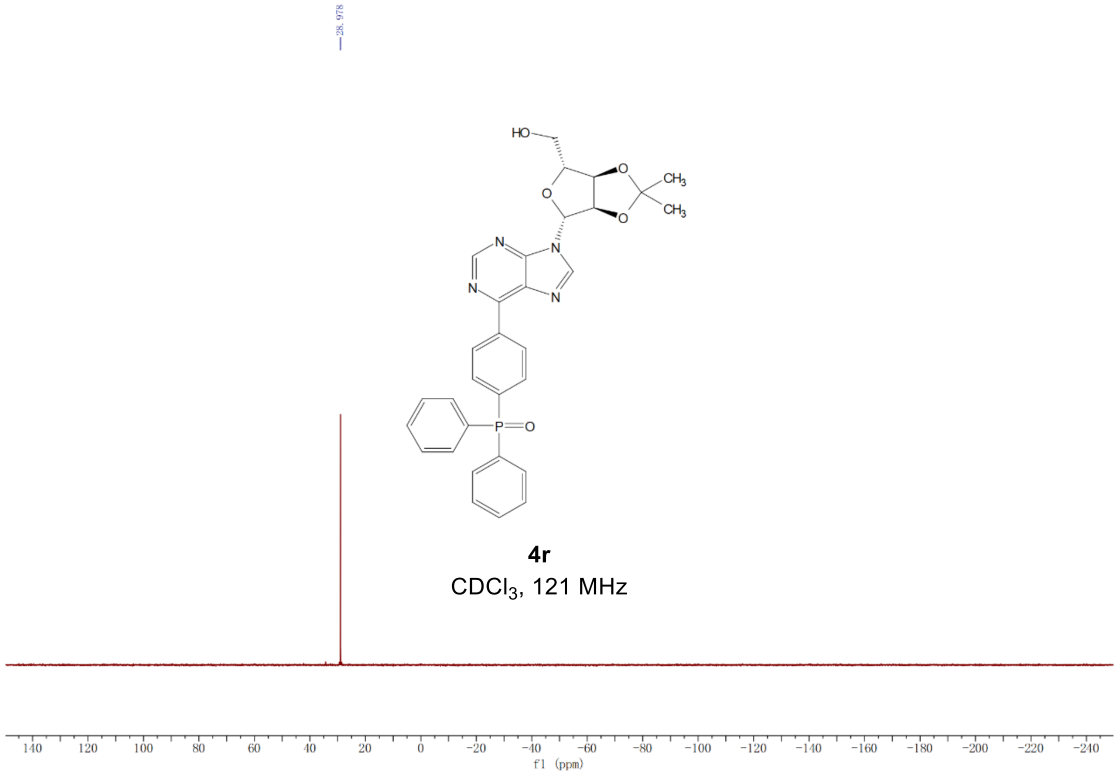
28.978
HO
O
O
O
CH3
CH3
N
N
N
N
P=O
4r
CDCl3, 121 MHz
f1 (ppm)
